# Supplementary material for: Time-series transcriptome analysis identified differentially expressed genes in broiler chicken infected with mixed Eimeria species
Source: Front Genet. 2022 Aug 8;13:886781. doi: 10.3389/fgene.2022.886781 (PMC9393255; doi:10.3389/fgene.2022.886781)
Supplement: Supplementary file 2 [file DataSheet1.ZIP › 7dpi_GO.Gsea.1625106495923/gsea_report_for_1_1625106495923.html]

Report for 1 1625106495923 [GSEA]

| GS  follow link to MSigDB | GS DETAILS | SIZE | ES | NES | NOM p-val | FDR q-val | FWER p-val | RANK AT MAX | LEADING EDGE || 1 | GOCC\_RESPIRASOME | Details ... | 65 | 0.78 | 3.67 | 0.000 | 0.000 | 0.000 | 1395 | tags=71%, list=12%, signal=80% |
| 2 | GOBP\_ATP\_SYNTHESIS\_COUPLED\_ELECTRON\_TRANSPORT | Details ... | 71 | 0.78 | 3.66 | 0.000 | 0.000 | 0.000 | 1995 | tags=76%, list=17%, signal=91% |
| 3 | GOCC\_RESPIRATORY\_CHAIN\_COMPLEX | Details ... | 56 | 0.81 | 3.66 | 0.000 | 0.000 | 0.000 | 1395 | tags=75%, list=12%, signal=84% |
| 4 | GOBP\_OXIDATIVE\_PHOSPHORYLATION | Details ... | 85 | 0.73 | 3.56 | 0.000 | 0.000 | 0.000 | 1395 | tags=61%, list=12%, signal=69% |
| 5 | GOBP\_RESPIRATORY\_ELECTRON\_TRANSPORT\_CHAIN | Details ... | 85 | 0.72 | 3.53 | 0.000 | 0.000 | 0.000 | 1395 | tags=61%, list=12%, signal=69% |
| 6 | GOBP\_MITOCHONDRIAL\_ELECTRON\_TRANSPORT\_NADH\_TO\_UBIQUINONE | Details ... | 42 | 0.81 | 3.52 | 0.000 | 0.000 | 0.000 | 1395 | tags=71%, list=12%, signal=81% |
| 7 | GOCC\_INNER\_MITOCHONDRIAL\_MEMBRANE\_PROTEIN\_COMPLEX | Details ... | 93 | 0.71 | 3.51 | 0.000 | 0.000 | 0.000 | 1455 | tags=58%, list=12%, signal=66% |
| 8 | GOCC\_NADH\_DEHYDROGENASE\_COMPLEX | Details ... | 37 | 0.82 | 3.40 | 0.000 | 0.000 | 0.000 | 1395 | tags=76%, list=12%, signal=85% |
| 9 | GOCC\_MITOCHONDRIAL\_PROTEIN\_CONTAINING\_COMPLEX | Details ... | 196 | 0.61 | 3.35 | 0.000 | 0.000 | 0.000 | 2363 | tags=63%, list=20%, signal=77% |
| 10 | GOMF\_STRUCTURAL\_CONSTITUENT\_OF\_RIBOSOME | Details ... | 133 | 0.63 | 3.30 | 0.000 | 0.000 | 0.000 | 3240 | tags=74%, list=27%, signal=100% |
| 11 | GOMF\_NAD\_P\_H\_DEHYDROGENASE\_QUINONE\_ACTIVITY | Details ... | 38 | 0.79 | 3.29 | 0.000 | 0.000 | 0.000 | 1395 | tags=74%, list=12%, signal=83% |
| 12 | GOBP\_NADH\_DEHYDROGENASE\_COMPLEX\_ASSEMBLY | Details ... | 51 | 0.73 | 3.23 | 0.000 | 0.000 | 0.000 | 1497 | tags=65%, list=12%, signal=74% |
| 13 | GOCC\_ORGANELLAR\_RIBOSOME | Details ... | 77 | 0.66 | 3.19 | 0.000 | 0.000 | 0.000 | 2783 | tags=75%, list=23%, signal=97% |
| 14 | GOBP\_MITOCHONDRIAL\_RESPIRATORY\_CHAIN\_COMPLEX\_ASSEMBLY | Details ... | 79 | 0.66 | 3.18 | 0.000 | 0.000 | 0.000 | 2298 | tags=65%, list=19%, signal=79% |
| 15 | GOCC\_LARGE\_RIBOSOMAL\_SUBUNIT | Details ... | 95 | 0.62 | 3.09 | 0.000 | 0.000 | 0.000 | 3152 | tags=76%, list=26%, signal=102% |
| 16 | GOBP\_MITOCHONDRIAL\_TRANSLATIONAL\_TERMINATION | Details ... | 78 | 0.64 | 3.09 | 0.000 | 0.000 | 0.000 | 2783 | tags=76%, list=23%, signal=98% |
| 17 | GOCC\_PROTON\_TRANSPORTING\_V\_TYPE\_ATPASE\_COMPLEX | Details ... | 21 | 0.87 | 3.08 | 0.000 | 0.000 | 0.000 | 102 | tags=52%, list=1%, signal=53% |
| 18 | GOCC\_PROTON\_TRANSPORTING\_TWO\_SECTOR\_ATPASE\_COMPLEX | Details ... | 24 | 0.84 | 3.08 | 0.000 | 0.000 | 0.000 | 102 | tags=50%, list=1%, signal=50% |
| 19 | GOBP\_PHAGOSOME\_ACIDIFICATION | Details ... | 21 | 0.86 | 3.07 | 0.000 | 0.000 | 0.000 | 102 | tags=48%, list=1%, signal=48% |
| 20 | GOCC\_RIBOSOMAL\_SUBUNIT | Details ... | 155 | 0.58 | 3.06 | 0.000 | 0.000 | 0.000 | 3085 | tags=68%, list=26%, signal=91% |
| 21 | GOMF\_ATPASE\_ACTIVITY\_COUPLED\_TO\_TRANSMEMBRANE\_MOVEMENT\_OF\_IONS\_ROTATIONAL\_MECHANISM |  | 19 | 0.89 | 3.04 | 0.000 | 0.000 | 0.000 | 102 | tags=53%, list=1%, signal=53% |
| 22 | GOMF\_OXIDOREDUCTASE\_ACTIVITY\_ACTING\_ON\_NAD\_P\_H\_QUINONE\_OR\_SIMILAR\_COMPOUND\_AS\_ACCEPTOR |  | 45 | 0.70 | 3.03 | 0.000 | 0.000 | 0.000 | 1395 | tags=64%, list=12%, signal=73% |
| 23 | GOCC\_MITOCHONDRIAL\_LARGE\_RIBOSOMAL\_SUBUNIT |  | 50 | 0.66 | 2.97 | 0.000 | 0.000 | 0.000 | 2363 | tags=74%, list=20%, signal=92% |
| 24 | GOBP\_ELECTRON\_TRANSPORT\_CHAIN |  | 121 | 0.57 | 2.95 | 0.000 | 0.000 | 0.000 | 1464 | tags=51%, list=12%, signal=58% |
| 25 | GOBP\_TRANSFERRIN\_TRANSPORT |  | 27 | 0.77 | 2.91 | 0.000 | 0.000 | 0.000 | 363 | tags=44%, list=3%, signal=46% |
| 26 | GOBP\_CELLULAR\_RESPIRATION |  | 138 | 0.55 | 2.87 | 0.000 | 0.000 | 0.000 | 1395 | tags=45%, list=12%, signal=50% |
| 27 | GOCC\_OXIDOREDUCTASE\_COMPLEX |  | 82 | 0.58 | 2.83 | 0.000 | 0.000 | 0.000 | 1413 | tags=51%, list=12%, signal=58% |
| 28 | GOMF\_ELECTRON\_TRANSFER\_ACTIVITY |  | 91 | 0.56 | 2.82 | 0.000 | 0.000 | 0.000 | 1464 | tags=54%, list=12%, signal=61% |
| 29 | GOBP\_TRANSLATIONAL\_TERMINATION |  | 88 | 0.57 | 2.82 | 0.000 | 0.000 | 0.000 | 2783 | tags=68%, list=23%, signal=88% |
| 30 | GOBP\_MITOCHONDRIAL\_TRANSLATION |  | 114 | 0.55 | 2.79 | 0.000 | 0.000 | 0.000 | 2682 | tags=62%, list=22%, signal=79% |
| 31 | GOCC\_RIBOSOME |  | 185 | 0.50 | 2.74 | 0.000 | 0.000 | 0.000 | 3085 | tags=63%, list=26%, signal=84% |
| 32 | GOMF\_ATPASE\_COUPLED\_ION\_TRANSMEMBRANE\_TRANSPORTER\_ACTIVITY |  | 36 | 0.65 | 2.67 | 0.000 | 0.000 | 0.000 | 102 | tags=31%, list=1%, signal=31% |
| 33 | GOBP\_PHAGOSOME\_MATURATION |  | 37 | 0.65 | 2.64 | 0.000 | 0.000 | 0.000 | 689 | tags=35%, list=6%, signal=37% |
| 34 | GOBP\_PH\_REDUCTION |  | 45 | 0.60 | 2.64 | 0.000 | 0.000 | 0.000 | 405 | tags=33%, list=3%, signal=34% |
| 35 | GOCC\_CYTOCHROME\_COMPLEX |  | 18 | 0.77 | 2.61 | 0.000 | 0.000 | 0.000 | 2326 | tags=83%, list=19%, signal=103% |
| 36 | GOCC\_VACUOLAR\_PROTON\_TRANSPORTING\_V\_TYPE\_ATPASE\_COMPLEX |  | 13 | 0.85 | 2.57 | 0.000 | 0.000 | 0.001 | 102 | tags=46%, list=1%, signal=46% |
| 37 | GOCC\_MITOCHONDRIAL\_SMALL\_RIBOSOMAL\_SUBUNIT |  | 25 | 0.69 | 2.56 | 0.000 | 0.000 | 0.001 | 2649 | tags=68%, list=22%, signal=87% |
| 38 | GOBP\_MITOCHONDRIAL\_GENE\_EXPRESSION |  | 137 | 0.48 | 2.56 | 0.000 | 0.000 | 0.001 | 2783 | tags=58%, list=23%, signal=75% |
| 39 | GOCC\_CYTOSOLIC\_LARGE\_RIBOSOMAL\_SUBUNIT |  | 44 | 0.59 | 2.55 | 0.000 | 0.000 | 0.001 | 3487 | tags=75%, list=29%, signal=105% |
| 40 | GOCC\_ORGANELLE\_INNER\_MEMBRANE |  | 390 | 0.41 | 2.55 | 0.000 | 0.000 | 0.001 | 2415 | tags=46%, list=20%, signal=55% |
| 41 | GOBP\_GLUTATHIONE\_DERIVATIVE\_METABOLIC\_PROCESS |  | 14 | 0.79 | 2.48 | 0.000 | 0.000 | 0.004 | 209 | tags=50%, list=2%, signal=51% |
| 42 | GOCC\_PROTON\_TRANSPORTING\_V\_TYPE\_ATPASE\_V0\_DOMAIN |  | 10 | 0.86 | 2.43 | 0.000 | 0.000 | 0.010 | 100 | tags=50%, list=1%, signal=50% |
| 43 | GOMF\_HEME\_COPPER\_TERMINAL\_OXIDASE\_ACTIVITY |  | 13 | 0.78 | 2.40 | 0.000 | 0.000 | 0.016 | 1927 | tags=69%, list=16%, signal=82% |
| 44 | GOBP\_TRANSLATIONAL\_ELONGATION |  | 115 | 0.47 | 2.39 | 0.000 | 0.000 | 0.018 | 2363 | tags=50%, list=20%, signal=61% |
| 45 | GOCC\_PROTON\_TRANSPORTING\_TWO\_SECTOR\_ATPASE\_COMPLEX\_PROTON\_TRANSPORTING\_DOMAIN |  | 11 | 0.82 | 2.39 | 0.000 | 0.000 | 0.018 | 100 | tags=45%, list=1%, signal=46% |
| 46 | GOMF\_OXIDOREDUCTASE\_ACTIVITY\_ACTING\_ON\_NAD\_P\_H |  | 73 | 0.50 | 2.38 | 0.000 | 0.000 | 0.021 | 1468 | tags=44%, list=12%, signal=50% |
| 47 | GOCC\_SMALL\_RIBOSOMAL\_SUBUNIT |  | 62 | 0.50 | 2.34 | 0.000 | 0.001 | 0.040 | 3085 | tags=61%, list=26%, signal=82% |
| 48 | GOCC\_PROTON\_TRANSPORTING\_TWO\_SECTOR\_ATPASE\_COMPLEX\_CATALYTIC\_DOMAIN |  | 7 | 0.94 | 2.33 | 0.000 | 0.001 | 0.051 | 80 | tags=57%, list=1%, signal=57% |
| 49 | GOBP\_MITOCHONDRIAL\_ELECTRON\_TRANSPORT\_UBIQUINOL\_TO\_CYTOCHROME\_C |  | 10 | 0.82 | 2.32 | 0.000 | 0.001 | 0.057 | 1700 | tags=90%, list=14%, signal=105% |
| 50 | GOBP\_IRON\_ION\_TRANSPORT |  | 55 | 0.50 | 2.32 | 0.000 | 0.001 | 0.058 | 363 | tags=24%, list=3%, signal=24% |
| 51 | GOBP\_PROTON\_TRANSMEMBRANE\_TRANSPORT |  | 85 | 0.47 | 2.30 | 0.000 | 0.001 | 0.070 | 1371 | tags=33%, list=11%, signal=37% |
| 52 | GOMF\_GLUTATHIONE\_PEROXIDASE\_ACTIVITY |  | 16 | 0.68 | 2.24 | 0.000 | 0.003 | 0.152 | 190 | tags=38%, list=2%, signal=38% |
| 53 | GOBP\_MITOCHONDRIAL\_ELECTRON\_TRANSPORT\_CYTOCHROME\_C\_TO\_OXYGEN |  | 11 | 0.77 | 2.22 | 0.000 | 0.004 | 0.188 | 1927 | tags=73%, list=16%, signal=87% |
| 54 | GOCC\_RESPIRATORY\_CHAIN\_COMPLEX\_III |  | 9 | 0.81 | 2.22 | 0.001 | 0.004 | 0.204 | 2326 | tags=100%, list=19%, signal=124% |
| 55 | GOCC\_CYTOSOLIC\_RIBOSOME |  | 85 | 0.45 | 2.20 | 0.000 | 0.004 | 0.229 | 3146 | tags=59%, list=26%, signal=79% |
| 56 | GOCC\_PROTON\_TRANSPORTING\_V\_TYPE\_ATPASE\_V1\_DOMAIN |  | 6 | 0.92 | 2.16 | 0.000 | 0.007 | 0.353 | 80 | tags=50%, list=1%, signal=50% |
| 57 | GOBP\_CELLULAR\_RESPONSE\_TO\_INTERFERON\_BETA |  | 11 | 0.74 | 2.16 | 0.002 | 0.007 | 0.356 | 1309 | tags=45%, list=11%, signal=51% |
| 58 | GOCC\_PROTEASOME\_CORE\_COMPLEX |  | 13 | 0.70 | 2.15 | 0.000 | 0.008 | 0.382 | 3533 | tags=92%, list=29%, signal=131% |
| 59 | GOBP\_ATP\_METABOLIC\_PROCESS |  | 201 | 0.38 | 2.15 | 0.000 | 0.008 | 0.392 | 1395 | tags=33%, list=12%, signal=37% |
| 60 | GOBP\_REGULATION\_OF\_PH |  | 79 | 0.44 | 2.10 | 0.000 | 0.013 | 0.581 | 405 | tags=23%, list=3%, signal=23% |
| 61 | GOBP\_CELLULAR\_MONOVALENT\_INORGANIC\_CATION\_HOMEOSTASIS |  | 86 | 0.43 | 2.10 | 0.000 | 0.013 | 0.591 | 405 | tags=22%, list=3%, signal=23% |
| 62 | GOBP\_MONOVALENT\_INORGANIC\_CATION\_HOMEOSTASIS |  | 108 | 0.41 | 2.10 | 0.000 | 0.014 | 0.611 | 405 | tags=20%, list=3%, signal=21% |
| 63 | GOBP\_VACUOLAR\_ACIDIFICATION |  | 21 | 0.59 | 2.09 | 0.000 | 0.015 | 0.634 | 664 | tags=33%, list=6%, signal=35% |
| 64 | GOMF\_PROTON\_TRANSMEMBRANE\_TRANSPORTER\_ACTIVITY |  | 72 | 0.45 | 2.09 | 0.000 | 0.015 | 0.637 | 1125 | tags=32%, list=9%, signal=35% |
| 65 | GOBP\_ENERGY\_DERIVATION\_BY\_OXIDATION\_OF\_ORGANIC\_COMPOUNDS |  | 198 | 0.38 | 2.07 | 0.000 | 0.017 | 0.691 | 1445 | tags=34%, list=12%, signal=38% |
| 66 | GOCC\_RESPIRATORY\_CHAIN\_COMPLEX\_IV |  | 8 | 0.79 | 2.07 | 0.000 | 0.017 | 0.701 | 1700 | tags=75%, list=14%, signal=87% |
| 67 | GOBP\_REGULATION\_OF\_INFLAMMATORY\_RESPONSE\_TO\_ANTIGENIC\_STIMULUS |  | 12 | 0.69 | 2.07 | 0.002 | 0.017 | 0.701 | 1445 | tags=33%, list=12%, signal=38% |
| 68 | GOMF\_GLUTATHIONE\_TRANSFERASE\_ACTIVITY |  | 14 | 0.66 | 2.07 | 0.000 | 0.017 | 0.708 | 209 | tags=43%, list=2%, signal=44% |
| 69 | GOBP\_CYTOCHROME\_COMPLEX\_ASSEMBLY |  | 25 | 0.54 | 2.04 | 0.000 | 0.023 | 0.813 | 2548 | tags=52%, list=21%, signal=66% |
| 70 | GOBP\_POSITIVE\_REGULATION\_OF\_EPIDERMAL\_GROWTH\_FACTOR\_ACTIVATED\_RECEPTOR\_ACTIVITY |  | 9 | 0.75 | 2.03 | 0.002 | 0.025 | 0.849 | 450 | tags=33%, list=4%, signal=35% |
| 71 | GOBP\_RESPONSE\_TO\_INTERFERON\_BETA |  | 14 | 0.65 | 2.03 | 0.002 | 0.025 | 0.850 | 1309 | tags=43%, list=11%, signal=48% |
| 72 | GOBP\_RESPIRATORY\_CHAIN\_COMPLEX\_IV\_ASSEMBLY |  | 17 | 0.60 | 2.03 | 0.002 | 0.025 | 0.853 | 2461 | tags=53%, list=20%, signal=66% |
| 73 | GOMF\_ANTIOXIDANT\_ACTIVITY |  | 52 | 0.45 | 2.03 | 0.000 | 0.024 | 0.855 | 1959 | tags=37%, list=16%, signal=43% |
| 74 | GOCC\_MITOCHONDRIAL\_MATRIX |  | 364 | 0.34 | 2.01 | 0.000 | 0.029 | 0.902 | 2109 | tags=35%, list=18%, signal=42% |
| 75 | GOBP\_POSITIVE\_REGULATION\_OF\_BICELLULAR\_TIGHT\_JUNCTION\_ASSEMBLY |  | 6 | 0.84 | 2.01 | 0.002 | 0.030 | 0.914 | 1030 | tags=50%, list=9%, signal=55% |
| 76 | GOMF\_PROTEIN\_TAG |  | 9 | 0.74 | 2.00 | 0.004 | 0.032 | 0.928 | 2862 | tags=78%, list=24%, signal=102% |
| 77 | GOBP\_TRANSITION\_METAL\_ION\_TRANSPORT |  | 87 | 0.40 | 2.00 | 0.000 | 0.033 | 0.938 | 382 | tags=17%, list=3%, signal=18% |
| 78 | GOBP\_AEROBIC\_RESPIRATION |  | 64 | 0.42 | 1.97 | 0.000 | 0.042 | 0.973 | 1992 | tags=41%, list=17%, signal=48% |
| 79 | GOCC\_MITOCHONDRIAL\_RESPIRATORY\_CHAIN\_COMPLEX\_IV |  | 7 | 0.78 | 1.97 | 0.004 | 0.043 | 0.975 | 1700 | tags=71%, list=14%, signal=83% |
| 80 | GOCC\_PREFOLDIN\_COMPLEX |  | 5 | 0.89 | 1.97 | 0.001 | 0.043 | 0.976 | 1350 | tags=100%, list=11%, signal=113% |
| 81 | GOMF\_NUCLEOSIDE\_DIPHOSPHATE\_KINASE\_ACTIVITY |  | 10 | 0.70 | 1.97 | 0.003 | 0.043 | 0.977 | 1965 | tags=60%, list=16%, signal=72% |
| 82 | GOMF\_UBIQUINOL\_CYTOCHROME\_C\_REDUCTASE\_ACTIVITY |  | 6 | 0.83 | 1.96 | 0.003 | 0.043 | 0.980 | 1263 | tags=83%, list=11%, signal=93% |
| 83 | GOBP\_NEGATIVE\_REGULATION\_OF\_VIRAL\_PROCESS |  | 38 | 0.47 | 1.96 | 0.000 | 0.045 | 0.982 | 758 | tags=29%, list=6%, signal=31% |
| 84 | GOBP\_NEGATIVE\_REGULATION\_OF\_AMYLOID\_FIBRIL\_FORMATION |  | 7 | 0.79 | 1.95 | 0.003 | 0.048 | 0.987 | 1091 | tags=57%, list=9%, signal=63% |
| 85 | GOMF\_INTRAMOLECULAR\_OXIDOREDUCTASE\_ACTIVITY\_TRANSPOSING\_C\_C\_BONDS |  | 6 | 0.83 | 1.95 | 0.004 | 0.048 | 0.988 | 1504 | tags=83%, list=13%, signal=95% |
| 86 | GOBP\_SULFUR\_AMINO\_ACID\_CATABOLIC\_PROCESS |  | 10 | 0.68 | 1.94 | 0.008 | 0.049 | 0.990 | 1955 | tags=60%, list=16%, signal=72% |
| 87 | GOBP\_MDA\_5\_SIGNALING\_PATHWAY |  | 8 | 0.75 | 1.94 | 0.004 | 0.049 | 0.990 | 332 | tags=50%, list=3%, signal=51% |
| 88 | GOBP\_EPITHELIAL\_CELL\_MATURATION |  | 13 | 0.63 | 1.94 | 0.004 | 0.049 | 0.990 | 843 | tags=54%, list=7%, signal=58% |
| 89 | GOBP\_MITOCHONDRIAL\_CYTOCHROME\_C\_OXIDASE\_ASSEMBLY |  | 15 | 0.60 | 1.94 | 0.007 | 0.048 | 0.990 | 2461 | tags=53%, list=20%, signal=67% |
| 90 | GOCC\_INTRINSIC\_COMPONENT\_OF\_MITOCHONDRIAL\_INNER\_MEMBRANE |  | 43 | 0.45 | 1.94 | 0.002 | 0.048 | 0.990 | 2257 | tags=47%, list=19%, signal=57% |
| 91 | GOBP\_CELLULAR\_RESPONSE\_TO\_ALDEHYDE |  | 10 | 0.68 | 1.93 | 0.007 | 0.052 | 0.994 | 1464 | tags=50%, list=12%, signal=57% |
| 92 | GOMF\_PEROXIREDOXIN\_ACTIVITY |  | 5 | 0.88 | 1.93 | 0.002 | 0.052 | 0.994 | 228 | tags=40%, list=2%, signal=41% |
| 93 | GOBP\_RESPONSE\_TO\_XENOBIOTIC\_STIMULUS |  | 60 | 0.42 | 1.92 | 0.000 | 0.059 | 0.998 | 1249 | tags=30%, list=10%, signal=33% |
| 94 | GOMF\_SUCCINATE\_DEHYDROGENASE\_ACTIVITY |  | 5 | 0.88 | 1.92 | 0.003 | 0.059 | 0.998 | 1135 | tags=80%, list=9%, signal=88% |
| 95 | GOCC\_RESPIRATORY\_CHAIN\_COMPLEX\_II |  | 5 | 0.88 | 1.92 | 0.001 | 0.059 | 0.998 | 1135 | tags=80%, list=9%, signal=88% |
| 96 | GOBP\_3\_PHOSPHOADENOSINE\_5\_PHOSPHOSULFATE\_METABOLIC\_PROCESS |  | 12 | 0.64 | 1.92 | 0.006 | 0.059 | 0.998 | 2329 | tags=50%, list=19%, signal=62% |
| 97 | GOMF\_PHOSPHOLIPASE\_A2\_ACTIVITY\_CONSUMING\_1\_2\_DIPALMITOYLPHOSPHATIDYLCHOLINE |  | 9 | 0.70 | 1.91 | 0.007 | 0.059 | 0.999 | 299 | tags=44%, list=2%, signal=46% |
| 98 | GOMF\_TRANSFERASE\_ACTIVITY\_TRANSFERRING\_ALKYL\_OR\_ARYL\_OTHER\_THAN\_METHYL\_GROUPS |  | 42 | 0.45 | 1.90 | 0.000 | 0.070 | 1.000 | 810 | tags=29%, list=7%, signal=31% |
| 99 | GOBP\_CELL\_REDOX\_HOMEOSTASIS |  | 34 | 0.47 | 1.88 | 0.001 | 0.082 | 1.000 | 1468 | tags=35%, list=12%, signal=40% |
| 100 | GOBP\_SODIUM\_ION\_HOMEOSTASIS |  | 25 | 0.50 | 1.87 | 0.005 | 0.087 | 1.000 | 226 | tags=24%, list=2%, signal=24% |
| 101 | GOBP\_PROTEIN\_IMPORT\_INTO\_MITOCHONDRIAL\_MATRIX |  | 15 | 0.57 | 1.84 | 0.002 | 0.109 | 1.000 | 2491 | tags=60%, list=21%, signal=76% |
| 102 | GOMF\_PROTON\_EXPORTING\_ATPASE\_ACTIVITY\_PHOSPHORYLATIVE\_MECHANISM |  | 6 | 0.79 | 1.84 | 0.011 | 0.114 | 1.000 | 61 | tags=33%, list=1%, signal=33% |
| 103 | GOCC\_TIM23\_MITOCHONDRIAL\_IMPORT\_INNER\_MEMBRANE\_TRANSLOCASE\_COMPLEX |  | 11 | 0.62 | 1.83 | 0.007 | 0.124 | 1.000 | 2491 | tags=64%, list=21%, signal=80% |
| 104 | GOMF\_LIGAND\_GATED\_SODIUM\_CHANNEL\_ACTIVITY |  | 7 | 0.74 | 1.82 | 0.014 | 0.134 | 1.000 | 226 | tags=43%, list=2%, signal=44% |
| 105 | GOBP\_REGULATION\_OF\_HYPERSENSITIVITY |  | 4 | 0.90 | 1.81 | 0.002 | 0.138 | 1.000 | 49 | tags=25%, list=0%, signal=25% |
| 106 | GOMF\_NEDD8\_SPECIFIC\_PROTEASE\_ACTIVITY |  | 6 | 0.77 | 1.80 | 0.010 | 0.144 | 1.000 | 1245 | tags=33%, list=10%, signal=37% |
| 107 | GOMF\_OXIDOREDUCTASE\_ACTIVITY\_ACTING\_ON\_PEROXIDE\_AS\_ACCEPTOR |  | 33 | 0.45 | 1.80 | 0.009 | 0.145 | 1.000 | 228 | tags=21%, list=2%, signal=22% |
| 108 | GOMF\_ACYLGLYCEROL\_LIPASE\_ACTIVITY |  | 9 | 0.66 | 1.80 | 0.014 | 0.144 | 1.000 | 703 | tags=44%, list=6%, signal=47% |
| 109 | GOMF\_SMALL\_RIBOSOMAL\_SUBUNIT\_RRNA\_BINDING |  | 8 | 0.68 | 1.79 | 0.017 | 0.157 | 1.000 | 3050 | tags=88%, list=25%, signal=117% |
| 110 | GOBP\_REGULATION\_OF\_ODONTOGENESIS\_OF\_DENTIN\_CONTAINING\_TOOTH |  | 5 | 0.81 | 1.79 | 0.014 | 0.158 | 1.000 | 1723 | tags=80%, list=14%, signal=93% |
| 111 | GOMF\_RRNA\_BINDING |  | 46 | 0.41 | 1.79 | 0.002 | 0.156 | 1.000 | 2512 | tags=37%, list=21%, signal=47% |
| 112 | GOBP\_HYPERSENSITIVITY |  | 6 | 0.75 | 1.78 | 0.018 | 0.171 | 1.000 | 525 | tags=33%, list=4%, signal=35% |
| 113 | GOCC\_RNA\_POLYMERASE\_II\_CORE\_COMPLEX |  | 10 | 0.62 | 1.77 | 0.016 | 0.178 | 1.000 | 2813 | tags=80%, list=23%, signal=104% |
| 114 | GOBP\_COTRANSLATIONAL\_PROTEIN\_TARGETING\_TO\_MEMBRANE |  | 87 | 0.36 | 1.77 | 0.000 | 0.179 | 1.000 | 3487 | tags=56%, list=29%, signal=79% |
| 115 | GOBP\_NUCLEOTIDE\_SUGAR\_BIOSYNTHETIC\_PROCESS |  | 19 | 0.51 | 1.76 | 0.002 | 0.184 | 1.000 | 685 | tags=37%, list=6%, signal=39% |
| 116 | GOBP\_PROTON\_TRANSPORTING\_ATP\_SYNTHASE\_COMPLEX\_ASSEMBLY |  | 5 | 0.81 | 1.76 | 0.014 | 0.187 | 1.000 | 857 | tags=40%, list=7%, signal=43% |
| 117 | GOBP\_NEGATIVE\_REGULATION\_OF\_VIRAL\_GENOME\_REPLICATION |  | 22 | 0.48 | 1.75 | 0.003 | 0.199 | 1.000 | 758 | tags=32%, list=6%, signal=34% |
| 118 | GOBP\_CELLULAR\_RESPONSE\_TO\_EXOGENOUS\_DSRNA |  | 8 | 0.67 | 1.74 | 0.018 | 0.208 | 1.000 | 969 | tags=38%, list=8%, signal=41% |
| 119 | GOBP\_MITOCHONDRIAL\_RESPIRASOME\_ASSEMBLY |  | 4 | 0.86 | 1.74 | 0.010 | 0.210 | 1.000 | 499 | tags=75%, list=4%, signal=78% |
| 120 | GOBP\_REGULATION\_OF\_CELLULAR\_AMINO\_ACID\_METABOLIC\_PROCESS |  | 45 | 0.40 | 1.74 | 0.005 | 0.211 | 1.000 | 3147 | tags=51%, list=26%, signal=69% |
| 121 | GOMF\_SPHINGOSINE\_1\_PHOSPHATE\_RECEPTOR\_ACTIVITY |  | 5 | 0.79 | 1.73 | 0.020 | 0.220 | 1.000 | 447 | tags=40%, list=4%, signal=42% |
| 122 | GOBP\_PROTEIN\_INSERTION\_INTO\_MITOCHONDRIAL\_INNER\_MEMBRANE |  | 9 | 0.63 | 1.73 | 0.024 | 0.232 | 1.000 | 2341 | tags=67%, list=19%, signal=83% |
| 123 | GOCC\_RIBONUCLEASE\_P\_COMPLEX |  | 8 | 0.66 | 1.72 | 0.021 | 0.233 | 1.000 | 2339 | tags=63%, list=19%, signal=78% |
| 124 | GOMF\_RIBONUCLEASE\_P\_ACTIVITY |  | 6 | 0.73 | 1.72 | 0.022 | 0.232 | 1.000 | 1517 | tags=67%, list=13%, signal=76% |
| 125 | GOMF\_RIBONUCLEASE\_P\_RNA\_BINDING |  | 6 | 0.73 | 1.72 | 0.028 | 0.241 | 1.000 | 1517 | tags=67%, list=13%, signal=76% |
| 126 | GOCC\_MULTIMERIC\_RIBONUCLEASE\_P\_COMPLEX |  | 6 | 0.73 | 1.72 | 0.023 | 0.240 | 1.000 | 1517 | tags=67%, list=13%, signal=76% |
| 127 | GOCC\_PROTEASOME\_CORE\_COMPLEX\_ALPHA\_SUBUNIT\_COMPLEX |  | 7 | 0.69 | 1.71 | 0.023 | 0.246 | 1.000 | 3533 | tags=86%, list=29%, signal=121% |
| 128 | GOBP\_NEGATIVE\_REGULATION\_OF\_RESPIRATORY\_BURST\_INVOLVED\_IN\_INFLAMMATORY\_RESPONSE |  | 4 | 0.86 | 1.71 | 0.013 | 0.246 | 1.000 | 1540 | tags=75%, list=13%, signal=86% |
| 129 | GOBP\_NUCLEOSIDE\_DIPHOSPHATE\_BIOSYNTHETIC\_PROCESS |  | 9 | 0.62 | 1.71 | 0.027 | 0.250 | 1.000 | 1965 | tags=67%, list=16%, signal=80% |
| 130 | GOBP\_NUCLEOSIDE\_TRIPHOSPHATE\_BIOSYNTHETIC\_PROCESS |  | 42 | 0.40 | 1.71 | 0.002 | 0.249 | 1.000 | 1267 | tags=26%, list=11%, signal=29% |
| 131 | GOBP\_NEGATIVE\_REGULATION\_OF\_INFLAMMATORY\_RESPONSE\_TO\_ANTIGENIC\_STIMULUS |  | 4 | 0.85 | 1.70 | 0.012 | 0.253 | 1.000 | 1445 | tags=75%, list=12%, signal=85% |
| 132 | GOBP\_INNER\_MITOCHONDRIAL\_MEMBRANE\_ORGANIZATION |  | 31 | 0.44 | 1.70 | 0.009 | 0.261 | 1.000 | 2341 | tags=55%, list=19%, signal=68% |
| 133 | GOCC\_ORGANELLE\_ENVELOPE\_LUMEN |  | 67 | 0.36 | 1.70 | 0.000 | 0.260 | 1.000 | 1289 | tags=27%, list=11%, signal=30% |
| 134 | GOMF\_QUINONE\_BINDING |  | 12 | 0.57 | 1.70 | 0.022 | 0.263 | 1.000 | 1629 | tags=58%, list=14%, signal=67% |
| 135 | GOBP\_REGULATION\_OF\_MEMBRANE\_REPOLARIZATION\_DURING\_ACTION\_POTENTIAL |  | 6 | 0.72 | 1.69 | 0.028 | 0.262 | 1.000 | 3217 | tags=83%, list=27%, signal=114% |
| 136 | GOBP\_GENERATION\_OF\_PRECURSOR\_METABOLITES\_AND\_ENERGY |  | 350 | 0.28 | 1.69 | 0.000 | 0.271 | 1.000 | 1513 | tags=27%, list=13%, signal=30% |
| 137 | GOBP\_RESPONSE\_TO\_TYPE\_I\_INTERFERON |  | 49 | 0.38 | 1.69 | 0.004 | 0.270 | 1.000 | 1017 | tags=24%, list=8%, signal=27% |
| 138 | GOBP\_GLYOXYLATE\_METABOLIC\_PROCESS |  | 7 | 0.69 | 1.69 | 0.028 | 0.274 | 1.000 | 2002 | tags=43%, list=17%, signal=51% |
| 139 | GOBP\_DNA\_DEAMINATION |  | 5 | 0.76 | 1.68 | 0.026 | 0.277 | 1.000 | 2421 | tags=80%, list=20%, signal=100% |
| 140 | GOMF\_CYTOCHROME\_B5\_REDUCTASE\_ACTIVITY\_ACTING\_ON\_NAD\_P\_H |  | 4 | 0.83 | 1.68 | 0.021 | 0.276 | 1.000 | 9 | tags=25%, list=0%, signal=25% |
| 141 | GOBP\_L\_HISTIDINE\_TRANSMEMBRANE\_TRANSPORT |  | 5 | 0.76 | 1.68 | 0.031 | 0.288 | 1.000 | 1443 | tags=40%, list=12%, signal=45% |
| 142 | GOBP\_ARTERY\_SMOOTH\_MUSCLE\_CONTRACTION |  | 8 | 0.63 | 1.67 | 0.027 | 0.288 | 1.000 | 7 | tags=13%, list=0%, signal=12% |
| 143 | GOCC\_PHAGOCYTIC\_VESICLE\_MEMBRANE |  | 54 | 0.37 | 1.67 | 0.000 | 0.291 | 1.000 | 927 | tags=22%, list=8%, signal=24% |
| 144 | GOBP\_HOMOSERINE\_METABOLIC\_PROCESS |  | 4 | 0.84 | 1.67 | 0.018 | 0.290 | 1.000 | 1955 | tags=100%, list=16%, signal=119% |
| 145 | GOBP\_EPITHELIAL\_CELL\_DIFFERENTIATION\_INVOLVED\_IN\_PROSTATE\_GLAND\_DEVELOPMENT |  | 8 | 0.64 | 1.67 | 0.029 | 0.288 | 1.000 | 162 | tags=25%, list=1%, signal=25% |
| 146 | GOCC\_R2TP\_COMPLEX |  | 3 | 0.92 | 1.67 | 0.008 | 0.296 | 1.000 | 566 | tags=33%, list=5%, signal=35% |
| 147 | GOBP\_VESICLE\_FUSION\_WITH\_ENDOPLASMIC\_RETICULUM\_GOLGI\_INTERMEDIATE\_COMPARTMENT\_ERGIC\_MEMBRANE |  | 4 | 0.83 | 1.66 | 0.018 | 0.305 | 1.000 | 806 | tags=50%, list=7%, signal=54% |
| 148 | GOMF\_THREONINE\_TYPE\_ENDOPEPTIDASE\_ACTIVITY |  | 7 | 0.67 | 1.66 | 0.033 | 0.305 | 1.000 | 3006 | tags=86%, list=25%, signal=114% |
| 149 | GOCC\_BORC\_COMPLEX |  | 8 | 0.64 | 1.66 | 0.031 | 0.305 | 1.000 | 1612 | tags=63%, list=13%, signal=72% |
| 150 | GOBP\_MALATE\_METABOLIC\_PROCESS |  | 6 | 0.70 | 1.66 | 0.030 | 0.305 | 1.000 | 1026 | tags=50%, list=9%, signal=55% |
| 151 | GOMF\_COPPER\_CHAPERONE\_ACTIVITY |  | 5 | 0.76 | 1.66 | 0.033 | 0.303 | 1.000 | 2258 | tags=60%, list=19%, signal=74% |
| 152 | GOBP\_MEDIUM\_CHAIN\_FATTY\_ACID\_METABOLIC\_PROCESS |  | 9 | 0.61 | 1.66 | 0.023 | 0.303 | 1.000 | 1796 | tags=56%, list=15%, signal=65% |
| 153 | GOMF\_OXIDOREDUCTASE\_ACTIVITY\_ACTING\_ON\_NAD\_P\_H\_HEME\_PROTEIN\_AS\_ACCEPTOR |  | 7 | 0.66 | 1.66 | 0.041 | 0.302 | 1.000 | 9 | tags=14%, list=0%, signal=14% |
| 154 | GOBP\_CELLULAR\_RESPONSE\_TO\_UV\_B |  | 7 | 0.66 | 1.65 | 0.035 | 0.306 | 1.000 | 959 | tags=43%, list=8%, signal=47% |
| 155 | GOBP\_REGULATION\_OF\_OSTEOBLAST\_PROLIFERATION |  | 17 | 0.49 | 1.65 | 0.024 | 0.306 | 1.000 | 431 | tags=29%, list=4%, signal=30% |
| 156 | GOBP\_3\_PHOSPHOADENOSINE\_5\_PHOSPHOSULFATE\_BIOSYNTHETIC\_PROCESS |  | 5 | 0.77 | 1.65 | 0.032 | 0.307 | 1.000 | 452 | tags=40%, list=4%, signal=42% |
| 157 | GOCC\_PROTEASOME\_CORE\_COMPLEX\_BETA\_SUBUNIT\_COMPLEX |  | 5 | 0.75 | 1.65 | 0.032 | 0.305 | 1.000 | 2964 | tags=100%, list=25%, signal=133% |
| 158 | GOBP\_GLYCEROL\_ETHER\_METABOLIC\_PROCESS |  | 16 | 0.50 | 1.65 | 0.024 | 0.313 | 1.000 | 1468 | tags=38%, list=12%, signal=43% |
| 159 | GOBP\_DOLICHYL\_DIPHOSPHATE\_BIOSYNTHETIC\_PROCESS |  | 6 | 0.70 | 1.65 | 0.039 | 0.313 | 1.000 | 2301 | tags=67%, list=19%, signal=82% |
| 160 | GOBP\_REGULATION\_OF\_MITOCHONDRIAL\_OUTER\_MEMBRANE\_PERMEABILIZATION\_INVOLVED\_IN\_APOPTOTIC\_SIGNALING\_PATHWAY |  | 28 | 0.43 | 1.64 | 0.013 | 0.314 | 1.000 | 775 | tags=21%, list=6%, signal=23% |
| 161 | GOBP\_REGULATION\_OF\_ACUTE\_INFLAMMATORY\_RESPONSE\_TO\_ANTIGENIC\_STIMULUS |  | 7 | 0.66 | 1.64 | 0.043 | 0.312 | 1.000 | 1445 | tags=29%, list=12%, signal=32% |
| 162 | GOBP\_ELASTIN\_METABOLIC\_PROCESS |  | 3 | 0.90 | 1.63 | 0.014 | 0.329 | 1.000 | 721 | tags=67%, list=6%, signal=71% |
| 163 | GOCC\_INTRINSIC\_COMPONENT\_OF\_MITOCHONDRIAL\_MEMBRANE |  | 66 | 0.35 | 1.63 | 0.005 | 0.332 | 1.000 | 2257 | tags=39%, list=19%, signal=48% |
| 164 | GOMF\_INTRAMOLECULAR\_OXIDOREDUCTASE\_ACTIVITY |  | 38 | 0.39 | 1.63 | 0.009 | 0.335 | 1.000 | 1658 | tags=34%, list=14%, signal=40% |
| 165 | GOBP\_GLUTATHIONE\_METABOLIC\_PROCESS |  | 40 | 0.39 | 1.63 | 0.011 | 0.335 | 1.000 | 1872 | tags=35%, list=16%, signal=41% |
| 166 | GOCC\_NUCLEAR\_EXOSOME\_RNASE\_COMPLEX |  | 14 | 0.52 | 1.63 | 0.031 | 0.339 | 1.000 | 2530 | tags=57%, list=21%, signal=72% |
| 167 | GOCC\_MYOFILAMENT |  | 13 | 0.53 | 1.63 | 0.028 | 0.339 | 1.000 | 2565 | tags=46%, list=21%, signal=59% |
| 168 | GOCC\_MITOCHONDRIAL\_INTERMEMBRANE\_SPACE\_PROTEIN\_TRANSPORTER\_COMPLEX |  | 4 | 0.81 | 1.63 | 0.042 | 0.338 | 1.000 | 2341 | tags=100%, list=19%, signal=124% |
| 169 | GOBP\_MUSCLE\_FILAMENT\_SLIDING |  | 14 | 0.52 | 1.62 | 0.027 | 0.337 | 1.000 | 1505 | tags=29%, list=13%, signal=33% |
| 170 | GOBP\_RIBONUCLEOSIDE\_TRIPHOSPHATE\_BIOSYNTHETIC\_PROCESS |  | 34 | 0.40 | 1.62 | 0.007 | 0.337 | 1.000 | 1267 | tags=26%, list=11%, signal=30% |
| 171 | GOBP\_HYDROGEN\_PEROXIDE\_CATABOLIC\_PROCESS |  | 13 | 0.53 | 1.62 | 0.035 | 0.336 | 1.000 | 228 | tags=23%, list=2%, signal=23% |
| 172 | GOCC\_PROTEASOME\_REGULATORY\_PARTICLE\_LID\_SUBCOMPLEX |  | 6 | 0.70 | 1.62 | 0.047 | 0.345 | 1.000 | 2428 | tags=50%, list=20%, signal=63% |
| 173 | GOBP\_ASPARTATE\_METABOLIC\_PROCESS |  | 5 | 0.74 | 1.61 | 0.048 | 0.356 | 1.000 | 2086 | tags=80%, list=17%, signal=97% |
| 174 | GOBP\_DEOXYRIBOSE\_PHOSPHATE\_BIOSYNTHETIC\_PROCESS |  | 10 | 0.56 | 1.60 | 0.038 | 0.373 | 1.000 | 383 | tags=30%, list=3%, signal=31% |
| 175 | GOBP\_RESPONSE\_TO\_ALDOSTERONE |  | 5 | 0.73 | 1.60 | 0.051 | 0.377 | 1.000 | 1148 | tags=60%, list=10%, signal=66% |
| 176 | GOBP\_REGULATION\_OF\_MYD88\_INDEPENDENT\_TOLL\_LIKE\_RECEPTOR\_SIGNALING\_PATHWAY |  | 6 | 0.67 | 1.60 | 0.049 | 0.386 | 1.000 | 128 | tags=33%, list=1%, signal=34% |
| 177 | GOBP\_CELL\_ADHESION\_MOLECULE\_PRODUCTION |  | 11 | 0.55 | 1.60 | 0.041 | 0.386 | 1.000 | 1942 | tags=45%, list=16%, signal=54% |
| 178 | GOBP\_REGULATION\_OF\_ELECTRON\_TRANSFER\_ACTIVITY |  | 3 | 0.89 | 1.60 | 0.024 | 0.385 | 1.000 | 50 | tags=33%, list=0%, signal=33% |
| 179 | GOMF\_OXIDOREDUCTASE\_ACTIVITY\_ACTING\_ON\_METAL\_IONS\_NAD\_OR\_NADP\_AS\_ACCEPTOR |  | 7 | 0.65 | 1.60 | 0.050 | 0.384 | 1.000 | 910 | tags=57%, list=8%, signal=62% |
| 180 | GOBP\_ATP\_BIOSYNTHETIC\_PROCESS |  | 22 | 0.44 | 1.59 | 0.023 | 0.383 | 1.000 | 1267 | tags=32%, list=11%, signal=36% |
| 181 | GOBP\_PANTOTHENATE\_METABOLIC\_PROCESS |  | 5 | 0.73 | 1.59 | 0.049 | 0.383 | 1.000 | 174 | tags=40%, list=1%, signal=41% |
| 182 | GOBP\_CELLULAR\_OXIDANT\_DETOXIFICATION |  | 61 | 0.35 | 1.59 | 0.011 | 0.385 | 1.000 | 1821 | tags=30%, list=15%, signal=35% |
| 183 | GOBP\_U4\_SNRNA\_3\_END\_PROCESSING |  | 7 | 0.64 | 1.59 | 0.052 | 0.384 | 1.000 | 2421 | tags=71%, list=20%, signal=89% |
| 184 | GOBP\_SERINE\_FAMILY\_AMINO\_ACID\_METABOLIC\_PROCESS |  | 29 | 0.41 | 1.59 | 0.023 | 0.383 | 1.000 | 1415 | tags=38%, list=12%, signal=43% |
| 185 | GOBP\_METANEPHRIC\_RENAL\_VESICLE\_MORPHOGENESIS |  | 7 | 0.63 | 1.59 | 0.047 | 0.387 | 1.000 | 1723 | tags=29%, list=14%, signal=33% |
| 186 | GOCC\_MHC\_CLASS\_I\_PEPTIDE\_LOADING\_COMPLEX |  | 6 | 0.66 | 1.58 | 0.051 | 0.396 | 1.000 | 806 | tags=33%, list=7%, signal=36% |
| 187 | GOBP\_REGULATION\_OF\_ODONTOGENESIS |  | 8 | 0.61 | 1.58 | 0.053 | 0.402 | 1.000 | 1723 | tags=50%, list=14%, signal=58% |
| 188 | GOMF\_PHOSPHATIDIC\_ACID\_TRANSFER\_ACTIVITY |  | 5 | 0.72 | 1.58 | 0.054 | 0.412 | 1.000 | 1289 | tags=40%, list=11%, signal=45% |
| 189 | GOBP\_GLUTAMINE\_FAMILY\_AMINO\_ACID\_BIOSYNTHETIC\_PROCESS |  | 8 | 0.60 | 1.57 | 0.057 | 0.425 | 1.000 | 1665 | tags=38%, list=14%, signal=44% |
| 190 | GOMF\_PROTEIN\_TRANSPORTER\_ACTIVITY |  | 19 | 0.46 | 1.57 | 0.036 | 0.426 | 1.000 | 2341 | tags=53%, list=19%, signal=65% |
| 191 | GOBP\_CALCIUM\_INDEPENDENT\_CELL\_CELL\_ADHESION\_VIA\_PLASMA\_MEMBRANE\_CELL\_ADHESION\_MOLECULES |  | 11 | 0.53 | 1.57 | 0.045 | 0.424 | 1.000 | 1030 | tags=36%, list=9%, signal=40% |
| 192 | GOBP\_CORNIFICATION |  | 26 | 0.41 | 1.57 | 0.014 | 0.424 | 1.000 | 1137 | tags=31%, list=9%, signal=34% |
| 193 | GOBP\_LYSOSOMAL\_LUMEN\_ACIDIFICATION |  | 8 | 0.60 | 1.57 | 0.053 | 0.422 | 1.000 | 664 | tags=38%, list=6%, signal=40% |
| 194 | GOMF\_OXO\_ACID\_LYASE\_ACTIVITY |  | 5 | 0.71 | 1.56 | 0.056 | 0.430 | 1.000 | 44 | tags=20%, list=0%, signal=20% |
| 195 | GOMF\_PEPTIDE\_TRANSMEMBRANE\_TRANSPORTER\_ACTIVITY |  | 24 | 0.42 | 1.56 | 0.021 | 0.441 | 1.000 | 2030 | tags=42%, list=17%, signal=50% |
| 196 | GOBP\_PANCREATIC\_A\_CELL\_DIFFERENTIATION |  | 7 | 0.62 | 1.55 | 0.053 | 0.449 | 1.000 | 1084 | tags=71%, list=9%, signal=78% |
| 197 | GOBP\_REGULATION\_OF\_LIPID\_TRANSPORTER\_ACTIVITY |  | 3 | 0.86 | 1.55 | 0.036 | 0.448 | 1.000 | 83 | tags=33%, list=1%, signal=34% |
| 198 | GOBP\_PEPTIDYL\_DIPHTHAMIDE\_BIOSYNTHETIC\_PROCESS\_FROM\_PEPTIDYL\_HISTIDINE |  | 7 | 0.63 | 1.55 | 0.063 | 0.453 | 1.000 | 3725 | tags=86%, list=31%, signal=124% |
| 199 | GOBP\_GTP\_BIOSYNTHETIC\_PROCESS |  | 6 | 0.66 | 1.55 | 0.055 | 0.451 | 1.000 | 2779 | tags=67%, list=23%, signal=87% |
| 200 | GOBP\_KERATINIZATION |  | 28 | 0.40 | 1.55 | 0.020 | 0.450 | 1.000 | 1137 | tags=29%, list=9%, signal=31% |
| 201 | GOMF\_PHOSPHOLIPASE\_A2\_ACTIVITY |  | 13 | 0.51 | 1.55 | 0.048 | 0.450 | 1.000 | 299 | tags=31%, list=2%, signal=32% |
| 202 | GOBP\_MITOCHONDRION\_ORGANIZATION |  | 394 | 0.27 | 1.55 | 0.000 | 0.453 | 1.000 | 1497 | tags=23%, list=12%, signal=25% |
| 203 | GOBP\_DEOXYRIBONUCLEOSIDE\_DIPHOSPHATE\_METABOLIC\_PROCESS |  | 3 | 0.86 | 1.55 | 0.042 | 0.453 | 1.000 | 383 | tags=67%, list=3%, signal=69% |
| 204 | GOBP\_ASPARTATE\_FAMILY\_AMINO\_ACID\_CATABOLIC\_PROCESS |  | 22 | 0.42 | 1.55 | 0.025 | 0.450 | 1.000 | 2550 | tags=55%, list=21%, signal=69% |
| 205 | GOBP\_ADENYLATE\_CYCLASE\_INHIBITING\_SEROTONIN\_RECEPTOR\_SIGNALING\_PATHWAY |  | 3 | 0.85 | 1.55 | 0.040 | 0.449 | 1.000 | 1807 | tags=100%, list=15%, signal=118% |
| 206 | GOBP\_CYSTEINE\_METABOLIC\_PROCESS |  | 8 | 0.59 | 1.54 | 0.065 | 0.449 | 1.000 | 1955 | tags=63%, list=16%, signal=75% |
| 207 | GOBP\_POSITIVE\_REGULATION\_OF\_ODONTOGENESIS |  | 5 | 0.71 | 1.54 | 0.067 | 0.448 | 1.000 | 1188 | tags=40%, list=10%, signal=44% |
| 208 | GOMF\_TAP\_BINDING |  | 3 | 0.86 | 1.54 | 0.044 | 0.446 | 1.000 | 806 | tags=67%, list=7%, signal=71% |
| 209 | GOMF\_TAP1\_BINDING |  | 3 | 0.86 | 1.54 | 0.041 | 0.444 | 1.000 | 806 | tags=67%, list=7%, signal=71% |
| 210 | GOBP\_FATTY\_ACID\_BETA\_OXIDATION\_USING\_ACYL\_COA\_DEHYDROGENASE |  | 8 | 0.59 | 1.54 | 0.058 | 0.444 | 1.000 | 2069 | tags=50%, list=17%, signal=60% |
| 211 | GOMF\_MACROMOLECULE\_TRANSMEMBRANE\_TRANSPORTER\_ACTIVITY |  | 16 | 0.47 | 1.54 | 0.035 | 0.452 | 1.000 | 2030 | tags=44%, list=17%, signal=53% |
| 212 | GOBP\_RESPONSE\_TO\_MINERALOCORTICOID |  | 18 | 0.45 | 1.54 | 0.037 | 0.454 | 1.000 | 1148 | tags=33%, list=10%, signal=37% |
| 213 | GOCC\_ENDOPLASMIC\_RETICULUM\_TUBULAR\_NETWORK\_MEMBRANE |  | 4 | 0.78 | 1.54 | 0.065 | 0.452 | 1.000 | 1070 | tags=50%, list=9%, signal=55% |
| 214 | GOMF\_TRNA\_SPECIFIC\_RIBONUCLEASE\_ACTIVITY |  | 8 | 0.59 | 1.54 | 0.058 | 0.451 | 1.000 | 1517 | tags=50%, list=13%, signal=57% |
| 215 | GOBP\_TONGUE\_MORPHOGENESIS |  | 3 | 0.85 | 1.53 | 0.045 | 0.453 | 1.000 | 908 | tags=67%, list=8%, signal=72% |
| 216 | GOBP\_HYDROGEN\_SULFIDE\_METABOLIC\_PROCESS |  | 4 | 0.75 | 1.53 | 0.062 | 0.452 | 1.000 | 1955 | tags=75%, list=16%, signal=90% |
| 217 | GOBP\_POSITIVE\_REGULATION\_OF\_STRIATED\_MUSCLE\_CONTRACTION |  | 5 | 0.70 | 1.53 | 0.069 | 0.454 | 1.000 | 509 | tags=60%, list=4%, signal=63% |
| 218 | GOBP\_OLIGOSACCHARIDE\_BIOSYNTHETIC\_PROCESS |  | 11 | 0.54 | 1.53 | 0.060 | 0.454 | 1.000 | 1506 | tags=36%, list=13%, signal=42% |
| 219 | GOBP\_CELLULAR\_RESPONSE\_TO\_PH |  | 16 | 0.46 | 1.53 | 0.051 | 0.455 | 1.000 | 1292 | tags=38%, list=11%, signal=42% |
| 220 | GOBP\_TRNA\_5\_LEADER\_REMOVAL |  | 8 | 0.59 | 1.53 | 0.065 | 0.460 | 1.000 | 1517 | tags=50%, list=13%, signal=57% |
| 221 | GOBP\_ISG15\_PROTEIN\_CONJUGATION |  | 3 | 0.84 | 1.53 | 0.046 | 0.460 | 1.000 | 141 | tags=33%, list=1%, signal=34% |
| 222 | GOBP\_NEGATIVE\_REGULATION\_OF\_OXIDOREDUCTASE\_ACTIVITY |  | 13 | 0.49 | 1.53 | 0.046 | 0.463 | 1.000 | 670 | tags=23%, list=6%, signal=24% |
| 223 | GOBP\_REGULATION\_OF\_MDA\_5\_SIGNALING\_PATHWAY |  | 6 | 0.64 | 1.53 | 0.071 | 0.461 | 1.000 | 1306 | tags=50%, list=11%, signal=56% |
| 224 | GOBP\_CYTOPLASMIC\_PATTERN\_RECOGNITION\_RECEPTOR\_SIGNALING\_PATHWAY\_IN\_RESPONSE\_TO\_VIRUS |  | 20 | 0.44 | 1.52 | 0.036 | 0.460 | 1.000 | 332 | tags=20%, list=3%, signal=21% |
| 225 | GOBP\_GLYCOLYTIC\_PROCESS\_THROUGH\_FRUCTOSE\_6\_PHOSPHATE |  | 22 | 0.42 | 1.52 | 0.029 | 0.459 | 1.000 | 1251 | tags=32%, list=10%, signal=35% |
| 226 | GOMF\_L\_HISTIDINE\_TRANSMEMBRANE\_TRANSPORTER\_ACTIVITY |  | 4 | 0.76 | 1.52 | 0.074 | 0.462 | 1.000 | 682 | tags=25%, list=6%, signal=26% |
| 227 | GOBP\_NEGATIVE\_REGULATION\_OF\_OXIDATIVE\_STRESS\_INDUCED\_INTRINSIC\_APOPTOTIC\_SIGNALING\_PATHWAY |  | 15 | 0.47 | 1.52 | 0.047 | 0.461 | 1.000 | 2060 | tags=47%, list=17%, signal=56% |
| 228 | GOBP\_GUANOSINE\_CONTAINING\_COMPOUND\_BIOSYNTHETIC\_PROCESS |  | 8 | 0.59 | 1.52 | 0.063 | 0.463 | 1.000 | 1836 | tags=50%, list=15%, signal=59% |
| 229 | GOBP\_NEGATIVE\_REGULATION\_OF\_AMINO\_ACID\_TRANSPORT |  | 6 | 0.65 | 1.52 | 0.080 | 0.462 | 1.000 | 804 | tags=50%, list=7%, signal=54% |
| 230 | GOBP\_UTP\_BIOSYNTHETIC\_PROCESS |  | 6 | 0.64 | 1.52 | 0.068 | 0.461 | 1.000 | 1836 | tags=50%, list=15%, signal=59% |
| 231 | GOBP\_RESPONSE\_TO\_MERCURY\_ION |  | 6 | 0.65 | 1.52 | 0.065 | 0.459 | 1.000 | 1548 | tags=50%, list=13%, signal=57% |
| 232 | GOBP\_DEOXYRIBONUCLEOTIDE\_BIOSYNTHETIC\_PROCESS |  | 13 | 0.49 | 1.52 | 0.045 | 0.461 | 1.000 | 383 | tags=23%, list=3%, signal=24% |
| 233 | GOMF\_ALPHA\_ADRENERGIC\_RECEPTOR\_ACTIVITY |  | 4 | 0.76 | 1.52 | 0.067 | 0.461 | 1.000 | 2740 | tags=75%, list=23%, signal=97% |
| 234 | GOBP\_CELLULAR\_RESPONSE\_TO\_DSRNA |  | 12 | 0.51 | 1.52 | 0.058 | 0.461 | 1.000 | 969 | tags=25%, list=8%, signal=27% |
| 235 | GOMF\_SHORT\_CHAIN\_CARBOXYLESTERASE\_ACTIVITY |  | 3 | 0.83 | 1.52 | 0.057 | 0.459 | 1.000 | 201 | tags=67%, list=2%, signal=68% |
| 236 | GOCC\_CYTOSOLIC\_SMALL\_RIBOSOMAL\_SUBUNIT |  | 37 | 0.36 | 1.52 | 0.016 | 0.459 | 1.000 | 3085 | tags=54%, list=26%, signal=73% |
| 237 | GOBP\_ACUTE\_INFLAMMATORY\_RESPONSE\_TO\_ANTIGENIC\_STIMULUS |  | 11 | 0.52 | 1.51 | 0.064 | 0.460 | 1.000 | 1445 | tags=36%, list=12%, signal=41% |
| 238 | GOBP\_VITAMIN\_CATABOLIC\_PROCESS |  | 6 | 0.65 | 1.51 | 0.066 | 0.458 | 1.000 | 1868 | tags=50%, list=16%, signal=59% |
| 239 | GOBP\_RESPONSE\_TO\_EPINEPHRINE |  | 13 | 0.49 | 1.51 | 0.047 | 0.466 | 1.000 | 509 | tags=23%, list=4%, signal=24% |
| 240 | GOMF\_CATALYTIC\_ACTIVITY\_ACTING\_ON\_A\_RRNA |  | 16 | 0.46 | 1.51 | 0.057 | 0.464 | 1.000 | 1916 | tags=50%, list=16%, signal=59% |
| 241 | GOBP\_SUCCINATE\_METABOLIC\_PROCESS |  | 5 | 0.69 | 1.51 | 0.071 | 0.463 | 1.000 | 2297 | tags=60%, list=19%, signal=74% |
| 242 | GOBP\_REGULATION\_OF\_MEMBRANE\_REPOLARIZATION\_DURING\_VENTRICULAR\_CARDIAC\_MUSCLE\_CELL\_ACTION\_POTENTIAL |  | 3 | 0.82 | 1.51 | 0.052 | 0.462 | 1.000 | 2179 | tags=100%, list=18%, signal=122% |
| 243 | GOCC\_DOPAMINERGIC\_SYNAPSE |  | 5 | 0.69 | 1.51 | 0.077 | 0.461 | 1.000 | 969 | tags=60%, list=8%, signal=65% |
| 244 | GOCC\_EUKARYOTIC\_TRANSLATION\_INITIATION\_FACTOR\_4F\_COMPLEX |  | 9 | 0.55 | 1.51 | 0.066 | 0.460 | 1.000 | 5357 | tags=100%, list=45%, signal=180% |
| 245 | GOBP\_THREONINE\_CATABOLIC\_PROCESS |  | 4 | 0.74 | 1.51 | 0.076 | 0.458 | 1.000 | 2320 | tags=75%, list=19%, signal=93% |
| 246 | GOMF\_ACETYLESTERASE\_ACTIVITY |  | 3 | 0.83 | 1.51 | 0.057 | 0.459 | 1.000 | 201 | tags=67%, list=2%, signal=68% |
| 247 | GOBP\_CLEAVAGE\_FURROW\_FORMATION |  | 6 | 0.64 | 1.50 | 0.069 | 0.466 | 1.000 | 3449 | tags=67%, list=29%, signal=93% |
| 248 | GOBP\_THREONINE\_METABOLIC\_PROCESS |  | 4 | 0.74 | 1.50 | 0.084 | 0.464 | 1.000 | 2320 | tags=75%, list=19%, signal=93% |
| 249 | GOBP\_HYDROGEN\_SULFIDE\_BIOSYNTHETIC\_PROCESS |  | 4 | 0.75 | 1.50 | 0.074 | 0.463 | 1.000 | 1955 | tags=75%, list=16%, signal=90% |
| 250 | GOBP\_IRON\_SULFUR\_CLUSTER\_ASSEMBLY |  | 18 | 0.44 | 1.50 | 0.047 | 0.462 | 1.000 | 3150 | tags=61%, list=26%, signal=83% |
| 251 | GOBP\_GLUTAMATE\_BIOSYNTHETIC\_PROCESS |  | 3 | 0.82 | 1.50 | 0.065 | 0.464 | 1.000 | 687 | tags=33%, list=6%, signal=35% |
| 252 | GOBP\_MEDIUM\_CHAIN\_FATTY\_ACID\_CATABOLIC\_PROCESS |  | 3 | 0.83 | 1.50 | 0.067 | 0.462 | 1.000 | 201 | tags=67%, list=2%, signal=68% |
| 253 | GOBP\_OSTEOBLAST\_PROLIFERATION |  | 20 | 0.42 | 1.50 | 0.042 | 0.466 | 1.000 | 431 | tags=25%, list=4%, signal=26% |
| 254 | GOBP\_NEGATIVE\_REGULATION\_OF\_WNT\_SIGNALING\_PATHWAY\_INVOLVED\_IN\_HEART\_DEVELOPMENT |  | 3 | 0.83 | 1.50 | 0.060 | 0.471 | 1.000 | 1104 | tags=67%, list=9%, signal=73% |
| 255 | GOBP\_NOREPINEPHRINE\_BIOSYNTHETIC\_PROCESS |  | 4 | 0.75 | 1.50 | 0.076 | 0.471 | 1.000 | 584 | tags=50%, list=5%, signal=53% |
| 256 | GOBP\_CELLULAR\_SODIUM\_ION\_HOMEOSTASIS |  | 10 | 0.53 | 1.50 | 0.068 | 0.471 | 1.000 | 79 | tags=20%, list=1%, signal=20% |
| 257 | GOMF\_ADP\_RIBOSE\_DIPHOSPHATASE\_ACTIVITY |  | 4 | 0.75 | 1.49 | 0.083 | 0.475 | 1.000 | 1776 | tags=50%, list=15%, signal=59% |
| 258 | GOBP\_PURINE\_DEOXYRIBONUCLEOTIDE\_BIOSYNTHETIC\_PROCESS |  | 4 | 0.74 | 1.49 | 0.081 | 0.478 | 1.000 | 383 | tags=50%, list=3%, signal=52% |
| 259 | GOBP\_POSITIVE\_REGULATION\_OF\_PLATELET\_DERIVED\_GROWTH\_FACTOR\_RECEPTOR\_SIGNALING\_PATHWAY |  | 5 | 0.68 | 1.49 | 0.093 | 0.478 | 1.000 | 1858 | tags=60%, list=15%, signal=71% |
| 260 | GOBP\_TERPENOID\_CATABOLIC\_PROCESS |  | 3 | 0.83 | 1.49 | 0.069 | 0.479 | 1.000 | 1868 | tags=67%, list=16%, signal=79% |
| 261 | GOBP\_REGULATION\_OF\_RIBONUCLEASE\_ACTIVITY |  | 5 | 0.68 | 1.49 | 0.081 | 0.481 | 1.000 | 138 | tags=20%, list=1%, signal=20% |
| 262 | GOBP\_ATP\_SYNTHESIS\_COUPLED\_PROTON\_TRANSPORT |  | 4 | 0.75 | 1.49 | 0.084 | 0.480 | 1.000 | 1267 | tags=50%, list=11%, signal=56% |
| 263 | GOBP\_POSITIVE\_REGULATION\_OF\_CARDIAC\_MUSCLE\_CONTRACTION |  | 4 | 0.73 | 1.49 | 0.088 | 0.479 | 1.000 | 509 | tags=75%, list=4%, signal=78% |
| 264 | GOMF\_BH3\_DOMAIN\_BINDING |  | 4 | 0.73 | 1.49 | 0.092 | 0.480 | 1.000 | 3023 | tags=75%, list=25%, signal=100% |
| 265 | GOMF\_INTRACILIARY\_TRANSPORT\_PARTICLE\_B\_BINDING |  | 4 | 0.73 | 1.48 | 0.086 | 0.480 | 1.000 | 301 | tags=25%, list=3%, signal=26% |
| 266 | GOMF\_UBIQUINONE\_BINDING |  | 5 | 0.67 | 1.48 | 0.091 | 0.480 | 1.000 | 1629 | tags=60%, list=14%, signal=69% |
| 267 | GOBP\_CELLULAR\_RESPONSE\_TO\_MINERALOCORTICOID\_STIMULUS |  | 5 | 0.68 | 1.48 | 0.103 | 0.481 | 1.000 | 1148 | tags=60%, list=10%, signal=66% |
| 268 | GOBP\_VIRAL\_RELEASE\_FROM\_HOST\_CELL |  | 22 | 0.41 | 1.48 | 0.046 | 0.480 | 1.000 | 2585 | tags=50%, list=22%, signal=64% |
| 269 | GOBP\_NEGATIVE\_REGULATION\_OF\_GLYCOPROTEIN\_METABOLIC\_PROCESS |  | 13 | 0.48 | 1.48 | 0.055 | 0.479 | 1.000 | 721 | tags=31%, list=6%, signal=33% |
| 270 | GOMF\_ARYL\_SULFOTRANSFERASE\_ACTIVITY |  | 3 | 0.83 | 1.48 | 0.066 | 0.483 | 1.000 | 54 | tags=33%, list=0%, signal=33% |
| 271 | GOBP\_PENTOSE\_PHOSPHATE\_SHUNT\_NON\_OXIDATIVE\_BRANCH |  | 4 | 0.74 | 1.48 | 0.093 | 0.485 | 1.000 | 1947 | tags=75%, list=16%, signal=89% |
| 272 | GOBP\_REGULATION\_OF\_ENDOSOME\_TO\_PLASMA\_MEMBRANE\_PROTEIN\_TRANSPORT |  | 3 | 0.81 | 1.48 | 0.073 | 0.486 | 1.000 | 2117 | tags=67%, list=18%, signal=81% |
| 273 | GOBP\_GLUTAMATE\_CATABOLIC\_PROCESS |  | 5 | 0.66 | 1.47 | 0.092 | 0.491 | 1.000 | 673 | tags=40%, list=6%, signal=42% |
| 274 | GOBP\_CARDIOLIPIN\_METABOLIC\_PROCESS |  | 14 | 0.47 | 1.47 | 0.074 | 0.491 | 1.000 | 2411 | tags=50%, list=20%, signal=62% |
| 275 | GOBP\_REGULATION\_OF\_MEMBRANE\_REPOLARIZATION\_DURING\_CARDIAC\_MUSCLE\_CELL\_ACTION\_POTENTIAL |  | 4 | 0.73 | 1.47 | 0.097 | 0.489 | 1.000 | 3217 | tags=100%, list=27%, signal=137% |
| 276 | GOBP\_GROOMING\_BEHAVIOR |  | 7 | 0.60 | 1.47 | 0.082 | 0.488 | 1.000 | 319 | tags=43%, list=3%, signal=44% |
| 277 | GOMF\_PROTON\_TRANSPORTING\_ATP\_SYNTHASE\_ACTIVITY\_ROTATIONAL\_MECHANISM |  | 3 | 0.81 | 1.47 | 0.077 | 0.488 | 1.000 | 1267 | tags=67%, list=11%, signal=75% |
| 278 | GOMF\_CADHERIN\_BINDING\_INVOLVED\_IN\_CELL\_CELL\_ADHESION |  | 15 | 0.46 | 1.47 | 0.050 | 0.491 | 1.000 | 1500 | tags=33%, list=12%, signal=38% |
| 279 | GOMF\_STRUCTURAL\_CONSTITUENT\_OF\_MYELIN\_SHEATH |  | 7 | 0.59 | 1.47 | 0.083 | 0.492 | 1.000 | 345 | tags=29%, list=3%, signal=29% |
| 280 | GOBP\_NOREPINEPHRINE\_METABOLIC\_PROCESS |  | 6 | 0.63 | 1.47 | 0.089 | 0.491 | 1.000 | 584 | tags=50%, list=5%, signal=53% |
| 281 | GOCC\_TRANSLOCON\_COMPLEX |  | 5 | 0.67 | 1.47 | 0.086 | 0.489 | 1.000 | 960 | tags=40%, list=8%, signal=43% |
| 282 | GOBP\_MEMBRANE\_REPOLARIZATION\_DURING\_VENTRICULAR\_CARDIAC\_MUSCLE\_CELL\_ACTION\_POTENTIAL |  | 8 | 0.56 | 1.47 | 0.082 | 0.490 | 1.000 | 2179 | tags=63%, list=18%, signal=76% |
| 283 | GOBP\_RELAXATION\_OF\_SMOOTH\_MUSCLE |  | 6 | 0.62 | 1.47 | 0.082 | 0.490 | 1.000 | 2778 | tags=50%, list=23%, signal=65% |
| 284 | GOBP\_REGULATION\_OF\_SYSTEMIC\_ARTERIAL\_BLOOD\_PRESSURE\_BY\_VASOPRESSIN |  | 4 | 0.72 | 1.46 | 0.096 | 0.505 | 1.000 | 1090 | tags=75%, list=9%, signal=82% |
| 285 | GOBP\_FLAVONOID\_METABOLIC\_PROCESS |  | 3 | 0.81 | 1.46 | 0.082 | 0.504 | 1.000 | 2261 | tags=100%, list=19%, signal=123% |
| 286 | GOBP\_NEGATIVE\_REGULATION\_OF\_MEMBRANE\_DEPOLARIZATION |  | 6 | 0.62 | 1.46 | 0.096 | 0.503 | 1.000 | 673 | tags=33%, list=6%, signal=35% |
| 287 | GOBP\_NEGATIVE\_REGULATION\_OF\_PEPTIDE\_SECRETION |  | 42 | 0.34 | 1.46 | 0.027 | 0.504 | 1.000 | 1585 | tags=29%, list=13%, signal=33% |
| 288 | GOBP\_POSITIVE\_REGULATION\_OF\_INTERFERON\_ALPHA\_PRODUCTION |  | 16 | 0.44 | 1.46 | 0.061 | 0.503 | 1.000 | 159 | tags=25%, list=1%, signal=25% |
| 289 | GOBP\_REGULATION\_OF\_AMYLOID\_FIBRIL\_FORMATION |  | 10 | 0.51 | 1.46 | 0.088 | 0.504 | 1.000 | 1091 | tags=40%, list=9%, signal=44% |
| 290 | GOCC\_U6\_SNRNP |  | 6 | 0.63 | 1.46 | 0.109 | 0.505 | 1.000 | 3249 | tags=83%, list=27%, signal=114% |
| 291 | GOMF\_MALATE\_DEHYDROGENASE\_ACTIVITY |  | 5 | 0.65 | 1.45 | 0.094 | 0.507 | 1.000 | 980 | tags=40%, list=8%, signal=44% |
| 292 | GOCC\_PARALLEL\_FIBER\_TO\_PURKINJE\_CELL\_SYNAPSE |  | 7 | 0.58 | 1.45 | 0.094 | 0.516 | 1.000 | 2151 | tags=43%, list=18%, signal=52% |
| 293 | GOMF\_THIOREDOXIN\_PEROXIDASE\_ACTIVITY |  | 3 | 0.80 | 1.45 | 0.091 | 0.516 | 1.000 | 228 | tags=33%, list=2%, signal=34% |
| 294 | GOMF\_SOLUTE\_ANION\_ANTIPORTER\_ACTIVITY |  | 23 | 0.39 | 1.45 | 0.055 | 0.515 | 1.000 | 712 | tags=30%, list=6%, signal=32% |
| 295 | GOCC\_CHAPERONIN\_CONTAINING\_T\_COMPLEX |  | 8 | 0.55 | 1.45 | 0.091 | 0.513 | 1.000 | 5355 | tags=100%, list=45%, signal=180% |
| 296 | GOCC\_PROTEASOME\_ACCESSORY\_COMPLEX |  | 20 | 0.41 | 1.45 | 0.060 | 0.515 | 1.000 | 2619 | tags=40%, list=22%, signal=51% |
| 297 | GOBP\_POSITIVE\_REGULATION\_OF\_ENDOTHELIAL\_CELL\_DEVELOPMENT |  | 5 | 0.66 | 1.45 | 0.100 | 0.515 | 1.000 | 101 | tags=20%, list=1%, signal=20% |
| 298 | GOBP\_NEGATIVE\_REGULATION\_OF\_LOW\_DENSITY\_LIPOPROTEIN\_PARTICLE\_CLEARANCE |  | 3 | 0.80 | 1.45 | 0.089 | 0.514 | 1.000 | 158 | tags=33%, list=1%, signal=34% |
| 299 | GOCC\_SODIUM\_CHANNEL\_COMPLEX |  | 12 | 0.48 | 1.44 | 0.085 | 0.518 | 1.000 | 472 | tags=33%, list=4%, signal=35% |
| 300 | GOBP\_CELLULAR\_RESPONSE\_TO\_EPINEPHRINE\_STIMULUS |  | 10 | 0.51 | 1.44 | 0.091 | 0.516 | 1.000 | 509 | tags=20%, list=4%, signal=21% |
| 301 | GOMF\_HEXOSE\_TRANSMEMBRANE\_TRANSPORTER\_ACTIVITY |  | 14 | 0.46 | 1.44 | 0.067 | 0.515 | 1.000 | 633 | tags=36%, list=5%, signal=38% |
| 302 | GOBP\_SERINE\_FAMILY\_AMINO\_ACID\_CATABOLIC\_PROCESS |  | 9 | 0.54 | 1.44 | 0.080 | 0.515 | 1.000 | 1224 | tags=33%, list=10%, signal=37% |
| 303 | GOBP\_REGULATION\_OF\_NADP\_METABOLIC\_PROCESS |  | 3 | 0.80 | 1.44 | 0.090 | 0.516 | 1.000 | 1251 | tags=67%, list=10%, signal=74% |
| 304 | GOCC\_LSM2\_8\_COMPLEX |  | 6 | 0.63 | 1.44 | 0.097 | 0.515 | 1.000 | 3249 | tags=83%, list=27%, signal=114% |
| 305 | GOBP\_DEOXYRIBONUCLEOSIDE\_TRIPHOSPHATE\_BIOSYNTHETIC\_PROCESS |  | 5 | 0.65 | 1.44 | 0.097 | 0.518 | 1.000 | 65 | tags=20%, list=1%, signal=20% |
| 306 | GOBP\_DETOXIFICATION |  | 75 | 0.30 | 1.44 | 0.020 | 0.520 | 1.000 | 228 | tags=12%, list=2%, signal=12% |
| 307 | GOBP\_RNA\_IMPORT\_INTO\_MITOCHONDRION |  | 4 | 0.72 | 1.44 | 0.107 | 0.523 | 1.000 | 2512 | tags=75%, list=21%, signal=95% |
| 308 | GOBP\_L\_PHENYLALANINE\_METABOLIC\_PROCESS |  | 6 | 0.60 | 1.43 | 0.095 | 0.526 | 1.000 | 586 | tags=50%, list=5%, signal=53% |
| 309 | GOBP\_POSITIVE\_REGULATION\_OF\_MITOCHONDRIAL\_OUTER\_MEMBRANE\_PERMEABILIZATION\_INVOLVED\_IN\_APOPTOTIC\_SIGNALING\_PATHWAY |  | 23 | 0.39 | 1.43 | 0.050 | 0.526 | 1.000 | 2260 | tags=35%, list=19%, signal=43% |
| 310 | GOBP\_POTASSIUM\_ION\_HOMEOSTASIS |  | 14 | 0.46 | 1.43 | 0.087 | 0.525 | 1.000 | 2376 | tags=57%, list=20%, signal=71% |
| 311 | GOBP\_SENSORY\_PERCEPTION\_OF\_TOUCH |  | 3 | 0.79 | 1.43 | 0.106 | 0.526 | 1.000 | 403 | tags=33%, list=3%, signal=34% |
| 312 | GOBP\_TRNA\_5\_END\_PROCESSING |  | 11 | 0.50 | 1.43 | 0.087 | 0.527 | 1.000 | 1517 | tags=36%, list=13%, signal=42% |
| 313 | GOBP\_ALPHA\_AMINO\_ACID\_CATABOLIC\_PROCESS |  | 62 | 0.31 | 1.43 | 0.011 | 0.525 | 1.000 | 1224 | tags=24%, list=10%, signal=27% |
| 314 | GOBP\_POSITIVE\_REGULATION\_OF\_TRIGLYCERIDE\_LIPASE\_ACTIVITY |  | 4 | 0.70 | 1.43 | 0.114 | 0.524 | 1.000 | 216 | tags=25%, list=2%, signal=25% |
| 315 | GOCC\_CYTOPLASMIC\_SIDE\_OF\_ROUGH\_ENDOPLASMIC\_RETICULUM\_MEMBRANE |  | 5 | 0.64 | 1.43 | 0.104 | 0.523 | 1.000 | 2332 | tags=80%, list=19%, signal=99% |
| 316 | GOBP\_MESENCHYMAL\_TO\_EPITHELIAL\_TRANSITION\_INVOLVED\_IN\_METANEPHROS\_MORPHOGENESIS |  | 5 | 0.65 | 1.43 | 0.112 | 0.533 | 1.000 | 1723 | tags=40%, list=14%, signal=47% |
| 317 | GOBP\_RESPONSE\_TO\_GONADOTROPIN\_RELEASING\_HORMONE |  | 3 | 0.79 | 1.43 | 0.104 | 0.532 | 1.000 | 622 | tags=33%, list=5%, signal=35% |
| 318 | GOCC\_ROUGH\_ENDOPLASMIC\_RETICULUM\_MEMBRANE |  | 23 | 0.39 | 1.43 | 0.060 | 0.531 | 1.000 | 960 | tags=22%, list=8%, signal=24% |
| 319 | GOMF\_OXIDOREDUCTASE\_ACTIVITY\_ACTING\_ON\_METAL\_IONS |  | 14 | 0.45 | 1.42 | 0.079 | 0.533 | 1.000 | 910 | tags=36%, list=8%, signal=39% |
| 320 | GOMF\_THIOREDOXIN\_DISULFIDE\_REDUCTASE\_ACTIVITY |  | 5 | 0.64 | 1.42 | 0.102 | 0.539 | 1.000 | 1959 | tags=60%, list=16%, signal=72% |
| 321 | GOBP\_AROMATIC\_AMINO\_ACID\_FAMILY\_METABOLIC\_PROCESS |  | 17 | 0.43 | 1.42 | 0.076 | 0.538 | 1.000 | 739 | tags=29%, list=6%, signal=31% |
| 322 | GOCC\_LEWY\_BODY |  | 6 | 0.60 | 1.42 | 0.110 | 0.540 | 1.000 | 989 | tags=50%, list=8%, signal=54% |
| 323 | GOMF\_S\_ADENOSYL\_L\_METHIONINE\_BINDING |  | 13 | 0.46 | 1.42 | 0.082 | 0.544 | 1.000 | 2007 | tags=54%, list=17%, signal=65% |
| 324 | GOMF\_LACTATE\_DEHYDROGENASE\_ACTIVITY |  | 3 | 0.79 | 1.42 | 0.108 | 0.542 | 1.000 | 2583 | tags=100%, list=22%, signal=127% |
| 325 | GOBP\_REGULATION\_OF\_LOW\_DENSITY\_LIPOPROTEIN\_PARTICLE\_RECEPTOR\_CATABOLIC\_PROCESS |  | 4 | 0.70 | 1.42 | 0.122 | 0.543 | 1.000 | 158 | tags=25%, list=1%, signal=25% |
| 326 | GOBP\_ISOPENTENYL\_DIPHOSPHATE\_BIOSYNTHETIC\_PROCESS |  | 4 | 0.70 | 1.42 | 0.120 | 0.543 | 1.000 | 636 | tags=50%, list=5%, signal=53% |
| 327 | GOBP\_MAMMARY\_GLAND\_INVOLUTION |  | 4 | 0.71 | 1.42 | 0.127 | 0.543 | 1.000 | 3501 | tags=100%, list=29%, signal=141% |
| 328 | GOMF\_1\_ALKYL\_2\_ACETYLGLYCEROPHOSPHOCHOLINE\_ESTERASE\_ACTIVITY |  | 6 | 0.60 | 1.41 | 0.112 | 0.542 | 1.000 | 4839 | tags=100%, list=40%, signal=167% |
| 329 | GOBP\_NEGATIVE\_REGULATION\_OF\_VIRAL\_LIFE\_CYCLE |  | 9 | 0.51 | 1.41 | 0.109 | 0.552 | 1.000 | 2441 | tags=67%, list=20%, signal=84% |
| 330 | GOBP\_REGULATION\_OF\_CARDIAC\_MUSCLE\_CELL\_MEMBRANE\_POTENTIAL |  | 3 | 0.77 | 1.41 | 0.119 | 0.551 | 1.000 | 79 | tags=33%, list=1%, signal=34% |
| 331 | GOCC\_OUTER\_MITOCHONDRIAL\_MEMBRANE\_PROTEIN\_COMPLEX |  | 16 | 0.43 | 1.41 | 0.080 | 0.558 | 1.000 | 2097 | tags=56%, list=17%, signal=68% |
| 332 | GOBP\_SODIUM\_ION\_EXPORT\_ACROSS\_PLASMA\_MEMBRANE |  | 7 | 0.56 | 1.40 | 0.112 | 0.561 | 1.000 | 79 | tags=29%, list=1%, signal=29% |
| 333 | GOBP\_TYROSINE\_METABOLIC\_PROCESS |  | 4 | 0.70 | 1.40 | 0.124 | 0.560 | 1.000 | 546 | tags=75%, list=5%, signal=79% |
| 334 | GOMF\_ACIDIC\_AMINO\_ACID\_TRANSMEMBRANE\_TRANSPORTER\_ACTIVITY |  | 8 | 0.53 | 1.40 | 0.112 | 0.560 | 1.000 | 1278 | tags=38%, list=11%, signal=42% |
| 335 | GOBP\_DETECTION\_OF\_TEMPERATURE\_STIMULUS |  | 11 | 0.48 | 1.40 | 0.105 | 0.561 | 1.000 | 1235 | tags=36%, list=10%, signal=40% |
| 336 | GOBP\_RIBOSOME\_ASSEMBLY |  | 50 | 0.32 | 1.40 | 0.031 | 0.569 | 1.000 | 1863 | tags=34%, list=16%, signal=40% |
| 337 | GOMF\_2\_IRON\_2\_SULFUR\_CLUSTER\_BINDING |  | 18 | 0.42 | 1.40 | 0.083 | 0.569 | 1.000 | 1492 | tags=39%, list=12%, signal=44% |
| 338 | GOBP\_TRICARBOXYLIC\_ACID\_CYCLE |  | 31 | 0.36 | 1.40 | 0.051 | 0.568 | 1.000 | 1242 | tags=29%, list=10%, signal=32% |
| 339 | GOBP\_REGULATION\_OF\_FEMALE\_RECEPTIVITY |  | 5 | 0.64 | 1.40 | 0.114 | 0.569 | 1.000 | 670 | tags=40%, list=6%, signal=42% |
| 340 | GOBP\_PROTEIN\_MATURATION\_BY\_IRON\_SULFUR\_CLUSTER\_TRANSFER |  | 7 | 0.56 | 1.40 | 0.124 | 0.568 | 1.000 | 2191 | tags=71%, list=18%, signal=87% |
| 341 | GOBP\_INTERFERON\_GAMMA\_MEDIATED\_SIGNALING\_PATHWAY |  | 45 | 0.32 | 1.40 | 0.039 | 0.566 | 1.000 | 864 | tags=24%, list=7%, signal=26% |
| 342 | GOBP\_NEGATIVE\_REGULATION\_OF\_VIRAL\_RELEASE\_FROM\_HOST\_CELL |  | 5 | 0.64 | 1.39 | 0.111 | 0.568 | 1.000 | 2441 | tags=60%, list=20%, signal=75% |
| 343 | GOBP\_VASCULOGENESIS\_INVOLVED\_IN\_CORONARY\_VASCULAR\_MORPHOGENESIS |  | 3 | 0.77 | 1.39 | 0.122 | 0.567 | 1.000 | 844 | tags=33%, list=7%, signal=36% |
| 344 | GOBP\_POSITIVE\_REGULATION\_OF\_OSTEOBLAST\_PROLIFERATION |  | 6 | 0.59 | 1.39 | 0.110 | 0.570 | 1.000 | 270 | tags=33%, list=2%, signal=34% |
| 345 | GOBP\_REGULATION\_OF\_CELLULAR\_AMINE\_METABOLIC\_PROCESS |  | 53 | 0.31 | 1.39 | 0.030 | 0.574 | 1.000 | 2619 | tags=38%, list=22%, signal=48% |
| 346 | GOBP\_PROSTATE\_GLANDULAR\_ACINUS\_DEVELOPMENT |  | 8 | 0.53 | 1.39 | 0.096 | 0.572 | 1.000 | 162 | tags=25%, list=1%, signal=25% |
| 347 | GOBP\_RENAL\_VESICLE\_DEVELOPMENT |  | 11 | 0.47 | 1.39 | 0.112 | 0.578 | 1.000 | 1723 | tags=27%, list=14%, signal=32% |
| 348 | GOBP\_ADP\_TRANSPORT |  | 6 | 0.58 | 1.39 | 0.113 | 0.580 | 1.000 | 2023 | tags=50%, list=17%, signal=60% |
| 349 | GOBP\_REGULATION\_OF\_VIRAL\_TRANSCRIPTION |  | 29 | 0.35 | 1.38 | 0.068 | 0.586 | 1.000 | 2420 | tags=41%, list=20%, signal=52% |
| 350 | GOMF\_ADP\_TRANSMEMBRANE\_TRANSPORTER\_ACTIVITY |  | 6 | 0.58 | 1.38 | 0.119 | 0.585 | 1.000 | 2023 | tags=50%, list=17%, signal=60% |
| 351 | GOBP\_POSITIVE\_REGULATION\_OF\_VOLTAGE\_GATED\_CALCIUM\_CHANNEL\_ACTIVITY |  | 8 | 0.53 | 1.38 | 0.113 | 0.588 | 1.000 | 5689 | tags=100%, list=47%, signal=190% |
| 352 | GOBP\_NEGATIVE\_REGULATION\_OF\_HEXOKINASE\_ACTIVITY |  | 3 | 0.76 | 1.38 | 0.131 | 0.587 | 1.000 | 1823 | tags=67%, list=15%, signal=79% |
| 353 | GOBP\_NUCLEOTIDE\_SUGAR\_METABOLIC\_PROCESS |  | 31 | 0.35 | 1.38 | 0.064 | 0.586 | 1.000 | 685 | tags=23%, list=6%, signal=24% |
| 354 | GOBP\_L\_SERINE\_METABOLIC\_PROCESS |  | 7 | 0.55 | 1.38 | 0.124 | 0.585 | 1.000 | 1415 | tags=57%, list=12%, signal=65% |
| 355 | GOBP\_GDP\_MANNOSE\_METABOLIC\_PROCESS |  | 5 | 0.62 | 1.38 | 0.119 | 0.587 | 1.000 | 685 | tags=40%, list=6%, signal=42% |
| 356 | GOBP\_NEGATIVE\_REGULATION\_OF\_HAIR\_FOLLICLE\_DEVELOPMENT |  | 4 | 0.69 | 1.38 | 0.134 | 0.585 | 1.000 | 1188 | tags=25%, list=10%, signal=28% |
| 357 | GOBP\_NEURON\_FATE\_DETERMINATION |  | 3 | 0.76 | 1.38 | 0.141 | 0.586 | 1.000 | 1130 | tags=67%, list=9%, signal=74% |
| 358 | GOBP\_NEGATIVE\_REGULATION\_OF\_INSULIN\_LIKE\_GROWTH\_FACTOR\_RECEPTOR\_SIGNALING\_PATHWAY |  | 4 | 0.68 | 1.38 | 0.143 | 0.584 | 1.000 | 134 | tags=25%, list=1%, signal=25% |
| 359 | GOBP\_RELAXATION\_OF\_VASCULAR\_ASSOCIATED\_SMOOTH\_MUSCLE |  | 4 | 0.68 | 1.38 | 0.146 | 0.584 | 1.000 | 197 | tags=25%, list=2%, signal=25% |
| 360 | GOCC\_BCL\_2\_FAMILY\_PROTEIN\_COMPLEX |  | 4 | 0.68 | 1.38 | 0.139 | 0.584 | 1.000 | 775 | tags=50%, list=6%, signal=53% |
| 361 | GOBP\_AMINOGLYCOSIDE\_ANTIBIOTIC\_METABOLIC\_PROCESS |  | 5 | 0.63 | 1.38 | 0.131 | 0.584 | 1.000 | 1464 | tags=40%, list=12%, signal=46% |
| 362 | GOBP\_NEUROEPITHELIAL\_CELL\_DIFFERENTIATION |  | 12 | 0.46 | 1.38 | 0.100 | 0.583 | 1.000 | 1084 | tags=33%, list=9%, signal=37% |
| 363 | GOBP\_ROSTROCAUDAL\_NEURAL\_TUBE\_PATTERNING |  | 5 | 0.62 | 1.37 | 0.127 | 0.586 | 1.000 | 1130 | tags=80%, list=9%, signal=88% |
| 364 | GOBP\_DETECTION\_OF\_VIRUS |  | 4 | 0.68 | 1.37 | 0.137 | 0.595 | 1.000 | 159 | tags=50%, list=1%, signal=51% |
| 365 | GOBP\_CELLULAR\_RESPONSE\_TO\_TOXIC\_SUBSTANCE |  | 70 | 0.29 | 1.37 | 0.015 | 0.594 | 1.000 | 1821 | tags=27%, list=15%, signal=32% |
| 366 | GOBP\_ACROSOME\_REACTION |  | 15 | 0.42 | 1.37 | 0.102 | 0.593 | 1.000 | 580 | tags=27%, list=5%, signal=28% |
| 367 | GOMF\_RRNA\_ADENINE\_METHYLTRANSFERASE\_ACTIVITY |  | 9 | 0.50 | 1.37 | 0.125 | 0.592 | 1.000 | 1916 | tags=56%, list=16%, signal=66% |
| 368 | GOMF\_APOLIPOPROTEIN\_RECEPTOR\_BINDING |  | 3 | 0.76 | 1.37 | 0.138 | 0.593 | 1.000 | 158 | tags=33%, list=1%, signal=34% |
| 369 | GOBP\_INTERFERON\_ALPHA\_PRODUCTION |  | 19 | 0.39 | 1.37 | 0.088 | 0.592 | 1.000 | 536 | tags=26%, list=4%, signal=28% |
| 370 | GOMF\_L\_ASPARTATE\_TRANSMEMBRANE\_TRANSPORTER\_ACTIVITY |  | 7 | 0.55 | 1.37 | 0.130 | 0.591 | 1.000 | 1278 | tags=43%, list=11%, signal=48% |
| 371 | GOBP\_REGULATION\_OF\_GLUCAGON\_SECRETION |  | 5 | 0.62 | 1.37 | 0.135 | 0.595 | 1.000 | 689 | tags=60%, list=6%, signal=64% |
| 372 | GOBP\_L\_ASPARTATE\_TRANSMEMBRANE\_TRANSPORT |  | 8 | 0.53 | 1.36 | 0.131 | 0.595 | 1.000 | 1278 | tags=38%, list=11%, signal=42% |
| 373 | GOBP\_PLASMA\_MEMBRANE\_REPAIR |  | 9 | 0.50 | 1.36 | 0.121 | 0.594 | 1.000 | 935 | tags=33%, list=8%, signal=36% |
| 374 | GOBP\_REGULATION\_OF\_POLYAMINE\_TRANSMEMBRANE\_TRANSPORT |  | 4 | 0.67 | 1.36 | 0.149 | 0.596 | 1.000 | 1035 | tags=50%, list=9%, signal=55% |
| 375 | GOBP\_ETHER\_METABOLIC\_PROCESS |  | 20 | 0.39 | 1.36 | 0.077 | 0.594 | 1.000 | 1468 | tags=35%, list=12%, signal=40% |
| 376 | GOBP\_MEDIUM\_CHAIN\_FATTY\_ACID\_BIOSYNTHETIC\_PROCESS |  | 5 | 0.63 | 1.36 | 0.137 | 0.593 | 1.000 | 201 | tags=40%, list=2%, signal=41% |
| 377 | GOBP\_PROTEIN\_NEDDYLATION |  | 17 | 0.41 | 1.36 | 0.105 | 0.593 | 1.000 | 1495 | tags=35%, list=12%, signal=40% |
| 378 | GOBP\_INSULIN\_RECEPTOR\_SIGNALING\_PATHWAY |  | 111 | 0.27 | 1.36 | 0.018 | 0.595 | 1.000 | 234 | tags=12%, list=2%, signal=12% |
| 379 | GOBP\_ESTABLISHMENT\_OR\_MAINTENANCE\_OF\_TRANSMEMBRANE\_ELECTROCHEMICAL\_GRADIENT |  | 5 | 0.61 | 1.36 | 0.138 | 0.596 | 1.000 | 775 | tags=40%, list=6%, signal=43% |
| 380 | GOMF\_ORNITHINE\_DECARBOXYLASE\_REGULATOR\_ACTIVITY |  | 4 | 0.67 | 1.36 | 0.140 | 0.597 | 1.000 | 1035 | tags=50%, list=9%, signal=55% |
| 381 | GOMF\_UBIQUITIN\_LIKE\_PROTEIN\_SPECIFIC\_PROTEASE\_ACTIVITY |  | 16 | 0.41 | 1.36 | 0.110 | 0.598 | 1.000 | 1245 | tags=25%, list=10%, signal=28% |
| 382 | GOBP\_CELLULAR\_RESPONSE\_TO\_VIRUS |  | 39 | 0.33 | 1.36 | 0.043 | 0.597 | 1.000 | 678 | tags=18%, list=6%, signal=19% |
| 383 | GOCC\_PAM\_COMPLEX\_TIM23\_ASSOCIATED\_IMPORT\_MOTOR |  | 4 | 0.67 | 1.36 | 0.157 | 0.597 | 1.000 | 863 | tags=50%, list=7%, signal=54% |
| 384 | GOBP\_NEGATIVE\_REGULATION\_OF\_MYELOID\_CELL\_APOPTOTIC\_PROCESS |  | 6 | 0.58 | 1.36 | 0.152 | 0.597 | 1.000 | 2239 | tags=83%, list=19%, signal=102% |
| 385 | GOCC\_ACROSOMAL\_MEMBRANE |  | 9 | 0.49 | 1.36 | 0.129 | 0.597 | 1.000 | 1363 | tags=56%, list=11%, signal=63% |
| 386 | GOMF\_BMP\_RECEPTOR\_BINDING |  | 9 | 0.50 | 1.35 | 0.127 | 0.599 | 1.000 | 134 | tags=22%, list=1%, signal=22% |
| 387 | GOBP\_PYRIMIDINE\_NUCLEOSIDE\_TRIPHOSPHATE\_BIOSYNTHETIC\_PROCESS |  | 15 | 0.42 | 1.35 | 0.109 | 0.600 | 1.000 | 1093 | tags=20%, list=9%, signal=22% |
| 388 | GOBP\_REGULATION\_OF\_TRANSLATIONAL\_FIDELITY |  | 12 | 0.45 | 1.35 | 0.113 | 0.601 | 1.000 | 2555 | tags=50%, list=21%, signal=63% |
| 389 | GOBP\_NEGATIVE\_REGULATION\_OF\_COLLAGEN\_METABOLIC\_PROCESS |  | 7 | 0.55 | 1.35 | 0.136 | 0.601 | 1.000 | 721 | tags=57%, list=6%, signal=61% |
| 390 | GOMF\_ATPASE\_COUPLED\_LIPID\_TRANSMEMBRANE\_TRANSPORTER\_ACTIVITY |  | 4 | 0.67 | 1.35 | 0.156 | 0.603 | 1.000 | 2156 | tags=75%, list=18%, signal=91% |
| 391 | GOBP\_MITOCHONDRIAL\_MEMBRANE\_ORGANIZATION |  | 89 | 0.27 | 1.35 | 0.022 | 0.603 | 1.000 | 2341 | tags=38%, list=19%, signal=47% |
| 392 | GOMF\_CARBOXY\_LYASE\_ACTIVITY |  | 27 | 0.35 | 1.35 | 0.090 | 0.603 | 1.000 | 957 | tags=22%, list=8%, signal=24% |
| 393 | GOBP\_PROTEIN\_TARGETING\_TO\_MITOCHONDRION |  | 77 | 0.28 | 1.35 | 0.017 | 0.602 | 1.000 | 2576 | tags=36%, list=21%, signal=46% |
| 394 | GOBP\_AMINO\_SUGAR\_CATABOLIC\_PROCESS |  | 6 | 0.57 | 1.35 | 0.144 | 0.604 | 1.000 | 130 | tags=33%, list=1%, signal=34% |
| 395 | GOCC\_MICOS\_COMPLEX |  | 7 | 0.54 | 1.35 | 0.129 | 0.603 | 1.000 | 2013 | tags=57%, list=17%, signal=69% |
| 396 | GOMF\_MONOCARBOXYLIC\_ACID\_BINDING |  | 40 | 0.32 | 1.35 | 0.063 | 0.602 | 1.000 | 1003 | tags=28%, list=8%, signal=30% |
| 397 | GOBP\_DIPEPTIDE\_TRANSMEMBRANE\_TRANSPORT |  | 4 | 0.67 | 1.35 | 0.162 | 0.602 | 1.000 | 14 | tags=25%, list=0%, signal=25% |
| 398 | GOBP\_NEGATIVE\_REGULATION\_OF\_OSTEOBLAST\_PROLIFERATION |  | 8 | 0.52 | 1.35 | 0.129 | 0.602 | 1.000 | 431 | tags=38%, list=4%, signal=39% |
| 399 | GOMF\_WW\_DOMAIN\_BINDING |  | 16 | 0.41 | 1.35 | 0.093 | 0.601 | 1.000 | 523 | tags=25%, list=4%, signal=26% |
| 400 | GOMF\_NUCLEOBASE\_CONTAINING\_COMPOUND\_KINASE\_ACTIVITY |  | 31 | 0.34 | 1.34 | 0.089 | 0.601 | 1.000 | 1197 | tags=26%, list=10%, signal=29% |
| 401 | GOMF\_FATTY\_ACID\_BINDING |  | 22 | 0.37 | 1.34 | 0.098 | 0.601 | 1.000 | 416 | tags=23%, list=3%, signal=23% |
| 402 | GOMF\_SULFATE\_TRANSMEMBRANE\_TRANSPORTER\_ACTIVITY |  | 12 | 0.45 | 1.34 | 0.123 | 0.601 | 1.000 | 712 | tags=33%, list=6%, signal=35% |
| 403 | GOBP\_EYE\_PIGMENTATION |  | 3 | 0.75 | 1.34 | 0.167 | 0.600 | 1.000 | 489 | tags=67%, list=4%, signal=69% |
| 404 | GOCC\_SPECTRIN\_ASSOCIATED\_CYTOSKELETON |  | 7 | 0.54 | 1.34 | 0.145 | 0.599 | 1.000 | 2621 | tags=71%, list=22%, signal=91% |
| 405 | GOBP\_N\_ACETYLNEURAMINATE\_METABOLIC\_PROCESS |  | 9 | 0.50 | 1.34 | 0.137 | 0.598 | 1.000 | 186 | tags=22%, list=2%, signal=23% |
| 406 | GOBP\_PYRIMIDINE\_DEOXYRIBONUCLEOSIDE\_TRIPHOSPHATE\_METABOLIC\_PROCESS |  | 5 | 0.61 | 1.34 | 0.152 | 0.600 | 1.000 | 65 | tags=20%, list=1%, signal=20% |
| 407 | GOBP\_POSITIVE\_REGULATION\_OF\_TRANSCRIPTION\_FROM\_RNA\_POLYMERASE\_II\_PROMOTER\_IN\_RESPONSE\_TO\_OXIDATIVE\_STRESS |  | 4 | 0.66 | 1.34 | 0.157 | 0.599 | 1.000 | 2493 | tags=50%, list=21%, signal=63% |
| 408 | GOMF\_OXIDOREDUCTASE\_ACTIVITY\_ACTING\_ON\_A\_SULFUR\_GROUP\_OF\_DONORS\_NAD\_P\_AS\_ACCEPTOR |  | 9 | 0.50 | 1.34 | 0.147 | 0.604 | 1.000 | 6053 | tags=100%, list=50%, signal=201% |
| 409 | GOBP\_GLUTAMINE\_FAMILY\_AMINO\_ACID\_CATABOLIC\_PROCESS |  | 18 | 0.39 | 1.34 | 0.102 | 0.605 | 1.000 | 687 | tags=28%, list=6%, signal=29% |
| 410 | GOBP\_POSITIVE\_REGULATION\_OF\_TRANSFORMING\_GROWTH\_FACTOR\_BETA1\_PRODUCTION |  | 4 | 0.66 | 1.34 | 0.161 | 0.605 | 1.000 | 43 | tags=25%, list=0%, signal=25% |
| 411 | GOCC\_MITOCHONDRIAL\_OUTER\_MEMBRANE\_TRANSLOCASE\_COMPLEX |  | 5 | 0.62 | 1.34 | 0.152 | 0.606 | 1.000 | 2030 | tags=80%, list=17%, signal=96% |
| 412 | GOMF\_RACEMASE\_AND\_EPIMERASE\_ACTIVITY |  | 10 | 0.48 | 1.33 | 0.142 | 0.609 | 1.000 | 1315 | tags=50%, list=11%, signal=56% |
| 413 | GOBP\_RESPONSE\_TO\_AMINE |  | 29 | 0.35 | 1.33 | 0.093 | 0.613 | 1.000 | 1192 | tags=24%, list=10%, signal=27% |
| 414 | GOBP\_ESTABLISHMENT\_OF\_PROTEIN\_LOCALIZATION\_TO\_ENDOPLASMIC\_RETICULUM |  | 98 | 0.26 | 1.33 | 0.021 | 0.614 | 1.000 | 3085 | tags=46%, list=26%, signal=61% |
| 415 | GOBP\_REGULATION\_OF\_WATER\_LOSS\_VIA\_SKIN |  | 11 | 0.46 | 1.33 | 0.141 | 0.613 | 1.000 | 1030 | tags=36%, list=9%, signal=40% |
| 416 | GOBP\_2FE\_2S\_CLUSTER\_ASSEMBLY |  | 4 | 0.67 | 1.33 | 0.173 | 0.612 | 1.000 | 2191 | tags=75%, list=18%, signal=92% |
| 417 | GOCC\_MAST\_CELL\_GRANULE |  | 3 | 0.73 | 1.33 | 0.173 | 0.616 | 1.000 | 535 | tags=67%, list=4%, signal=70% |
| 418 | GOBP\_CLEAVAGE\_INVOLVED\_IN\_RRNA\_PROCESSING |  | 24 | 0.36 | 1.33 | 0.100 | 0.616 | 1.000 | 2425 | tags=42%, list=20%, signal=52% |
| 419 | GOBP\_REGULATION\_OF\_EPIDERMAL\_GROWTH\_FACTOR\_ACTIVATED\_RECEPTOR\_ACTIVITY |  | 21 | 0.37 | 1.33 | 0.101 | 0.616 | 1.000 | 450 | tags=14%, list=4%, signal=15% |
| 420 | GOBP\_DICARBOXYLIC\_ACID\_BIOSYNTHETIC\_PROCESS |  | 9 | 0.49 | 1.33 | 0.133 | 0.615 | 1.000 | 687 | tags=33%, list=6%, signal=35% |
| 421 | GOBP\_DEFENSE\_RESPONSE\_TO\_VIRUS |  | 135 | 0.25 | 1.33 | 0.013 | 0.614 | 1.000 | 758 | tags=16%, list=6%, signal=16% |
| 422 | GOBP\_POSITIVE\_REGULATION\_OF\_MITOCHONDRIAL\_TRANSLATION |  | 13 | 0.43 | 1.33 | 0.139 | 0.613 | 1.000 | 1610 | tags=31%, list=13%, signal=35% |
| 423 | GOBP\_METANEPHRIC\_MESENCHYMAL\_CELL\_DIFFERENTIATION |  | 4 | 0.66 | 1.33 | 0.170 | 0.613 | 1.000 | 1490 | tags=50%, list=12%, signal=57% |
| 424 | GOCC\_ALVEOLAR\_LAMELLAR\_BODY |  | 4 | 0.66 | 1.33 | 0.171 | 0.613 | 1.000 | 1787 | tags=50%, list=15%, signal=59% |
| 425 | GOMF\_PSEUDOURIDINE\_SYNTHASE\_ACTIVITY |  | 11 | 0.46 | 1.32 | 0.144 | 0.616 | 1.000 | 6506 | tags=100%, list=54%, signal=218% |
| 426 | GOCC\_CATION\_TRANSPORTING\_ATPASE\_COMPLEX |  | 5 | 0.61 | 1.32 | 0.163 | 0.615 | 1.000 | 79 | tags=40%, list=1%, signal=40% |
| 427 | GOBP\_PURINE\_NUCLEOSIDE\_DIPHOSPHATE\_BIOSYNTHETIC\_PROCESS |  | 5 | 0.61 | 1.32 | 0.154 | 0.615 | 1.000 | 1197 | tags=60%, list=10%, signal=67% |
| 428 | GOMF\_LIPID\_KINASE\_ACTIVITY |  | 4 | 0.65 | 1.32 | 0.167 | 0.614 | 1.000 | 743 | tags=50%, list=6%, signal=53% |
| 429 | GOMF\_OXIDOREDUCTASE\_ACTIVITY |  | 493 | 0.24 | 1.32 | 0.000 | 0.617 | 1.000 | 1674 | tags=27%, list=14%, signal=30% |
| 430 | GOBP\_POLYADENYLATION\_DEPENDENT\_SNORNA\_3\_END\_PROCESSING |  | 6 | 0.57 | 1.32 | 0.166 | 0.618 | 1.000 | 730 | tags=33%, list=6%, signal=35% |
| 431 | GOBP\_DETECTION\_OF\_CHEMICAL\_STIMULUS\_INVOLVED\_IN\_SENSORY\_PERCEPTION\_OF\_TASTE |  | 3 | 0.72 | 1.32 | 0.184 | 0.618 | 1.000 | 297 | tags=33%, list=2%, signal=34% |
| 432 | GOBP\_NEGATIVE\_REGULATION\_OF\_ATP\_METABOLIC\_PROCESS |  | 18 | 0.39 | 1.32 | 0.114 | 0.619 | 1.000 | 1060 | tags=22%, list=9%, signal=24% |
| 433 | GOBP\_NEGATIVE\_REGULATION\_OF\_VIRAL\_ENTRY\_INTO\_HOST\_CELL |  | 8 | 0.50 | 1.32 | 0.149 | 0.622 | 1.000 | 2441 | tags=63%, list=20%, signal=78% |
| 434 | GOBP\_NADP\_METABOLIC\_PROCESS |  | 26 | 0.35 | 1.32 | 0.106 | 0.621 | 1.000 | 702 | tags=23%, list=6%, signal=24% |
| 435 | GOBP\_REGULATION\_OF\_AMINO\_ACID\_TRANSPORT |  | 19 | 0.38 | 1.32 | 0.122 | 0.621 | 1.000 | 804 | tags=32%, list=7%, signal=34% |
| 436 | GOMF\_PROTEIN\_TRANSMEMBRANE\_TRANSPORTER\_ACTIVITY |  | 14 | 0.42 | 1.32 | 0.138 | 0.620 | 1.000 | 2030 | tags=43%, list=17%, signal=52% |
| 437 | GOMF\_NEUROTRANSMITTER\_RECEPTOR\_REGULATOR\_ACTIVITY |  | 5 | 0.61 | 1.31 | 0.173 | 0.622 | 1.000 | 612 | tags=40%, list=5%, signal=42% |
| 438 | GOBP\_LATERAL\_VENTRICLE\_DEVELOPMENT |  | 9 | 0.48 | 1.31 | 0.163 | 0.623 | 1.000 | 1548 | tags=44%, list=13%, signal=51% |
| 439 | GOCC\_BBSOME |  | 7 | 0.53 | 1.31 | 0.151 | 0.623 | 1.000 | 5701 | tags=100%, list=47%, signal=190% |
| 440 | GOBP\_POSITIVE\_REGULATION\_OF\_ESTABLISHMENT\_OF\_PROTEIN\_LOCALIZATION\_TO\_TELOMERE |  | 9 | 0.48 | 1.31 | 0.144 | 0.626 | 1.000 | 6258 | tags=100%, list=52%, signal=209% |
| 441 | GOBP\_RESPONSE\_TO\_VIRUS |  | 196 | 0.23 | 1.31 | 0.042 | 0.626 | 1.000 | 1373 | tags=21%, list=11%, signal=23% |
| 442 | GOBP\_MODULATION\_BY\_SYMBIONT\_OF\_HOST\_APOPTOTIC\_PROCESS |  | 3 | 0.72 | 1.31 | 0.182 | 0.627 | 1.000 | 568 | tags=67%, list=5%, signal=70% |
| 443 | GOBP\_SELENIUM\_COMPOUND\_METABOLIC\_PROCESS |  | 3 | 0.72 | 1.31 | 0.181 | 0.627 | 1.000 | 1959 | tags=67%, list=16%, signal=80% |
| 444 | GOBP\_NEUROENDOCRINE\_CELL\_DIFFERENTIATION |  | 6 | 0.56 | 1.31 | 0.167 | 0.625 | 1.000 | 1084 | tags=50%, list=9%, signal=55% |
| 445 | GOBP\_RIBONUCLEOSIDE\_DIPHOSPHATE\_CATABOLIC\_PROCESS |  | 5 | 0.59 | 1.31 | 0.164 | 0.624 | 1.000 | 1776 | tags=40%, list=15%, signal=47% |
| 446 | GOBP\_NEGATIVE\_REGULATION\_OF\_PHOSPHOLIPASE\_ACTIVITY |  | 6 | 0.56 | 1.31 | 0.159 | 0.625 | 1.000 | 909 | tags=50%, list=8%, signal=54% |
| 447 | GOBP\_CELLULAR\_POTASSIUM\_ION\_HOMEOSTASIS |  | 7 | 0.53 | 1.31 | 0.158 | 0.628 | 1.000 | 2376 | tags=57%, list=20%, signal=71% |
| 448 | GOBP\_NEGATIVE\_REGULATION\_OF\_GLYCOLYTIC\_PROCESS |  | 8 | 0.50 | 1.31 | 0.148 | 0.627 | 1.000 | 339 | tags=25%, list=3%, signal=26% |
| 449 | GOBP\_POSITIVE\_REGULATION\_OF\_RESPONSE\_TO\_WOUNDING |  | 47 | 0.30 | 1.30 | 0.074 | 0.627 | 1.000 | 1342 | tags=26%, list=11%, signal=29% |
| 450 | GOBP\_EPITHELIAL\_MESENCHYMAL\_CELL\_SIGNALING |  | 5 | 0.59 | 1.30 | 0.164 | 0.626 | 1.000 | 1723 | tags=40%, list=14%, signal=47% |
| 451 | GOBP\_REGULATION\_OF\_MOTOR\_NEURON\_APOPTOTIC\_PROCESS |  | 4 | 0.65 | 1.30 | 0.175 | 0.626 | 1.000 | 4260 | tags=100%, list=35%, signal=155% |
| 452 | GOBP\_CELLULAR\_RESPONSE\_TO\_POTASSIUM\_ION |  | 4 | 0.65 | 1.30 | 0.174 | 0.625 | 1.000 | 1482 | tags=50%, list=12%, signal=57% |
| 453 | GOBP\_TRANSFORMING\_GROWTH\_FACTOR\_BETA1\_PRODUCTION |  | 10 | 0.46 | 1.30 | 0.150 | 0.625 | 1.000 | 1340 | tags=30%, list=11%, signal=34% |
| 454 | GOBP\_MUCOCILIARY\_CLEARANCE |  | 4 | 0.65 | 1.30 | 0.179 | 0.625 | 1.000 | 1143 | tags=50%, list=10%, signal=55% |
| 455 | GOBP\_COPPER\_ION\_IMPORT |  | 4 | 0.65 | 1.30 | 0.189 | 0.626 | 1.000 | 363 | tags=50%, list=3%, signal=52% |
| 456 | GOBP\_NEGATIVE\_REGULATION\_OF\_LIPOPROTEIN\_PARTICLE\_CLEARANCE |  | 4 | 0.64 | 1.30 | 0.184 | 0.629 | 1.000 | 158 | tags=25%, list=1%, signal=25% |
| 457 | GOMF\_EPINEPHRINE\_BINDING |  | 5 | 0.60 | 1.30 | 0.173 | 0.628 | 1.000 | 4823 | tags=100%, list=40%, signal=167% |
| 458 | GOBP\_NEGATIVE\_REGULATION\_OF\_PEPTIDE\_HORMONE\_SECRETION |  | 24 | 0.36 | 1.30 | 0.116 | 0.632 | 1.000 | 1569 | tags=29%, list=13%, signal=33% |
| 459 | GOBP\_POSITIVE\_REGULATION\_OF\_INTRINSIC\_APOPTOTIC\_SIGNALING\_PATHWAY\_BY\_P53\_CLASS\_MEDIATOR |  | 3 | 0.72 | 1.29 | 0.201 | 0.643 | 1.000 | 3414 | tags=100%, list=28%, signal=140% |
| 460 | GOMF\_C\_X\_C\_CHEMOKINE\_RECEPTOR\_ACTIVITY |  | 4 | 0.64 | 1.29 | 0.187 | 0.644 | 1.000 | 701 | tags=50%, list=6%, signal=53% |
| 461 | GOMF\_PROTEIN\_DISULFIDE\_OXIDOREDUCTASE\_ACTIVITY |  | 19 | 0.37 | 1.29 | 0.150 | 0.645 | 1.000 | 2191 | tags=47%, list=18%, signal=58% |
| 462 | GOBP\_REGULATION\_OF\_FATTY\_ACID\_OXIDATION |  | 28 | 0.34 | 1.29 | 0.118 | 0.644 | 1.000 | 1003 | tags=21%, list=8%, signal=23% |
| 463 | GOCC\_NUCLEOLAR\_RIBONUCLEASE\_P\_COMPLEX |  | 3 | 0.71 | 1.29 | 0.208 | 0.644 | 1.000 | 871 | tags=67%, list=7%, signal=72% |
| 464 | GOBP\_SENSORY\_PERCEPTION\_OF\_TASTE |  | 17 | 0.38 | 1.29 | 0.148 | 0.646 | 1.000 | 297 | tags=24%, list=2%, signal=24% |
| 465 | GOMF\_ION\_TRANSMEMBRANE\_TRANSPORTER\_ACTIVITY\_PHOSPHORYLATIVE\_MECHANISM |  | 11 | 0.44 | 1.29 | 0.172 | 0.647 | 1.000 | 61 | tags=18%, list=1%, signal=18% |
| 466 | GOBP\_RIBOSOMAL\_LARGE\_SUBUNIT\_EXPORT\_FROM\_NUCLEUS |  | 7 | 0.52 | 1.29 | 0.170 | 0.646 | 1.000 | 262 | tags=14%, list=2%, signal=15% |
| 467 | GOMF\_C\_ACETYLTRANSFERASE\_ACTIVITY |  | 6 | 0.55 | 1.29 | 0.174 | 0.645 | 1.000 | 1648 | tags=50%, list=14%, signal=58% |
| 468 | GOBP\_PROTEIN\_RETENTION\_IN\_ER\_LUMEN |  | 3 | 0.71 | 1.29 | 0.203 | 0.648 | 1.000 | 3474 | tags=100%, list=29%, signal=141% |
| 469 | GOCC\_WEIBEL\_PALADE\_BODY |  | 5 | 0.59 | 1.29 | 0.187 | 0.648 | 1.000 | 4974 | tags=100%, list=41%, signal=171% |
| 470 | GOBP\_OLFACTORY\_BEHAVIOR |  | 4 | 0.64 | 1.28 | 0.182 | 0.651 | 1.000 | 265 | tags=25%, list=2%, signal=26% |
| 471 | GOCC\_UNIPLEX\_COMPLEX |  | 6 | 0.55 | 1.28 | 0.184 | 0.656 | 1.000 | 883 | tags=33%, list=7%, signal=36% |
| 472 | GOBP\_NEGATIVE\_REGULATION\_OF\_VASCULAR\_ASSOCIATED\_SMOOTH\_MUSCLE\_CELL\_PROLIFERATION |  | 15 | 0.40 | 1.28 | 0.152 | 0.656 | 1.000 | 843 | tags=27%, list=7%, signal=29% |
| 473 | GOBP\_MHC\_PROTEIN\_COMPLEX\_ASSEMBLY |  | 3 | 0.71 | 1.28 | 0.220 | 0.655 | 1.000 | 606 | tags=33%, list=5%, signal=35% |
| 474 | GOBP\_NEGATIVE\_REGULATION\_OF\_HEMATOPOIETIC\_STEM\_CELL\_DIFFERENTIATION |  | 3 | 0.71 | 1.28 | 0.209 | 0.653 | 1.000 | 2493 | tags=67%, list=21%, signal=84% |
| 475 | GOBP\_L\_GLUTAMATE\_TRANSMEMBRANE\_TRANSPORT |  | 14 | 0.40 | 1.28 | 0.159 | 0.653 | 1.000 | 812 | tags=21%, list=7%, signal=23% |
| 476 | GOMF\_OXIDOREDUCTASE\_ACTIVITY\_ACTING\_ON\_THE\_CH\_CH\_GROUP\_OF\_DONORS |  | 48 | 0.29 | 1.28 | 0.067 | 0.657 | 1.000 | 1483 | tags=27%, list=12%, signal=31% |
| 477 | GOMF\_ARACHIDONIC\_ACID\_MONOOXYGENASE\_ACTIVITY |  | 3 | 0.70 | 1.28 | 0.221 | 0.656 | 1.000 | 1398 | tags=67%, list=12%, signal=75% |
| 478 | GOMF\_ALDITOL\_NADPPLUS\_1\_OXIDOREDUCTASE\_ACTIVITY |  | 7 | 0.52 | 1.28 | 0.181 | 0.656 | 1.000 | 1464 | tags=43%, list=12%, signal=49% |
| 479 | GOBP\_NEGATIVE\_REGULATION\_OF\_PHOSPHOLIPID\_METABOLIC\_PROCESS |  | 5 | 0.59 | 1.28 | 0.192 | 0.655 | 1.000 | 4941 | tags=100%, list=41%, signal=170% |
| 480 | GOBP\_AMINO\_SUGAR\_METABOLIC\_PROCESS |  | 29 | 0.33 | 1.28 | 0.120 | 0.654 | 1.000 | 583 | tags=21%, list=5%, signal=22% |
| 481 | GOMF\_CALCIUM\_DEPENDENT\_PHOSPHOLIPASE\_A2\_ACTIVITY |  | 5 | 0.58 | 1.28 | 0.190 | 0.655 | 1.000 | 299 | tags=40%, list=2%, signal=41% |
| 482 | GOBP\_POSITIVE\_REGULATION\_OF\_FATTY\_ACID\_METABOLIC\_PROCESS |  | 27 | 0.33 | 1.28 | 0.128 | 0.655 | 1.000 | 339 | tags=19%, list=3%, signal=19% |
| 483 | GOBP\_REGULATION\_OF\_CELLULAR\_PH\_REDUCTION |  | 6 | 0.53 | 1.27 | 0.184 | 0.657 | 1.000 | 179 | tags=33%, list=1%, signal=34% |
| 484 | GOMF\_UBIQUITIN\_LIKE\_PROTEIN\_CONJUGATING\_ENZYME\_ACTIVITY |  | 32 | 0.32 | 1.27 | 0.111 | 0.657 | 1.000 | 1809 | tags=25%, list=15%, signal=29% |
| 485 | GOMF\_ISOMERASE\_ACTIVITY |  | 116 | 0.25 | 1.27 | 0.052 | 0.657 | 1.000 | 1658 | tags=24%, list=14%, signal=28% |
| 486 | GOBP\_NEGATIVE\_REGULATION\_OF\_MITOCHONDRIAL\_OUTER\_MEMBRANE\_PERMEABILIZATION\_INVOLVED\_IN\_APOPTOTIC\_SIGNALING\_PATHWAY |  | 5 | 0.59 | 1.27 | 0.204 | 0.656 | 1.000 | 422 | tags=40%, list=4%, signal=41% |
| 487 | GOBP\_RRNA\_METHYLATION |  | 17 | 0.38 | 1.27 | 0.157 | 0.656 | 1.000 | 1916 | tags=47%, list=16%, signal=56% |
| 488 | GOBP\_REGULATION\_OF\_LYSOSOMAL\_LUMEN\_PH |  | 12 | 0.42 | 1.27 | 0.166 | 0.654 | 1.000 | 664 | tags=25%, list=6%, signal=26% |
| 489 | GOBP\_PENTOSE\_METABOLIC\_PROCESS |  | 10 | 0.45 | 1.27 | 0.172 | 0.653 | 1.000 | 702 | tags=40%, list=6%, signal=42% |
| 490 | GOBP\_BICARBONATE\_TRANSPORT |  | 31 | 0.32 | 1.27 | 0.106 | 0.656 | 1.000 | 452 | tags=19%, list=4%, signal=20% |
| 491 | GOBP\_REGULATION\_OF\_MITOCHONDRIAL\_MEMBRANE\_PERMEABILITY |  | 48 | 0.29 | 1.27 | 0.086 | 0.657 | 1.000 | 1244 | tags=23%, list=10%, signal=25% |
| 492 | GOMF\_TRANSCRIPTION\_REGULATOR\_INHIBITOR\_ACTIVITY |  | 14 | 0.41 | 1.27 | 0.155 | 0.660 | 1.000 | 277 | tags=21%, list=2%, signal=22% |
| 493 | GOBP\_C4\_DICARBOXYLATE\_TRANSPORT |  | 18 | 0.37 | 1.27 | 0.151 | 0.660 | 1.000 | 1482 | tags=33%, list=12%, signal=38% |
| 494 | GOBP\_VIRAL\_BUDDING\_VIA\_HOST\_ESCRT\_COMPLEX |  | 19 | 0.36 | 1.27 | 0.136 | 0.660 | 1.000 | 3080 | tags=63%, list=26%, signal=85% |
| 495 | GOBP\_MESENCHYMAL\_CELL\_DIFFERENTIATION\_INVOLVED\_IN\_KIDNEY\_DEVELOPMENT |  | 6 | 0.53 | 1.27 | 0.186 | 0.661 | 1.000 | 1723 | tags=50%, list=14%, signal=58% |
| 496 | GOBP\_ISOPRENOID\_CATABOLIC\_PROCESS |  | 6 | 0.54 | 1.27 | 0.202 | 0.660 | 1.000 | 2190 | tags=50%, list=18%, signal=61% |
| 497 | GOMF\_TRANSLATION\_ELONGATION\_FACTOR\_ACTIVITY |  | 14 | 0.40 | 1.27 | 0.155 | 0.661 | 1.000 | 7265 | tags=100%, list=60%, signal=253% |
| 498 | GOBP\_REGULATION\_OF\_OXIDATIVE\_PHOSPHORYLATION |  | 10 | 0.44 | 1.26 | 0.172 | 0.660 | 1.000 | 1761 | tags=40%, list=15%, signal=47% |
| 499 | GOCC\_MMXD\_COMPLEX |  | 3 | 0.69 | 1.26 | 0.224 | 0.660 | 1.000 | 2025 | tags=67%, list=17%, signal=80% |
| 500 | GOBP\_URETER\_MORPHOGENESIS |  | 5 | 0.58 | 1.26 | 0.190 | 0.659 | 1.000 | 2453 | tags=60%, list=20%, signal=75% |
| 501 | GOMF\_HYALURONONGLUCOSAMINIDASE\_ACTIVITY |  | 4 | 0.63 | 1.26 | 0.203 | 0.659 | 1.000 | 959 | tags=50%, list=8%, signal=54% |
| 502 | GOBP\_PYRIMIDINE\_NUCLEOSIDE\_DIPHOSPHATE\_METABOLIC\_PROCESS |  | 5 | 0.58 | 1.26 | 0.199 | 0.659 | 1.000 | 65 | tags=20%, list=1%, signal=20% |
| 503 | GOMF\_SODIUM\_CHANNEL\_ACTIVITY |  | 22 | 0.35 | 1.26 | 0.156 | 0.658 | 1.000 | 472 | tags=18%, list=4%, signal=19% |
| 504 | GOMF\_ANDROSTAN\_3\_ALPHA\_17\_BETA\_DIOL\_DEHYDROGENASE\_ACTIVITY |  | 3 | 0.70 | 1.26 | 0.230 | 0.662 | 1.000 | 3631 | tags=100%, list=30%, signal=143% |
| 505 | GOBP\_ALPHA\_AMINO\_ACID\_BIOSYNTHETIC\_PROCESS |  | 43 | 0.29 | 1.26 | 0.106 | 0.665 | 1.000 | 1955 | tags=30%, list=16%, signal=36% |
| 506 | GOBP\_POSITIVE\_REGULATION\_OF\_INTERLEUKIN\_13\_PRODUCTION |  | 5 | 0.58 | 1.26 | 0.199 | 0.664 | 1.000 | 749 | tags=40%, list=6%, signal=43% |
| 507 | GOMF\_CALCIUM\_INDEPENDENT\_PHOSPHOLIPASE\_A2\_ACTIVITY |  | 7 | 0.51 | 1.26 | 0.183 | 0.663 | 1.000 | 299 | tags=29%, list=2%, signal=29% |
| 508 | GOBP\_HEAT\_ACCLIMATION |  | 3 | 0.70 | 1.26 | 0.232 | 0.663 | 1.000 | 116 | tags=33%, list=1%, signal=34% |
| 509 | GOBP\_POSITIVE\_REGULATION\_OF\_PROSTAGLANDIN\_BIOSYNTHETIC\_PROCESS |  | 5 | 0.57 | 1.26 | 0.188 | 0.664 | 1.000 | 1237 | tags=60%, list=10%, signal=67% |
| 510 | GOBP\_AMINOACYL\_TRNA\_METABOLISM\_INVOLVED\_IN\_TRANSLATIONAL\_FIDELITY |  | 8 | 0.48 | 1.26 | 0.179 | 0.664 | 1.000 | 2555 | tags=50%, list=21%, signal=63% |
| 511 | GOBP\_MESENCHYME\_MIGRATION |  | 5 | 0.58 | 1.26 | 0.203 | 0.662 | 1.000 | 1505 | tags=40%, list=13%, signal=46% |
| 512 | GOBP\_MESONEPHRIC\_TUBULE\_FORMATION |  | 3 | 0.69 | 1.26 | 0.224 | 0.662 | 1.000 | 1490 | tags=67%, list=12%, signal=76% |
| 513 | GOBP\_RESPONSE\_TO\_COBALT\_ION |  | 8 | 0.48 | 1.26 | 0.197 | 0.661 | 1.000 | 514 | tags=25%, list=4%, signal=26% |
| 514 | GOBP\_REGULATION\_OF\_OLIGODENDROCYTE\_DIFFERENTIATION |  | 27 | 0.33 | 1.25 | 0.132 | 0.663 | 1.000 | 1084 | tags=30%, list=9%, signal=32% |
| 515 | GOBP\_CYSTEINE\_CATABOLIC\_PROCESS |  | 3 | 0.69 | 1.25 | 0.214 | 0.663 | 1.000 | 937 | tags=33%, list=8%, signal=36% |
| 516 | GOMF\_HYDRO\_LYASE\_ACTIVITY |  | 45 | 0.29 | 1.25 | 0.115 | 0.663 | 1.000 | 1648 | tags=33%, list=14%, signal=38% |
| 517 | GOBP\_NUCLEAR\_TRANSCRIBED\_MRNA\_CATABOLIC\_PROCESS\_EXONUCLEOLYTIC\_3\_5 |  | 10 | 0.45 | 1.25 | 0.180 | 0.662 | 1.000 | 2421 | tags=50%, list=20%, signal=63% |
| 518 | GOBP\_POSITIVE\_REGULATION\_OF\_NEURON\_PROJECTION\_REGENERATION |  | 9 | 0.46 | 1.25 | 0.191 | 0.667 | 1.000 | 822 | tags=22%, list=7%, signal=24% |
| 519 | GOMF\_AMINOACYL\_TRNA\_EDITING\_ACTIVITY |  | 8 | 0.48 | 1.25 | 0.190 | 0.666 | 1.000 | 2555 | tags=50%, list=21%, signal=63% |
| 520 | GOBP\_NEGATIVE\_REGULATION\_OF\_CELL\_FATE\_COMMITMENT |  | 7 | 0.50 | 1.25 | 0.183 | 0.669 | 1.000 | 2770 | tags=57%, list=23%, signal=74% |
| 521 | GOBP\_NEGATIVE\_REGULATION\_OF\_TYPE\_I\_INTERFERON\_PRODUCTION |  | 28 | 0.32 | 1.25 | 0.152 | 0.669 | 1.000 | 957 | tags=25%, list=8%, signal=27% |
| 522 | GOBP\_NEGATIVE\_REGULATION\_OF\_RELEASE\_OF\_CYTOCHROME\_C\_FROM\_MITOCHONDRIA |  | 12 | 0.41 | 1.25 | 0.179 | 0.668 | 1.000 | 1146 | tags=42%, list=10%, signal=46% |
| 523 | GOCC\_TRANSCRIPTION\_FACTOR\_TFIIA\_COMPLEX |  | 4 | 0.61 | 1.25 | 0.203 | 0.668 | 1.000 | 1319 | tags=25%, list=11%, signal=28% |
| 524 | GOCC\_LAMELLAR\_BODY |  | 7 | 0.50 | 1.25 | 0.192 | 0.667 | 1.000 | 1787 | tags=43%, list=15%, signal=50% |
| 525 | GOBP\_MALE\_SEX\_DETERMINATION |  | 6 | 0.53 | 1.25 | 0.197 | 0.668 | 1.000 | 2089 | tags=50%, list=17%, signal=60% |
| 526 | GOMF\_TRANSFERASE\_ACTIVITY\_TRANSFERRING\_ALDEHYDE\_OR\_KETONIC\_GROUPS |  | 3 | 0.69 | 1.25 | 0.225 | 0.670 | 1.000 | 1465 | tags=67%, list=12%, signal=76% |
| 527 | GOBP\_PRIMARY\_ALCOHOL\_CATABOLIC\_PROCESS |  | 4 | 0.62 | 1.25 | 0.216 | 0.669 | 1.000 | 4510 | tags=100%, list=38%, signal=160% |
| 528 | GOBP\_RRNA\_TRANSPORT |  | 4 | 0.62 | 1.25 | 0.207 | 0.669 | 1.000 | 2512 | tags=50%, list=21%, signal=63% |
| 529 | GOBP\_MITOCHONDRIAL\_ADP\_TRANSMEMBRANE\_TRANSPORT |  | 3 | 0.70 | 1.25 | 0.240 | 0.667 | 1.000 | 276 | tags=33%, list=2%, signal=34% |
| 530 | GOBP\_REGULATION\_OF\_MITOCHONDRIAL\_ATP\_SYNTHESIS\_COUPLED\_ELECTRON\_TRANSPORT |  | 5 | 0.56 | 1.24 | 0.205 | 0.671 | 1.000 | 863 | tags=40%, list=7%, signal=43% |
| 531 | GOBP\_REGULATION\_OF\_INTRACELLULAR\_LIPID\_TRANSPORT |  | 6 | 0.52 | 1.24 | 0.196 | 0.672 | 1.000 | 810 | tags=33%, list=7%, signal=36% |
| 532 | GOBP\_PROTEIN\_SIALYLATION |  | 3 | 0.69 | 1.24 | 0.254 | 0.675 | 1.000 | 2739 | tags=67%, list=23%, signal=86% |
| 533 | GOBP\_REGULATION\_OF\_SERINE\_TYPE\_PEPTIDASE\_ACTIVITY |  | 4 | 0.62 | 1.24 | 0.218 | 0.675 | 1.000 | 2623 | tags=50%, list=22%, signal=64% |
| 534 | GOMF\_CIS\_TRANS\_ISOMERASE\_ACTIVITY |  | 31 | 0.31 | 1.24 | 0.145 | 0.674 | 1.000 | 1175 | tags=19%, list=10%, signal=21% |
| 535 | GOBP\_MYD88\_INDEPENDENT\_TOLL\_LIKE\_RECEPTOR\_SIGNALING\_PATHWAY |  | 26 | 0.33 | 1.24 | 0.139 | 0.673 | 1.000 | 672 | tags=19%, list=6%, signal=20% |
| 536 | GOBP\_PROTOPORPHYRINOGEN\_IX\_BIOSYNTHETIC\_PROCESS |  | 7 | 0.49 | 1.24 | 0.205 | 0.677 | 1.000 | 249 | tags=14%, list=2%, signal=15% |
| 537 | GOBP\_NEGATIVE\_REGULATION\_OF\_SERINE\_TYPE\_PEPTIDASE\_ACTIVITY |  | 4 | 0.62 | 1.24 | 0.224 | 0.676 | 1.000 | 2623 | tags=50%, list=22%, signal=64% |
| 538 | GOBP\_SYNAPTIC\_TRANSMISSION\_DOPAMINERGIC |  | 13 | 0.40 | 1.24 | 0.195 | 0.676 | 1.000 | 969 | tags=23%, list=8%, signal=25% |
| 539 | GOMF\_OXIDOREDUCTASE\_ACTIVITY\_ACTING\_ON\_CH\_OH\_GROUP\_OF\_DONORS |  | 82 | 0.25 | 1.23 | 0.075 | 0.682 | 1.000 | 1648 | tags=27%, list=14%, signal=31% |
| 540 | GOBP\_POSITIVE\_REGULATION\_OF\_INTERFERON\_BETA\_PRODUCTION |  | 24 | 0.34 | 1.23 | 0.151 | 0.685 | 1.000 | 159 | tags=17%, list=1%, signal=17% |
| 541 | GOBP\_NEGATIVE\_REGULATION\_OF\_OXIDATIVE\_STRESS\_INDUCED\_CELL\_DEATH |  | 29 | 0.32 | 1.23 | 0.152 | 0.686 | 1.000 | 2060 | tags=41%, list=17%, signal=50% |
| 542 | GOBP\_MACROMOLECULE\_DEPALMITOYLATION |  | 11 | 0.41 | 1.23 | 0.194 | 0.686 | 1.000 | 1608 | tags=45%, list=13%, signal=52% |
| 543 | GOBP\_REGULATION\_OF\_THE\_FORCE\_OF\_HEART\_CONTRACTION |  | 17 | 0.37 | 1.23 | 0.183 | 0.687 | 1.000 | 558 | tags=18%, list=5%, signal=18% |
| 544 | GOMF\_S100\_PROTEIN\_BINDING |  | 9 | 0.45 | 1.23 | 0.217 | 0.688 | 1.000 | 1581 | tags=44%, list=13%, signal=51% |
| 545 | GOBP\_MITOCHONDRIAL\_ACETYL\_COA\_BIOSYNTHETIC\_PROCESS\_FROM\_PYRUVATE |  | 4 | 0.61 | 1.23 | 0.231 | 0.687 | 1.000 | 407 | tags=25%, list=3%, signal=26% |
| 546 | GOBP\_POSITIVE\_REGULATION\_OF\_WOUND\_HEALING |  | 38 | 0.30 | 1.23 | 0.127 | 0.686 | 1.000 | 1342 | tags=26%, list=11%, signal=30% |
| 547 | GOBP\_RIBOSOMAL\_LARGE\_SUBUNIT\_BIOGENESIS |  | 63 | 0.26 | 1.23 | 0.100 | 0.686 | 1.000 | 2783 | tags=37%, list=23%, signal=47% |
| 548 | GOBP\_INFLAMMATORY\_RESPONSE\_TO\_ANTIGENIC\_STIMULUS |  | 24 | 0.33 | 1.23 | 0.163 | 0.685 | 1.000 | 1445 | tags=25%, list=12%, signal=28% |
| 549 | GOBP\_VACUOLE\_FUSION |  | 3 | 0.68 | 1.23 | 0.256 | 0.684 | 1.000 | 577 | tags=33%, list=5%, signal=35% |
| 550 | GOBP\_NADH\_OXIDATION |  | 4 | 0.61 | 1.23 | 0.217 | 0.685 | 1.000 | 9 | tags=25%, list=0%, signal=25% |
| 551 | GOMF\_PHOSPHOTRANSFERASE\_ACTIVITY\_PHOSPHATE\_GROUP\_AS\_ACCEPTOR |  | 27 | 0.32 | 1.23 | 0.151 | 0.684 | 1.000 | 1197 | tags=26%, list=10%, signal=29% |
| 552 | GOMF\_3\_OXO\_ARACHIDOYL\_COA\_SYNTHASE\_ACTIVITY |  | 4 | 0.60 | 1.23 | 0.236 | 0.689 | 1.000 | 899 | tags=50%, list=7%, signal=54% |
| 553 | GOBP\_NEGATIVE\_REGULATION\_OF\_IMMATURE\_T\_CELL\_PROLIFERATION |  | 4 | 0.61 | 1.22 | 0.229 | 0.691 | 1.000 | 1723 | tags=50%, list=14%, signal=58% |
| 554 | GOBP\_AMMONIUM\_TRANSPORT |  | 7 | 0.49 | 1.22 | 0.213 | 0.690 | 1.000 | 1596 | tags=57%, list=13%, signal=66% |
| 555 | GOMF\_C4\_DICARBOXYLATE\_TRANSMEMBRANE\_TRANSPORTER\_ACTIVITY |  | 12 | 0.41 | 1.22 | 0.200 | 0.689 | 1.000 | 1278 | tags=33%, list=11%, signal=37% |
| 556 | GOBP\_FATTY\_ACID\_ELONGATION\_SATURATED\_FATTY\_ACID |  | 4 | 0.60 | 1.22 | 0.220 | 0.690 | 1.000 | 899 | tags=50%, list=7%, signal=54% |
| 557 | GOBP\_POSITIVE\_REGULATION\_OF\_LEUKOCYTE\_TETHERING\_OR\_ROLLING |  | 5 | 0.56 | 1.22 | 0.222 | 0.690 | 1.000 | 49 | tags=20%, list=0%, signal=20% |
| 558 | GOCC\_SEC61\_TRANSLOCON\_COMPLEX |  | 4 | 0.60 | 1.22 | 0.220 | 0.690 | 1.000 | 410 | tags=25%, list=3%, signal=26% |
| 559 | GOMF\_PROTEIN\_PHOSPHATASE\_2B\_BINDING |  | 4 | 0.61 | 1.22 | 0.235 | 0.691 | 1.000 | 79 | tags=25%, list=1%, signal=25% |
| 560 | GOBP\_POSITIVE\_REGULATION\_OF\_G\_PROTEIN\_COUPLED\_RECEPTOR\_SIGNALING\_PATHWAY |  | 11 | 0.42 | 1.22 | 0.206 | 0.694 | 1.000 | 3079 | tags=55%, list=26%, signal=73% |
| 561 | GOBP\_RESPONSE\_TO\_SELENIUM\_ION |  | 8 | 0.46 | 1.22 | 0.219 | 0.695 | 1.000 | 1821 | tags=50%, list=15%, signal=59% |
| 562 | GOBP\_NEGATIVE\_REGULATION\_OF\_INNATE\_IMMUNE\_RESPONSE |  | 30 | 0.31 | 1.22 | 0.159 | 0.697 | 1.000 | 1017 | tags=23%, list=8%, signal=25% |
| 563 | GOBP\_GASTRIC\_ACID\_SECRETION |  | 9 | 0.45 | 1.22 | 0.200 | 0.700 | 1.000 | 1234 | tags=67%, list=10%, signal=74% |
| 564 | GOCC\_ZONA\_PELLUCIDA\_RECEPTOR\_COMPLEX |  | 5 | 0.55 | 1.22 | 0.228 | 0.699 | 1.000 | 5355 | tags=100%, list=45%, signal=180% |
| 565 | GOMF\_INTRAMOLECULAR\_OXIDOREDUCTASE\_ACTIVITY\_INTERCONVERTING\_ALDOSES\_AND\_KETOSES |  | 8 | 0.46 | 1.21 | 0.219 | 0.699 | 1.000 | 1092 | tags=38%, list=9%, signal=41% |
| 566 | GOBP\_REGULATION\_OF\_MITOCHONDRIAL\_GENE\_EXPRESSION |  | 23 | 0.33 | 1.21 | 0.160 | 0.698 | 1.000 | 697 | tags=17%, list=6%, signal=18% |
| 567 | GOMF\_RETINOIC\_ACID\_BINDING |  | 9 | 0.44 | 1.21 | 0.215 | 0.700 | 1.000 | 817 | tags=44%, list=7%, signal=48% |
| 568 | GOBP\_NADH\_METABOLIC\_PROCESS |  | 31 | 0.31 | 1.21 | 0.176 | 0.698 | 1.000 | 1251 | tags=23%, list=10%, signal=25% |
| 569 | GOBP\_RIBONUCLEOSIDE\_TRIPHOSPHATE\_METABOLIC\_PROCESS |  | 47 | 0.28 | 1.21 | 0.129 | 0.698 | 1.000 | 1267 | tags=21%, list=11%, signal=24% |
| 570 | GOBP\_PROTEIN\_TRANSMEMBRANE\_IMPORT\_INTO\_INTRACELLULAR\_ORGANELLE |  | 30 | 0.31 | 1.21 | 0.155 | 0.700 | 1.000 | 2491 | tags=33%, list=21%, signal=42% |
| 571 | GOMF\_AMMONIUM\_TRANSMEMBRANE\_TRANSPORTER\_ACTIVITY |  | 7 | 0.49 | 1.21 | 0.215 | 0.699 | 1.000 | 1596 | tags=57%, list=13%, signal=66% |
| 572 | GOBP\_CELLULAR\_RESPONSE\_TO\_FOLLICLE\_STIMULATING\_HORMONE\_STIMULUS |  | 5 | 0.55 | 1.21 | 0.218 | 0.700 | 1.000 | 1388 | tags=40%, list=12%, signal=45% |
| 573 | GOCC\_PEPTIDASE\_COMPLEX |  | 73 | 0.25 | 1.21 | 0.115 | 0.699 | 1.000 | 3133 | tags=51%, list=26%, signal=68% |
| 574 | GOCC\_GOLGI\_TRANSPORT\_COMPLEX |  | 13 | 0.40 | 1.21 | 0.218 | 0.698 | 1.000 | 744 | tags=23%, list=6%, signal=25% |
| 575 | GOMF\_CARBON\_CARBON\_LYASE\_ACTIVITY |  | 38 | 0.29 | 1.21 | 0.137 | 0.699 | 1.000 | 1191 | tags=21%, list=10%, signal=23% |
| 576 | GOMF\_P\_TYPE\_TRANSMEMBRANE\_TRANSPORTER\_ACTIVITY |  | 3 | 0.67 | 1.21 | 0.250 | 0.699 | 1.000 | 48 | tags=33%, list=0%, signal=33% |
| 577 | GOBP\_MEMBRANE\_REPOLARIZATION\_DURING\_ACTION\_POTENTIAL |  | 17 | 0.36 | 1.21 | 0.196 | 0.700 | 1.000 | 7665 | tags=100%, list=64%, signal=276% |
| 578 | GOBP\_HYDROGEN\_PEROXIDE\_METABOLIC\_PROCESS |  | 28 | 0.31 | 1.21 | 0.154 | 0.705 | 1.000 | 646 | tags=18%, list=5%, signal=19% |
| 579 | GOBP\_SKELETAL\_MUSCLE\_SATELLITE\_CELL\_DIFFERENTIATION |  | 5 | 0.55 | 1.21 | 0.234 | 0.703 | 1.000 | 5394 | tags=100%, list=45%, signal=181% |
| 580 | GOBP\_RIBOSOMAL\_SMALL\_SUBUNIT\_ASSEMBLY |  | 15 | 0.38 | 1.21 | 0.211 | 0.703 | 1.000 | 1804 | tags=47%, list=15%, signal=55% |
| 581 | GOBP\_POSITIVE\_REGULATION\_OF\_NEUROTRANSMITTER\_UPTAKE |  | 3 | 0.67 | 1.21 | 0.268 | 0.702 | 1.000 | 293 | tags=33%, list=2%, signal=34% |
| 582 | GOBP\_REGULATION\_OF\_ACUTE\_INFLAMMATORY\_RESPONSE |  | 26 | 0.32 | 1.21 | 0.167 | 0.702 | 1.000 | 83 | tags=12%, list=1%, signal=12% |
| 583 | GOBP\_RRNA\_MODIFICATION |  | 25 | 0.32 | 1.20 | 0.176 | 0.703 | 1.000 | 1916 | tags=40%, list=16%, signal=47% |
| 584 | GOCC\_EUKARYOTIC\_TRANSLATION\_INITIATION\_FACTOR\_2\_COMPLEX |  | 4 | 0.60 | 1.20 | 0.243 | 0.702 | 1.000 | 4833 | tags=100%, list=40%, signal=167% |
| 585 | GOBP\_POLYPRENOL\_METABOLIC\_PROCESS |  | 6 | 0.51 | 1.20 | 0.233 | 0.706 | 1.000 | 2774 | tags=50%, list=23%, signal=65% |
| 586 | GOMF\_PALMITOYL\_PROTEIN\_HYDROLASE\_ACTIVITY |  | 11 | 0.41 | 1.20 | 0.212 | 0.705 | 1.000 | 1608 | tags=45%, list=13%, signal=52% |
| 587 | GOMF\_CXCR\_CHEMOKINE\_RECEPTOR\_BINDING |  | 4 | 0.60 | 1.20 | 0.253 | 0.710 | 1.000 | 1235 | tags=50%, list=10%, signal=56% |
| 588 | GOBP\_SPERMINE\_METABOLIC\_PROCESS |  | 4 | 0.60 | 1.20 | 0.244 | 0.710 | 1.000 | 2144 | tags=50%, list=18%, signal=61% |
| 589 | GOMF\_OXIDOREDUCTASE\_ACTIVITY\_ACTING\_ON\_OTHER\_NITROGENOUS\_COMPOUNDS\_AS\_DONORS |  | 4 | 0.60 | 1.20 | 0.252 | 0.711 | 1.000 | 2041 | tags=75%, list=17%, signal=90% |
| 590 | GOBP\_POSITIVE\_REGULATION\_OF\_VIRAL\_TRANSCRIPTION |  | 22 | 0.33 | 1.20 | 0.193 | 0.711 | 1.000 | 2813 | tags=45%, list=23%, signal=59% |
| 591 | GOCC\_RNA\_POLYMERASE\_III\_COMPLEX |  | 14 | 0.37 | 1.20 | 0.216 | 0.714 | 1.000 | 2334 | tags=50%, list=19%, signal=62% |
| 592 | GOBP\_L\_ALANINE\_TRANSPORT |  | 5 | 0.54 | 1.20 | 0.226 | 0.715 | 1.000 | 1708 | tags=60%, list=14%, signal=70% |
| 593 | GOBP\_ENDOCRINE\_PANCREAS\_DEVELOPMENT |  | 31 | 0.30 | 1.19 | 0.188 | 0.716 | 1.000 | 1084 | tags=23%, list=9%, signal=25% |
| 594 | GOBP\_ANTIGEN\_PROCESSING\_AND\_PRESENTATION\_OF\_EXOGENOUS\_PEPTIDE\_ANTIGEN\_VIA\_MHC\_CLASS\_I |  | 54 | 0.27 | 1.19 | 0.137 | 0.717 | 1.000 | 2619 | tags=39%, list=22%, signal=50% |
| 595 | GOCC\_EXTRINSIC\_COMPONENT\_OF\_EXTERNAL\_SIDE\_OF\_PLASMA\_MEMBRANE |  | 5 | 0.54 | 1.19 | 0.234 | 0.719 | 1.000 | 267 | tags=40%, list=2%, signal=41% |
| 596 | GOBP\_PROSTANOID\_BIOSYNTHETIC\_PROCESS |  | 22 | 0.33 | 1.19 | 0.195 | 0.718 | 1.000 | 416 | tags=23%, list=3%, signal=23% |
| 597 | GOBP\_REGULATION\_OF\_PEPTIDYL\_CYSTEINE\_S\_NITROSYLATION |  | 8 | 0.45 | 1.19 | 0.245 | 0.718 | 1.000 | 1854 | tags=50%, list=15%, signal=59% |
| 598 | GOBP\_KETONE\_BIOSYNTHETIC\_PROCESS |  | 29 | 0.31 | 1.19 | 0.203 | 0.723 | 1.000 | 1629 | tags=34%, list=14%, signal=40% |
| 599 | GOBP\_AROMATIC\_AMINO\_ACID\_FAMILY\_CATABOLIC\_PROCESS |  | 11 | 0.41 | 1.19 | 0.216 | 0.722 | 1.000 | 586 | tags=27%, list=5%, signal=29% |
| 600 | GOCC\_RIBONUCLEASE\_MRP\_COMPLEX |  | 5 | 0.55 | 1.19 | 0.252 | 0.721 | 1.000 | 871 | tags=40%, list=7%, signal=43% |
| 601 | GOBP\_DETECTION\_OF\_TEMPERATURE\_STIMULUS\_INVOLVED\_IN\_SENSORY\_PERCEPTION |  | 10 | 0.42 | 1.19 | 0.219 | 0.721 | 1.000 | 1235 | tags=30%, list=10%, signal=33% |
| 602 | GOBP\_SULFATE\_TRANSPORT |  | 13 | 0.39 | 1.19 | 0.222 | 0.720 | 1.000 | 712 | tags=31%, list=6%, signal=33% |
| 603 | GOBP\_PROTEIN\_DENEDDYLATION |  | 9 | 0.44 | 1.19 | 0.247 | 0.719 | 1.000 | 6726 | tags=100%, list=56%, signal=227% |
| 604 | GOBP\_ASPARTATE\_FAMILY\_AMINO\_ACID\_METABOLIC\_PROCESS |  | 42 | 0.28 | 1.19 | 0.169 | 0.718 | 1.000 | 1192 | tags=24%, list=10%, signal=26% |
| 605 | GOBP\_BOX\_C\_D\_SNORNP\_ASSEMBLY |  | 6 | 0.50 | 1.19 | 0.239 | 0.721 | 1.000 | 566 | tags=17%, list=5%, signal=17% |
| 606 | GOBP\_REGULATION\_OF\_MITOCHONDRIAL\_ELECTRON\_TRANSPORT\_NADH\_TO\_UBIQUINONE |  | 4 | 0.59 | 1.19 | 0.260 | 0.720 | 1.000 | 863 | tags=25%, list=7%, signal=27% |
| 607 | GOMF\_SECONDARY\_ACTIVE\_SULFATE\_TRANSMEMBRANE\_TRANSPORTER\_ACTIVITY |  | 8 | 0.45 | 1.19 | 0.231 | 0.720 | 1.000 | 452 | tags=38%, list=4%, signal=39% |
| 608 | GOBP\_RESPONSE\_TO\_TESTOSTERONE |  | 27 | 0.31 | 1.19 | 0.198 | 0.720 | 1.000 | 166 | tags=11%, list=1%, signal=11% |
| 609 | GOBP\_NEGATIVE\_REGULATION\_OF\_SYNAPTIC\_TRANSMISSION\_GLUTAMATERGIC |  | 4 | 0.59 | 1.19 | 0.258 | 0.720 | 1.000 | 550 | tags=25%, list=5%, signal=26% |
| 610 | GOBP\_ANGIOTENSIN\_ACTIVATED\_SIGNALING\_PATHWAY |  | 8 | 0.45 | 1.19 | 0.243 | 0.719 | 1.000 | 14 | tags=13%, list=0%, signal=13% |
| 611 | GOBP\_FRUCTOSE\_6\_PHOSPHATE\_METABOLIC\_PROCESS |  | 9 | 0.44 | 1.18 | 0.229 | 0.721 | 1.000 | 702 | tags=33%, list=6%, signal=35% |
| 612 | GOBP\_CREATINE\_METABOLIC\_PROCESS |  | 4 | 0.59 | 1.18 | 0.258 | 0.720 | 1.000 | 2789 | tags=75%, list=23%, signal=98% |
| 613 | GOCC\_STRIATED\_MUSCLE\_THIN\_FILAMENT |  | 12 | 0.40 | 1.18 | 0.227 | 0.719 | 1.000 | 2565 | tags=42%, list=21%, signal=53% |
| 614 | GOMF\_L\_ALANINE\_TRANSMEMBRANE\_TRANSPORTER\_ACTIVITY |  | 5 | 0.54 | 1.18 | 0.255 | 0.718 | 1.000 | 1708 | tags=60%, list=14%, signal=70% |
| 615 | GOBP\_POSITIVE\_REGULATION\_OF\_COMPLEMENT\_ACTIVATION |  | 4 | 0.58 | 1.18 | 0.254 | 0.719 | 1.000 | 5036 | tags=100%, list=42%, signal=172% |
| 616 | GOMF\_5S\_RRNA\_BINDING |  | 10 | 0.42 | 1.18 | 0.246 | 0.719 | 1.000 | 632 | tags=20%, list=5%, signal=21% |
| 617 | GOMF\_LIGASE\_ACTIVITY\_FORMING\_CARBON\_OXYGEN\_BONDS |  | 20 | 0.34 | 1.18 | 0.214 | 0.720 | 1.000 | 6540 | tags=95%, list=54%, signal=208% |
| 618 | GOBP\_SNO\_S\_RNA\_3\_END\_PROCESSING |  | 8 | 0.45 | 1.18 | 0.242 | 0.722 | 1.000 | 730 | tags=25%, list=6%, signal=27% |
| 619 | GOMF\_ALCOHOL\_DEHYDROGENASE\_NADPPLUS\_ACTIVITY |  | 12 | 0.40 | 1.18 | 0.236 | 0.724 | 1.000 | 1561 | tags=33%, list=13%, signal=38% |
| 620 | GOBP\_EXTRACELLULAR\_TRANSPORT |  | 20 | 0.34 | 1.18 | 0.213 | 0.725 | 1.000 | 1143 | tags=30%, list=10%, signal=33% |
| 621 | GOBP\_POSITIVE\_REGULATION\_OF\_FATTY\_ACID\_OXIDATION |  | 12 | 0.39 | 1.18 | 0.235 | 0.723 | 1.000 | 339 | tags=25%, list=3%, signal=26% |
| 622 | GOMF\_MEMBRANE\_INSERTASE\_ACTIVITY |  | 8 | 0.45 | 1.18 | 0.244 | 0.726 | 1.000 | 6592 | tags=100%, list=55%, signal=221% |
| 623 | GOBP\_MATURATION\_OF\_SSU\_RRNA\_FROM\_TRICISTRONIC\_RRNA\_TRANSCRIPT\_SSU\_RRNA\_5\_8S\_RRNA\_LSU\_RRNA |  | 27 | 0.31 | 1.18 | 0.203 | 0.725 | 1.000 | 2831 | tags=44%, list=24%, signal=58% |
| 624 | GOBP\_RIBOSOME\_BIOGENESIS |  | 243 | 0.21 | 1.18 | 0.038 | 0.725 | 1.000 | 2425 | tags=29%, list=20%, signal=35% |
| 625 | GOBP\_PROSTANOID\_METABOLIC\_PROCESS |  | 29 | 0.31 | 1.18 | 0.193 | 0.724 | 1.000 | 486 | tags=21%, list=4%, signal=22% |
| 626 | GOMF\_SUGAR\_TRANSMEMBRANE\_TRANSPORTER\_ACTIVITY |  | 17 | 0.35 | 1.18 | 0.220 | 0.725 | 1.000 | 633 | tags=29%, list=5%, signal=31% |
| 627 | GOBP\_POSITIVE\_REGULATION\_OF\_CELLULAR\_PH\_REDUCTION |  | 4 | 0.59 | 1.18 | 0.275 | 0.725 | 1.000 | 179 | tags=50%, list=1%, signal=51% |
| 628 | GOBP\_PROSTATE\_GLAND\_MORPHOGENESIS |  | 21 | 0.33 | 1.18 | 0.210 | 0.725 | 1.000 | 488 | tags=19%, list=4%, signal=20% |
| 629 | GOBP\_MEIOTIC\_CYTOKINESIS |  | 4 | 0.58 | 1.17 | 0.261 | 0.727 | 1.000 | 1211 | tags=25%, list=10%, signal=28% |
| 630 | GOBP\_CRISTAE\_FORMATION |  | 16 | 0.36 | 1.17 | 0.221 | 0.726 | 1.000 | 2298 | tags=50%, list=19%, signal=62% |
| 631 | GOBP\_CELLULAR\_AMINO\_ACID\_BIOSYNTHETIC\_PROCESS |  | 49 | 0.27 | 1.17 | 0.166 | 0.726 | 1.000 | 1955 | tags=31%, list=16%, signal=36% |
| 632 | GOCC\_ARP2\_3\_PROTEIN\_COMPLEX |  | 9 | 0.43 | 1.17 | 0.246 | 0.725 | 1.000 | 2546 | tags=44%, list=21%, signal=56% |
| 633 | GOBP\_TONIC\_SMOOTH\_MUSCLE\_CONTRACTION |  | 10 | 0.42 | 1.17 | 0.249 | 0.725 | 1.000 | 7 | tags=10%, list=0%, signal=10% |
| 634 | GOBP\_REGULATION\_OF\_GASTRIC\_ACID\_SECRETION |  | 7 | 0.48 | 1.17 | 0.255 | 0.726 | 1.000 | 1234 | tags=71%, list=10%, signal=80% |
| 635 | GOBP\_LOW\_DENSITY\_LIPOPROTEIN\_PARTICLE\_REMODELING |  | 7 | 0.46 | 1.17 | 0.236 | 0.728 | 1.000 | 2107 | tags=57%, list=18%, signal=69% |
| 636 | GOBP\_REGULATION\_OF\_VIRAL\_LIFE\_CYCLE |  | 80 | 0.24 | 1.17 | 0.121 | 0.729 | 1.000 | 758 | tags=15%, list=6%, signal=16% |
| 637 | GOBP\_ESCRT\_COMPLEX\_DISASSEMBLY |  | 10 | 0.41 | 1.17 | 0.248 | 0.728 | 1.000 | 2888 | tags=70%, list=24%, signal=92% |
| 638 | GOBP\_POSITIVE\_REGULATION\_OF\_UNSATURATED\_FATTY\_ACID\_BIOSYNTHETIC\_PROCESS |  | 6 | 0.50 | 1.17 | 0.249 | 0.729 | 1.000 | 1237 | tags=50%, list=10%, signal=56% |
| 639 | GOBP\_REGULATION\_OF\_WNT\_SIGNALING\_PATHWAY\_INVOLVED\_IN\_HEART\_DEVELOPMENT |  | 4 | 0.58 | 1.17 | 0.272 | 0.730 | 1.000 | 1104 | tags=50%, list=9%, signal=55% |
| 640 | GOBP\_POSITIVE\_REGULATION\_OF\_OLIGODENDROCYTE\_DIFFERENTIATION |  | 18 | 0.34 | 1.17 | 0.231 | 0.729 | 1.000 | 1049 | tags=28%, list=9%, signal=30% |
| 641 | GOBP\_REGULATION\_OF\_T\_HELPER\_2\_CELL\_CYTOKINE\_PRODUCTION |  | 7 | 0.48 | 1.17 | 0.255 | 0.728 | 1.000 | 799 | tags=43%, list=7%, signal=46% |
| 642 | GOBP\_UBIQUINONE\_METABOLIC\_PROCESS |  | 16 | 0.35 | 1.17 | 0.241 | 0.731 | 1.000 | 1629 | tags=31%, list=14%, signal=36% |
| 643 | GOMF\_OXIDOREDUCTASE\_ACTIVITY\_ACTING\_ON\_A\_SULFUR\_GROUP\_OF\_DONORS |  | 47 | 0.27 | 1.17 | 0.179 | 0.730 | 1.000 | 2191 | tags=32%, list=18%, signal=39% |
| 644 | GOBP\_SULFUR\_COMPOUND\_BIOSYNTHETIC\_PROCESS |  | 148 | 0.22 | 1.17 | 0.101 | 0.730 | 1.000 | 1547 | tags=22%, list=13%, signal=25% |
| 645 | GOBP\_MITOCHONDRIAL\_TRANSPORT |  | 185 | 0.21 | 1.17 | 0.075 | 0.730 | 1.000 | 1455 | tags=19%, list=12%, signal=21% |
| 646 | GOBP\_NEPHRON\_TUBULE\_FORMATION |  | 5 | 0.53 | 1.17 | 0.263 | 0.729 | 1.000 | 1490 | tags=40%, list=12%, signal=46% |
| 647 | GOBP\_REGULATION\_OF\_BIOLOGICAL\_PROCESS\_INVOLVED\_IN\_SYMBIOTIC\_INTERACTION |  | 116 | 0.23 | 1.17 | 0.111 | 0.729 | 1.000 | 1038 | tags=16%, list=9%, signal=17% |
| 648 | GOBP\_CELLULAR\_RESPONSE\_TO\_ACIDIC\_PH |  | 9 | 0.43 | 1.17 | 0.230 | 0.728 | 1.000 | 1292 | tags=33%, list=11%, signal=37% |
| 649 | GOBP\_NUCLEOSIDE\_TRIPHOSPHATE\_METABOLIC\_PROCESS |  | 62 | 0.26 | 1.17 | 0.169 | 0.727 | 1.000 | 1267 | tags=19%, list=11%, signal=22% |
| 650 | GOBP\_NEGATIVE\_REGULATION\_OF\_INTRINSIC\_APOPTOTIC\_SIGNALING\_PATHWAY |  | 66 | 0.25 | 1.17 | 0.154 | 0.727 | 1.000 | 2060 | tags=33%, list=17%, signal=40% |
| 651 | GOBP\_CITRULLINE\_METABOLIC\_PROCESS |  | 6 | 0.50 | 1.16 | 0.252 | 0.726 | 1.000 | 1515 | tags=33%, list=13%, signal=38% |
| 652 | GOCC\_PTW\_PP1\_PHOSPHATASE\_COMPLEX |  | 5 | 0.53 | 1.16 | 0.261 | 0.732 | 1.000 | 5629 | tags=100%, list=47%, signal=188% |
| 653 | GOBP\_SULFUR\_COMPOUND\_METABOLIC\_PROCESS |  | 271 | 0.20 | 1.16 | 0.088 | 0.743 | 1.000 | 1578 | tags=21%, list=13%, signal=23% |
| 654 | GOCC\_INTRACILIARY\_TRANSPORT\_PARTICLE\_B |  | 17 | 0.35 | 1.16 | 0.245 | 0.743 | 1.000 | 1782 | tags=24%, list=15%, signal=28% |
| 655 | GOBP\_NEGATIVE\_REGULATION\_OF\_TRANSFORMING\_GROWTH\_FACTOR\_BETA\_PRODUCTION |  | 10 | 0.41 | 1.16 | 0.260 | 0.746 | 1.000 | 1340 | tags=30%, list=11%, signal=34% |
| 656 | GOBP\_MONOACYLGLYCEROL\_CATABOLIC\_PROCESS |  | 5 | 0.53 | 1.15 | 0.274 | 0.746 | 1.000 | 703 | tags=40%, list=6%, signal=42% |
| 657 | GOBP\_REGULATION\_OF\_ADIPONECTIN\_SECRETION |  | 4 | 0.58 | 1.15 | 0.287 | 0.746 | 1.000 | 2561 | tags=75%, list=21%, signal=95% |
| 658 | GOBP\_NEGATIVE\_REGULATION\_OF\_NUCLEOTIDE\_METABOLIC\_PROCESS |  | 13 | 0.38 | 1.15 | 0.259 | 0.750 | 1.000 | 339 | tags=15%, list=3%, signal=16% |
| 659 | GOBP\_RESPONSE\_TO\_ELECTRICAL\_STIMULUS |  | 24 | 0.32 | 1.15 | 0.231 | 0.750 | 1.000 | 403 | tags=17%, list=3%, signal=17% |
| 660 | GOBP\_UNSATURATED\_FATTY\_ACID\_BIOSYNTHETIC\_PROCESS |  | 33 | 0.29 | 1.15 | 0.236 | 0.749 | 1.000 | 1335 | tags=30%, list=11%, signal=34% |
| 661 | GOBP\_IMMATURE\_T\_CELL\_PROLIFERATION\_IN\_THYMUS |  | 6 | 0.49 | 1.15 | 0.271 | 0.750 | 1.000 | 1723 | tags=50%, list=14%, signal=58% |
| 662 | GOBP\_PYRIMIDINE\_NUCLEOTIDE\_BIOSYNTHETIC\_PROCESS |  | 23 | 0.31 | 1.15 | 0.240 | 0.751 | 1.000 | 1965 | tags=22%, list=16%, signal=26% |
| 663 | GOBP\_VIRAL\_BUDDING |  | 24 | 0.32 | 1.15 | 0.236 | 0.750 | 1.000 | 3080 | tags=63%, list=26%, signal=84% |
| 664 | GOBP\_ENDOSOME\_TO\_PLASMA\_MEMBRANE\_PROTEIN\_TRANSPORT |  | 10 | 0.41 | 1.15 | 0.262 | 0.750 | 1.000 | 2591 | tags=50%, list=22%, signal=64% |
| 665 | GOBP\_NEGATIVE\_REGULATION\_OF\_ENDOTHELIAL\_CELL\_CHEMOTAXIS |  | 3 | 0.64 | 1.15 | 0.298 | 0.750 | 1.000 | 126 | tags=33%, list=1%, signal=34% |
| 666 | GOBP\_MITOCHONDRIAL\_RESPIRATORY\_CHAIN\_COMPLEX\_III\_ASSEMBLY |  | 7 | 0.46 | 1.15 | 0.272 | 0.750 | 1.000 | 2548 | tags=57%, list=21%, signal=72% |
| 667 | GOBP\_EMBRYONIC\_SKELETAL\_JOINT\_DEVELOPMENT |  | 11 | 0.39 | 1.15 | 0.266 | 0.751 | 1.000 | 2011 | tags=55%, list=17%, signal=65% |
| 668 | GOBP\_DOLICHOL\_METABOLIC\_PROCESS |  | 5 | 0.52 | 1.15 | 0.275 | 0.752 | 1.000 | 2774 | tags=60%, list=23%, signal=78% |
| 669 | GOBP\_AXONEME\_ASSEMBLY |  | 29 | 0.30 | 1.15 | 0.238 | 0.752 | 1.000 | 1660 | tags=24%, list=14%, signal=28% |
| 670 | GOBP\_ACTIVATION\_OF\_CYSTEINE\_TYPE\_ENDOPEPTIDASE\_ACTIVITY\_INVOLVED\_IN\_APOPTOTIC\_PROCESS\_BY\_CYTOCHROME\_C |  | 6 | 0.49 | 1.15 | 0.273 | 0.751 | 1.000 | 2022 | tags=50%, list=17%, signal=60% |
| 671 | GOBP\_POSITIVE\_REGULATION\_OF\_INTRACELLULAR\_STEROID\_HORMONE\_RECEPTOR\_SIGNALING\_PATHWAY |  | 9 | 0.42 | 1.15 | 0.278 | 0.750 | 1.000 | 766 | tags=33%, list=6%, signal=36% |
| 672 | GOBP\_NEGATIVE\_REGULATION\_OF\_VIRAL\_TRANSCRIPTION |  | 7 | 0.46 | 1.15 | 0.275 | 0.750 | 1.000 | 660 | tags=29%, list=5%, signal=30% |
| 673 | GOBP\_ORGANIC\_ACID\_CATABOLIC\_PROCESS |  | 192 | 0.21 | 1.15 | 0.088 | 0.749 | 1.000 | 1530 | tags=21%, list=13%, signal=24% |
| 674 | GOBP\_CELLULAR\_RESPONSE\_TO\_INCREASED\_OXYGEN\_LEVELS |  | 13 | 0.37 | 1.15 | 0.263 | 0.750 | 1.000 | 83 | tags=15%, list=1%, signal=15% |
| 675 | GOMF\_SMALL\_PROTEIN\_ACTIVATING\_ENZYME\_BINDING |  | 5 | 0.52 | 1.15 | 0.285 | 0.750 | 1.000 | 5767 | tags=100%, list=48%, signal=192% |
| 676 | GOBP\_TAIL\_ANCHORED\_MEMBRANE\_PROTEIN\_INSERTION\_INTO\_ER\_MEMBRANE |  | 9 | 0.42 | 1.15 | 0.269 | 0.749 | 1.000 | 6976 | tags=100%, list=58%, signal=238% |
| 677 | GOBP\_PEPTIDYL\_HISTIDINE\_MODIFICATION |  | 11 | 0.39 | 1.15 | 0.262 | 0.749 | 1.000 | 2035 | tags=36%, list=17%, signal=44% |
| 678 | GOBP\_CELLULAR\_RESPONSE\_TO\_ESTROGEN\_STIMULUS |  | 11 | 0.39 | 1.14 | 0.272 | 0.750 | 1.000 | 766 | tags=27%, list=6%, signal=29% |
| 679 | GOBP\_TRANSDIFFERENTIATION |  | 5 | 0.53 | 1.14 | 0.292 | 0.750 | 1.000 | 673 | tags=40%, list=6%, signal=42% |
| 680 | GOMF\_CARBOXYLIC\_ESTER\_HYDROLASE\_ACTIVITY |  | 82 | 0.24 | 1.14 | 0.187 | 0.749 | 1.000 | 1047 | tags=17%, list=9%, signal=19% |
| 681 | GOBP\_POSTSYNAPTIC\_NEUROTRANSMITTER\_RECEPTOR\_INTERNALIZATION |  | 10 | 0.40 | 1.14 | 0.281 | 0.748 | 1.000 | 16 | tags=10%, list=0%, signal=10% |
| 682 | GOMF\_CYTOKINE\_ACTIVITY |  | 81 | 0.24 | 1.14 | 0.175 | 0.748 | 1.000 | 1237 | tags=21%, list=10%, signal=23% |
| 683 | GOBP\_GROWTH\_INVOLVED\_IN\_HEART\_MORPHOGENESIS |  | 3 | 0.62 | 1.14 | 0.300 | 0.749 | 1.000 | 4525 | tags=100%, list=38%, signal=160% |
| 684 | GOBP\_ENDONUCLEOLYTIC\_CLEAVAGE\_IN\_ITS1\_TO\_SEPARATE\_SSU\_RRNA\_FROM\_5\_8S\_RRNA\_AND\_LSU\_RRNA\_FROM\_TRICISTRONIC\_RRNA\_TRANSCRIPT\_SSU\_RRNA\_5\_8S\_RRNA\_LSU\_RRNA |  | 6 | 0.49 | 1.14 | 0.280 | 0.748 | 1.000 | 790 | tags=33%, list=7%, signal=36% |
| 685 | GOBP\_NEGATIVE\_REGULATION\_OF\_HEMATOPOIETIC\_PROGENITOR\_CELL\_DIFFERENTIATION |  | 6 | 0.48 | 1.14 | 0.280 | 0.748 | 1.000 | 2493 | tags=50%, list=21%, signal=63% |
| 686 | GOMF\_RECEPTOR\_SERINE\_THREONINE\_KINASE\_BINDING |  | 16 | 0.35 | 1.14 | 0.260 | 0.749 | 1.000 | 134 | tags=13%, list=1%, signal=13% |
| 687 | GOBP\_MONOVALENT\_INORGANIC\_ANION\_HOMEOSTASIS |  | 9 | 0.42 | 1.14 | 0.274 | 0.748 | 1.000 | 1482 | tags=44%, list=12%, signal=51% |
| 688 | GOBP\_ARACHIDONIC\_ACID\_SECRETION |  | 14 | 0.36 | 1.14 | 0.250 | 0.749 | 1.000 | 432 | tags=29%, list=4%, signal=30% |
| 689 | GOMF\_AMIDE\_TRANSMEMBRANE\_TRANSPORTER\_ACTIVITY |  | 38 | 0.27 | 1.14 | 0.217 | 0.749 | 1.000 | 2235 | tags=37%, list=19%, signal=45% |
| 690 | GOBP\_RESPONSE\_TO\_AMPHETAMINE |  | 21 | 0.32 | 1.14 | 0.260 | 0.748 | 1.000 | 1090 | tags=24%, list=9%, signal=26% |
| 691 | GOBP\_PHOSPHATIDYLSERINE\_METABOLIC\_PROCESS |  | 24 | 0.31 | 1.14 | 0.250 | 0.748 | 1.000 | 738 | tags=25%, list=6%, signal=27% |
| 692 | GOMF\_PROSTANOID\_RECEPTOR\_ACTIVITY |  | 7 | 0.46 | 1.14 | 0.279 | 0.749 | 1.000 | 1234 | tags=29%, list=10%, signal=32% |
| 693 | GOCC\_EMC\_COMPLEX |  | 7 | 0.45 | 1.14 | 0.279 | 0.748 | 1.000 | 6592 | tags=100%, list=55%, signal=222% |
| 694 | GOBP\_PERICYTE\_CELL\_DIFFERENTIATION |  | 7 | 0.46 | 1.14 | 0.287 | 0.750 | 1.000 | 1490 | tags=29%, list=12%, signal=33% |
| 695 | GOBP\_RRNA\_METABOLIC\_PROCESS |  | 187 | 0.21 | 1.14 | 0.131 | 0.750 | 1.000 | 2425 | tags=30%, list=20%, signal=38% |
| 696 | GOMF\_THIOLESTER\_HYDROLASE\_ACTIVITY |  | 32 | 0.28 | 1.14 | 0.257 | 0.751 | 1.000 | 2348 | tags=38%, list=20%, signal=46% |
| 697 | GOMF\_PROTON\_CHANNEL\_ACTIVITY |  | 7 | 0.45 | 1.14 | 0.281 | 0.750 | 1.000 | 1267 | tags=43%, list=11%, signal=48% |
| 698 | GOBP\_PROTEIN\_OXIDATION |  | 13 | 0.37 | 1.14 | 0.278 | 0.751 | 1.000 | 2045 | tags=31%, list=17%, signal=37% |
| 699 | GOBP\_REGULATION\_OF\_VIRAL\_GENOME\_REPLICATION |  | 48 | 0.26 | 1.14 | 0.234 | 0.751 | 1.000 | 340 | tags=13%, list=3%, signal=13% |
| 700 | GOBP\_DEOXYRIBONUCLEOSIDE\_TRIPHOSPHATE\_METABOLIC\_PROCESS |  | 12 | 0.37 | 1.13 | 0.285 | 0.751 | 1.000 | 238 | tags=17%, list=2%, signal=17% |
| 701 | GOBP\_CELLULAR\_MODIFIED\_AMINO\_ACID\_METABOLIC\_PROCESS |  | 126 | 0.22 | 1.13 | 0.143 | 0.750 | 1.000 | 738 | tags=14%, list=6%, signal=15% |
| 702 | GOBP\_NEGATIVE\_REGULATION\_OF\_OXIDATIVE\_PHOSPHORYLATION |  | 5 | 0.52 | 1.13 | 0.292 | 0.750 | 1.000 | 1060 | tags=40%, list=9%, signal=44% |
| 703 | GOBP\_RESPONSE\_TO\_POTASSIUM\_ION |  | 5 | 0.52 | 1.13 | 0.304 | 0.749 | 1.000 | 1482 | tags=40%, list=12%, signal=46% |
| 704 | GOMF\_AMINE\_BINDING |  | 6 | 0.48 | 1.13 | 0.285 | 0.749 | 1.000 | 1807 | tags=50%, list=15%, signal=59% |
| 705 | GOBP\_RESPONSE\_TO\_PH |  | 30 | 0.29 | 1.13 | 0.251 | 0.751 | 1.000 | 1292 | tags=27%, list=11%, signal=30% |
| 706 | GOMF\_RRNA\_CYTOSINE\_METHYLTRANSFERASE\_ACTIVITY |  | 4 | 0.56 | 1.13 | 0.300 | 0.750 | 1.000 | 1903 | tags=50%, list=16%, signal=59% |
| 707 | GOBP\_PROTEIN\_NITROSYLATION |  | 11 | 0.39 | 1.13 | 0.283 | 0.752 | 1.000 | 2178 | tags=55%, list=18%, signal=67% |
| 708 | GOBP\_GLUCOSE\_6\_PHOSPHATE\_METABOLIC\_PROCESS |  | 20 | 0.32 | 1.13 | 0.258 | 0.752 | 1.000 | 1251 | tags=35%, list=10%, signal=39% |
| 709 | GOCC\_F\_ACTIN\_CAPPING\_PROTEIN\_COMPLEX |  | 7 | 0.45 | 1.13 | 0.286 | 0.752 | 1.000 | 6554 | tags=100%, list=55%, signal=220% |
| 710 | GOBP\_NEGATIVE\_REGULATION\_OF\_SMOOTH\_MUSCLE\_CELL\_PROLIFERATION |  | 30 | 0.29 | 1.13 | 0.246 | 0.752 | 1.000 | 843 | tags=20%, list=7%, signal=21% |
| 711 | GOBP\_NEGATIVE\_REGULATION\_OF\_HORMONE\_SECRETION |  | 35 | 0.28 | 1.13 | 0.269 | 0.754 | 1.000 | 1569 | tags=26%, list=13%, signal=29% |
| 712 | GOBP\_RESPONSE\_TO\_WATER\_DEPRIVATION |  | 3 | 0.63 | 1.13 | 0.315 | 0.753 | 1.000 | 535 | tags=67%, list=4%, signal=70% |
| 713 | GOBP\_POSITIVE\_REGULATION\_OF\_NEUROTROPHIN\_TRK\_RECEPTOR\_SIGNALING\_PATHWAY |  | 5 | 0.51 | 1.13 | 0.307 | 0.752 | 1.000 | 2467 | tags=80%, list=21%, signal=101% |
| 714 | GOBP\_POSITIVE\_REGULATION\_OF\_APOPTOTIC\_PROCESS\_INVOLVED\_IN\_DEVELOPMENT |  | 3 | 0.62 | 1.13 | 0.321 | 0.753 | 1.000 | 4525 | tags=100%, list=38%, signal=160% |
| 715 | GOBP\_EPIDERMIS\_MORPHOGENESIS |  | 17 | 0.34 | 1.13 | 0.292 | 0.754 | 1.000 | 2059 | tags=35%, list=17%, signal=43% |
| 716 | GOMF\_HEXOKINASE\_ACTIVITY |  | 5 | 0.52 | 1.13 | 0.310 | 0.753 | 1.000 | 361 | tags=40%, list=3%, signal=41% |
| 717 | GOBP\_CELLULAR\_AMINO\_ACID\_METABOLIC\_PROCESS |  | 229 | 0.20 | 1.13 | 0.065 | 0.754 | 1.000 | 1955 | tags=23%, list=16%, signal=27% |
| 718 | GOBP\_POSITIVE\_REGULATION\_OF\_PROTEIN\_CATABOLIC\_PROCESS\_IN\_THE\_VACUOLE |  | 5 | 0.52 | 1.13 | 0.310 | 0.755 | 1.000 | 1499 | tags=40%, list=12%, signal=46% |
| 719 | GOBP\_REGULATION\_OF\_INTEGRIN\_BIOSYNTHETIC\_PROCESS |  | 4 | 0.56 | 1.13 | 0.313 | 0.755 | 1.000 | 2089 | tags=75%, list=17%, signal=91% |
| 720 | GOBP\_REGULATION\_OF\_PHOSPHATIDYLINOSITOL\_BIOSYNTHETIC\_PROCESS |  | 4 | 0.57 | 1.13 | 0.323 | 0.755 | 1.000 | 5197 | tags=100%, list=43%, signal=176% |
| 721 | GOBP\_PROTEIN\_INSERTION\_INTO\_ER\_MEMBRANE\_BY\_STOP\_TRANSFER\_MEMBRANE\_ANCHOR\_SEQUENCE |  | 7 | 0.45 | 1.12 | 0.294 | 0.755 | 1.000 | 6592 | tags=100%, list=55%, signal=222% |
| 722 | GOBP\_RIBOSOMAL\_SMALL\_SUBUNIT\_BIOGENESIS |  | 59 | 0.25 | 1.12 | 0.236 | 0.754 | 1.000 | 2413 | tags=32%, list=20%, signal=40% |
| 723 | GOBP\_NEGATIVE\_REGULATION\_OF\_FATTY\_ACID\_BETA\_OXIDATION |  | 5 | 0.52 | 1.12 | 0.303 | 0.756 | 1.000 | 50 | tags=20%, list=0%, signal=20% |
| 724 | GOBP\_MITOCHONDRIAL\_TRNA\_METHYLATION |  | 4 | 0.55 | 1.12 | 0.307 | 0.757 | 1.000 | 5389 | tags=100%, list=45%, signal=181% |
| 725 | GOBP\_SHORT\_CHAIN\_FATTY\_ACID\_METABOLIC\_PROCESS |  | 13 | 0.36 | 1.12 | 0.287 | 0.757 | 1.000 | 1216 | tags=31%, list=10%, signal=34% |
| 726 | GOBP\_SERINE\_FAMILY\_AMINO\_ACID\_BIOSYNTHETIC\_PROCESS |  | 11 | 0.39 | 1.12 | 0.291 | 0.756 | 1.000 | 1955 | tags=45%, list=16%, signal=54% |
| 727 | GOBP\_NUCLEOSIDE\_BISPHOSPHATE\_METABOLIC\_PROCESS |  | 100 | 0.22 | 1.12 | 0.175 | 0.755 | 1.000 | 1578 | tags=24%, list=13%, signal=27% |
| 728 | GOBP\_NEGATIVE\_REGULATION\_OF\_PEPTIDYL\_CYSTEINE\_S\_NITROSYLATION |  | 5 | 0.51 | 1.12 | 0.306 | 0.754 | 1.000 | 2934 | tags=60%, list=24%, signal=79% |
| 729 | GOCC\_LSM1\_7\_PAT1\_COMPLEX |  | 4 | 0.56 | 1.12 | 0.298 | 0.754 | 1.000 | 5284 | tags=100%, list=44%, signal=178% |
| 730 | GOBP\_POSITIVE\_REGULATION\_OF\_THE\_FORCE\_OF\_HEART\_CONTRACTION |  | 4 | 0.56 | 1.12 | 0.310 | 0.753 | 1.000 | 79 | tags=25%, list=1%, signal=25% |
| 731 | GOBP\_REGULATION\_OF\_MEMBRANE\_PERMEABILITY |  | 58 | 0.25 | 1.12 | 0.257 | 0.752 | 1.000 | 1244 | tags=21%, list=10%, signal=23% |
| 732 | GOBP\_NEGATIVE\_REGULATION\_OF\_MITOCHONDRION\_ORGANIZATION |  | 34 | 0.28 | 1.12 | 0.262 | 0.753 | 1.000 | 1146 | tags=24%, list=10%, signal=26% |
| 733 | GOBP\_NEGATIVE\_REGULATION\_OF\_CLATHRIN\_DEPENDENT\_ENDOCYTOSIS |  | 4 | 0.56 | 1.12 | 0.307 | 0.752 | 1.000 | 5302 | tags=100%, list=44%, signal=179% |
| 734 | GOBP\_MICROGLIA\_DIFFERENTIATION |  | 4 | 0.56 | 1.12 | 0.303 | 0.751 | 1.000 | 5297 | tags=100%, list=44%, signal=179% |
| 735 | GOBP\_DISACCHARIDE\_BIOSYNTHETIC\_PROCESS |  | 3 | 0.62 | 1.12 | 0.330 | 0.751 | 1.000 | 178 | tags=33%, list=1%, signal=34% |
| 736 | GOMF\_N\_ACETYLLACTOSAMINE\_SYNTHASE\_ACTIVITY |  | 5 | 0.51 | 1.12 | 0.308 | 0.753 | 1.000 | 827 | tags=40%, list=7%, signal=43% |
| 737 | GOBP\_REGULATION\_OF\_KILLING\_OF\_CELLS\_OF\_OTHER\_ORGANISM |  | 8 | 0.43 | 1.12 | 0.297 | 0.752 | 1.000 | 593 | tags=38%, list=5%, signal=39% |
| 738 | GOBP\_RESPONSE\_TO\_ESTROGEN |  | 51 | 0.25 | 1.12 | 0.239 | 0.753 | 1.000 | 766 | tags=18%, list=6%, signal=19% |
| 739 | GOBP\_ETHANOL\_CATABOLIC\_PROCESS |  | 3 | 0.62 | 1.12 | 0.335 | 0.753 | 1.000 | 4510 | tags=100%, list=38%, signal=160% |
| 740 | GOBP\_CELLULAR\_RESPONSE\_TO\_OXYGEN\_RADICAL |  | 15 | 0.34 | 1.12 | 0.282 | 0.753 | 1.000 | 228 | tags=13%, list=2%, signal=14% |
| 741 | GOMF\_ANKYRIN\_BINDING |  | 14 | 0.35 | 1.12 | 0.300 | 0.752 | 1.000 | 946 | tags=21%, list=8%, signal=23% |
| 742 | GOBP\_HYALURONAN\_CATABOLIC\_PROCESS |  | 14 | 0.36 | 1.12 | 0.312 | 0.752 | 1.000 | 1292 | tags=29%, list=11%, signal=32% |
| 743 | GOBP\_ACUTE\_INFLAMMATORY\_RESPONSE |  | 53 | 0.25 | 1.12 | 0.225 | 0.751 | 1.000 | 1445 | tags=26%, list=12%, signal=30% |
| 744 | GOBP\_OXALOACETATE\_METABOLIC\_PROCESS |  | 9 | 0.40 | 1.12 | 0.296 | 0.752 | 1.000 | 980 | tags=33%, list=8%, signal=36% |
| 745 | GOMF\_INSULIN\_LIKE\_GROWTH\_FACTOR\_II\_BINDING |  | 7 | 0.45 | 1.12 | 0.295 | 0.752 | 1.000 | 454 | tags=29%, list=4%, signal=30% |
| 746 | GOBP\_NEGATIVE\_REGULATION\_OF\_SKELETAL\_MUSCLE\_CELL\_DIFFERENTIATION |  | 3 | 0.61 | 1.12 | 0.307 | 0.751 | 1.000 | 4685 | tags=100%, list=39%, signal=164% |
| 747 | GOMF\_FK506\_BINDING |  | 7 | 0.45 | 1.12 | 0.298 | 0.750 | 1.000 | 1128 | tags=29%, list=9%, signal=32% |
| 748 | GOBP\_QUINONE\_METABOLIC\_PROCESS |  | 25 | 0.30 | 1.12 | 0.265 | 0.751 | 1.000 | 1629 | tags=28%, list=14%, signal=32% |
| 749 | GOBP\_CATECHOLAMINE\_UPTAKE\_INVOLVED\_IN\_SYNAPTIC\_TRANSMISSION |  | 7 | 0.44 | 1.12 | 0.291 | 0.750 | 1.000 | 293 | tags=14%, list=2%, signal=15% |
| 750 | GOBP\_NEGATIVE\_REGULATION\_OF\_MOLECULAR\_FUNCTION\_IN\_OTHER\_ORGANISM |  | 3 | 0.62 | 1.12 | 0.328 | 0.750 | 1.000 | 514 | tags=33%, list=4%, signal=35% |
| 751 | GOMF\_3\_CHLOROALLYL\_ALDEHYDE\_DEHYDROGENASE\_ACTIVITY |  | 3 | 0.62 | 1.11 | 0.326 | 0.750 | 1.000 | 4510 | tags=100%, list=38%, signal=160% |
| 752 | GOBP\_NEUROTRANSMITTER\_RECEPTOR\_TRANSPORT\_ENDOSOME\_TO\_PLASMA\_MEMBRANE |  | 8 | 0.42 | 1.11 | 0.293 | 0.750 | 1.000 | 2591 | tags=63%, list=22%, signal=80% |
| 753 | GOCC\_CHAPERONE\_COMPLEX |  | 22 | 0.31 | 1.11 | 0.268 | 0.750 | 1.000 | 2629 | tags=32%, list=22%, signal=41% |
| 754 | GOBP\_REGULATION\_OF\_UNSATURATED\_FATTY\_ACID\_BIOSYNTHETIC\_PROCESS |  | 9 | 0.41 | 1.11 | 0.286 | 0.749 | 1.000 | 1238 | tags=44%, list=10%, signal=50% |
| 755 | GOBP\_APOPTOTIC\_MITOCHONDRIAL\_CHANGES |  | 76 | 0.23 | 1.11 | 0.218 | 0.749 | 1.000 | 1694 | tags=24%, list=14%, signal=27% |
| 756 | GOMF\_ADRENERGIC\_RECEPTOR\_ACTIVITY |  | 5 | 0.50 | 1.11 | 0.308 | 0.750 | 1.000 | 2740 | tags=60%, list=23%, signal=78% |
| 757 | GOBP\_AMINO\_ACID\_ACTIVATION |  | 27 | 0.29 | 1.11 | 0.278 | 0.750 | 1.000 | 3074 | tags=44%, list=26%, signal=60% |
| 758 | GOMF\_DISULFIDE\_OXIDOREDUCTASE\_ACTIVITY |  | 32 | 0.28 | 1.11 | 0.264 | 0.749 | 1.000 | 2191 | tags=34%, list=18%, signal=42% |
| 759 | GOBP\_RNA\_5\_END\_PROCESSING |  | 15 | 0.34 | 1.11 | 0.277 | 0.749 | 1.000 | 1517 | tags=27%, list=13%, signal=30% |
| 760 | GOBP\_ICOSANOID\_BIOSYNTHETIC\_PROCESS |  | 34 | 0.27 | 1.11 | 0.275 | 0.749 | 1.000 | 416 | tags=21%, list=3%, signal=21% |
| 761 | GOBP\_POSITIVE\_REGULATION\_OF\_INSULIN\_SECRETION\_INVOLVED\_IN\_CELLULAR\_RESPONSE\_TO\_GLUCOSE\_STIMULUS |  | 20 | 0.32 | 1.11 | 0.299 | 0.749 | 1.000 | 407 | tags=20%, list=3%, signal=21% |
| 762 | GOBP\_ANAPHASE\_PROMOTING\_COMPLEX\_DEPENDENT\_CATABOLIC\_PROCESS |  | 64 | 0.24 | 1.11 | 0.236 | 0.751 | 1.000 | 3006 | tags=36%, list=25%, signal=48% |
| 763 | GOBP\_POSITIVE\_REGULATION\_OF\_BLOOD\_CIRCULATION |  | 27 | 0.29 | 1.11 | 0.294 | 0.751 | 1.000 | 558 | tags=22%, list=5%, signal=23% |
| 764 | GOBP\_ENDOPLASMIC\_RETICULUM\_PLASMA\_MEMBRANE\_TETHERING |  | 4 | 0.55 | 1.11 | 0.319 | 0.751 | 1.000 | 304 | tags=25%, list=3%, signal=26% |
| 765 | GOBP\_POSITIVE\_REGULATION\_OF\_GASTRULATION |  | 4 | 0.55 | 1.11 | 0.327 | 0.751 | 1.000 | 5452 | tags=100%, list=45%, signal=183% |
| 766 | GOBP\_POSITIVE\_REGULATION\_OF\_RIG\_I\_SIGNALING\_PATHWAY |  | 6 | 0.47 | 1.11 | 0.311 | 0.754 | 1.000 | 332 | tags=33%, list=3%, signal=34% |
| 767 | GOBP\_OLIGOPEPTIDE\_TRANSPORT |  | 9 | 0.41 | 1.11 | 0.304 | 0.753 | 1.000 | 1442 | tags=33%, list=12%, signal=38% |
| 768 | GOBP\_SPERM\_AXONEME\_ASSEMBLY |  | 15 | 0.34 | 1.11 | 0.301 | 0.754 | 1.000 | 265 | tags=13%, list=2%, signal=14% |
| 769 | GOBP\_PROTEIN\_LOCALIZATION\_TO\_MITOCHONDRION |  | 108 | 0.22 | 1.11 | 0.227 | 0.754 | 1.000 | 2576 | tags=33%, list=21%, signal=42% |
| 770 | GOBP\_MITOCHONDRIAL\_RNA\_MODIFICATION |  | 8 | 0.42 | 1.11 | 0.316 | 0.756 | 1.000 | 6910 | tags=100%, list=58%, signal=235% |
| 771 | GOBP\_REGULATION\_OF\_MITOCHONDRION\_ORGANIZATION |  | 111 | 0.22 | 1.11 | 0.203 | 0.755 | 1.000 | 1211 | tags=17%, list=10%, signal=19% |
| 772 | GOBP\_REGULATION\_OF\_CATECHOLAMINE\_UPTAKE\_INVOLVED\_IN\_SYNAPTIC\_TRANSMISSION |  | 5 | 0.50 | 1.11 | 0.310 | 0.754 | 1.000 | 293 | tags=20%, list=2%, signal=20% |
| 773 | GOBP\_REGULATION\_OF\_TRANSCRIPTION\_BY\_GLUCOSE |  | 4 | 0.55 | 1.10 | 0.332 | 0.754 | 1.000 | 2090 | tags=50%, list=17%, signal=61% |
| 774 | GOMF\_ATPASE\_BINDING |  | 62 | 0.24 | 1.10 | 0.228 | 0.755 | 1.000 | 449 | tags=11%, list=4%, signal=12% |
| 775 | GOCC\_KERATIN\_FILAMENT |  | 9 | 0.41 | 1.10 | 0.315 | 0.757 | 1.000 | 1960 | tags=33%, list=16%, signal=40% |
| 776 | GOBP\_MITOTIC\_CYTOKINETIC\_PROCESS |  | 20 | 0.31 | 1.10 | 0.298 | 0.757 | 1.000 | 2585 | tags=50%, list=22%, signal=64% |
| 777 | GOBP\_REGULATION\_OF\_INSULIN\_LIKE\_GROWTH\_FACTOR\_RECEPTOR\_SIGNALING\_PATHWAY |  | 14 | 0.35 | 1.10 | 0.303 | 0.758 | 1.000 | 1130 | tags=29%, list=9%, signal=32% |
| 778 | GOBP\_REGULATION\_OF\_URINE\_VOLUME |  | 14 | 0.35 | 1.10 | 0.314 | 0.762 | 1.000 | 535 | tags=14%, list=4%, signal=15% |
| 779 | GOBP\_NEGATIVE\_REGULATION\_OF\_MYOBLAST\_DIFFERENTIATION |  | 19 | 0.32 | 1.10 | 0.314 | 0.761 | 1.000 | 309 | tags=16%, list=3%, signal=16% |
| 780 | GOBP\_POLYAMINE\_TRANSPORT |  | 6 | 0.46 | 1.10 | 0.329 | 0.761 | 1.000 | 1035 | tags=33%, list=9%, signal=36% |
| 781 | GOMF\_RECEPTOR\_INHIBITOR\_ACTIVITY |  | 12 | 0.36 | 1.10 | 0.316 | 0.762 | 1.000 | 259 | tags=25%, list=2%, signal=26% |
| 782 | GOBP\_OUTER\_DYNEIN\_ARM\_ASSEMBLY |  | 8 | 0.42 | 1.10 | 0.312 | 0.762 | 1.000 | 1143 | tags=25%, list=10%, signal=28% |
| 783 | GOMF\_ALPHA\_N\_ACETYLGALACTOSAMINIDE\_ALPHA\_2\_6\_SIALYLTRANSFERASE\_ACTIVITY |  | 6 | 0.47 | 1.10 | 0.333 | 0.762 | 1.000 | 1300 | tags=33%, list=11%, signal=37% |
| 784 | GOBP\_KILLING\_OF\_CELLS\_OF\_OTHER\_ORGANISM |  | 19 | 0.32 | 1.10 | 0.316 | 0.763 | 1.000 | 593 | tags=21%, list=5%, signal=22% |
| 785 | GOBP\_ENDOCARDIAL\_CELL\_DIFFERENTIATION |  | 5 | 0.50 | 1.10 | 0.314 | 0.763 | 1.000 | 3013 | tags=60%, list=25%, signal=80% |
| 786 | GOBP\_ENDOPLASMIC\_RETICULUM\_LOCALIZATION |  | 4 | 0.55 | 1.10 | 0.336 | 0.762 | 1.000 | 304 | tags=25%, list=3%, signal=26% |
| 787 | GOBP\_ASPARTATE\_TRANSMEMBRANE\_TRANSPORT |  | 7 | 0.44 | 1.10 | 0.328 | 0.761 | 1.000 | 812 | tags=29%, list=7%, signal=31% |
| 788 | GOBP\_POSITIVE\_REGULATION\_OF\_MEMBRANE\_PERMEABILITY |  | 45 | 0.26 | 1.10 | 0.272 | 0.761 | 1.000 | 1244 | tags=20%, list=10%, signal=22% |
| 789 | GOBP\_PENETRATION\_OF\_ZONA\_PELLUCIDA |  | 4 | 0.54 | 1.10 | 0.323 | 0.761 | 1.000 | 1270 | tags=50%, list=11%, signal=56% |
| 790 | GOBP\_POSITIVE\_REGULATION\_OF\_GLIAL\_CELL\_DIFFERENTIATION |  | 30 | 0.28 | 1.09 | 0.301 | 0.764 | 1.000 | 1084 | tags=23%, list=9%, signal=26% |
| 791 | GOBP\_SEPTUM\_PRIMUM\_DEVELOPMENT |  | 3 | 0.60 | 1.09 | 0.338 | 0.764 | 1.000 | 4779 | tags=100%, list=40%, signal=166% |
| 792 | GOCC\_ENDOCYTIC\_VESICLE\_MEMBRANE |  | 99 | 0.22 | 1.09 | 0.227 | 0.764 | 1.000 | 927 | tags=13%, list=8%, signal=14% |
| 793 | GOCC\_PHAGOCYTIC\_VESICLE |  | 101 | 0.22 | 1.09 | 0.240 | 0.763 | 1.000 | 1033 | tags=15%, list=9%, signal=16% |
| 794 | GOBP\_L\_GLUTAMATE\_IMPORT\_ACROSS\_PLASMA\_MEMBRANE |  | 10 | 0.38 | 1.09 | 0.323 | 0.766 | 1.000 | 432 | tags=20%, list=4%, signal=21% |
| 795 | GOBP\_TRNA\_THREONYLCARBAMOYLADENOSINE\_METABOLIC\_PROCESS |  | 11 | 0.37 | 1.09 | 0.317 | 0.765 | 1.000 | 7532 | tags=100%, list=63%, signal=268% |
| 796 | GOBP\_REGULATION\_OF\_TRANSCRIPTION\_FROM\_RNA\_POLYMERASE\_II\_PROMOTER\_IN\_RESPONSE\_TO\_HYPOXIA |  | 57 | 0.24 | 1.09 | 0.265 | 0.764 | 1.000 | 3006 | tags=40%, list=25%, signal=54% |
| 797 | GOBP\_BRANCHING\_INVOLVED\_IN\_PROSTATE\_GLAND\_MORPHOGENESIS |  | 10 | 0.39 | 1.09 | 0.312 | 0.764 | 1.000 | 488 | tags=20%, list=4%, signal=21% |
| 798 | GOBP\_PROTEIN\_TRANSMEMBRANE\_TRANSPORT |  | 47 | 0.25 | 1.09 | 0.271 | 0.765 | 1.000 | 2045 | tags=26%, list=17%, signal=31% |
| 799 | GOBP\_REGULATION\_OF\_RECEPTOR\_MEDIATED\_ENDOCYTOSIS\_INVOLVED\_IN\_CHOLESTEROL\_TRANSPORT |  | 3 | 0.60 | 1.09 | 0.348 | 0.765 | 1.000 | 158 | tags=33%, list=1%, signal=34% |
| 800 | GOBP\_HYDROCARBON\_METABOLIC\_PROCESS |  | 4 | 0.54 | 1.09 | 0.335 | 0.765 | 1.000 | 535 | tags=25%, list=4%, signal=26% |
| 801 | GOBP\_NEGATIVE\_REGULATION\_OF\_CHOLESTEROL\_STORAGE |  | 7 | 0.44 | 1.09 | 0.321 | 0.765 | 1.000 | 309 | tags=29%, list=3%, signal=29% |
| 802 | GOBP\_PLATELET\_ACTIVATING\_FACTOR\_METABOLIC\_PROCESS |  | 6 | 0.47 | 1.09 | 0.329 | 0.764 | 1.000 | 2107 | tags=50%, list=18%, signal=61% |
| 803 | GOBP\_CENTRAL\_NERVOUS\_SYSTEM\_MATURATION |  | 4 | 0.54 | 1.09 | 0.345 | 0.763 | 1.000 | 43 | tags=25%, list=0%, signal=25% |
| 804 | GOMF\_ARYLESTERASE\_ACTIVITY |  | 3 | 0.60 | 1.09 | 0.349 | 0.763 | 1.000 | 14 | tags=33%, list=0%, signal=33% |
| 805 | GOBP\_REGULATION\_OF\_RECEPTOR\_LOCALIZATION\_TO\_SYNAPSE |  | 12 | 0.36 | 1.09 | 0.327 | 0.762 | 1.000 | 7657 | tags=100%, list=64%, signal=276% |
| 806 | GOBP\_VESICLE\_FUSION\_WITH\_GOLGI\_APPARATUS |  | 8 | 0.41 | 1.09 | 0.326 | 0.762 | 1.000 | 7066 | tags=100%, list=59%, signal=243% |
| 807 | GOBP\_MAMMALIAN\_OOGENESIS\_STAGE |  | 5 | 0.49 | 1.09 | 0.333 | 0.762 | 1.000 | 33 | tags=20%, list=0%, signal=20% |
| 808 | GOMF\_NUCLEOSIDE\_MONOPHOSPHATE\_KINASE\_ACTIVITY |  | 14 | 0.34 | 1.09 | 0.327 | 0.765 | 1.000 | 1197 | tags=29%, list=10%, signal=32% |
| 809 | GOCC\_SARCOPLASMIC\_RETICULUM\_LUMEN |  | 4 | 0.54 | 1.09 | 0.344 | 0.766 | 1.000 | 144 | tags=25%, list=1%, signal=25% |
| 810 | GOBP\_POSITIVE\_REGULATION\_OF\_GONAD\_DEVELOPMENT |  | 5 | 0.50 | 1.09 | 0.343 | 0.765 | 1.000 | 6045 | tags=100%, list=50%, signal=201% |
| 811 | GOBP\_N\_ACETYLNEURAMINATE\_CATABOLIC\_PROCESS |  | 4 | 0.54 | 1.09 | 0.334 | 0.766 | 1.000 | 44 | tags=25%, list=0%, signal=25% |
| 812 | GOBP\_BMP\_SIGNALING\_PATHWAY\_INVOLVED\_IN\_HEART\_DEVELOPMENT |  | 4 | 0.54 | 1.08 | 0.330 | 0.773 | 1.000 | 1723 | tags=50%, list=14%, signal=58% |
| 813 | GOCC\_PRERIBOSOME\_LARGE\_SUBUNIT\_PRECURSOR |  | 21 | 0.31 | 1.08 | 0.304 | 0.773 | 1.000 | 2970 | tags=48%, list=25%, signal=63% |
| 814 | GOBP\_PENTOSE\_CATABOLIC\_PROCESS |  | 5 | 0.50 | 1.08 | 0.346 | 0.772 | 1.000 | 663 | tags=40%, list=6%, signal=42% |
| 815 | GOBP\_NEGATIVE\_REGULATION\_OF\_AMINE\_TRANSPORT |  | 16 | 0.33 | 1.08 | 0.323 | 0.772 | 1.000 | 804 | tags=25%, list=7%, signal=27% |
| 816 | GOBP\_OLIGOSACCHARIDE\_LIPID\_INTERMEDIATE\_BIOSYNTHETIC\_PROCESS |  | 17 | 0.32 | 1.08 | 0.336 | 0.774 | 1.000 | 2301 | tags=41%, list=19%, signal=51% |
| 817 | GOBP\_POSITIVE\_REGULATION\_OF\_ENDOTHELIAL\_CELL\_APOPTOTIC\_PROCESS |  | 12 | 0.36 | 1.08 | 0.328 | 0.774 | 1.000 | 1613 | tags=33%, list=13%, signal=38% |
| 818 | GOBP\_DENSE\_CORE\_GRANULE\_EXOCYTOSIS |  | 8 | 0.42 | 1.08 | 0.340 | 0.776 | 1.000 | 29 | tags=25%, list=0%, signal=25% |
| 819 | GOBP\_PYRIMIDINE\_RIBONUCLEOTIDE\_BIOSYNTHETIC\_PROCESS |  | 17 | 0.33 | 1.08 | 0.334 | 0.775 | 1.000 | 1965 | tags=24%, list=16%, signal=28% |
| 820 | GOMF\_FATTY\_ACID\_SYNTHASE\_ACTIVITY |  | 8 | 0.41 | 1.08 | 0.331 | 0.777 | 1.000 | 1518 | tags=38%, list=13%, signal=43% |
| 821 | GOBP\_REGULATION\_OF\_MRNA\_BINDING |  | 7 | 0.44 | 1.08 | 0.341 | 0.776 | 1.000 | 6769 | tags=100%, list=56%, signal=229% |
| 822 | GOBP\_NUCLEOSIDE\_DIPHOSPHATE\_CATABOLIC\_PROCESS |  | 10 | 0.38 | 1.08 | 0.343 | 0.776 | 1.000 | 1776 | tags=30%, list=15%, signal=35% |
| 823 | GOMF\_CATION\_CHLORIDE\_SYMPORTER\_ACTIVITY |  | 12 | 0.36 | 1.08 | 0.344 | 0.778 | 1.000 | 1482 | tags=42%, list=12%, signal=47% |
| 824 | GOBP\_OVULATION |  | 13 | 0.35 | 1.08 | 0.345 | 0.778 | 1.000 | 449 | tags=23%, list=4%, signal=24% |
| 825 | GOMF\_VASOPRESSIN\_RECEPTOR\_ACTIVITY |  | 4 | 0.54 | 1.08 | 0.345 | 0.778 | 1.000 | 1090 | tags=50%, list=9%, signal=55% |
| 826 | GOCC\_DYNACTIN\_COMPLEX |  | 10 | 0.38 | 1.08 | 0.346 | 0.777 | 1.000 | 7416 | tags=100%, list=62%, signal=261% |
| 827 | GOMF\_VOLTAGE\_GATED\_POTASSIUM\_CHANNEL\_ACTIVITY\_INVOLVED\_IN\_VENTRICULAR\_CARDIAC\_MUSCLE\_CELL\_ACTION\_POTENTIAL\_REPOLARIZATION |  | 6 | 0.46 | 1.08 | 0.350 | 0.777 | 1.000 | 2179 | tags=50%, list=18%, signal=61% |
| 828 | GOBP\_NEGATIVE\_REGULATION\_OF\_ORGANIC\_ACID\_TRANSPORT |  | 11 | 0.37 | 1.08 | 0.348 | 0.777 | 1.000 | 804 | tags=27%, list=7%, signal=29% |
| 829 | GOBP\_POLYAMINE\_BIOSYNTHETIC\_PROCESS |  | 12 | 0.36 | 1.08 | 0.342 | 0.777 | 1.000 | 1035 | tags=25%, list=9%, signal=27% |
| 830 | GOBP\_POSITIVE\_REGULATION\_OF\_TRANSLATIONAL\_ELONGATION |  | 3 | 0.60 | 1.08 | 0.382 | 0.776 | 1.000 | 4840 | tags=100%, list=40%, signal=167% |
| 831 | GOBP\_MONOSACCHARIDE\_BIOSYNTHETIC\_PROCESS |  | 70 | 0.22 | 1.08 | 0.280 | 0.777 | 1.000 | 1464 | tags=26%, list=12%, signal=29% |
| 832 | GOBP\_NEGATIVE\_REGULATION\_OF\_LIPASE\_ACTIVITY |  | 11 | 0.37 | 1.07 | 0.345 | 0.777 | 1.000 | 909 | tags=27%, list=8%, signal=29% |
| 833 | GOBP\_NEGATIVE\_REGULATION\_OF\_LOW\_DENSITY\_LIPOPROTEIN\_RECEPTOR\_ACTIVITY |  | 5 | 0.49 | 1.07 | 0.355 | 0.776 | 1.000 | 158 | tags=20%, list=1%, signal=20% |
| 834 | GOBP\_LEYDIG\_CELL\_DIFFERENTIATION |  | 9 | 0.40 | 1.07 | 0.345 | 0.775 | 1.000 | 750 | tags=22%, list=6%, signal=24% |
| 835 | GOBP\_MOTILE\_CILIUM\_ASSEMBLY |  | 9 | 0.39 | 1.07 | 0.350 | 0.775 | 1.000 | 1718 | tags=33%, list=14%, signal=39% |
| 836 | GOBP\_CARDIOLIPIN\_ACYL\_CHAIN\_REMODELING |  | 6 | 0.45 | 1.07 | 0.334 | 0.779 | 1.000 | 2298 | tags=50%, list=19%, signal=62% |
| 837 | GOMF\_C\_METHYLTRANSFERASE\_ACTIVITY |  | 5 | 0.48 | 1.07 | 0.341 | 0.780 | 1.000 | 6189 | tags=100%, list=52%, signal=206% |
| 838 | GOBP\_MITOCHONDRIAL\_RIBOSOME\_ASSEMBLY |  | 6 | 0.45 | 1.07 | 0.345 | 0.780 | 1.000 | 6584 | tags=100%, list=55%, signal=221% |
| 839 | GOBP\_NEGATIVE\_REGULATION\_OF\_SPHINGOLIPID\_BIOSYNTHETIC\_PROCESS |  | 6 | 0.46 | 1.07 | 0.372 | 0.780 | 1.000 | 224 | tags=33%, list=2%, signal=34% |
| 840 | GOBP\_PURINE\_CONTAINING\_COMPOUND\_BIOSYNTHETIC\_PROCESS |  | 127 | 0.20 | 1.07 | 0.236 | 0.779 | 1.000 | 1335 | tags=20%, list=11%, signal=23% |
| 841 | GOBP\_CARDIOLIPIN\_BIOSYNTHETIC\_PROCESS |  | 7 | 0.43 | 1.07 | 0.340 | 0.779 | 1.000 | 2411 | tags=57%, list=20%, signal=71% |
| 842 | GOBP\_POSITIVE\_REGULATION\_OF\_CELL\_ADHESION\_MOLECULE\_PRODUCTION |  | 4 | 0.53 | 1.07 | 0.346 | 0.779 | 1.000 | 1237 | tags=50%, list=10%, signal=56% |
| 843 | GOBP\_XENOBIOTIC\_DETOXIFICATION\_BY\_TRANSMEMBRANE\_EXPORT\_ACROSS\_THE\_PLASMA\_MEMBRANE |  | 5 | 0.49 | 1.07 | 0.356 | 0.779 | 1.000 | 74 | tags=20%, list=1%, signal=20% |
| 844 | GOBP\_TOOTH\_ERUPTION |  | 6 | 0.45 | 1.07 | 0.331 | 0.779 | 1.000 | 196 | tags=17%, list=2%, signal=17% |
| 845 | GOBP\_NEGATIVE\_REGULATION\_OF\_CERAMIDE\_BIOSYNTHETIC\_PROCESS |  | 5 | 0.49 | 1.07 | 0.351 | 0.779 | 1.000 | 224 | tags=40%, list=2%, signal=41% |
| 846 | GOBP\_TRNA\_AMINOACYLATION\_FOR\_MITOCHONDRIAL\_PROTEIN\_TRANSLATION |  | 6 | 0.46 | 1.07 | 0.337 | 0.779 | 1.000 | 6485 | tags=100%, list=54%, signal=217% |
| 847 | GOBP\_REGULATION\_OF\_CILIUM\_BEAT\_FREQUENCY |  | 8 | 0.41 | 1.07 | 0.345 | 0.779 | 1.000 | 68 | tags=13%, list=1%, signal=13% |
| 848 | GOBP\_SMALL\_MOLECULE\_CATABOLIC\_PROCESS |  | 312 | 0.18 | 1.07 | 0.167 | 0.780 | 1.000 | 1648 | tags=20%, list=14%, signal=22% |
| 849 | GOBP\_DETECTION\_OF\_MECHANICAL\_STIMULUS |  | 25 | 0.29 | 1.07 | 0.336 | 0.782 | 1.000 | 329 | tags=8%, list=3%, signal=8% |
| 850 | GOMF\_PEPTIDOGLYCAN\_MURALYTIC\_ACTIVITY |  | 3 | 0.59 | 1.07 | 0.371 | 0.783 | 1.000 | 203 | tags=33%, list=2%, signal=34% |
| 851 | GOBP\_PYRIMIDINE\_DEOXYRIBONUCLEOTIDE\_BIOSYNTHETIC\_PROCESS |  | 6 | 0.45 | 1.07 | 0.353 | 0.783 | 1.000 | 65 | tags=17%, list=1%, signal=17% |
| 852 | GOMF\_CHITIN\_BINDING |  | 3 | 0.59 | 1.07 | 0.374 | 0.782 | 1.000 | 130 | tags=33%, list=1%, signal=34% |
| 853 | GOMF\_CARBOHYDRATE\_PROTON\_SYMPORTER\_ACTIVITY |  | 5 | 0.49 | 1.07 | 0.359 | 0.782 | 1.000 | 595 | tags=40%, list=5%, signal=42% |
| 854 | GOMF\_SULFATIDE\_BINDING |  | 4 | 0.53 | 1.07 | 0.366 | 0.782 | 1.000 | 2631 | tags=75%, list=22%, signal=96% |
| 855 | GOBP\_PSEUDOURIDINE\_SYNTHESIS |  | 15 | 0.33 | 1.07 | 0.341 | 0.782 | 1.000 | 8014 | tags=100%, list=67%, signal=300% |
| 856 | GOBP\_MITOCHONDRIAL\_TRANSMEMBRANE\_TRANSPORT |  | 69 | 0.23 | 1.06 | 0.317 | 0.782 | 1.000 | 1455 | tags=23%, list=12%, signal=26% |
| 857 | GOBP\_POSITIVE\_REGULATION\_OF\_HYPERSENSITIVITY |  | 3 | 0.59 | 1.06 | 0.376 | 0.785 | 1.000 | 4962 | tags=100%, list=41%, signal=170% |
| 858 | GOCC\_ENDOLYSOSOME |  | 17 | 0.32 | 1.06 | 0.350 | 0.787 | 1.000 | 974 | tags=24%, list=8%, signal=26% |
| 859 | GOCC\_EXTRINSIC\_COMPONENT\_OF\_ENDOSOME\_MEMBRANE |  | 9 | 0.39 | 1.06 | 0.371 | 0.786 | 1.000 | 267 | tags=22%, list=2%, signal=23% |
| 860 | GOMF\_ADENYLATE\_KINASE\_ACTIVITY |  | 4 | 0.52 | 1.06 | 0.372 | 0.786 | 1.000 | 1197 | tags=50%, list=10%, signal=56% |
| 861 | GOBP\_POSITIVE\_REGULATION\_OF\_MESENCHYMAL\_CELL\_PROLIFERATION |  | 16 | 0.33 | 1.06 | 0.353 | 0.786 | 1.000 | 489 | tags=13%, list=4%, signal=13% |
| 862 | GOBP\_REGULATION\_OF\_BLOOD\_VESSEL\_REMODELING |  | 4 | 0.53 | 1.06 | 0.369 | 0.787 | 1.000 | 1878 | tags=50%, list=16%, signal=59% |
| 863 | GOMF\_RACEMASE\_AND\_EPIMERASE\_ACTIVITY\_ACTING\_ON\_CARBOHYDRATES\_AND\_DERIVATIVES |  | 7 | 0.43 | 1.06 | 0.356 | 0.790 | 1.000 | 1315 | tags=43%, list=11%, signal=48% |
| 864 | GOBP\_MATURATION\_OF\_SSU\_RRNA |  | 39 | 0.25 | 1.06 | 0.345 | 0.790 | 1.000 | 2359 | tags=31%, list=20%, signal=38% |
| 865 | GOCC\_RDNA\_HETEROCHROMATIN |  | 3 | 0.59 | 1.06 | 0.389 | 0.789 | 1.000 | 1173 | tags=67%, list=10%, signal=74% |
| 866 | GOMF\_GLUCOSE\_BINDING |  | 7 | 0.42 | 1.06 | 0.362 | 0.788 | 1.000 | 361 | tags=29%, list=3%, signal=29% |
| 867 | GOBP\_UREA\_METABOLIC\_PROCESS |  | 6 | 0.44 | 1.06 | 0.367 | 0.787 | 1.000 | 1192 | tags=33%, list=10%, signal=37% |
| 868 | GOBP\_ENDONUCLEOLYTIC\_CLEAVAGE\_INVOLVED\_IN\_RRNA\_PROCESSING |  | 12 | 0.35 | 1.06 | 0.359 | 0.789 | 1.000 | 790 | tags=17%, list=7%, signal=18% |
| 869 | GOBP\_REGULATION\_OF\_MACROAUTOPHAGY |  | 131 | 0.21 | 1.06 | 0.333 | 0.789 | 1.000 | 244 | tags=7%, list=2%, signal=7% |
| 870 | GOBP\_CELL\_PROLIFERATION\_INVOLVED\_IN\_KIDNEY\_DEVELOPMENT |  | 17 | 0.31 | 1.06 | 0.373 | 0.789 | 1.000 | 525 | tags=18%, list=4%, signal=18% |
| 871 | GOMF\_INORGANIC\_ANION\_EXCHANGER\_ACTIVITY |  | 10 | 0.37 | 1.06 | 0.359 | 0.789 | 1.000 | 295 | tags=20%, list=2%, signal=20% |
| 872 | GOBP\_NEGATIVE\_REGULATION\_OF\_MACROPHAGE\_CHEMOTAXIS |  | 5 | 0.48 | 1.06 | 0.376 | 0.790 | 1.000 | 982 | tags=40%, list=8%, signal=44% |
| 873 | GOMF\_STEROL\_ESTERASE\_ACTIVITY |  | 3 | 0.59 | 1.06 | 0.398 | 0.789 | 1.000 | 635 | tags=33%, list=5%, signal=35% |
| 874 | GOBP\_UREA\_CYCLE |  | 5 | 0.48 | 1.06 | 0.369 | 0.788 | 1.000 | 1192 | tags=40%, list=10%, signal=44% |
| 875 | GOBP\_POSITIVE\_REGULATION\_OF\_LOW\_DENSITY\_LIPOPROTEIN\_RECEPTOR\_ACTIVITY |  | 3 | 0.58 | 1.06 | 0.389 | 0.787 | 1.000 | 83 | tags=33%, list=1%, signal=34% |
| 876 | GOMF\_STORE\_OPERATED\_CALCIUM\_CHANNEL\_ACTIVITY |  | 8 | 0.39 | 1.06 | 0.363 | 0.789 | 1.000 | 7274 | tags=100%, list=61%, signal=253% |
| 877 | GOBP\_REGULATION\_OF\_T\_HELPER\_17\_CELL\_LINEAGE\_COMMITMENT |  | 3 | 0.58 | 1.05 | 0.386 | 0.789 | 1.000 | 799 | tags=33%, list=7%, signal=36% |
| 878 | GOBP\_SEX\_DETERMINATION |  | 10 | 0.38 | 1.05 | 0.352 | 0.793 | 1.000 | 750 | tags=20%, list=6%, signal=21% |
| 879 | GOBP\_PH\_ELEVATION |  | 6 | 0.45 | 1.05 | 0.359 | 0.792 | 1.000 | 1 | tags=17%, list=0%, signal=17% |
| 880 | GOBP\_ENDOPLASMIC\_RETICULUM\_TUBULAR\_NETWORK\_ORGANIZATION |  | 17 | 0.31 | 1.05 | 0.384 | 0.792 | 1.000 | 1070 | tags=24%, list=9%, signal=26% |
| 881 | GOBP\_NEGATIVE\_REGULATION\_OF\_CELLULAR\_RESPONSE\_TO\_HYPOXIA |  | 5 | 0.48 | 1.05 | 0.370 | 0.792 | 1.000 | 2221 | tags=60%, list=18%, signal=74% |
| 882 | GOBP\_L\_SERINE\_BIOSYNTHETIC\_PROCESS |  | 5 | 0.48 | 1.05 | 0.372 | 0.792 | 1.000 | 1415 | tags=60%, list=12%, signal=68% |
| 883 | GOBP\_REGULATION\_OF\_NEUROMUSCULAR\_JUNCTION\_DEVELOPMENT |  | 4 | 0.52 | 1.05 | 0.374 | 0.791 | 1.000 | 5723 | tags=100%, list=48%, signal=191% |
| 884 | GOBP\_GALACTOLIPID\_BIOSYNTHETIC\_PROCESS |  | 4 | 0.52 | 1.05 | 0.375 | 0.791 | 1.000 | 827 | tags=50%, list=7%, signal=54% |
| 885 | GOBP\_POSITIVE\_REGULATION\_OF\_OSTEOCLAST\_DIFFERENTIATION |  | 13 | 0.34 | 1.05 | 0.368 | 0.791 | 1.000 | 21 | tags=15%, list=0%, signal=15% |
| 886 | GOBP\_NEPHRIC\_DUCT\_MORPHOGENESIS |  | 7 | 0.42 | 1.05 | 0.363 | 0.790 | 1.000 | 1723 | tags=43%, list=14%, signal=50% |
| 887 | GOBP\_FATTY\_ACID\_BETA\_OXIDATION |  | 62 | 0.23 | 1.05 | 0.323 | 0.791 | 1.000 | 2156 | tags=31%, list=18%, signal=37% |
| 888 | GOMF\_SPHINGOSINE\_1\_PHOSPHATE\_PHOSPHATASE\_ACTIVITY |  | 5 | 0.48 | 1.05 | 0.386 | 0.791 | 1.000 | 133 | tags=20%, list=1%, signal=20% |
| 889 | GOBP\_MRNA\_PSEUDOURIDINE\_SYNTHESIS |  | 5 | 0.48 | 1.05 | 0.378 | 0.790 | 1.000 | 6258 | tags=100%, list=52%, signal=209% |
| 890 | GOBP\_CARBOHYDRATE\_IMPORT\_ACROSS\_PLASMA\_MEMBRANE |  | 4 | 0.52 | 1.05 | 0.374 | 0.793 | 1.000 | 595 | tags=50%, list=5%, signal=53% |
| 891 | GOCC\_ESCRT\_I\_COMPLEX |  | 9 | 0.38 | 1.05 | 0.376 | 0.793 | 1.000 | 770 | tags=22%, list=6%, signal=24% |
| 892 | GOBP\_PYRIMIDINE\_NUCLEOSIDE\_TRIPHOSPHATE\_METABOLIC\_PROCESS |  | 19 | 0.30 | 1.05 | 0.370 | 0.792 | 1.000 | 1093 | tags=16%, list=9%, signal=17% |
| 893 | GOBP\_DEHYDROASCORBIC\_ACID\_TRANSPORT |  | 6 | 0.44 | 1.05 | 0.379 | 0.793 | 1.000 | 595 | tags=33%, list=5%, signal=35% |
| 894 | GOBP\_BONE\_MINERALIZATION\_INVOLVED\_IN\_BONE\_MATURATION |  | 7 | 0.42 | 1.05 | 0.372 | 0.793 | 1.000 | 1156 | tags=43%, list=10%, signal=47% |
| 895 | GOBP\_CELLULAR\_AMINO\_ACID\_CATABOLIC\_PROCESS |  | 76 | 0.22 | 1.05 | 0.359 | 0.794 | 1.000 | 937 | tags=17%, list=8%, signal=18% |
| 896 | GOCC\_EXORIBONUCLEASE\_COMPLEX |  | 22 | 0.29 | 1.05 | 0.380 | 0.793 | 1.000 | 2530 | tags=36%, list=21%, signal=46% |
| 897 | GOBP\_POSITIVE\_REGULATION\_OF\_COAGULATION |  | 16 | 0.32 | 1.05 | 0.374 | 0.794 | 1.000 | 2506 | tags=56%, list=21%, signal=71% |
| 898 | GOBP\_POSITIVE\_REGULATION\_OF\_NUCLEOTIDE\_BIOSYNTHETIC\_PROCESS |  | 11 | 0.36 | 1.05 | 0.369 | 0.794 | 1.000 | 593 | tags=27%, list=5%, signal=29% |
| 899 | GOBP\_NUCLEOSIDE\_MONOPHOSPHATE\_PHOSPHORYLATION |  | 9 | 0.38 | 1.05 | 0.383 | 0.793 | 1.000 | 1197 | tags=33%, list=10%, signal=37% |
| 900 | GOMF\_MONOCARBOXYLATE\_SODIUM\_SYMPORTER\_ACTIVITY |  | 7 | 0.42 | 1.05 | 0.382 | 0.793 | 1.000 | 629 | tags=29%, list=5%, signal=30% |
| 901 | GOBP\_POSITIVE\_REGULATION\_OF\_ERBB\_SIGNALING\_PATHWAY |  | 30 | 0.27 | 1.05 | 0.382 | 0.793 | 1.000 | 1070 | tags=20%, list=9%, signal=22% |
| 902 | GOBP\_DNA\_REPLICATION\_DEPENDENT\_NUCLEOSOME\_ORGANIZATION |  | 7 | 0.42 | 1.04 | 0.381 | 0.798 | 1.000 | 6963 | tags=100%, list=58%, signal=238% |
| 903 | GOMF\_LONG\_CHAIN\_FATTY\_ACID\_BINDING |  | 7 | 0.42 | 1.04 | 0.372 | 0.798 | 1.000 | 1003 | tags=43%, list=8%, signal=47% |
| 904 | GOBP\_POSITIVE\_REGULATION\_OF\_ACTION\_POTENTIAL |  | 3 | 0.58 | 1.04 | 0.399 | 0.797 | 1.000 | 79 | tags=33%, list=1%, signal=34% |
| 905 | GOCC\_ENDOPEPTIDASE\_COMPLEX |  | 54 | 0.23 | 1.04 | 0.382 | 0.797 | 1.000 | 3133 | tags=50%, list=26%, signal=67% |
| 906 | GOBP\_DRINKING\_BEHAVIOR |  | 4 | 0.52 | 1.04 | 0.388 | 0.797 | 1.000 | 558 | tags=50%, list=5%, signal=52% |
| 907 | GOMF\_3\_PHOSPHOADENOSINE\_5\_PHOSPHOSULFATE\_BINDING |  | 3 | 0.57 | 1.04 | 0.391 | 0.797 | 1.000 | 54 | tags=33%, list=0%, signal=33% |
| 908 | GOBP\_CYTOPLASMIC\_TRANSLATION |  | 80 | 0.21 | 1.04 | 0.375 | 0.797 | 1.000 | 3155 | tags=40%, list=26%, signal=54% |
| 909 | GOMF\_CALCIUM\_DEPENDENT\_PROTEIN\_BINDING |  | 43 | 0.24 | 1.04 | 0.369 | 0.799 | 1.000 | 2006 | tags=35%, list=17%, signal=42% |
| 910 | GOMF\_D\_GLUCOSE\_TRANSMEMBRANE\_TRANSPORTER\_ACTIVITY |  | 3 | 0.57 | 1.04 | 0.388 | 0.798 | 1.000 | 595 | tags=67%, list=5%, signal=70% |
| 911 | GOBP\_G\_PROTEIN\_COUPLED\_RECEPTOR\_INTERNALIZATION |  | 8 | 0.40 | 1.04 | 0.372 | 0.798 | 1.000 | 1587 | tags=50%, list=13%, signal=58% |
| 912 | GOBP\_REGULATION\_OF\_APOPTOTIC\_PROCESS\_INVOLVED\_IN\_DEVELOPMENT |  | 5 | 0.47 | 1.04 | 0.396 | 0.797 | 1.000 | 6319 | tags=100%, list=53%, signal=211% |
| 913 | GOBP\_NEGATIVE\_REGULATION\_OF\_CELL\_PROLIFERATION\_INVOLVED\_IN\_KIDNEY\_DEVELOPMENT |  | 4 | 0.52 | 1.04 | 0.398 | 0.796 | 1.000 | 1723 | tags=50%, list=14%, signal=58% |
| 914 | GOMF\_BH\_DOMAIN\_BINDING |  | 8 | 0.40 | 1.04 | 0.377 | 0.796 | 1.000 | 775 | tags=25%, list=6%, signal=27% |
| 915 | GOBP\_IMMATURE\_T\_CELL\_PROLIFERATION |  | 8 | 0.40 | 1.04 | 0.390 | 0.796 | 1.000 | 1723 | tags=38%, list=14%, signal=44% |
| 916 | GOBP\_CELLULAR\_KETONE\_METABOLIC\_PROCESS |  | 176 | 0.19 | 1.04 | 0.349 | 0.795 | 1.000 | 1678 | tags=21%, list=14%, signal=24% |
| 917 | GOBP\_MEMBRANE\_RAFT\_ASSEMBLY |  | 8 | 0.40 | 1.04 | 0.388 | 0.795 | 1.000 | 2736 | tags=63%, list=23%, signal=81% |
| 918 | GOCC\_SAM\_COMPLEX |  | 10 | 0.37 | 1.04 | 0.368 | 0.795 | 1.000 | 2097 | tags=50%, list=17%, signal=61% |
| 919 | GOBP\_ACETYL\_COA\_METABOLIC\_PROCESS |  | 33 | 0.26 | 1.04 | 0.382 | 0.795 | 1.000 | 1150 | tags=24%, list=10%, signal=27% |
| 920 | GOBP\_PROTEASOMAL\_UBIQUITIN\_INDEPENDENT\_PROTEIN\_CATABOLIC\_PROCESS |  | 15 | 0.33 | 1.04 | 0.405 | 0.794 | 1.000 | 3133 | tags=67%, list=26%, signal=90% |
| 921 | GOMF\_CARBON\_OXYGEN\_LYASE\_ACTIVITY |  | 58 | 0.23 | 1.04 | 0.360 | 0.794 | 1.000 | 587 | tags=16%, list=5%, signal=16% |
| 922 | GOBP\_REGULATION\_OF\_NUCLEOTIDE\_BIOSYNTHETIC\_PROCESS |  | 23 | 0.29 | 1.04 | 0.393 | 0.796 | 1.000 | 607 | tags=22%, list=5%, signal=23% |
| 923 | GOBP\_SPHINGOLIPID\_MEDIATED\_SIGNALING\_PATHWAY |  | 11 | 0.35 | 1.04 | 0.363 | 0.796 | 1.000 | 447 | tags=18%, list=4%, signal=19% |
| 924 | GOMF\_CULLIN\_FAMILY\_PROTEIN\_BINDING |  | 19 | 0.30 | 1.04 | 0.388 | 0.795 | 1.000 | 1327 | tags=21%, list=11%, signal=24% |
| 925 | GOBP\_NEGATIVE\_REGULATION\_OF\_ENDOPLASMIC\_RETICULUM\_CALCIUM\_ION\_CONCENTRATION |  | 5 | 0.47 | 1.04 | 0.384 | 0.795 | 1.000 | 1053 | tags=40%, list=9%, signal=44% |
| 926 | GOBP\_REGULATION\_OF\_CELLULAR\_KETONE\_METABOLIC\_PROCESS |  | 132 | 0.20 | 1.04 | 0.340 | 0.794 | 1.000 | 1678 | tags=21%, list=14%, signal=24% |
| 927 | GOBP\_VAGINA\_DEVELOPMENT |  | 6 | 0.43 | 1.04 | 0.385 | 0.794 | 1.000 | 2962 | tags=83%, list=25%, signal=111% |
| 928 | GOBP\_CELLULAR\_COPPER\_ION\_HOMEOSTASIS |  | 9 | 0.38 | 1.04 | 0.383 | 0.793 | 1.000 | 2461 | tags=44%, list=20%, signal=56% |
| 929 | GOMF\_STRUCTURAL\_CONSTITUENT\_OF\_MUSCLE |  | 22 | 0.29 | 1.04 | 0.382 | 0.793 | 1.000 | 1652 | tags=27%, list=14%, signal=32% |
| 930 | GOCC\_ENDOLYSOSOME\_MEMBRANE |  | 11 | 0.35 | 1.04 | 0.382 | 0.793 | 1.000 | 158 | tags=18%, list=1%, signal=18% |
| 931 | GOBP\_MYOBLAST\_MIGRATION |  | 10 | 0.37 | 1.04 | 0.392 | 0.793 | 1.000 | 267 | tags=10%, list=2%, signal=10% |
| 932 | GOBP\_REGULATION\_OF\_CILIUM\_MOVEMENT |  | 13 | 0.34 | 1.03 | 0.390 | 0.793 | 1.000 | 265 | tags=15%, list=2%, signal=16% |
| 933 | GOBP\_ISOCITRATE\_METABOLIC\_PROCESS |  | 4 | 0.51 | 1.03 | 0.392 | 0.792 | 1.000 | 5855 | tags=100%, list=49%, signal=195% |
| 934 | GOBP\_RIBOSE\_PHOSPHATE\_BIOSYNTHETIC\_PROCESS |  | 125 | 0.20 | 1.03 | 0.388 | 0.792 | 1.000 | 1335 | tags=19%, list=11%, signal=21% |
| 935 | GOBP\_REGULATION\_OF\_EXTRACELLULAR\_MATRIX\_CONSTITUENT\_SECRETION |  | 6 | 0.44 | 1.03 | 0.383 | 0.792 | 1.000 | 1069 | tags=33%, list=9%, signal=37% |
| 936 | GOBP\_COPPER\_ION\_HOMEOSTASIS |  | 11 | 0.36 | 1.03 | 0.384 | 0.792 | 1.000 | 2461 | tags=36%, list=20%, signal=46% |
| 937 | GOBP\_PROTEIN\_K11\_LINKED\_UBIQUITINATION |  | 24 | 0.28 | 1.03 | 0.378 | 0.791 | 1.000 | 1809 | tags=25%, list=15%, signal=29% |
| 938 | GOCC\_SIGNAL\_PEPTIDASE\_COMPLEX |  | 4 | 0.51 | 1.03 | 0.389 | 0.791 | 1.000 | 5935 | tags=100%, list=49%, signal=198% |
| 939 | GOMF\_ISOCITRATE\_DEHYDROGENASE\_ACTIVITY |  | 4 | 0.51 | 1.03 | 0.381 | 0.791 | 1.000 | 5855 | tags=100%, list=49%, signal=195% |
| 940 | GOBP\_REGULATION\_OF\_PHOSPHOLIPID\_CATABOLIC\_PROCESS |  | 4 | 0.51 | 1.03 | 0.403 | 0.791 | 1.000 | 5855 | tags=100%, list=49%, signal=195% |
| 941 | GOBP\_NEGATIVE\_REGULATION\_OF\_NERVOUS\_SYSTEM\_PROCESS |  | 11 | 0.36 | 1.03 | 0.403 | 0.790 | 1.000 | 993 | tags=27%, list=8%, signal=30% |
| 942 | GOBP\_ALPHA\_AMINO\_ACID\_METABOLIC\_PROCESS |  | 130 | 0.20 | 1.03 | 0.373 | 0.790 | 1.000 | 1415 | tags=21%, list=12%, signal=23% |
| 943 | GOMF\_CYTIDYLYLTRANSFERASE\_ACTIVITY |  | 7 | 0.41 | 1.03 | 0.391 | 0.789 | 1.000 | 135 | tags=14%, list=1%, signal=14% |
| 944 | GOMF\_SODIUM\_CHANNEL\_INHIBITOR\_ACTIVITY |  | 6 | 0.44 | 1.03 | 0.388 | 0.791 | 1.000 | 472 | tags=33%, list=4%, signal=35% |
| 945 | GOBP\_METHYLGLYOXAL\_METABOLIC\_PROCESS |  | 5 | 0.47 | 1.03 | 0.400 | 0.791 | 1.000 | 6331 | tags=100%, list=53%, signal=211% |
| 946 | GOBP\_NEGATIVE\_REGULATION\_OF\_RIG\_I\_SIGNALING\_PATHWAY |  | 6 | 0.44 | 1.03 | 0.392 | 0.791 | 1.000 | 1306 | tags=33%, list=11%, signal=37% |
| 947 | GOBP\_ALDITOL\_BIOSYNTHETIC\_PROCESS |  | 6 | 0.44 | 1.03 | 0.394 | 0.791 | 1.000 | 673 | tags=33%, list=6%, signal=35% |
| 948 | GOBP\_MONOSACCHARIDE\_CATABOLIC\_PROCESS |  | 47 | 0.24 | 1.03 | 0.390 | 0.794 | 1.000 | 1465 | tags=23%, list=12%, signal=27% |
| 949 | GOBP\_POSITIVE\_REGULATION\_OF\_SMOOTHENED\_SIGNALING\_PATHWAY |  | 26 | 0.28 | 1.03 | 0.399 | 0.794 | 1.000 | 1097 | tags=23%, list=9%, signal=25% |
| 950 | GOCC\_APICOLATERAL\_PLASMA\_MEMBRANE |  | 12 | 0.34 | 1.03 | 0.387 | 0.795 | 1.000 | 374 | tags=17%, list=3%, signal=17% |
| 951 | GOBP\_POSITIVE\_REGULATION\_OF\_T\_HELPER\_2\_CELL\_CYTOKINE\_PRODUCTION |  | 6 | 0.44 | 1.03 | 0.385 | 0.794 | 1.000 | 525 | tags=33%, list=4%, signal=35% |
| 952 | GOMF\_NEDD8\_TRANSFERASE\_ACTIVITY |  | 6 | 0.44 | 1.03 | 0.393 | 0.794 | 1.000 | 1495 | tags=50%, list=12%, signal=57% |
| 953 | GOBP\_GLUCOSE\_IMPORT\_ACROSS\_PLASMA\_MEMBRANE |  | 3 | 0.57 | 1.03 | 0.419 | 0.794 | 1.000 | 595 | tags=67%, list=5%, signal=70% |
| 954 | GOBP\_MONOCARBOXYLIC\_ACID\_CATABOLIC\_PROCESS |  | 100 | 0.20 | 1.03 | 0.405 | 0.794 | 1.000 | 1530 | tags=21%, list=13%, signal=24% |
| 955 | GOBP\_REGULATION\_OF\_KETONE\_BIOSYNTHETIC\_PROCESS |  | 14 | 0.33 | 1.03 | 0.403 | 0.796 | 1.000 | 602 | tags=29%, list=5%, signal=30% |
| 956 | GOMF\_DEHYDROASCORBIC\_ACID\_TRANSMEMBRANE\_TRANSPORTER\_ACTIVITY |  | 6 | 0.44 | 1.03 | 0.401 | 0.796 | 1.000 | 595 | tags=33%, list=5%, signal=35% |
| 957 | GOBP\_ORGANISM\_EMERGENCE\_FROM\_PROTECTIVE\_STRUCTURE |  | 11 | 0.35 | 1.03 | 0.402 | 0.795 | 1.000 | 7765 | tags=100%, list=65%, signal=283% |
| 958 | GOBP\_NEGATIVE\_REGULATION\_OF\_LIPID\_CATABOLIC\_PROCESS |  | 14 | 0.32 | 1.02 | 0.394 | 0.797 | 1.000 | 1237 | tags=29%, list=10%, signal=32% |
| 959 | GOBP\_RIBONUCLEOSIDE\_DIPHOSPHATE\_BIOSYNTHETIC\_PROCESS |  | 5 | 0.47 | 1.02 | 0.407 | 0.797 | 1.000 | 1965 | tags=60%, list=16%, signal=72% |
| 960 | GOBP\_RIBOSOMAL\_LARGE\_SUBUNIT\_ASSEMBLY |  | 22 | 0.29 | 1.02 | 0.424 | 0.796 | 1.000 | 2783 | tags=41%, list=23%, signal=53% |
| 961 | GOBP\_NEGATIVE\_REGULATION\_OF\_POSTTRANSCRIPTIONAL\_GENE\_SILENCING |  | 12 | 0.34 | 1.02 | 0.410 | 0.795 | 1.000 | 1017 | tags=25%, list=8%, signal=27% |
| 962 | GOBP\_PRESYNAPTIC\_DENSE\_CORE\_VESICLE\_EXOCYTOSIS |  | 5 | 0.47 | 1.02 | 0.400 | 0.794 | 1.000 | 27 | tags=20%, list=0%, signal=20% |
| 963 | GOBP\_RESPONSE\_TO\_OXYGEN\_RADICAL |  | 17 | 0.30 | 1.02 | 0.381 | 0.794 | 1.000 | 228 | tags=12%, list=2%, signal=12% |
| 964 | GOBP\_PROTEIN\_PEPTIDYL\_PROLYL\_ISOMERIZATION |  | 30 | 0.26 | 1.02 | 0.416 | 0.795 | 1.000 | 2787 | tags=37%, list=23%, signal=48% |
| 965 | GOCC\_U12\_TYPE\_SPLICEOSOMAL\_COMPLEX |  | 24 | 0.28 | 1.02 | 0.402 | 0.797 | 1.000 | 2573 | tags=42%, list=21%, signal=53% |
| 966 | GOMF\_CYCLIN\_BINDING |  | 20 | 0.30 | 1.02 | 0.420 | 0.797 | 1.000 | 843 | tags=15%, list=7%, signal=16% |
| 967 | GOCC\_CHECKPOINT\_CLAMP\_COMPLEX |  | 3 | 0.57 | 1.02 | 0.414 | 0.796 | 1.000 | 898 | tags=33%, list=7%, signal=36% |
| 968 | GOBP\_REGULATION\_OF\_MYELOID\_CELL\_APOPTOTIC\_PROCESS |  | 17 | 0.30 | 1.02 | 0.397 | 0.795 | 1.000 | 267 | tags=18%, list=2%, signal=18% |
| 969 | GOBP\_NUCLEAR\_TRANSCRIBED\_MRNA\_CATABOLIC\_PROCESS\_NONSENSE\_MEDIATED\_DECAY |  | 101 | 0.20 | 1.02 | 0.396 | 0.795 | 1.000 | 3085 | tags=44%, list=26%, signal=58% |
| 970 | GOBP\_DRUG\_METABOLIC\_PROCESS |  | 19 | 0.29 | 1.02 | 0.409 | 0.795 | 1.000 | 772 | tags=26%, list=6%, signal=28% |
| 971 | GOMF\_SECONDARY\_ACTIVE\_MONOCARBOXYLATE\_TRANSMEMBRANE\_TRANSPORTER\_ACTIVITY |  | 8 | 0.39 | 1.02 | 0.407 | 0.794 | 1.000 | 629 | tags=25%, list=5%, signal=26% |
| 972 | GOBP\_HYPOTHALAMUS\_CELL\_DIFFERENTIATION |  | 5 | 0.46 | 1.02 | 0.401 | 0.795 | 1.000 | 6450 | tags=100%, list=54%, signal=216% |
| 973 | GOMF\_SERINE\_TRANSMEMBRANE\_TRANSPORTER\_ACTIVITY |  | 6 | 0.43 | 1.02 | 0.403 | 0.795 | 1.000 | 3496 | tags=83%, list=29%, signal=117% |
| 974 | GOBP\_ONE\_CARBON\_METABOLIC\_PROCESS |  | 29 | 0.27 | 1.02 | 0.406 | 0.794 | 1.000 | 284 | tags=14%, list=2%, signal=14% |
| 975 | GOBP\_PLASMA\_MEMBRANE\_PHOSPHOLIPID\_SCRAMBLING |  | 10 | 0.36 | 1.02 | 0.410 | 0.794 | 1.000 | 291 | tags=20%, list=2%, signal=20% |
| 976 | GOMF\_ACYL\_COA\_DEHYDROGENASE\_ACTIVITY |  | 8 | 0.39 | 1.02 | 0.405 | 0.793 | 1.000 | 1895 | tags=38%, list=16%, signal=44% |
| 977 | GOBP\_POSITIVE\_REGULATION\_OF\_INFLAMMATORY\_RESPONSE\_TO\_ANTIGENIC\_STIMULUS |  | 7 | 0.41 | 1.02 | 0.386 | 0.795 | 1.000 | 7105 | tags=100%, list=59%, signal=245% |
| 978 | GOBP\_POSITIVE\_REGULATION\_OF\_GLYCOGEN\_STARCH\_SYNTHASE\_ACTIVITY |  | 3 | 0.56 | 1.02 | 0.415 | 0.795 | 1.000 | 5316 | tags=100%, list=44%, signal=179% |
| 979 | GOBP\_RESPONSE\_TO\_VITAMIN\_A |  | 9 | 0.37 | 1.02 | 0.411 | 0.796 | 1.000 | 309 | tags=22%, list=3%, signal=23% |
| 980 | GOBP\_MEMBRANOUS\_SEPTUM\_MORPHOGENESIS |  | 5 | 0.47 | 1.02 | 0.416 | 0.797 | 1.000 | 6390 | tags=100%, list=53%, signal=214% |
| 981 | GOBP\_POSITIVE\_REGULATION\_OF\_TELOMERASE\_RNA\_LOCALIZATION\_TO\_CAJAL\_BODY |  | 13 | 0.33 | 1.02 | 0.414 | 0.797 | 1.000 | 8014 | tags=100%, list=67%, signal=300% |
| 982 | GOMF\_DOUBLE\_STRANDED\_RNA\_BINDING |  | 55 | 0.22 | 1.02 | 0.414 | 0.798 | 1.000 | 340 | tags=11%, list=3%, signal=11% |
| 983 | GOBP\_MIDBODY\_ABSCISSION |  | 16 | 0.31 | 1.02 | 0.425 | 0.800 | 1.000 | 2585 | tags=56%, list=22%, signal=72% |
| 984 | GOBP\_PROTEIN\_LOCALIZATION\_TO\_NUCLEAR\_BODY |  | 11 | 0.35 | 1.02 | 0.410 | 0.800 | 1.000 | 7830 | tags=100%, list=65%, signal=287% |
| 985 | GOMF\_METAL\_CLUSTER\_BINDING |  | 55 | 0.22 | 1.02 | 0.433 | 0.800 | 1.000 | 1492 | tags=25%, list=12%, signal=29% |
| 986 | GOBP\_CELLULAR\_RESPONSE\_TO\_ELECTRICAL\_STIMULUS |  | 5 | 0.46 | 1.01 | 0.405 | 0.801 | 1.000 | 79 | tags=20%, list=1%, signal=20% |
| 987 | GOBP\_POSITIVE\_REGULATION\_OF\_T\_HELPER\_1\_CELL\_DIFFERENTIATION |  | 6 | 0.43 | 1.01 | 0.415 | 0.801 | 1.000 | 267 | tags=17%, list=2%, signal=17% |
| 988 | GOCC\_CYTOPLASMIC\_EXOSOME\_RNASE\_COMPLEX |  | 13 | 0.33 | 1.01 | 0.409 | 0.801 | 1.000 | 2421 | tags=38%, list=20%, signal=48% |
| 989 | GOBP\_PEPTIDYL\_GLUTAMINE\_METHYLATION |  | 3 | 0.56 | 1.01 | 0.430 | 0.801 | 1.000 | 5240 | tags=100%, list=44%, signal=177% |
| 990 | GOBP\_MITOCHONDRIAL\_RNA\_CATABOLIC\_PROCESS |  | 6 | 0.43 | 1.01 | 0.403 | 0.801 | 1.000 | 6848 | tags=100%, list=57%, signal=233% |
| 991 | GOBP\_POSITIVE\_REGULATION\_OF\_HORMONE\_METABOLIC\_PROCESS |  | 9 | 0.37 | 1.01 | 0.422 | 0.801 | 1.000 | 1478 | tags=33%, list=12%, signal=38% |
| 992 | GOMF\_STAT\_FAMILY\_PROTEIN\_BINDING |  | 5 | 0.46 | 1.01 | 0.421 | 0.800 | 1.000 | 876 | tags=40%, list=7%, signal=43% |
| 993 | GOBP\_AMINOGLYCAN\_CATABOLIC\_PROCESS |  | 47 | 0.23 | 1.01 | 0.412 | 0.802 | 1.000 | 580 | tags=11%, list=5%, signal=11% |
| 994 | GOMF\_ACTIVIN\_RECEPTOR\_BINDING |  | 6 | 0.43 | 1.01 | 0.417 | 0.804 | 1.000 | 6804 | tags=100%, list=57%, signal=231% |
| 995 | GOBP\_NEGATIVE\_REGULATION\_OF\_DENDRITE\_MORPHOGENESIS |  | 6 | 0.43 | 1.01 | 0.406 | 0.803 | 1.000 | 170 | tags=17%, list=1%, signal=17% |
| 996 | GOBP\_REGULATION\_OF\_FATTY\_ACID\_METABOLIC\_PROCESS |  | 66 | 0.22 | 1.01 | 0.453 | 0.803 | 1.000 | 1377 | tags=20%, list=11%, signal=22% |
| 997 | GOBP\_PROTEIN\_LOCALIZATION\_TO\_NUCLEOPLASM |  | 11 | 0.35 | 1.01 | 0.423 | 0.803 | 1.000 | 7830 | tags=100%, list=65%, signal=287% |
| 998 | GOBP\_REGULATION\_OF\_PHOTORECEPTOR\_CELL\_DIFFERENTIATION |  | 4 | 0.50 | 1.01 | 0.407 | 0.802 | 1.000 | 6045 | tags=100%, list=50%, signal=201% |
| 999 | GOMF\_PHOSPHOLIPASE\_A1\_ACTIVITY |  | 5 | 0.46 | 1.01 | 0.418 | 0.804 | 1.000 | 1363 | tags=40%, list=11%, signal=45% |
| 1000 | GOBP\_CYCLOOXYGENASE\_PATHWAY |  | 8 | 0.38 | 1.01 | 0.411 | 0.804 | 1.000 | 416 | tags=25%, list=3%, signal=26% |
| 1001 | GOBP\_DEOXYRIBONUCLEOTIDE\_METABOLIC\_PROCESS |  | 30 | 0.26 | 1.01 | 0.405 | 0.803 | 1.000 | 383 | tags=13%, list=3%, signal=14% |
| 1002 | GOMF\_RNA\_POLYMERASE\_II\_CORE\_PROMOTER\_SEQUENCE\_SPECIFIC\_DNA\_BINDING |  | 11 | 0.35 | 1.01 | 0.429 | 0.804 | 1.000 | 908 | tags=18%, list=8%, signal=20% |
| 1003 | GOBP\_MESENCHYMAL\_CELL\_PROLIFERATION |  | 28 | 0.26 | 1.01 | 0.425 | 0.803 | 1.000 | 1754 | tags=29%, list=15%, signal=33% |
| 1004 | GOBP\_NEGATIVE\_REGULATION\_OF\_FATTY\_ACID\_OXIDATION |  | 10 | 0.36 | 1.01 | 0.421 | 0.803 | 1.000 | 50 | tags=10%, list=0%, signal=10% |
| 1005 | GOBP\_CELLULAR\_SPHINGOLIPID\_HOMEOSTASIS |  | 5 | 0.46 | 1.01 | 0.423 | 0.803 | 1.000 | 1070 | tags=40%, list=9%, signal=44% |
| 1006 | GOBP\_NEGATIVE\_REGULATION\_OF\_STEM\_CELL\_PROLIFERATION |  | 3 | 0.55 | 1.01 | 0.421 | 0.803 | 1.000 | 5369 | tags=100%, list=45%, signal=181% |
| 1007 | GOBP\_ENDOTHELIN\_RECEPTOR\_SIGNALING\_PATHWAY |  | 7 | 0.40 | 1.01 | 0.421 | 0.804 | 1.000 | 7166 | tags=100%, list=60%, signal=248% |
| 1008 | GOMF\_ATP\_TRANSMEMBRANE\_TRANSPORTER\_ACTIVITY |  | 8 | 0.39 | 1.01 | 0.425 | 0.804 | 1.000 | 2023 | tags=38%, list=17%, signal=45% |
| 1009 | GOMF\_S\_METHYLTRANSFERASE\_ACTIVITY |  | 4 | 0.50 | 1.01 | 0.419 | 0.806 | 1.000 | 713 | tags=25%, list=6%, signal=27% |
| 1010 | GOBP\_PIGMENT\_CATABOLIC\_PROCESS |  | 4 | 0.50 | 1.01 | 0.422 | 0.806 | 1.000 | 1320 | tags=75%, list=11%, signal=84% |
| 1011 | GOBP\_RESPONSE\_TO\_HYDROPEROXIDE |  | 14 | 0.32 | 1.00 | 0.424 | 0.805 | 1.000 | 190 | tags=14%, list=2%, signal=14% |
| 1012 | GOBP\_RESPONSE\_TO\_INTERFERON\_ALPHA |  | 11 | 0.34 | 1.00 | 0.443 | 0.806 | 1.000 | 1017 | tags=36%, list=8%, signal=40% |
| 1013 | GOBP\_TRNA\_PSEUDOURIDINE\_SYNTHESIS |  | 5 | 0.46 | 1.00 | 0.432 | 0.805 | 1.000 | 6506 | tags=100%, list=54%, signal=218% |
| 1014 | GOBP\_FERTILIZATION |  | 75 | 0.21 | 1.00 | 0.455 | 0.805 | 1.000 | 1538 | tags=21%, list=13%, signal=24% |
| 1015 | GOMF\_E\_BOX\_BINDING |  | 32 | 0.26 | 1.00 | 0.427 | 0.804 | 1.000 | 1590 | tags=28%, list=13%, signal=32% |
| 1016 | GOBP\_ESTABLISHMENT\_OF\_BLOOD\_BRAIN\_BARRIER |  | 6 | 0.43 | 1.00 | 0.423 | 0.804 | 1.000 | 374 | tags=17%, list=3%, signal=17% |
| 1017 | GOBP\_CELLULAR\_RESPONSE\_TO\_LAMINAR\_FLUID\_SHEAR\_STRESS |  | 6 | 0.43 | 1.00 | 0.418 | 0.804 | 1.000 | 1192 | tags=33%, list=10%, signal=37% |
| 1018 | GOBP\_REGULATION\_OF\_PHOSPHOLIPASE\_C\_ACTIVATING\_G\_PROTEIN\_COUPLED\_RECEPTOR\_SIGNALING\_PATHWAY |  | 4 | 0.50 | 1.00 | 0.445 | 0.807 | 1.000 | 1630 | tags=50%, list=14%, signal=58% |
| 1019 | GOCC\_MYOSIN\_FILAMENT |  | 8 | 0.38 | 1.00 | 0.425 | 0.809 | 1.000 | 575 | tags=25%, list=5%, signal=26% |
| 1020 | GOBP\_NEGATIVE\_REGULATION\_OF\_CARBOHYDRATE\_METABOLIC\_PROCESS |  | 32 | 0.25 | 1.00 | 0.443 | 0.809 | 1.000 | 339 | tags=9%, list=3%, signal=10% |
| 1021 | GOBP\_ESTABLISHMENT\_OF\_PROTEIN\_LOCALIZATION\_TO\_MITOCHONDRIAL\_MEMBRANE |  | 37 | 0.24 | 1.00 | 0.448 | 0.808 | 1.000 | 2341 | tags=35%, list=19%, signal=44% |
| 1022 | GOBP\_REGULATION\_OF\_OXIDATIVE\_STRESS\_INDUCED\_INTRINSIC\_APOPTOTIC\_SIGNALING\_PATHWAY |  | 23 | 0.27 | 1.00 | 0.456 | 0.808 | 1.000 | 463 | tags=13%, list=4%, signal=14% |
| 1023 | GOBP\_POSITIVE\_REGULATION\_OF\_MITOCHONDRION\_ORGANIZATION |  | 61 | 0.22 | 1.00 | 0.462 | 0.809 | 1.000 | 1211 | tags=16%, list=10%, signal=18% |
| 1024 | GOCC\_ARYL\_HYDROCARBON\_RECEPTOR\_COMPLEX |  | 8 | 0.38 | 1.00 | 0.434 | 0.810 | 1.000 | 2323 | tags=38%, list=19%, signal=46% |
| 1025 | GOMF\_CATALYTIC\_ACTIVITY\_ACTING\_ON\_A\_TRNA |  | 85 | 0.20 | 1.00 | 0.460 | 0.811 | 1.000 | 2633 | tags=34%, list=22%, signal=43% |
| 1026 | GOMF\_LARGE\_RIBOSOMAL\_SUBUNIT\_RRNA\_BINDING |  | 3 | 0.55 | 1.00 | 0.447 | 0.811 | 1.000 | 5368 | tags=100%, list=45%, signal=181% |
| 1027 | GOBP\_RESPONSE\_TO\_VITAMIN\_E |  | 9 | 0.37 | 1.00 | 0.438 | 0.811 | 1.000 | 249 | tags=22%, list=2%, signal=23% |
| 1028 | GOBP\_ONE\_CARBON\_COMPOUND\_TRANSPORT |  | 8 | 0.38 | 1.00 | 0.443 | 0.813 | 1.000 | 1548 | tags=38%, list=13%, signal=43% |
| 1029 | GOBP\_ACETYL\_COA\_BIOSYNTHETIC\_PROCESS\_FROM\_PYRUVATE |  | 13 | 0.32 | 1.00 | 0.433 | 0.814 | 1.000 | 607 | tags=23%, list=5%, signal=24% |
| 1030 | GOBP\_PYRIMIDINE\_DEOXYRIBONUCLEOTIDE\_METABOLIC\_PROCESS |  | 17 | 0.30 | 1.00 | 0.466 | 0.813 | 1.000 | 346 | tags=12%, list=3%, signal=12% |
| 1031 | GOBP\_NEGATIVE\_REGULATION\_OF\_PHOTORECEPTOR\_CELL\_DIFFERENTIATION |  | 4 | 0.50 | 0.99 | 0.441 | 0.816 | 1.000 | 6045 | tags=100%, list=50%, signal=201% |
| 1032 | GOBP\_COPPER\_ION\_TRANSPORT |  | 13 | 0.33 | 0.99 | 0.445 | 0.816 | 1.000 | 363 | tags=15%, list=3%, signal=16% |
| 1033 | GOBP\_POSITIVE\_REGULATION\_OF\_VOLTAGE\_GATED\_POTASSIUM\_CHANNEL\_ACTIVITY |  | 7 | 0.40 | 0.99 | 0.444 | 0.815 | 1.000 | 1854 | tags=43%, list=15%, signal=51% |
| 1034 | GOBP\_GLYCINE\_METABOLIC\_PROCESS |  | 12 | 0.33 | 0.99 | 0.434 | 0.816 | 1.000 | 1224 | tags=25%, list=10%, signal=28% |
| 1035 | GOBP\_REGULATION\_OF\_SPHINGOLIPID\_BIOSYNTHETIC\_PROCESS |  | 14 | 0.32 | 0.99 | 0.447 | 0.816 | 1.000 | 224 | tags=14%, list=2%, signal=15% |
| 1036 | GOBP\_NEGATIVE\_REGULATION\_OF\_ADENYLATE\_CYCLASE\_ACTIVATING\_ADRENERGIC\_RECEPTOR\_SIGNALING\_PATHWAY |  | 4 | 0.49 | 0.99 | 0.448 | 0.816 | 1.000 | 6109 | tags=100%, list=51%, signal=203% |
| 1037 | GOBP\_POSITIVE\_REGULATION\_OF\_MICROTUBULE\_BINDING |  | 5 | 0.45 | 0.99 | 0.436 | 0.815 | 1.000 | 6549 | tags=100%, list=55%, signal=220% |
| 1038 | GOMF\_ISOPRENOID\_BINDING |  | 18 | 0.29 | 0.99 | 0.451 | 0.815 | 1.000 | 817 | tags=28%, list=7%, signal=30% |
| 1039 | GOBP\_POSITIVE\_REGULATION\_OF\_CELLULAR\_AMINE\_METABOLIC\_PROCESS |  | 5 | 0.45 | 0.99 | 0.436 | 0.814 | 1.000 | 108 | tags=20%, list=1%, signal=20% |
| 1040 | GOBP\_NEGATIVE\_REGULATION\_OF\_FIBROBLAST\_MIGRATION |  | 4 | 0.49 | 0.99 | 0.449 | 0.814 | 1.000 | 6122 | tags=100%, list=51%, signal=204% |
| 1041 | GOMF\_MACROLIDE\_BINDING |  | 8 | 0.38 | 0.99 | 0.437 | 0.814 | 1.000 | 1128 | tags=25%, list=9%, signal=28% |
| 1042 | GOBP\_OXALATE\_TRANSPORT |  | 6 | 0.41 | 0.99 | 0.440 | 0.815 | 1.000 | 452 | tags=33%, list=4%, signal=35% |
| 1043 | GOBP\_PROTEIN\_DEPALMITOYLATION |  | 10 | 0.35 | 0.99 | 0.421 | 0.817 | 1.000 | 2200 | tags=50%, list=18%, signal=61% |
| 1044 | GOCC\_DENDRITIC\_SPINE\_HEAD |  | 5 | 0.45 | 0.99 | 0.443 | 0.817 | 1.000 | 16 | tags=20%, list=0%, signal=20% |
| 1045 | GOBP\_POSITIVE\_REGULATION\_OF\_INTEGRIN\_ACTIVATION |  | 9 | 0.36 | 0.99 | 0.432 | 0.816 | 1.000 | 126 | tags=11%, list=1%, signal=11% |
| 1046 | GOMF\_1\_ACYLGLYCEROPHOSPHOCHOLINE\_O\_ACYLTRANSFERASE\_ACTIVITY |  | 7 | 0.40 | 0.99 | 0.433 | 0.816 | 1.000 | 979 | tags=29%, list=8%, signal=31% |
| 1047 | GOBP\_CITRULLINE\_BIOSYNTHETIC\_PROCESS |  | 4 | 0.49 | 0.99 | 0.442 | 0.816 | 1.000 | 6109 | tags=100%, list=51%, signal=203% |
| 1048 | GOBP\_MITOCHONDRIAL\_OUTER\_MEMBRANE\_PERMEABILIZATION |  | 37 | 0.24 | 0.99 | 0.462 | 0.816 | 1.000 | 775 | tags=16%, list=6%, signal=17% |
| 1049 | GOBP\_POST\_EMBRYONIC\_EYE\_MORPHOGENESIS |  | 3 | 0.55 | 0.99 | 0.452 | 0.817 | 1.000 | 775 | tags=33%, list=6%, signal=36% |
| 1050 | GOMF\_MORPHOGEN\_ACTIVITY |  | 4 | 0.49 | 0.99 | 0.442 | 0.817 | 1.000 | 1130 | tags=50%, list=9%, signal=55% |
| 1051 | GOCC\_PALMITOYLTRANSFERASE\_COMPLEX |  | 9 | 0.36 | 0.99 | 0.444 | 0.817 | 1.000 | 1354 | tags=33%, list=11%, signal=38% |
| 1052 | GOBP\_NEGATIVE\_REGULATION\_OF\_CELL\_ADHESION\_MOLECULE\_PRODUCTION |  | 3 | 0.55 | 0.99 | 0.444 | 0.817 | 1.000 | 5412 | tags=100%, list=45%, signal=182% |
| 1053 | GOBP\_NEGATIVE\_REGULATION\_OF\_ADENYLATE\_CYCLASE\_ACTIVATING\_G\_PROTEIN\_COUPLED\_RECEPTOR\_SIGNALING\_PATHWAY |  | 4 | 0.49 | 0.99 | 0.437 | 0.816 | 1.000 | 6109 | tags=100%, list=51%, signal=203% |
| 1054 | GOBP\_CYTOPLASMIC\_SEQUESTERING\_OF\_TRANSCRIPTION\_FACTOR |  | 12 | 0.33 | 0.99 | 0.466 | 0.820 | 1.000 | 2532 | tags=50%, list=21%, signal=63% |
| 1055 | GOBP\_POSITIVE\_REGULATION\_OF\_ATP\_BIOSYNTHETIC\_PROCESS |  | 8 | 0.38 | 0.98 | 0.460 | 0.823 | 1.000 | 577 | tags=25%, list=5%, signal=26% |
| 1056 | GOCC\_9PLUS2\_MOTILE\_CILIUM |  | 44 | 0.23 | 0.98 | 0.470 | 0.823 | 1.000 | 866 | tags=20%, list=7%, signal=22% |
| 1057 | GOMF\_ENDOPEPTIDASE\_ACTIVATOR\_ACTIVITY |  | 6 | 0.42 | 0.98 | 0.446 | 0.824 | 1.000 | 6922 | tags=100%, list=58%, signal=236% |
| 1058 | GOMF\_POTASSIUM\_CHLORIDE\_SYMPORTER\_ACTIVITY |  | 6 | 0.42 | 0.98 | 0.457 | 0.824 | 1.000 | 1482 | tags=50%, list=12%, signal=57% |
| 1059 | GOBP\_SNO\_S\_RNA\_METABOLIC\_PROCESS |  | 10 | 0.35 | 0.98 | 0.450 | 0.824 | 1.000 | 730 | tags=20%, list=6%, signal=21% |
| 1060 | GOBP\_COENZYME\_A\_BIOSYNTHETIC\_PROCESS |  | 9 | 0.36 | 0.98 | 0.440 | 0.824 | 1.000 | 1796 | tags=56%, list=15%, signal=65% |
| 1061 | GOBP\_MODULATION\_BY\_SYMBIONT\_OF\_HOST\_PROGRAMMED\_CELL\_DEATH |  | 6 | 0.41 | 0.98 | 0.454 | 0.823 | 1.000 | 568 | tags=33%, list=5%, signal=35% |
| 1062 | GOBP\_NEGATIVE\_REGULATION\_OF\_LEUKOCYTE\_ADHESION\_TO\_VASCULAR\_ENDOTHELIAL\_CELL |  | 3 | 0.55 | 0.98 | 0.457 | 0.823 | 1.000 | 2640 | tags=67%, list=22%, signal=85% |
| 1063 | GOBP\_PYRIMIDINE\_RIBONUCLEOSIDE\_METABOLIC\_PROCESS |  | 6 | 0.42 | 0.98 | 0.456 | 0.822 | 1.000 | 6983 | tags=100%, list=58%, signal=239% |
| 1064 | GOBP\_WNT\_SIGNALING\_PATHWAY\_INVOLVED\_IN\_HEART\_DEVELOPMENT |  | 5 | 0.45 | 0.98 | 0.460 | 0.822 | 1.000 | 1104 | tags=40%, list=9%, signal=44% |
| 1065 | GOCC\_CORNIFIED\_ENVELOPE |  | 12 | 0.33 | 0.98 | 0.460 | 0.821 | 1.000 | 693 | tags=25%, list=6%, signal=27% |
| 1066 | GOBP\_NUCLEOSIDE\_BISPHOSPHATE\_CATABOLIC\_PROCESS |  | 4 | 0.49 | 0.98 | 0.458 | 0.821 | 1.000 | 1796 | tags=50%, list=15%, signal=59% |
| 1067 | GOBP\_RESPONSE\_TO\_DSRNA |  | 23 | 0.27 | 0.98 | 0.473 | 0.822 | 1.000 | 159 | tags=9%, list=1%, signal=9% |
| 1068 | GOBP\_DICARBOXYLIC\_ACID\_CATABOLIC\_PROCESS |  | 12 | 0.33 | 0.98 | 0.455 | 0.821 | 1.000 | 673 | tags=17%, list=6%, signal=18% |
| 1069 | GOBP\_MYOSIN\_FILAMENT\_ORGANIZATION |  | 3 | 0.54 | 0.98 | 0.453 | 0.822 | 1.000 | 5574 | tags=100%, list=46%, signal=187% |
| 1070 | GOBP\_CEREBROSPINAL\_FLUID\_CIRCULATION |  | 6 | 0.41 | 0.98 | 0.470 | 0.822 | 1.000 | 1143 | tags=33%, list=10%, signal=37% |
| 1071 | GOBP\_NEGATIVE\_REGULATION\_OF\_DENDRITIC\_SPINE\_MORPHOGENESIS |  | 3 | 0.55 | 0.98 | 0.463 | 0.823 | 1.000 | 386 | tags=33%, list=3%, signal=34% |
| 1072 | GOBP\_PHOSPHATIDYLGLYCEROL\_METABOLIC\_PROCESS |  | 24 | 0.27 | 0.98 | 0.476 | 0.823 | 1.000 | 299 | tags=13%, list=2%, signal=13% |
| 1073 | GOCC\_CHITOSOME |  | 5 | 0.45 | 0.98 | 0.451 | 0.822 | 1.000 | 384 | tags=20%, list=3%, signal=21% |
| 1074 | GOBP\_TRICARBOXYLIC\_ACID\_METABOLIC\_PROCESS |  | 11 | 0.34 | 0.98 | 0.452 | 0.822 | 1.000 | 7973 | tags=100%, list=66%, signal=297% |
| 1075 | GOBP\_UTP\_METABOLIC\_PROCESS |  | 8 | 0.38 | 0.98 | 0.463 | 0.825 | 1.000 | 1836 | tags=38%, list=15%, signal=44% |
| 1076 | GOBP\_POSITIVE\_REGULATION\_OF\_BLOOD\_BRAIN\_BARRIER\_PERMEABILITY |  | 5 | 0.44 | 0.98 | 0.457 | 0.825 | 1.000 | 954 | tags=40%, list=8%, signal=43% |
| 1077 | GOBP\_REGULATION\_OF\_EXCITATORY\_SYNAPSE\_ASSEMBLY |  | 6 | 0.42 | 0.98 | 0.461 | 0.825 | 1.000 | 220 | tags=17%, list=2%, signal=17% |
| 1078 | GOMF\_OLIGOPEPTIDE\_BINDING |  | 9 | 0.36 | 0.98 | 0.477 | 0.827 | 1.000 | 67 | tags=11%, list=1%, signal=11% |
| 1079 | GOBP\_NEGATIVE\_REGULATION\_OF\_PROTEOLYSIS\_INVOLVED\_IN\_CELLULAR\_PROTEIN\_CATABOLIC\_PROCESS |  | 47 | 0.23 | 0.98 | 0.509 | 0.827 | 1.000 | 1531 | tags=19%, list=13%, signal=22% |
| 1080 | GOBP\_POSITIVE\_REGULATION\_OF\_RESPONSE\_TO\_REACTIVE\_OXYGEN\_SPECIES |  | 3 | 0.54 | 0.98 | 0.468 | 0.827 | 1.000 | 5514 | tags=100%, list=46%, signal=185% |
| 1081 | GOCC\_ENDOPLASMIC\_RETICULUM\_PALMITOYLTRANSFERASE\_COMPLEX |  | 7 | 0.39 | 0.98 | 0.447 | 0.826 | 1.000 | 462 | tags=29%, list=4%, signal=30% |
| 1082 | GOBP\_REGULATION\_OF\_PROTON\_TRANSPORT |  | 14 | 0.31 | 0.98 | 0.463 | 0.826 | 1.000 | 8302 | tags=100%, list=69%, signal=323% |
| 1083 | GOBP\_REGULATION\_OF\_NAD\_P\_H\_OXIDASE\_ACTIVITY |  | 7 | 0.40 | 0.97 | 0.458 | 0.826 | 1.000 | 1459 | tags=43%, list=12%, signal=49% |
| 1084 | GOBP\_REGULATION\_OF\_DENDRITIC\_CELL\_CHEMOTAXIS |  | 6 | 0.42 | 0.97 | 0.458 | 0.826 | 1.000 | 1306 | tags=33%, list=11%, signal=37% |
| 1085 | GOBP\_POSITIVE\_REGULATION\_OF\_ACUTE\_INFLAMMATORY\_RESPONSE |  | 17 | 0.29 | 0.97 | 0.466 | 0.826 | 1.000 | 1237 | tags=18%, list=10%, signal=20% |
| 1086 | GOBP\_REGULATION\_OF\_COLLAGEN\_METABOLIC\_PROCESS |  | 29 | 0.25 | 0.97 | 0.503 | 0.827 | 1.000 | 1159 | tags=24%, list=10%, signal=27% |
| 1087 | GOBP\_POSITIVE\_REGULATION\_OF\_TOLL\_LIKE\_RECEPTOR\_9\_SIGNALING\_PATHWAY |  | 5 | 0.44 | 0.97 | 0.458 | 0.827 | 1.000 | 1070 | tags=40%, list=9%, signal=44% |
| 1088 | GOBP\_RESPONSE\_TO\_CATECHOLAMINE |  | 59 | 0.21 | 0.97 | 0.530 | 0.826 | 1.000 | 670 | tags=14%, list=6%, signal=14% |
| 1089 | GOMF\_C\_ACYLTRANSFERASE\_ACTIVITY |  | 11 | 0.33 | 0.97 | 0.474 | 0.827 | 1.000 | 1648 | tags=36%, list=14%, signal=42% |
| 1090 | GOBP\_POSITIVE\_REGULATION\_OF\_VASCULAR\_ASSOCIATED\_SMOOTH\_MUSCLE\_CELL\_APOPTOTIC\_PROCESS |  | 7 | 0.39 | 0.97 | 0.478 | 0.828 | 1.000 | 989 | tags=43%, list=8%, signal=47% |
| 1091 | GOBP\_NEUROTRANSMITTER\_RECEPTOR\_INTERNALIZATION |  | 11 | 0.33 | 0.97 | 0.467 | 0.827 | 1.000 | 16 | tags=9%, list=0%, signal=9% |
| 1092 | GOBP\_REGULATION\_OF\_SECONDARY\_HEART\_FIELD\_CARDIOBLAST\_PROLIFERATION |  | 4 | 0.48 | 0.97 | 0.453 | 0.828 | 1.000 | 1084 | tags=25%, list=9%, signal=27% |
| 1093 | GOBP\_ALDITOL\_METABOLIC\_PROCESS |  | 19 | 0.28 | 0.97 | 0.504 | 0.828 | 1.000 | 691 | tags=21%, list=6%, signal=22% |
| 1094 | GOCC\_POLYSOMAL\_RIBOSOME |  | 28 | 0.25 | 0.97 | 0.493 | 0.827 | 1.000 | 1834 | tags=25%, list=15%, signal=29% |
| 1095 | GOBP\_CARDIOBLAST\_PROLIFERATION |  | 5 | 0.44 | 0.97 | 0.460 | 0.829 | 1.000 | 6696 | tags=100%, list=56%, signal=226% |
| 1096 | GOBP\_NEGATIVE\_REGULATION\_OF\_RESPONSE\_TO\_INTERFERON\_GAMMA |  | 4 | 0.48 | 0.97 | 0.474 | 0.829 | 1.000 | 83 | tags=25%, list=1%, signal=25% |
| 1097 | GOBP\_ANTRAL\_OVARIAN\_FOLLICLE\_GROWTH |  | 5 | 0.44 | 0.97 | 0.480 | 0.829 | 1.000 | 33 | tags=20%, list=0%, signal=20% |
| 1098 | GOCC\_INTRINSIC\_COMPONENT\_OF\_MITOCHONDRIAL\_OUTER\_MEMBRANE |  | 16 | 0.29 | 0.97 | 0.493 | 0.829 | 1.000 | 1266 | tags=31%, list=11%, signal=35% |
| 1099 | GOBP\_HIGH\_DENSITY\_LIPOPROTEIN\_PARTICLE\_REMODELING |  | 9 | 0.36 | 0.97 | 0.468 | 0.828 | 1.000 | 7662 | tags=100%, list=64%, signal=276% |
| 1100 | GOMF\_TRANSFERASE\_ACTIVITY\_TRANSFERRING\_NITROGENOUS\_GROUPS |  | 16 | 0.30 | 0.97 | 0.486 | 0.828 | 1.000 | 1348 | tags=31%, list=11%, signal=35% |
| 1101 | GOBP\_POSITIVE\_REGULATION\_OF\_DEFENSE\_RESPONSE\_TO\_BACTERIUM |  | 3 | 0.54 | 0.97 | 0.476 | 0.828 | 1.000 | 1413 | tags=67%, list=12%, signal=76% |
| 1102 | GOBP\_NEURON\_FATE\_SPECIFICATION |  | 12 | 0.32 | 0.97 | 0.480 | 0.828 | 1.000 | 2001 | tags=50%, list=17%, signal=60% |
| 1103 | GOBP\_REGULATION\_OF\_RIG\_I\_SIGNALING\_PATHWAY |  | 12 | 0.32 | 0.97 | 0.489 | 0.828 | 1.000 | 332 | tags=17%, list=3%, signal=17% |
| 1104 | GOBP\_FUCOSYLATION |  | 11 | 0.33 | 0.97 | 0.476 | 0.828 | 1.000 | 507 | tags=18%, list=4%, signal=19% |
| 1105 | GOMF\_RETINOL\_DEHYDROGENASE\_ACTIVITY |  | 10 | 0.34 | 0.97 | 0.463 | 0.829 | 1.000 | 914 | tags=20%, list=8%, signal=22% |
| 1106 | GOBP\_ACROSOME\_ASSEMBLY |  | 6 | 0.42 | 0.97 | 0.474 | 0.828 | 1.000 | 7006 | tags=100%, list=58%, signal=240% |
| 1107 | GOBP\_GAMMA\_AMINOBUTYRIC\_ACID\_SECRETION |  | 5 | 0.44 | 0.97 | 0.465 | 0.830 | 1.000 | 550 | tags=40%, list=5%, signal=42% |
| 1108 | GOBP\_UDP\_N\_ACETYLGLUCOSAMINE\_BIOSYNTHETIC\_PROCESS |  | 9 | 0.35 | 0.97 | 0.470 | 0.831 | 1.000 | 583 | tags=33%, list=5%, signal=35% |
| 1109 | GOBP\_REGULATION\_OF\_TYPE\_B\_PANCREATIC\_CELL\_PROLIFERATION |  | 8 | 0.37 | 0.97 | 0.478 | 0.830 | 1.000 | 892 | tags=38%, list=7%, signal=40% |
| 1110 | GOBP\_NEGATIVE\_REGULATION\_OF\_VIRAL\_INDUCED\_CYTOPLASMIC\_PATTERN\_RECOGNITION\_RECEPTOR\_SIGNALING\_PATHWAY |  | 9 | 0.36 | 0.97 | 0.474 | 0.830 | 1.000 | 1306 | tags=22%, list=11%, signal=25% |
| 1111 | GOMF\_ALPHA\_1\_3\_FUCOSYLTRANSFERASE\_ACTIVITY |  | 6 | 0.41 | 0.96 | 0.481 | 0.831 | 1.000 | 49 | tags=17%, list=0%, signal=17% |
| 1112 | GOBP\_HEME\_METABOLIC\_PROCESS |  | 26 | 0.25 | 0.96 | 0.509 | 0.831 | 1.000 | 1927 | tags=27%, list=16%, signal=32% |
| 1113 | GOMF\_MITOCHONDRION\_TARGETING\_SEQUENCE\_BINDING |  | 4 | 0.48 | 0.96 | 0.481 | 0.831 | 1.000 | 2030 | tags=75%, list=17%, signal=90% |
| 1114 | GOCC\_G\_PROTEIN\_BETA\_GAMMA\_SUBUNIT\_COMPLEX |  | 6 | 0.41 | 0.96 | 0.490 | 0.831 | 1.000 | 1824 | tags=50%, list=15%, signal=59% |
| 1115 | GOBP\_ENZYME\_DIRECTED\_RRNA\_PSEUDOURIDINE\_SYNTHESIS |  | 4 | 0.48 | 0.96 | 0.490 | 0.833 | 1.000 | 6258 | tags=100%, list=52%, signal=209% |
| 1116 | GOBP\_SCARNA\_LOCALIZATION\_TO\_CAJAL\_BODY |  | 4 | 0.48 | 0.96 | 0.482 | 0.832 | 1.000 | 6258 | tags=100%, list=52%, signal=209% |
| 1117 | GOBP\_NEGATIVE\_REGULATION\_OF\_GLUCOCORTICOID\_METABOLIC\_PROCESS |  | 4 | 0.48 | 0.96 | 0.492 | 0.832 | 1.000 | 134 | tags=25%, list=1%, signal=25% |
| 1118 | GOBP\_REGULATION\_OF\_VIRAL\_INDUCED\_CYTOPLASMIC\_PATTERN\_RECOGNITION\_RECEPTOR\_SIGNALING\_PATHWAY |  | 15 | 0.29 | 0.96 | 0.496 | 0.832 | 1.000 | 332 | tags=13%, list=3%, signal=14% |
| 1119 | GOMF\_1\_ACYL\_2\_LYSOPHOSPHATIDYLSERINE\_ACYLHYDROLASE\_ACTIVITY |  | 4 | 0.48 | 0.96 | 0.462 | 0.833 | 1.000 | 140 | tags=25%, list=1%, signal=25% |
| 1120 | GOBP\_PORPHYRIN\_CONTAINING\_COMPOUND\_METABOLIC\_PROCESS |  | 30 | 0.24 | 0.96 | 0.503 | 0.832 | 1.000 | 1927 | tags=27%, list=16%, signal=32% |
| 1121 | GOBP\_MULTIVESICULAR\_BODY\_ORGANIZATION |  | 29 | 0.25 | 0.96 | 0.518 | 0.834 | 1.000 | 2888 | tags=55%, list=24%, signal=72% |
| 1122 | GOBP\_NEGATIVE\_REGULATION\_OF\_MICROGLIAL\_CELL\_ACTIVATION |  | 7 | 0.38 | 0.96 | 0.488 | 0.835 | 1.000 | 892 | tags=29%, list=7%, signal=31% |
| 1123 | GOMF\_NAD\_BINDING |  | 40 | 0.23 | 0.96 | 0.518 | 0.834 | 1.000 | 1614 | tags=25%, list=13%, signal=29% |
| 1124 | GOBP\_VITAMIN\_B6\_METABOLIC\_PROCESS |  | 5 | 0.44 | 0.96 | 0.482 | 0.835 | 1.000 | 1348 | tags=40%, list=11%, signal=45% |
| 1125 | GOBP\_RIG\_I\_SIGNALING\_PATHWAY |  | 15 | 0.29 | 0.96 | 0.505 | 0.838 | 1.000 | 332 | tags=13%, list=3%, signal=14% |
| 1126 | GOMF\_OXALATE\_TRANSMEMBRANE\_TRANSPORTER\_ACTIVITY |  | 6 | 0.41 | 0.96 | 0.472 | 0.839 | 1.000 | 452 | tags=33%, list=4%, signal=35% |
| 1127 | GOBP\_RENAL\_ABSORPTION |  | 11 | 0.33 | 0.96 | 0.476 | 0.839 | 1.000 | 1463 | tags=27%, list=12%, signal=31% |
| 1128 | GOCC\_ESCRT\_III\_COMPLEX |  | 9 | 0.35 | 0.96 | 0.494 | 0.839 | 1.000 | 2888 | tags=67%, list=24%, signal=88% |
| 1129 | GOBP\_SYNAPTIC\_VESICLE\_MATURATION |  | 11 | 0.33 | 0.96 | 0.485 | 0.839 | 1.000 | 403 | tags=18%, list=3%, signal=19% |
| 1130 | GOBP\_REGULATION\_OF\_LEUKOCYTE\_TETHERING\_OR\_ROLLING |  | 8 | 0.37 | 0.95 | 0.487 | 0.841 | 1.000 | 1235 | tags=25%, list=10%, signal=28% |
| 1131 | GOBP\_L\_ALPHA\_AMINO\_ACID\_TRANSMEMBRANE\_TRANSPORT |  | 36 | 0.23 | 0.95 | 0.572 | 0.842 | 1.000 | 1443 | tags=22%, list=12%, signal=25% |
| 1132 | GOBP\_INTEGRIN\_BIOSYNTHETIC\_PROCESS |  | 5 | 0.44 | 0.95 | 0.478 | 0.842 | 1.000 | 2089 | tags=60%, list=17%, signal=73% |
| 1133 | GOBP\_NUCLEOSIDE\_PHOSPHATE\_BIOSYNTHETIC\_PROCESS |  | 170 | 0.18 | 0.95 | 0.655 | 0.842 | 1.000 | 1267 | tags=16%, list=11%, signal=18% |
| 1134 | GOBP\_ENTEROENDOCRINE\_CELL\_DIFFERENTIATION |  | 22 | 0.26 | 0.95 | 0.522 | 0.842 | 1.000 | 1084 | tags=23%, list=9%, signal=25% |
| 1135 | GOMF\_UBIQUITIN\_LIGASE\_INHIBITOR\_ACTIVITY |  | 5 | 0.44 | 0.95 | 0.474 | 0.841 | 1.000 | 6762 | tags=100%, list=56%, signal=229% |
| 1136 | GOBP\_PROSTATE\_GLANDULAR\_ACINUS\_MORPHOGENESIS |  | 5 | 0.44 | 0.95 | 0.498 | 0.842 | 1.000 | 8 | tags=20%, list=0%, signal=20% |
| 1137 | GOBP\_PARACRINE\_SIGNALING |  | 4 | 0.47 | 0.95 | 0.491 | 0.843 | 1.000 | 449 | tags=25%, list=4%, signal=26% |
| 1138 | GOBP\_RECEPTOR\_RECYCLING |  | 24 | 0.26 | 0.95 | 0.534 | 0.843 | 1.000 | 1073 | tags=21%, list=9%, signal=23% |
| 1139 | GOBP\_POSITIVE\_REGULATION\_OF\_HIGH\_VOLTAGE\_GATED\_CALCIUM\_CHANNEL\_ACTIVITY |  | 3 | 0.53 | 0.95 | 0.499 | 0.844 | 1.000 | 5689 | tags=100%, list=47%, signal=190% |
| 1140 | GOBP\_TELENCEPHALON\_REGIONALIZATION |  | 5 | 0.44 | 0.95 | 0.498 | 0.845 | 1.000 | 134 | tags=20%, list=1%, signal=20% |
| 1141 | GOBP\_FATTY\_ACID\_CATABOLIC\_PROCESS |  | 86 | 0.19 | 0.95 | 0.582 | 0.845 | 1.000 | 1530 | tags=20%, list=13%, signal=22% |
| 1142 | GOBP\_REGULATION\_OF\_TRANSLATIONAL\_ELONGATION |  | 15 | 0.29 | 0.95 | 0.531 | 0.847 | 1.000 | 8485 | tags=100%, list=71%, signal=340% |
| 1143 | GOBP\_CARBON\_DIOXIDE\_TRANSPORT |  | 4 | 0.47 | 0.95 | 0.499 | 0.847 | 1.000 | 14 | tags=25%, list=0%, signal=25% |
| 1144 | GOBP\_RESPIRATORY\_BURST\_INVOLVED\_IN\_INFLAMMATORY\_RESPONSE |  | 5 | 0.43 | 0.95 | 0.510 | 0.847 | 1.000 | 1540 | tags=60%, list=13%, signal=69% |
| 1145 | GOBP\_CELLULAR\_MONOVALENT\_INORGANIC\_ANION\_HOMEOSTASIS |  | 5 | 0.43 | 0.95 | 0.489 | 0.847 | 1.000 | 1482 | tags=40%, list=12%, signal=46% |
| 1146 | GOBP\_LIPID\_OXIDATION |  | 88 | 0.19 | 0.95 | 0.603 | 0.848 | 1.000 | 2156 | tags=27%, list=18%, signal=33% |
| 1147 | GOBP\_PIGMENT\_METABOLIC\_PROCESS |  | 47 | 0.22 | 0.95 | 0.568 | 0.850 | 1.000 | 2059 | tags=26%, list=17%, signal=31% |
| 1148 | GOBP\_REGULATION\_OF\_MITOCHONDRIAL\_RNA\_CATABOLIC\_PROCESS |  | 5 | 0.43 | 0.95 | 0.495 | 0.850 | 1.000 | 6848 | tags=100%, list=57%, signal=233% |
| 1149 | GOBP\_ADP\_BIOSYNTHETIC\_PROCESS |  | 4 | 0.47 | 0.95 | 0.493 | 0.849 | 1.000 | 1197 | tags=50%, list=10%, signal=56% |
| 1150 | GOBP\_RESPIRATORY\_BURST |  | 26 | 0.25 | 0.95 | 0.558 | 0.849 | 1.000 | 1540 | tags=35%, list=13%, signal=40% |
| 1151 | GOBP\_MITOCHONDRIAL\_RNA\_3\_END\_PROCESSING |  | 5 | 0.43 | 0.95 | 0.499 | 0.850 | 1.000 | 6848 | tags=100%, list=57%, signal=233% |
| 1152 | GOMF\_3\_KETO\_STEROL\_REDUCTASE\_ACTIVITY |  | 4 | 0.48 | 0.94 | 0.505 | 0.850 | 1.000 | 6288 | tags=100%, list=52%, signal=210% |
| 1153 | GOBP\_DYNAMIN\_FAMILY\_PROTEIN\_POLYMERIZATION\_INVOLVED\_IN\_MEMBRANE\_FISSION |  | 5 | 0.43 | 0.94 | 0.501 | 0.853 | 1.000 | 16 | tags=20%, list=0%, signal=20% |
| 1154 | GOBP\_SRP\_DEPENDENT\_COTRANSLATIONAL\_PROTEIN\_TARGETING\_TO\_MEMBRANE\_TRANSLOCATION |  | 6 | 0.40 | 0.94 | 0.505 | 0.854 | 1.000 | 410 | tags=17%, list=3%, signal=17% |
| 1155 | GOCC\_RAGULATOR\_COMPLEX |  | 5 | 0.43 | 0.94 | 0.506 | 0.855 | 1.000 | 1702 | tags=60%, list=14%, signal=70% |
| 1156 | GOBP\_DEFENSE\_RESPONSE\_TO\_FUNGUS |  | 8 | 0.36 | 0.94 | 0.507 | 0.855 | 1.000 | 423 | tags=25%, list=4%, signal=26% |
| 1157 | GOBP\_PHOSPHATIDYLGLYCEROL\_ACYL\_CHAIN\_REMODELING |  | 9 | 0.34 | 0.94 | 0.498 | 0.857 | 1.000 | 299 | tags=22%, list=2%, signal=23% |
| 1158 | GOBP\_NEGATIVE\_REGULATION\_OF\_PROTEASOMAL\_PROTEIN\_CATABOLIC\_PROCESS |  | 36 | 0.23 | 0.94 | 0.582 | 0.856 | 1.000 | 1531 | tags=19%, list=13%, signal=22% |
| 1159 | GOMF\_VOLTAGE\_GATED\_POTASSIUM\_CHANNEL\_ACTIVITY\_INVOLVED\_IN\_CARDIAC\_MUSCLE\_CELL\_ACTION\_POTENTIAL\_REPOLARIZATION |  | 8 | 0.36 | 0.94 | 0.498 | 0.857 | 1.000 | 7665 | tags=100%, list=64%, signal=276% |
| 1160 | GOCC\_POSTSYNAPTIC\_ENDOCYTIC\_ZONE |  | 5 | 0.43 | 0.94 | 0.505 | 0.856 | 1.000 | 16 | tags=20%, list=0%, signal=20% |
| 1161 | GOMF\_SODIUM\_CHLORIDE\_SYMPORTER\_ACTIVITY |  | 7 | 0.38 | 0.94 | 0.513 | 0.855 | 1.000 | 1482 | tags=43%, list=12%, signal=49% |
| 1162 | GOBP\_PYRUVATE\_TRANSMEMBRANE\_TRANSPORT |  | 3 | 0.52 | 0.94 | 0.498 | 0.855 | 1.000 | 407 | tags=33%, list=3%, signal=34% |
| 1163 | GOBP\_RNA\_PHOSPHODIESTER\_BOND\_HYDROLYSIS\_ENDONUCLEOLYTIC |  | 50 | 0.21 | 0.94 | 0.578 | 0.856 | 1.000 | 1054 | tags=18%, list=9%, signal=20% |
| 1164 | GOBP\_NEGATIVE\_REGULATION\_OF\_T\_CELL\_DIFFERENTIATION\_IN\_THYMUS |  | 5 | 0.43 | 0.94 | 0.510 | 0.855 | 1.000 | 1723 | tags=40%, list=14%, signal=47% |
| 1165 | GOCC\_MOTILE\_CILIUM |  | 80 | 0.20 | 0.94 | 0.629 | 0.855 | 1.000 | 1143 | tags=18%, list=10%, signal=19% |
| 1166 | GOBP\_VENTRICULAR\_SYSTEM\_DEVELOPMENT |  | 19 | 0.27 | 0.94 | 0.540 | 0.854 | 1.000 | 1064 | tags=26%, list=9%, signal=29% |
| 1167 | GOMF\_4\_GALACTOSYL\_N\_ACETYLGLUCOSAMINIDE\_3\_ALPHA\_L\_FUCOSYLTRANSFERASE\_ACTIVITY |  | 4 | 0.47 | 0.94 | 0.512 | 0.854 | 1.000 | 49 | tags=25%, list=0%, signal=25% |
| 1168 | GOMF\_ENOYL\_COA\_HYDRATASE\_ACTIVITY |  | 9 | 0.35 | 0.94 | 0.522 | 0.853 | 1.000 | 1648 | tags=44%, list=14%, signal=51% |
| 1169 | GOMF\_GUANOSINE\_DIPHOSPHATASE\_ACTIVITY |  | 9 | 0.34 | 0.94 | 0.511 | 0.854 | 1.000 | 7894 | tags=100%, list=66%, signal=292% |
| 1170 | GOCC\_DENDRITE\_MEMBRANE |  | 16 | 0.28 | 0.94 | 0.547 | 0.854 | 1.000 | 1410 | tags=25%, list=12%, signal=28% |
| 1171 | GOBP\_GLANDULAR\_EPITHELIAL\_CELL\_DIFFERENTIATION |  | 37 | 0.23 | 0.94 | 0.577 | 0.855 | 1.000 | 1084 | tags=19%, list=9%, signal=21% |
| 1172 | GOBP\_REGULATION\_OF\_MESENCHYMAL\_CELL\_PROLIFERATION |  | 20 | 0.26 | 0.94 | 0.551 | 0.856 | 1.000 | 1754 | tags=25%, list=15%, signal=29% |
| 1173 | GOBP\_ACETYL\_COA\_BIOSYNTHETIC\_PROCESS |  | 19 | 0.27 | 0.94 | 0.553 | 0.856 | 1.000 | 1150 | tags=26%, list=10%, signal=29% |
| 1174 | GOCC\_RNA\_POLYMERASE\_I\_COMPLEX |  | 10 | 0.33 | 0.94 | 0.509 | 0.856 | 1.000 | 2334 | tags=50%, list=19%, signal=62% |
| 1175 | GOBP\_POSITIVE\_REGULATION\_OF\_ENDOTHELIAL\_CELL\_DIFFERENTIATION |  | 8 | 0.35 | 0.94 | 0.515 | 0.856 | 1.000 | 1723 | tags=38%, list=14%, signal=44% |
| 1176 | GOBP\_SINGLE\_FERTILIZATION |  | 61 | 0.20 | 0.93 | 0.623 | 0.859 | 1.000 | 1538 | tags=21%, list=13%, signal=24% |
| 1177 | GOBP\_GLIAL\_CELL\_DERIVED\_NEUROTROPHIC\_FACTOR\_RECEPTOR\_SIGNALING\_PATHWAY |  | 9 | 0.35 | 0.93 | 0.503 | 0.858 | 1.000 | 525 | tags=22%, list=4%, signal=23% |
| 1178 | GOBP\_POSITIVE\_REGULATION\_OF\_EPITHELIAL\_CELL\_PROLIFERATION\_INVOLVED\_IN\_LUNG\_MORPHOGENESIS |  | 4 | 0.47 | 0.93 | 0.522 | 0.859 | 1.000 | 6390 | tags=100%, list=53%, signal=214% |
| 1179 | GOCC\_PERINUCLEOLAR\_COMPARTMENT |  | 3 | 0.51 | 0.93 | 0.517 | 0.859 | 1.000 | 1219 | tags=67%, list=10%, signal=74% |
| 1180 | GOBP\_INTRINSIC\_APOPTOTIC\_SIGNALING\_PATHWAY\_IN\_RESPONSE\_TO\_OSMOTIC\_STRESS |  | 4 | 0.46 | 0.93 | 0.525 | 0.858 | 1.000 | 6448 | tags=100%, list=54%, signal=216% |
| 1181 | GOMF\_TRANSLATION\_REPRESSOR\_ACTIVITY |  | 18 | 0.27 | 0.93 | 0.558 | 0.860 | 1.000 | 192 | tags=11%, list=2%, signal=11% |
| 1182 | GOBP\_STABILIZATION\_OF\_MEMBRANE\_POTENTIAL |  | 8 | 0.35 | 0.93 | 0.537 | 0.860 | 1.000 | 925 | tags=25%, list=8%, signal=27% |
| 1183 | GOMF\_PYRUVATE\_TRANSMEMBRANE\_TRANSPORTER\_ACTIVITY |  | 3 | 0.52 | 0.93 | 0.525 | 0.860 | 1.000 | 407 | tags=33%, list=3%, signal=34% |
| 1184 | GOBP\_ADAPTATION\_OF\_SIGNALING\_PATHWAY |  | 11 | 0.32 | 0.93 | 0.523 | 0.860 | 1.000 | 1587 | tags=36%, list=13%, signal=42% |
| 1185 | GOBP\_NEUROTRANSMITTER\_UPTAKE |  | 25 | 0.25 | 0.93 | 0.573 | 0.862 | 1.000 | 969 | tags=16%, list=8%, signal=17% |
| 1186 | GOBP\_PYRIMIDINE\_RIBONUCLEOSIDE\_TRIPHOSPHATE\_BIOSYNTHETIC\_PROCESS |  | 11 | 0.32 | 0.93 | 0.528 | 0.862 | 1.000 | 1836 | tags=27%, list=15%, signal=32% |
| 1187 | GOBP\_SPERM\_EGG\_RECOGNITION |  | 21 | 0.26 | 0.93 | 0.568 | 0.862 | 1.000 | 1301 | tags=24%, list=11%, signal=27% |
| 1188 | GOBP\_PROTEIN\_MATURATION\_BY\_PROTEIN\_FOLDING |  | 8 | 0.35 | 0.93 | 0.523 | 0.866 | 1.000 | 2045 | tags=25%, list=17%, signal=30% |
| 1189 | GOMF\_PHOSPHATIDYLINOSITOL\_3\_5\_BISPHOSPHATE\_BINDING |  | 19 | 0.27 | 0.93 | 0.585 | 0.868 | 1.000 | 8800 | tags=100%, list=73%, signal=373% |
| 1190 | GOBP\_UDP\_N\_ACETYLGLUCOSAMINE\_METABOLIC\_PROCESS |  | 12 | 0.30 | 0.93 | 0.545 | 0.867 | 1.000 | 583 | tags=25%, list=5%, signal=26% |
| 1191 | GOMF\_ACETYL\_COA\_C\_ACETYLTRANSFERASE\_ACTIVITY |  | 5 | 0.42 | 0.93 | 0.519 | 0.869 | 1.000 | 1648 | tags=40%, list=14%, signal=46% |
| 1192 | GOBP\_MONOACYLGLYCEROL\_METABOLIC\_PROCESS |  | 8 | 0.35 | 0.92 | 0.516 | 0.870 | 1.000 | 703 | tags=25%, list=6%, signal=27% |
| 1193 | GOBP\_POSITIVE\_REGULATION\_OF\_FATTY\_ACID\_BIOSYNTHETIC\_PROCESS |  | 15 | 0.28 | 0.92 | 0.545 | 0.870 | 1.000 | 1377 | tags=27%, list=11%, signal=30% |
| 1194 | GOMF\_PHOSPHOTRANSFERASE\_ACTIVITY\_NITROGENOUS\_GROUP\_AS\_ACCEPTOR |  | 4 | 0.46 | 0.92 | 0.541 | 0.870 | 1.000 | 1836 | tags=50%, list=15%, signal=59% |
| 1195 | GOBP\_PURINE\_NUCLEOSIDE\_DIPHOSPHATE\_CATABOLIC\_PROCESS |  | 3 | 0.51 | 0.92 | 0.533 | 0.869 | 1.000 | 5868 | tags=100%, list=49%, signal=195% |
| 1196 | GOBP\_REGULATION\_OF\_PHOSPHOLIPID\_METABOLIC\_PROCESS |  | 28 | 0.24 | 0.92 | 0.589 | 0.869 | 1.000 | 2169 | tags=25%, list=18%, signal=30% |
| 1197 | GOBP\_GLYCEROPHOSPHOLIPID\_CATABOLIC\_PROCESS |  | 22 | 0.25 | 0.92 | 0.593 | 0.868 | 1.000 | 703 | tags=18%, list=6%, signal=19% |
| 1198 | GOBP\_POSITIVE\_REGULATION\_OF\_PRI\_MIRNA\_TRANSCRIPTION\_BY\_RNA\_POLYMERASE\_II |  | 32 | 0.23 | 0.92 | 0.594 | 0.870 | 1.000 | 558 | tags=13%, list=5%, signal=13% |
| 1199 | GOBP\_C21\_STEROID\_HORMONE\_BIOSYNTHETIC\_PROCESS |  | 18 | 0.27 | 0.92 | 0.572 | 0.870 | 1.000 | 1478 | tags=28%, list=12%, signal=32% |
| 1200 | GOMF\_CDP\_ALCOHOL\_PHOSPHATIDYLTRANSFERASE\_ACTIVITY |  | 7 | 0.37 | 0.92 | 0.543 | 0.870 | 1.000 | 2214 | tags=43%, list=18%, signal=53% |
| 1201 | GOMF\_NEUREXIN\_FAMILY\_PROTEIN\_BINDING |  | 8 | 0.35 | 0.92 | 0.555 | 0.869 | 1.000 | 1204 | tags=25%, list=10%, signal=28% |
| 1202 | GOCC\_MKS\_COMPLEX |  | 12 | 0.31 | 0.92 | 0.547 | 0.869 | 1.000 | 1705 | tags=33%, list=14%, signal=39% |
| 1203 | GOBP\_MITOTIC\_CELL\_CYCLE\_ARREST |  | 13 | 0.29 | 0.92 | 0.559 | 0.869 | 1.000 | 1069 | tags=23%, list=9%, signal=25% |
| 1204 | GOCC\_DENDRITIC\_SPINE\_MEMBRANE |  | 6 | 0.39 | 0.92 | 0.531 | 0.873 | 1.000 | 43 | tags=17%, list=0%, signal=17% |
| 1205 | GOBP\_MEMBRANE\_REPOLARIZATION\_DURING\_ATRIAL\_CARDIAC\_MUSCLE\_CELL\_ACTION\_POTENTIAL |  | 4 | 0.46 | 0.92 | 0.547 | 0.873 | 1.000 | 509 | tags=25%, list=4%, signal=26% |
| 1206 | GOBP\_REGULATION\_OF\_LYSOSOME\_ORGANIZATION |  | 4 | 0.46 | 0.92 | 0.536 | 0.872 | 1.000 | 405 | tags=25%, list=3%, signal=26% |
| 1207 | GOBP\_ANTIGEN\_PROCESSING\_AND\_PRESENTATION\_OF\_ENDOGENOUS\_PEPTIDE\_ANTIGEN |  | 6 | 0.39 | 0.92 | 0.518 | 0.872 | 1.000 | 806 | tags=33%, list=7%, signal=36% |
| 1208 | GOBP\_PURINE\_RIBONUCLEOSIDE\_SALVAGE |  | 5 | 0.42 | 0.92 | 0.531 | 0.872 | 1.000 | 108 | tags=20%, list=1%, signal=20% |
| 1209 | GOBP\_REGULATION\_OF\_LOW\_DENSITY\_LIPOPROTEIN\_PARTICLE\_CLEARANCE |  | 7 | 0.37 | 0.92 | 0.539 | 0.873 | 1.000 | 158 | tags=14%, list=1%, signal=14% |
| 1210 | GOMF\_ICOSATETRAENOIC\_ACID\_BINDING |  | 3 | 0.50 | 0.92 | 0.527 | 0.872 | 1.000 | 1003 | tags=67%, list=8%, signal=73% |
| 1211 | GOBP\_REGULATION\_OF\_EPITHELIAL\_CELL\_DIFFERENTIATION\_INVOLVED\_IN\_KIDNEY\_DEVELOPMENT |  | 11 | 0.32 | 0.92 | 0.555 | 0.872 | 1.000 | 569 | tags=27%, list=5%, signal=29% |
| 1212 | GOBP\_NEGATIVE\_REGULATION\_OF\_TRANSFORMING\_GROWTH\_FACTOR\_BETA1\_PRODUCTION |  | 6 | 0.39 | 0.92 | 0.537 | 0.871 | 1.000 | 1340 | tags=33%, list=11%, signal=38% |
| 1213 | GOBP\_REGULATION\_OF\_HEMATOPOIETIC\_STEM\_CELL\_DIFFERENTIATION |  | 58 | 0.20 | 0.92 | 0.663 | 0.872 | 1.000 | 3006 | tags=41%, list=25%, signal=55% |
| 1214 | GOBP\_PROTEASOME\_ASSEMBLY |  | 11 | 0.32 | 0.92 | 0.560 | 0.872 | 1.000 | 2315 | tags=36%, list=19%, signal=45% |
| 1215 | GOBP\_NEGATIVE\_REGULATION\_OF\_RECEPTOR\_BINDING |  | 7 | 0.37 | 0.92 | 0.565 | 0.872 | 1.000 | 158 | tags=14%, list=1%, signal=14% |
| 1216 | GOCC\_AZUROPHIL\_GRANULE\_MEMBRANE |  | 44 | 0.21 | 0.92 | 0.611 | 0.871 | 1.000 | 394 | tags=9%, list=3%, signal=9% |
| 1217 | GOBP\_REGULATION\_OF\_GROWTH\_HORMONE\_RECEPTOR\_SIGNALING\_PATHWAY |  | 4 | 0.45 | 0.92 | 0.531 | 0.871 | 1.000 | 95 | tags=25%, list=1%, signal=25% |
| 1218 | GOBP\_POSITIVE\_REGULATION\_OF\_FIBROBLAST\_PROLIFERATION |  | 38 | 0.22 | 0.92 | 0.611 | 0.871 | 1.000 | 1188 | tags=18%, list=10%, signal=20% |
| 1219 | GOBP\_REGULATION\_OF\_CHAPERONE\_MEDIATED\_PROTEIN\_FOLDING |  | 5 | 0.42 | 0.92 | 0.540 | 0.873 | 1.000 | 6976 | tags=100%, list=58%, signal=238% |
| 1220 | GOBP\_PROTEIN\_MONO\_ADP\_RIBOSYLATION |  | 9 | 0.34 | 0.91 | 0.543 | 0.874 | 1.000 | 1427 | tags=44%, list=12%, signal=50% |
| 1221 | GOBP\_RELEASE\_OF\_CYTOCHROME\_C\_FROM\_MITOCHONDRIA |  | 36 | 0.22 | 0.91 | 0.617 | 0.873 | 1.000 | 1821 | tags=28%, list=15%, signal=33% |
| 1222 | GOMF\_NUCLEOSOMAL\_DNA\_BINDING |  | 19 | 0.26 | 0.91 | 0.595 | 0.873 | 1.000 | 8875 | tags=100%, list=74%, signal=382% |
| 1223 | GOCC\_RNA\_POLYMERASE\_II\_TRANSCRIPTION\_REPRESSOR\_COMPLEX |  | 3 | 0.51 | 0.91 | 0.546 | 0.872 | 1.000 | 5911 | tags=100%, list=49%, signal=197% |
| 1224 | GOMF\_PRE\_MRNA\_3\_SPLICE\_SITE\_BINDING |  | 3 | 0.51 | 0.91 | 0.543 | 0.872 | 1.000 | 5887 | tags=100%, list=49%, signal=196% |
| 1225 | GOBP\_MRNA\_3\_SPLICE\_SITE\_RECOGNITION |  | 5 | 0.41 | 0.91 | 0.520 | 0.871 | 1.000 | 7060 | tags=100%, list=59%, signal=242% |
| 1226 | GOBP\_REGULATION\_OF\_VENTRICULAR\_CARDIAC\_MUSCLE\_CELL\_MEMBRANE\_REPOLARIZATION |  | 14 | 0.29 | 0.91 | 0.568 | 0.873 | 1.000 | 2179 | tags=36%, list=18%, signal=44% |
| 1227 | GOMF\_ATPASE\_ACTIVATOR\_ACTIVITY |  | 19 | 0.27 | 0.91 | 0.582 | 0.873 | 1.000 | 2312 | tags=37%, list=19%, signal=46% |
| 1228 | GOBP\_RESPONSE\_TO\_WATER |  | 8 | 0.35 | 0.91 | 0.550 | 0.873 | 1.000 | 535 | tags=25%, list=4%, signal=26% |
| 1229 | GOMF\_ACID\_AMINO\_ACID\_LIGASE\_ACTIVITY |  | 16 | 0.28 | 0.91 | 0.566 | 0.873 | 1.000 | 1388 | tags=25%, list=12%, signal=28% |
| 1230 | GOBP\_DETERMINATION\_OF\_DORSAL\_VENTRAL\_ASYMMETRY |  | 6 | 0.39 | 0.91 | 0.539 | 0.874 | 1.000 | 7388 | tags=100%, list=62%, signal=260% |
| 1231 | GOBP\_RESPONSE\_TO\_ARSENIC\_CONTAINING\_SUBSTANCE |  | 23 | 0.25 | 0.91 | 0.608 | 0.876 | 1.000 | 1249 | tags=26%, list=10%, signal=29% |
| 1232 | GOBP\_BRADYKININ\_CATABOLIC\_PROCESS |  | 5 | 0.42 | 0.91 | 0.544 | 0.878 | 1.000 | 221 | tags=20%, list=2%, signal=20% |
| 1233 | GOMF\_RETINOIC\_ACID\_4\_HYDROXYLASE\_ACTIVITY |  | 4 | 0.45 | 0.91 | 0.566 | 0.878 | 1.000 | 638 | tags=50%, list=5%, signal=53% |
| 1234 | GOBP\_REGULATION\_OF\_AMINO\_ACID\_IMPORT\_ACROSS\_PLASMA\_MEMBRANE |  | 9 | 0.34 | 0.91 | 0.563 | 0.878 | 1.000 | 804 | tags=22%, list=7%, signal=24% |
| 1235 | GOBP\_POSITIVE\_REGULATION\_OF\_SPHINGOLIPID\_BIOSYNTHETIC\_PROCESS |  | 7 | 0.37 | 0.91 | 0.558 | 0.878 | 1.000 | 7579 | tags=100%, list=63%, signal=271% |
| 1236 | GOBP\_RRNA\_CONTAINING\_RIBONUCLEOPROTEIN\_COMPLEX\_EXPORT\_FROM\_NUCLEUS |  | 13 | 0.30 | 0.91 | 0.567 | 0.879 | 1.000 | 262 | tags=8%, list=2%, signal=8% |
| 1237 | GOBP\_PHENOTYPIC\_SWITCHING |  | 6 | 0.38 | 0.91 | 0.536 | 0.880 | 1.000 | 7401 | tags=100%, list=62%, signal=260% |
| 1238 | GOBP\_SENSORY\_PERCEPTION\_OF\_TEMPERATURE\_STIMULUS |  | 13 | 0.29 | 0.91 | 0.577 | 0.881 | 1.000 | 1904 | tags=31%, list=16%, signal=37% |
| 1239 | GOMF\_SIALYLTRANSFERASE\_ACTIVITY |  | 17 | 0.27 | 0.91 | 0.582 | 0.881 | 1.000 | 1781 | tags=29%, list=15%, signal=34% |
| 1240 | GOBP\_RESPONSE\_TO\_THYROID\_HORMONE |  | 16 | 0.28 | 0.91 | 0.587 | 0.881 | 1.000 | 1858 | tags=38%, list=15%, signal=44% |
| 1241 | GOBP\_SPHINGOID\_METABOLIC\_PROCESS |  | 16 | 0.28 | 0.91 | 0.585 | 0.881 | 1.000 | 171 | tags=13%, list=1%, signal=13% |
| 1242 | GOBP\_SIALYLATION |  | 16 | 0.28 | 0.91 | 0.592 | 0.881 | 1.000 | 186 | tags=13%, list=2%, signal=13% |
| 1243 | GOBP\_RESPONSE\_TO\_UV\_B |  | 12 | 0.30 | 0.90 | 0.576 | 0.881 | 1.000 | 959 | tags=25%, list=8%, signal=27% |
| 1244 | GOMF\_NADP\_RETINOL\_DEHYDROGENASE\_ACTIVITY |  | 7 | 0.36 | 0.90 | 0.566 | 0.882 | 1.000 | 2781 | tags=57%, list=23%, signal=74% |
| 1245 | GOCC\_SIGNAL\_RECOGNITION\_PARTICLE |  | 7 | 0.36 | 0.90 | 0.565 | 0.883 | 1.000 | 7637 | tags=100%, list=64%, signal=274% |
| 1246 | GOMF\_VOLTAGE\_GATED\_CALCIUM\_CHANNEL\_ACTIVITY\_INVOLVED\_IN\_REGULATION\_OF\_CYTOSOLIC\_CALCIUM\_LEVELS |  | 6 | 0.39 | 0.90 | 0.557 | 0.882 | 1.000 | 1953 | tags=50%, list=16%, signal=60% |
| 1247 | GOBP\_REGULATION\_OF\_HYDROGEN\_PEROXIDE\_MEDIATED\_PROGRAMMED\_CELL\_DEATH |  | 6 | 0.38 | 0.90 | 0.556 | 0.882 | 1.000 | 7414 | tags=100%, list=62%, signal=261% |
| 1248 | GOCC\_BOX\_H\_ACA\_TELOMERASE\_RNP\_COMPLEX |  | 3 | 0.50 | 0.90 | 0.556 | 0.883 | 1.000 | 2425 | tags=67%, list=20%, signal=84% |
| 1249 | GOBP\_L\_ASCORBIC\_ACID\_METABOLIC\_PROCESS |  | 7 | 0.36 | 0.90 | 0.561 | 0.882 | 1.000 | 1464 | tags=57%, list=12%, signal=65% |
| 1250 | GOMF\_PROTEIN\_TYROSINE\_SERINE\_THREONINE\_PHOSPHATASE\_ACTIVITY |  | 32 | 0.23 | 0.90 | 0.645 | 0.882 | 1.000 | 1431 | tags=22%, list=12%, signal=25% |
| 1251 | GOMF\_RNA\_DNA\_HYBRID\_RIBONUCLEASE\_ACTIVITY |  | 3 | 0.50 | 0.90 | 0.560 | 0.884 | 1.000 | 6038 | tags=100%, list=50%, signal=201% |
| 1252 | GOBP\_CHAPERONE\_MEDIATED\_PROTEIN\_TRANSPORT |  | 7 | 0.36 | 0.90 | 0.573 | 0.883 | 1.000 | 2341 | tags=57%, list=19%, signal=71% |
| 1253 | GOBP\_PROTOPORPHYRINOGEN\_IX\_METABOLIC\_PROCESS |  | 9 | 0.33 | 0.90 | 0.588 | 0.883 | 1.000 | 249 | tags=11%, list=2%, signal=11% |
| 1254 | GOBP\_POSITIVE\_REGULATION\_OF\_OSTEOCLAST\_DEVELOPMENT |  | 3 | 0.50 | 0.90 | 0.557 | 0.883 | 1.000 | 21 | tags=33%, list=0%, signal=33% |
| 1255 | GOBP\_GAMMA\_AMINOBUTYRIC\_ACID\_TRANSPORT |  | 9 | 0.34 | 0.90 | 0.577 | 0.886 | 1.000 | 629 | tags=33%, list=5%, signal=35% |
| 1256 | GOBP\_REGULATION\_OF\_PROTEIN\_LOCALIZATION\_TO\_CHROMOSOME\_TELOMERIC\_REGION |  | 11 | 0.31 | 0.90 | 0.590 | 0.887 | 1.000 | 8290 | tags=100%, list=69%, signal=322% |
| 1257 | GOBP\_REGULATION\_OF\_BRANCHING\_INVOLVED\_IN\_LUNG\_MORPHOGENESIS |  | 3 | 0.50 | 0.90 | 0.567 | 0.886 | 1.000 | 6045 | tags=100%, list=50%, signal=201% |
| 1258 | GOBP\_PROTEIN\_ADP\_RIBOSYLATION |  | 23 | 0.25 | 0.90 | 0.614 | 0.888 | 1.000 | 1900 | tags=39%, list=16%, signal=46% |
| 1259 | GOBP\_ASPARTATE\_FAMILY\_AMINO\_ACID\_BIOSYNTHETIC\_PROCESS |  | 18 | 0.26 | 0.90 | 0.627 | 0.888 | 1.000 | 1092 | tags=22%, list=9%, signal=24% |
| 1260 | GOBP\_REGULATION\_OF\_CHAPERONE\_MEDIATED\_AUTOPHAGY |  | 6 | 0.38 | 0.90 | 0.567 | 0.889 | 1.000 | 7427 | tags=100%, list=62%, signal=262% |
| 1261 | GOBP\_G\_PROTEIN\_COUPLED\_RECEPTOR\_SIGNALING\_PATHWAY\_INVOLVED\_IN\_HEART\_PROCESS |  | 10 | 0.32 | 0.90 | 0.562 | 0.891 | 1.000 | 423 | tags=20%, list=4%, signal=21% |
| 1262 | GOBP\_REGULATION\_OF\_OVARIAN\_FOLLICLE\_DEVELOPMENT |  | 3 | 0.49 | 0.89 | 0.578 | 0.892 | 1.000 | 580 | tags=33%, list=5%, signal=35% |
| 1263 | GOMF\_NUCLEOTIDASE\_ACTIVITY |  | 11 | 0.31 | 0.89 | 0.605 | 0.892 | 1.000 | 6210 | tags=91%, list=52%, signal=188% |
| 1264 | GOCC\_TERTIARY\_GRANULE\_LUMEN |  | 33 | 0.22 | 0.89 | 0.655 | 0.892 | 1.000 | 1357 | tags=21%, list=11%, signal=24% |
| 1265 | GOBP\_VIRION\_ASSEMBLY |  | 35 | 0.22 | 0.89 | 0.650 | 0.892 | 1.000 | 3080 | tags=54%, list=26%, signal=73% |
| 1266 | GOBP\_RRNA\_BASE\_METHYLATION |  | 5 | 0.41 | 0.89 | 0.571 | 0.892 | 1.000 | 7087 | tags=100%, list=59%, signal=244% |
| 1267 | GOBP\_COP9\_SIGNALOSOME\_ASSEMBLY |  | 4 | 0.44 | 0.89 | 0.557 | 0.892 | 1.000 | 6726 | tags=100%, list=56%, signal=227% |
| 1268 | GOBP\_ANTERIOR\_HEAD\_DEVELOPMENT |  | 3 | 0.50 | 0.89 | 0.577 | 0.893 | 1.000 | 6045 | tags=100%, list=50%, signal=201% |
| 1269 | GOBP\_SERINE\_TRANSPORT |  | 7 | 0.36 | 0.89 | 0.569 | 0.892 | 1.000 | 1298 | tags=29%, list=11%, signal=32% |
| 1270 | GOBP\_POSITIVE\_REGULATION\_OF\_CHONDROCYTE\_PROLIFERATION |  | 3 | 0.50 | 0.89 | 0.574 | 0.892 | 1.000 | 6045 | tags=100%, list=50%, signal=201% |
| 1271 | GOBP\_TENDON\_DEVELOPMENT |  | 4 | 0.44 | 0.89 | 0.566 | 0.892 | 1.000 | 6688 | tags=100%, list=56%, signal=226% |
| 1272 | GOBP\_VASCULAR\_ENDOTHELIAL\_CELL\_PROLIFERATION |  | 15 | 0.28 | 0.89 | 0.608 | 0.893 | 1.000 | 558 | tags=13%, list=5%, signal=14% |
| 1273 | GOBP\_AMINO\_SUGAR\_BIOSYNTHETIC\_PROCESS |  | 11 | 0.31 | 0.89 | 0.596 | 0.893 | 1.000 | 583 | tags=27%, list=5%, signal=29% |
| 1274 | GOBP\_POSITIVE\_REGULATION\_OF\_ACUTE\_INFLAMMATORY\_RESPONSE\_TO\_ANTIGENIC\_STIMULUS |  | 5 | 0.41 | 0.89 | 0.580 | 0.895 | 1.000 | 7105 | tags=100%, list=59%, signal=245% |
| 1275 | GOCC\_IMMUNOGLOBULIN\_COMPLEX |  | 4 | 0.44 | 0.89 | 0.587 | 0.895 | 1.000 | 398 | tags=25%, list=3%, signal=26% |
| 1276 | GOBP\_POSITIVE\_REGULATION\_OF\_SEQUESTERING\_OF\_CALCIUM\_ION |  | 9 | 0.33 | 0.89 | 0.591 | 0.895 | 1.000 | 8103 | tags=100%, list=67%, signal=307% |
| 1277 | GOBP\_SMALL\_NUCLEOLAR\_RIBONUCLEOPROTEIN\_COMPLEX\_ASSEMBLY |  | 8 | 0.34 | 0.89 | 0.583 | 0.896 | 1.000 | 566 | tags=13%, list=5%, signal=13% |
| 1278 | GOMF\_CATECHOLAMINE\_BINDING |  | 8 | 0.34 | 0.89 | 0.583 | 0.895 | 1.000 | 2740 | tags=50%, list=23%, signal=65% |
| 1279 | GOBP\_ANDROGEN\_CATABOLIC\_PROCESS |  | 3 | 0.49 | 0.89 | 0.586 | 0.895 | 1.000 | 6131 | tags=100%, list=51%, signal=204% |
| 1280 | GOBP\_PURINE\_NUCLEOSIDE\_BIOSYNTHETIC\_PROCESS |  | 16 | 0.27 | 0.89 | 0.616 | 0.895 | 1.000 | 1093 | tags=19%, list=9%, signal=21% |
| 1281 | GOBP\_POLYAMINE\_METABOLIC\_PROCESS |  | 13 | 0.29 | 0.89 | 0.615 | 0.895 | 1.000 | 1035 | tags=23%, list=9%, signal=25% |
| 1282 | GOCC\_ROUGH\_ENDOPLASMIC\_RETICULUM |  | 52 | 0.20 | 0.89 | 0.711 | 0.896 | 1.000 | 960 | tags=13%, list=8%, signal=15% |
| 1283 | GOBP\_SULFUR\_COMPOUND\_CATABOLIC\_PROCESS |  | 37 | 0.22 | 0.89 | 0.680 | 0.896 | 1.000 | 1365 | tags=22%, list=11%, signal=24% |
| 1284 | GOBP\_RNA\_SURVEILLANCE |  | 13 | 0.28 | 0.89 | 0.619 | 0.897 | 1.000 | 730 | tags=15%, list=6%, signal=16% |
| 1285 | GOBP\_NEGATIVE\_REGULATION\_OF\_PROTEIN\_FOLDING |  | 4 | 0.44 | 0.88 | 0.587 | 0.898 | 1.000 | 1531 | tags=50%, list=13%, signal=57% |
| 1286 | GOBP\_WHITE\_FAT\_CELL\_DIFFERENTIATION |  | 11 | 0.30 | 0.88 | 0.606 | 0.899 | 1.000 | 297 | tags=18%, list=2%, signal=19% |
| 1287 | GOBP\_PARTURITION |  | 8 | 0.34 | 0.88 | 0.615 | 0.899 | 1.000 | 7943 | tags=100%, list=66%, signal=295% |
| 1288 | GOBP\_FEMALE\_MEIOSIS\_I |  | 4 | 0.44 | 0.88 | 0.584 | 0.899 | 1.000 | 6762 | tags=100%, list=56%, signal=229% |
| 1289 | GOBP\_REGULATION\_OF\_CELL\_CHEMOTAXIS\_TO\_FIBROBLAST\_GROWTH\_FACTOR |  | 3 | 0.49 | 0.88 | 0.586 | 0.898 | 1.000 | 126 | tags=33%, list=1%, signal=34% |
| 1290 | GOBP\_REGULATION\_OF\_ESTABLISHMENT\_OF\_PROTEIN\_LOCALIZATION\_TO\_CHROMOSOME |  | 10 | 0.31 | 0.88 | 0.614 | 0.898 | 1.000 | 8290 | tags=100%, list=69%, signal=323% |
| 1291 | GOBP\_ANDROGEN\_METABOLIC\_PROCESS |  | 20 | 0.25 | 0.88 | 0.651 | 0.900 | 1.000 | 2339 | tags=45%, list=19%, signal=56% |
| 1292 | GOCC\_SNO\_S\_RNA\_CONTAINING\_RIBONUCLEOPROTEIN\_COMPLEX |  | 16 | 0.27 | 0.88 | 0.629 | 0.900 | 1.000 | 2952 | tags=50%, list=25%, signal=66% |
| 1293 | GOCC\_POLE\_PLASM |  | 10 | 0.31 | 0.88 | 0.603 | 0.899 | 1.000 | 562 | tags=20%, list=5%, signal=21% |
| 1294 | GOBP\_REGULATION\_OF\_T\_HELPER\_1\_CELL\_DIFFERENTIATION |  | 8 | 0.34 | 0.88 | 0.591 | 0.899 | 1.000 | 267 | tags=13%, list=2%, signal=13% |
| 1295 | GOBP\_PEROXISOME\_FISSION |  | 7 | 0.35 | 0.88 | 0.602 | 0.899 | 1.000 | 2118 | tags=43%, list=18%, signal=52% |
| 1296 | GOBP\_COMMON\_PARTNER\_SMAD\_PROTEIN\_PHOSPHORYLATION |  | 3 | 0.49 | 0.88 | 0.595 | 0.899 | 1.000 | 6133 | tags=100%, list=51%, signal=204% |
| 1297 | GOBP\_GUANOSINE\_CONTAINING\_COMPOUND\_METABOLIC\_PROCESS |  | 31 | 0.22 | 0.88 | 0.672 | 0.898 | 1.000 | 463 | tags=13%, list=4%, signal=13% |
| 1298 | GOBP\_PEPTIDE\_BIOSYNTHETIC\_PROCESS |  | 500 | 0.14 | 0.88 | 1.000 | 0.898 | 1.000 | 2783 | tags=33%, list=23%, signal=41% |
| 1299 | GOBP\_EPITHELIAL\_FLUID\_TRANSPORT |  | 9 | 0.32 | 0.88 | 0.599 | 0.898 | 1.000 | 7 | tags=11%, list=0%, signal=11% |
| 1300 | GOBP\_PROTON\_TRANSPORTING\_TWO\_SECTOR\_ATPASE\_COMPLEX\_ASSEMBLY |  | 10 | 0.31 | 0.88 | 0.597 | 0.898 | 1.000 | 857 | tags=20%, list=7%, signal=22% |
| 1301 | GOBP\_REGULATION\_OF\_SOMITOGENESIS |  | 6 | 0.37 | 0.88 | 0.594 | 0.898 | 1.000 | 207 | tags=17%, list=2%, signal=17% |
| 1302 | GOBP\_PARENTAL\_BEHAVIOR |  | 6 | 0.37 | 0.88 | 0.594 | 0.898 | 1.000 | 1090 | tags=33%, list=9%, signal=37% |
| 1303 | GOBP\_STEROID\_HORMONE\_BIOSYNTHETIC\_PROCESS |  | 29 | 0.23 | 0.88 | 0.686 | 0.898 | 1.000 | 1641 | tags=28%, list=14%, signal=32% |
| 1304 | GOBP\_NEGATIVE\_REGULATION\_OF\_MEIOTIC\_NUCLEAR\_DIVISION |  | 4 | 0.44 | 0.88 | 0.599 | 0.898 | 1.000 | 6762 | tags=100%, list=56%, signal=229% |
| 1305 | GOCC\_SECONDARY\_LYSOSOME |  | 13 | 0.29 | 0.88 | 0.625 | 0.897 | 1.000 | 594 | tags=23%, list=5%, signal=24% |
| 1306 | GOBP\_HYPEROSMOTIC\_RESPONSE |  | 18 | 0.26 | 0.88 | 0.667 | 0.898 | 1.000 | 1548 | tags=33%, list=13%, signal=38% |
| 1307 | GOMF\_FUCOSYLTRANSFERASE\_ACTIVITY |  | 8 | 0.34 | 0.88 | 0.596 | 0.899 | 1.000 | 49 | tags=13%, list=0%, signal=13% |
| 1308 | GOCC\_ELONGATOR\_HOLOENZYME\_COMPLEX |  | 4 | 0.43 | 0.88 | 0.594 | 0.900 | 1.000 | 6801 | tags=100%, list=57%, signal=230% |
| 1309 | GOBP\_AGGRESSIVE\_BEHAVIOR |  | 4 | 0.43 | 0.88 | 0.589 | 0.899 | 1.000 | 179 | tags=25%, list=1%, signal=25% |
| 1310 | GOMF\_BILE\_ACID\_BINDING |  | 6 | 0.37 | 0.88 | 0.582 | 0.899 | 1.000 | 1755 | tags=50%, list=15%, signal=59% |
| 1311 | GOBP\_IMMUNE\_COMPLEX\_CLEARANCE |  | 3 | 0.49 | 0.88 | 0.598 | 0.900 | 1.000 | 299 | tags=33%, list=2%, signal=34% |
| 1312 | GOBP\_NEPHRON\_TUBULE\_EPITHELIAL\_CELL\_DIFFERENTIATION |  | 11 | 0.30 | 0.88 | 0.621 | 0.900 | 1.000 | 569 | tags=27%, list=5%, signal=29% |
| 1313 | GOBP\_MITOCHONDRIAL\_TRANSCRIPTION |  | 11 | 0.30 | 0.88 | 0.608 | 0.901 | 1.000 | 579 | tags=18%, list=5%, signal=19% |
| 1314 | GOBP\_CELLULAR\_RESPONSE\_TO\_FLUID\_SHEAR\_STRESS |  | 15 | 0.27 | 0.87 | 0.650 | 0.902 | 1.000 | 1192 | tags=20%, list=10%, signal=22% |
| 1315 | GOBP\_AMINE\_BIOSYNTHETIC\_PROCESS |  | 26 | 0.23 | 0.87 | 0.679 | 0.902 | 1.000 | 1035 | tags=19%, list=9%, signal=21% |
| 1316 | GOBP\_NAD\_METABOLIC\_PROCESS |  | 38 | 0.21 | 0.87 | 0.707 | 0.902 | 1.000 | 577 | tags=11%, list=5%, signal=11% |
| 1317 | GOBP\_PROTEIN\_LOCALIZATION\_TO\_MICROTUBULE\_END |  | 4 | 0.44 | 0.87 | 0.588 | 0.903 | 1.000 | 6766 | tags=100%, list=56%, signal=229% |
| 1318 | GOBP\_REGULATION\_OF\_PHOSPHATIDYLCHOLINE\_METABOLIC\_PROCESS |  | 8 | 0.33 | 0.87 | 0.607 | 0.907 | 1.000 | 3045 | tags=63%, list=25%, signal=84% |
| 1319 | GOBP\_GLOMERULAR\_MESANGIAL\_CELL\_DIFFERENTIATION |  | 5 | 0.40 | 0.87 | 0.601 | 0.907 | 1.000 | 7263 | tags=100%, list=60%, signal=253% |
| 1320 | GOBP\_RESPONSE\_TO\_INTERLEUKIN\_12 |  | 37 | 0.21 | 0.87 | 0.701 | 0.907 | 1.000 | 839 | tags=14%, list=7%, signal=14% |
| 1321 | GOBP\_CHEMOSENSORY\_BEHAVIOR |  | 5 | 0.40 | 0.87 | 0.608 | 0.907 | 1.000 | 265 | tags=20%, list=2%, signal=20% |
| 1322 | GOBP\_RDNA\_HETEROCHROMATIN\_ASSEMBLY |  | 7 | 0.35 | 0.87 | 0.615 | 0.906 | 1.000 | 1173 | tags=29%, list=10%, signal=32% |
| 1323 | GOBP\_TRNA\_THIO\_MODIFICATION |  | 4 | 0.43 | 0.87 | 0.615 | 0.906 | 1.000 | 6846 | tags=100%, list=57%, signal=232% |
| 1324 | GOBP\_DIRECT\_OSSIFICATION |  | 5 | 0.40 | 0.87 | 0.604 | 0.907 | 1.000 | 7168 | tags=100%, list=60%, signal=248% |
| 1325 | GOCC\_CDC73\_PAF1\_COMPLEX |  | 8 | 0.33 | 0.87 | 0.616 | 0.907 | 1.000 | 8038 | tags=100%, list=67%, signal=302% |
| 1326 | GOBP\_POSITIVE\_REGULATION\_OF\_SKELETAL\_MUSCLE\_TISSUE\_REGENERATION |  | 3 | 0.48 | 0.87 | 0.595 | 0.908 | 1.000 | 309 | tags=33%, list=3%, signal=34% |
| 1327 | GOCC\_PRE\_SNORNP\_COMPLEX |  | 6 | 0.37 | 0.87 | 0.607 | 0.911 | 1.000 | 7609 | tags=100%, list=63%, signal=273% |
| 1328 | GOBP\_REGULATION\_OF\_SKELETAL\_MUSCLE\_TISSUE\_REGENERATION |  | 3 | 0.48 | 0.87 | 0.612 | 0.911 | 1.000 | 309 | tags=33%, list=3%, signal=34% |
| 1329 | GOMF\_SNORNA\_BINDING |  | 26 | 0.23 | 0.87 | 0.680 | 0.911 | 1.000 | 1695 | tags=23%, list=14%, signal=27% |
| 1330 | GOBP\_RESPONSE\_TO\_ACID\_CHEMICAL |  | 84 | 0.18 | 0.87 | 0.840 | 0.911 | 1.000 | 1388 | tags=15%, list=12%, signal=17% |
| 1331 | GOBP\_REGULATION\_OF\_OSTEOCLAST\_DEVELOPMENT |  | 6 | 0.37 | 0.87 | 0.627 | 0.912 | 1.000 | 21 | tags=17%, list=0%, signal=17% |
| 1332 | GOBP\_NEGATIVE\_REGULATION\_OF\_MONOCYTE\_CHEMOTAXIS |  | 6 | 0.37 | 0.86 | 0.604 | 0.911 | 1.000 | 982 | tags=33%, list=8%, signal=36% |
| 1333 | GOBP\_POSITIVE\_REGULATION\_OF\_INTRACELLULAR\_ESTROGEN\_RECEPTOR\_SIGNALING\_PATHWAY |  | 8 | 0.33 | 0.86 | 0.623 | 0.912 | 1.000 | 766 | tags=25%, list=6%, signal=27% |
| 1334 | GOBP\_REGULATION\_OF\_ATP\_BIOSYNTHETIC\_PROCESS |  | 11 | 0.30 | 0.86 | 0.646 | 0.913 | 1.000 | 577 | tags=18%, list=5%, signal=19% |
| 1335 | GOBP\_NEGATIVE\_REGULATION\_OF\_EXTRACELLULAR\_MATRIX\_ORGANIZATION |  | 9 | 0.32 | 0.86 | 0.631 | 0.912 | 1.000 | 1113 | tags=22%, list=9%, signal=24% |
| 1336 | GOBP\_MESANGIAL\_CELL\_DEVELOPMENT |  | 5 | 0.40 | 0.86 | 0.618 | 0.912 | 1.000 | 7263 | tags=100%, list=60%, signal=253% |
| 1337 | GOCC\_EUKARYOTIC\_TRANSLATION\_INITIATION\_FACTOR\_2B\_COMPLEX |  | 6 | 0.37 | 0.86 | 0.606 | 0.912 | 1.000 | 7605 | tags=100%, list=63%, signal=272% |
| 1338 | GOBP\_SPERM\_EJACULATION |  | 4 | 0.42 | 0.86 | 0.612 | 0.914 | 1.000 | 1090 | tags=50%, list=9%, signal=55% |
| 1339 | GOBP\_POSITIVE\_REGULATION\_OF\_VIRAL\_RELEASE\_FROM\_HOST\_CELL |  | 13 | 0.28 | 0.86 | 0.650 | 0.915 | 1.000 | 2585 | tags=46%, list=22%, signal=59% |
| 1340 | GOBP\_NEGATIVE\_REGULATION\_OF\_PROTEIN\_IMPORT |  | 11 | 0.30 | 0.86 | 0.637 | 0.914 | 1.000 | 2426 | tags=45%, list=20%, signal=57% |
| 1341 | GOMF\_CARBONYL\_REDUCTASE\_NADPH\_ACTIVITY |  | 3 | 0.48 | 0.86 | 0.613 | 0.914 | 1.000 | 6288 | tags=100%, list=52%, signal=210% |
| 1342 | GOBP\_POSITIVE\_REGULATION\_OF\_MRNA\_SPLICING\_VIA\_SPLICEOSOME |  | 15 | 0.27 | 0.86 | 0.651 | 0.914 | 1.000 | 659 | tags=13%, list=5%, signal=14% |
| 1343 | GOBP\_BINDING\_OF\_SPERM\_TO\_ZONA\_PELLUCIDA |  | 18 | 0.26 | 0.86 | 0.654 | 0.913 | 1.000 | 1301 | tags=22%, list=11%, signal=25% |
| 1344 | GOBP\_REGULATION\_OF\_LIPOPROTEIN\_PARTICLE\_CLEARANCE |  | 9 | 0.32 | 0.86 | 0.643 | 0.914 | 1.000 | 158 | tags=11%, list=1%, signal=11% |
| 1345 | GOBP\_OSTEOCLAST\_DEVELOPMENT |  | 8 | 0.33 | 0.86 | 0.640 | 0.915 | 1.000 | 21 | tags=13%, list=0%, signal=13% |
| 1346 | GOBP\_SNRNA\_PROCESSING |  | 22 | 0.24 | 0.86 | 0.689 | 0.914 | 1.000 | 730 | tags=14%, list=6%, signal=14% |
| 1347 | GOCC\_ENDOPLASMIC\_RETICULUM\_GOLGI\_INTERMEDIATE\_COMPARTMENT\_MEMBRANE |  | 55 | 0.19 | 0.86 | 0.780 | 0.915 | 1.000 | 1132 | tags=13%, list=9%, signal=14% |
| 1348 | GOBP\_CEREBRAL\_CORTEX\_TANGENTIAL\_MIGRATION |  | 5 | 0.39 | 0.86 | 0.619 | 0.914 | 1.000 | 7316 | tags=100%, list=61%, signal=256% |
| 1349 | GOBP\_TETRAPYRROLE\_BIOSYNTHETIC\_PROCESS |  | 24 | 0.23 | 0.86 | 0.693 | 0.914 | 1.000 | 9209 | tags=100%, list=77%, signal=428% |
| 1350 | GOBP\_NUCLEAR\_NCRNA\_SURVEILLANCE |  | 6 | 0.36 | 0.86 | 0.623 | 0.914 | 1.000 | 2421 | tags=50%, list=20%, signal=63% |
| 1351 | GOBP\_NUCLEOBASE\_CONTAINING\_SMALL\_MOLECULE\_CATABOLIC\_PROCESS |  | 35 | 0.21 | 0.86 | 0.753 | 0.916 | 1.000 | 346 | tags=9%, list=3%, signal=9% |
| 1352 | GOBP\_DRUG\_TRANSMEMBRANE\_TRANSPORT |  | 8 | 0.33 | 0.86 | 0.648 | 0.917 | 1.000 | 2156 | tags=50%, list=18%, signal=61% |
| 1353 | GOBP\_POSITIVE\_REGULATION\_OF\_EPITHELIAL\_CELL\_APOPTOTIC\_PROCESS |  | 21 | 0.24 | 0.86 | 0.717 | 0.917 | 1.000 | 1613 | tags=29%, list=13%, signal=33% |
| 1354 | GOBP\_POSITIVE\_REGULATION\_OF\_AMINO\_ACID\_TRANSPORT |  | 13 | 0.27 | 0.86 | 0.667 | 0.918 | 1.000 | 682 | tags=23%, list=6%, signal=24% |
| 1355 | GOCC\_JUNCTIONAL\_MEMBRANE\_COMPLEX |  | 3 | 0.47 | 0.85 | 0.620 | 0.918 | 1.000 | 6359 | tags=100%, list=53%, signal=212% |
| 1356 | GOBP\_ISOLEUCINE\_METABOLIC\_PROCESS |  | 5 | 0.39 | 0.85 | 0.608 | 0.919 | 1.000 | 2339 | tags=60%, list=19%, signal=74% |
| 1357 | GOMF\_MAP\_KINASE\_PHOSPHATASE\_ACTIVITY |  | 10 | 0.30 | 0.85 | 0.651 | 0.919 | 1.000 | 2345 | tags=50%, list=20%, signal=62% |
| 1358 | GOBP\_POSITIVE\_REGULATION\_OF\_CELLULAR\_RESPONSE\_TO\_TRANSFORMING\_GROWTH\_FACTOR\_BETA\_STIMULUS |  | 23 | 0.24 | 0.85 | 0.722 | 0.918 | 1.000 | 9192 | tags=100%, list=77%, signal=425% |
| 1359 | GOBP\_SEQUESTERING\_OF\_IRON\_ION |  | 3 | 0.47 | 0.85 | 0.638 | 0.918 | 1.000 | 222 | tags=33%, list=2%, signal=34% |
| 1360 | GOBP\_RESPONSE\_TO\_IRON\_II\_ION |  | 4 | 0.43 | 0.85 | 0.642 | 0.918 | 1.000 | 6904 | tags=100%, list=57%, signal=235% |
| 1361 | GOBP\_LOCOMOTION\_INVOLVED\_IN\_LOCOMOTORY\_BEHAVIOR |  | 4 | 0.43 | 0.85 | 0.632 | 0.917 | 1.000 | 664 | tags=25%, list=6%, signal=26% |
| 1362 | GOMF\_ANION\_CATION\_SYMPORTER\_ACTIVITY |  | 15 | 0.26 | 0.85 | 0.672 | 0.918 | 1.000 | 1482 | tags=33%, list=12%, signal=38% |
| 1363 | GOBP\_MAINTENANCE\_OF\_PROTEIN\_LOCATION\_IN\_MITOCHONDRION |  | 4 | 0.42 | 0.85 | 0.631 | 0.919 | 1.000 | 361 | tags=25%, list=3%, signal=26% |
| 1364 | GOMF\_CYTIDINE\_DEAMINASE\_ACTIVITY |  | 4 | 0.42 | 0.85 | 0.629 | 0.920 | 1.000 | 6983 | tags=100%, list=58%, signal=239% |
| 1365 | GOBP\_APICAL\_PROTEIN\_LOCALIZATION |  | 13 | 0.28 | 0.85 | 0.688 | 0.919 | 1.000 | 1900 | tags=31%, list=16%, signal=37% |
| 1366 | GOBP\_FEMALE\_MEIOTIC\_NUCLEAR\_DIVISION |  | 16 | 0.26 | 0.85 | 0.686 | 0.919 | 1.000 | 33 | tags=6%, list=0%, signal=6% |
| 1367 | GOBP\_STEROL\_IMPORT |  | 8 | 0.33 | 0.85 | 0.643 | 0.918 | 1.000 | 1702 | tags=38%, list=14%, signal=44% |
| 1368 | GOBP\_NEGATIVE\_REGULATION\_OF\_CELLULAR\_PROTEIN\_CATABOLIC\_PROCESS |  | 56 | 0.19 | 0.85 | 0.780 | 0.918 | 1.000 | 1531 | tags=18%, list=13%, signal=20% |
| 1369 | GOBP\_ACROSOMAL\_VESICLE\_EXOCYTOSIS |  | 7 | 0.34 | 0.85 | 0.629 | 0.918 | 1.000 | 580 | tags=29%, list=5%, signal=30% |
| 1370 | GOBP\_HISTONE\_H3\_K9\_DIMETHYLATION |  | 4 | 0.43 | 0.85 | 0.629 | 0.918 | 1.000 | 366 | tags=25%, list=3%, signal=26% |
| 1371 | GOCC\_SUMO\_LIGASE\_COMPLEX |  | 6 | 0.36 | 0.85 | 0.646 | 0.923 | 1.000 | 2767 | tags=67%, list=23%, signal=87% |
| 1372 | GOBP\_REGULATION\_OF\_BLOOD\_BRAIN\_BARRIER\_PERMEABILITY |  | 6 | 0.36 | 0.85 | 0.642 | 0.922 | 1.000 | 954 | tags=33%, list=8%, signal=36% |
| 1373 | GOBP\_POSITIVE\_REGULATION\_OF\_RESPONSE\_TO\_OXIDATIVE\_STRESS |  | 4 | 0.42 | 0.85 | 0.626 | 0.923 | 1.000 | 7005 | tags=100%, list=58%, signal=240% |
| 1374 | GOBP\_CELLULAR\_RESPONSE\_TO\_CAFFEINE |  | 7 | 0.34 | 0.85 | 0.657 | 0.923 | 1.000 | 339 | tags=14%, list=3%, signal=15% |
| 1375 | GOBP\_FUCOSE\_CATABOLIC\_PROCESS |  | 6 | 0.36 | 0.85 | 0.640 | 0.923 | 1.000 | 49 | tags=17%, list=0%, signal=17% |
| 1376 | GOBP\_GLYCOSIDE\_TRANSPORT |  | 3 | 0.47 | 0.85 | 0.630 | 0.923 | 1.000 | 147 | tags=33%, list=1%, signal=34% |
| 1377 | GOMF\_LIPID\_PHOSPHATASE\_ACTIVITY |  | 10 | 0.30 | 0.85 | 0.656 | 0.923 | 1.000 | 133 | tags=10%, list=1%, signal=10% |
| 1378 | GOBP\_NEGATIVE\_REGULATION\_OF\_FIBRINOLYSIS |  | 5 | 0.38 | 0.85 | 0.642 | 0.923 | 1.000 | 216 | tags=20%, list=2%, signal=20% |
| 1379 | GOBP\_REGULATION\_OF\_ORGANIC\_ACID\_TRANSPORT |  | 37 | 0.20 | 0.84 | 0.783 | 0.923 | 1.000 | 1237 | tags=22%, list=10%, signal=24% |
| 1380 | GOBP\_NCRNA\_PROCESSING |  | 297 | 0.15 | 0.84 | 1.000 | 0.923 | 1.000 | 2425 | tags=26%, list=20%, signal=32% |
| 1381 | GOCC\_CHYLOMICRON |  | 5 | 0.38 | 0.84 | 0.648 | 0.924 | 1.000 | 216 | tags=20%, list=2%, signal=20% |
| 1382 | GOBP\_CARDIAC\_CHAMBER\_FORMATION |  | 8 | 0.32 | 0.84 | 0.650 | 0.923 | 1.000 | 134 | tags=13%, list=1%, signal=13% |
| 1383 | GOBP\_PLASMA\_MEMBRANE\_RAFT\_ASSEMBLY |  | 4 | 0.42 | 0.84 | 0.650 | 0.923 | 1.000 | 2702 | tags=75%, list=22%, signal=97% |
| 1384 | GOBP\_ANDROGEN\_BIOSYNTHETIC\_PROCESS |  | 6 | 0.36 | 0.84 | 0.648 | 0.923 | 1.000 | 2190 | tags=50%, list=18%, signal=61% |
| 1385 | GOBP\_LOW\_DENSITY\_LIPOPROTEIN\_RECEPTOR\_PARTICLE\_METABOLIC\_PROCESS |  | 10 | 0.30 | 0.84 | 0.647 | 0.922 | 1.000 | 158 | tags=10%, list=1%, signal=10% |
| 1386 | GOBP\_REGULATION\_OF\_HISTONE\_H3\_K14\_ACETYLATION |  | 4 | 0.41 | 0.84 | 0.647 | 0.922 | 1.000 | 766 | tags=50%, list=6%, signal=53% |
| 1387 | GOBP\_FATTY\_ACID\_DERIVATIVE\_CATABOLIC\_PROCESS |  | 7 | 0.34 | 0.84 | 0.654 | 0.923 | 1.000 | 7943 | tags=100%, list=66%, signal=295% |
| 1388 | GOBP\_REVERSIBLE\_DIFFERENTIATION |  | 5 | 0.38 | 0.84 | 0.644 | 0.923 | 1.000 | 7401 | tags=100%, list=62%, signal=260% |
| 1389 | GOBP\_RESPONSE\_TO\_IMMOBILIZATION\_STRESS |  | 17 | 0.25 | 0.84 | 0.706 | 0.922 | 1.000 | 750 | tags=18%, list=6%, signal=19% |
| 1390 | GOBP\_COMMA\_SHAPED\_BODY\_MORPHOGENESIS |  | 3 | 0.47 | 0.84 | 0.652 | 0.921 | 1.000 | 1723 | tags=67%, list=14%, signal=78% |
| 1391 | GOCC\_PRERIBOSOME |  | 64 | 0.18 | 0.84 | 0.826 | 0.922 | 1.000 | 2970 | tags=42%, list=25%, signal=56% |
| 1392 | GOMF\_METALLOCHAPERONE\_ACTIVITY |  | 7 | 0.34 | 0.84 | 0.656 | 0.923 | 1.000 | 194 | tags=14%, list=2%, signal=15% |
| 1393 | GOBP\_REGULATION\_OF\_POSTSYNAPTIC\_NEUROTRANSMITTER\_RECEPTOR\_INTERNALIZATION |  | 6 | 0.36 | 0.84 | 0.651 | 0.924 | 1.000 | 7746 | tags=100%, list=64%, signal=281% |
| 1394 | GOCC\_INTRACILIARY\_TRANSPORT\_PARTICLE |  | 23 | 0.23 | 0.84 | 0.705 | 0.923 | 1.000 | 3017 | tags=35%, list=25%, signal=46% |
| 1395 | GOBP\_SUCCINYL\_COA\_METABOLIC\_PROCESS |  | 5 | 0.38 | 0.84 | 0.652 | 0.923 | 1.000 | 2550 | tags=60%, list=21%, signal=76% |
| 1396 | GOBP\_THIOESTER\_METABOLIC\_PROCESS |  | 79 | 0.18 | 0.84 | 0.822 | 0.924 | 1.000 | 1578 | tags=23%, list=13%, signal=26% |
| 1397 | GOBP\_NEGATIVE\_REGULATION\_OF\_ERAD\_PATHWAY |  | 5 | 0.38 | 0.84 | 0.648 | 0.923 | 1.000 | 7455 | tags=100%, list=62%, signal=264% |
| 1398 | GOBP\_REGULATION\_OF\_NEUROTRANSMITTER\_RECEPTOR\_LOCALIZATION\_TO\_POSTSYNAPTIC\_SPECIALIZATION\_MEMBRANE |  | 6 | 0.36 | 0.84 | 0.668 | 0.923 | 1.000 | 7657 | tags=100%, list=64%, signal=276% |
| 1399 | GOBP\_POSITIVE\_REGULATION\_OF\_TYPE\_I\_INTERFERON\_MEDIATED\_SIGNALING\_PATHWAY |  | 9 | 0.31 | 0.84 | 0.666 | 0.923 | 1.000 | 672 | tags=22%, list=6%, signal=24% |
| 1400 | GOBP\_THIAMINE\_CONTAINING\_COMPOUND\_METABOLIC\_PROCESS |  | 4 | 0.41 | 0.84 | 0.646 | 0.923 | 1.000 | 2473 | tags=75%, list=21%, signal=94% |
| 1401 | GOMF\_OXIDOREDUCTASE\_ACTIVITY\_ACTING\_ON\_THE\_CH\_CH\_GROUP\_OF\_DONORS\_OXYGEN\_AS\_ACCEPTOR |  | 8 | 0.32 | 0.84 | 0.666 | 0.924 | 1.000 | 8154 | tags=100%, list=68%, signal=311% |
| 1402 | GOBP\_AXIAL\_MESODERM\_DEVELOPMENT |  | 4 | 0.42 | 0.84 | 0.643 | 0.926 | 1.000 | 1576 | tags=50%, list=13%, signal=58% |
| 1403 | GOBP\_CELLULAR\_RESPONSE\_TO\_WATER\_STIMULUS |  | 3 | 0.46 | 0.84 | 0.652 | 0.926 | 1.000 | 179 | tags=33%, list=1%, signal=34% |
| 1404 | GOBP\_POSITIVE\_REGULATION\_OF\_SYSTEMIC\_ARTERIAL\_BLOOD\_PRESSURE |  | 5 | 0.38 | 0.83 | 0.656 | 0.930 | 1.000 | 817 | tags=40%, list=7%, signal=43% |
| 1405 | GOCC\_OLIGOSACCHARYLTRANSFERASE\_COMPLEX |  | 11 | 0.28 | 0.83 | 0.686 | 0.931 | 1.000 | 610 | tags=18%, list=5%, signal=19% |
| 1406 | GOMF\_SEQUENCE\_SPECIFIC\_MRNA\_BINDING |  | 9 | 0.30 | 0.83 | 0.665 | 0.930 | 1.000 | 8362 | tags=100%, list=70%, signal=329% |
| 1407 | GOBP\_EXOCYST\_LOCALIZATION |  | 5 | 0.37 | 0.83 | 0.663 | 0.930 | 1.000 | 1291 | tags=20%, list=11%, signal=22% |
| 1408 | GOBP\_INNER\_DYNEIN\_ARM\_ASSEMBLY |  | 4 | 0.42 | 0.83 | 0.661 | 0.930 | 1.000 | 7011 | tags=100%, list=58%, signal=240% |
| 1409 | GOBP\_METANEPHRIC\_NEPHRON\_MORPHOGENESIS |  | 15 | 0.25 | 0.83 | 0.711 | 0.929 | 1.000 | 1723 | tags=20%, list=14%, signal=23% |
| 1410 | GOCC\_MICROTUBULE\_ORGANIZING\_CENTER\_ATTACHMENT\_SITE |  | 6 | 0.36 | 0.83 | 0.654 | 0.930 | 1.000 | 98 | tags=17%, list=1%, signal=17% |
| 1411 | GOBP\_GLUTAMINE\_METABOLIC\_PROCESS |  | 15 | 0.25 | 0.83 | 0.706 | 0.931 | 1.000 | 687 | tags=13%, list=6%, signal=14% |
| 1412 | GOCC\_CORE\_MEDIATOR\_COMPLEX |  | 7 | 0.33 | 0.83 | 0.678 | 0.931 | 1.000 | 8013 | tags=100%, list=67%, signal=300% |
| 1413 | GOBP\_POSITIVE\_REGULATION\_OF\_SODIUM\_ION\_TRANSMEMBRANE\_TRANSPORTER\_ACTIVITY |  | 12 | 0.27 | 0.83 | 0.689 | 0.931 | 1.000 | 79 | tags=8%, list=1%, signal=8% |
| 1414 | GOBP\_PLASMA\_CELL\_DIFFERENTIATION |  | 4 | 0.41 | 0.83 | 0.665 | 0.932 | 1.000 | 818 | tags=25%, list=7%, signal=27% |
| 1415 | GOMF\_OXIDOREDUCTASE\_ACTIVITY\_ACTING\_ON\_PAIRED\_DONORS\_WITH\_INCORPORATION\_OR\_REDUCTION\_OF\_MOLECULAR\_OXYGEN\_REDUCED\_IRON\_SULFUR\_PROTEIN\_AS\_ONE\_DONOR\_AND\_INCORPORATION\_OF\_ONE\_ATOM\_OF\_OXYGEN |  | 3 | 0.46 | 0.83 | 0.666 | 0.932 | 1.000 | 1404 | tags=67%, list=12%, signal=75% |
| 1416 | GOBP\_CYTOPLASMIC\_TRANSLATIONAL\_ELONGATION |  | 10 | 0.29 | 0.83 | 0.683 | 0.932 | 1.000 | 8485 | tags=100%, list=71%, signal=340% |
| 1417 | GOBP\_REGULATION\_OF\_TOLL\_LIKE\_RECEPTOR\_7\_SIGNALING\_PATHWAY |  | 4 | 0.41 | 0.83 | 0.656 | 0.933 | 1.000 | 71 | tags=25%, list=1%, signal=25% |
| 1418 | GOBP\_POSITIVE\_REGULATION\_OF\_MEIOTIC\_NUCLEAR\_DIVISION |  | 6 | 0.36 | 0.83 | 0.665 | 0.934 | 1.000 | 7741 | tags=100%, list=64%, signal=281% |
| 1419 | GOBP\_REGULATION\_OF\_PROTEIN\_FOLDING |  | 9 | 0.30 | 0.83 | 0.675 | 0.934 | 1.000 | 2087 | tags=44%, list=17%, signal=54% |
| 1420 | GOBP\_PYRIMIDINE\_NUCLEOTIDE\_METABOLIC\_PROCESS |  | 38 | 0.20 | 0.83 | 0.792 | 0.934 | 1.000 | 463 | tags=8%, list=4%, signal=8% |
| 1421 | GOBP\_POSITIVE\_REGULATION\_OF\_EPITHELIAL\_TO\_MESENCHYMAL\_TRANSITION\_INVOLVED\_IN\_ENDOCARDIAL\_CUSHION\_FORMATION |  | 5 | 0.38 | 0.83 | 0.680 | 0.934 | 1.000 | 7506 | tags=100%, list=62%, signal=267% |
| 1422 | GOBP\_RRNA\_PSEUDOURIDINE\_SYNTHESIS |  | 7 | 0.33 | 0.83 | 0.668 | 0.934 | 1.000 | 8014 | tags=100%, list=67%, signal=300% |
| 1423 | GOBP\_MEMBRANE\_RAFT\_ORGANIZATION |  | 17 | 0.25 | 0.83 | 0.719 | 0.935 | 1.000 | 142 | tags=12%, list=1%, signal=12% |
| 1424 | GOMF\_TRANSLATION\_TERMINATION\_FACTOR\_ACTIVITY |  | 5 | 0.38 | 0.82 | 0.671 | 0.938 | 1.000 | 7465 | tags=100%, list=62%, signal=264% |
| 1425 | GOBP\_SHORT\_CHAIN\_FATTY\_ACID\_CATABOLIC\_PROCESS |  | 8 | 0.31 | 0.82 | 0.690 | 0.938 | 1.000 | 1216 | tags=38%, list=10%, signal=42% |
| 1426 | GOBP\_PIGMENT\_BIOSYNTHETIC\_PROCESS |  | 40 | 0.20 | 0.82 | 0.812 | 0.938 | 1.000 | 319 | tags=8%, list=3%, signal=8% |
| 1427 | GOMF\_MHC\_CLASS\_I\_PROTEIN\_BINDING |  | 10 | 0.29 | 0.82 | 0.690 | 0.937 | 1.000 | 1053 | tags=30%, list=9%, signal=33% |
| 1428 | GOMF\_PRENYLTRANSFERASE\_ACTIVITY |  | 16 | 0.25 | 0.82 | 0.731 | 0.938 | 1.000 | 1795 | tags=31%, list=15%, signal=37% |
| 1429 | GOBP\_RENAL\_SODIUM\_ION\_TRANSPORT |  | 3 | 0.45 | 0.82 | 0.669 | 0.939 | 1.000 | 2665 | tags=67%, list=22%, signal=86% |
| 1430 | GOBP\_NEGATIVE\_REGULATION\_OF\_HYPOXIA\_INDUCED\_INTRINSIC\_APOPTOTIC\_SIGNALING\_PATHWAY |  | 3 | 0.46 | 0.82 | 0.669 | 0.938 | 1.000 | 2221 | tags=67%, list=18%, signal=82% |
| 1431 | GOBP\_BASE\_EXCISION\_REPAIR\_AP\_SITE\_FORMATION |  | 8 | 0.31 | 0.82 | 0.697 | 0.938 | 1.000 | 3059 | tags=63%, list=25%, signal=84% |
| 1432 | GOBP\_RRNA\_3\_END\_PROCESSING |  | 10 | 0.29 | 0.82 | 0.705 | 0.939 | 1.000 | 8526 | tags=100%, list=71%, signal=344% |
| 1433 | GOBP\_SYNAPTIC\_VESICLE\_BUDDING\_FROM\_PRESYNAPTIC\_ENDOCYTIC\_ZONE\_MEMBRANE |  | 4 | 0.41 | 0.82 | 0.686 | 0.939 | 1.000 | 16 | tags=25%, list=0%, signal=25% |
| 1434 | GOBP\_RESPONSE\_TO\_ANGIOTENSIN |  | 17 | 0.25 | 0.82 | 0.741 | 0.938 | 1.000 | 14 | tags=6%, list=0%, signal=6% |
| 1435 | GOMF\_VOLTAGE\_GATED\_SODIUM\_CHANNEL\_ACTIVITY\_INVOLVED\_IN\_CARDIAC\_MUSCLE\_CELL\_ACTION\_POTENTIAL |  | 3 | 0.45 | 0.82 | 0.685 | 0.938 | 1.000 | 472 | tags=33%, list=4%, signal=35% |
| 1436 | GOBP\_MESENCHYMAL\_TO\_EPITHELIAL\_TRANSITION |  | 11 | 0.28 | 0.82 | 0.695 | 0.938 | 1.000 | 525 | tags=18%, list=4%, signal=19% |
| 1437 | GOBP\_NEGATIVE\_REGULATION\_OF\_MYELINATION |  | 7 | 0.33 | 0.82 | 0.685 | 0.939 | 1.000 | 993 | tags=29%, list=8%, signal=31% |
| 1438 | GOBP\_PURINE\_CONTAINING\_COMPOUND\_TRANSMEMBRANE\_TRANSPORT |  | 12 | 0.27 | 0.82 | 0.722 | 0.939 | 1.000 | 2023 | tags=33%, list=17%, signal=40% |
| 1439 | GOBP\_CYTOPLASMIC\_SEQUESTERING\_OF\_NF\_KAPPAB |  | 6 | 0.35 | 0.82 | 0.682 | 0.939 | 1.000 | 369 | tags=17%, list=3%, signal=17% |
| 1440 | GOBP\_POSITIVE\_REGULATION\_OF\_INTERLEUKIN\_5\_PRODUCTION |  | 7 | 0.33 | 0.82 | 0.702 | 0.938 | 1.000 | 749 | tags=29%, list=6%, signal=30% |
| 1441 | GOBP\_NEGATIVE\_REGULATION\_OF\_ESTABLISHMENT\_OF\_PROTEIN\_LOCALIZATION |  | 85 | 0.17 | 0.82 | 0.898 | 0.938 | 1.000 | 2200 | tags=25%, list=18%, signal=30% |
| 1442 | GOMF\_TRANSLATION\_INITIATION\_FACTOR\_ACTIVITY |  | 42 | 0.19 | 0.82 | 0.821 | 0.938 | 1.000 | 3089 | tags=40%, list=26%, signal=54% |
| 1443 | GOBP\_PEROXISOME\_PROLIFERATOR\_ACTIVATED\_RECEPTOR\_SIGNALING\_PATHWAY |  | 14 | 0.26 | 0.82 | 0.705 | 0.938 | 1.000 | 412 | tags=14%, list=3%, signal=15% |
| 1444 | GOBP\_NEPHRIC\_DUCT\_DEVELOPMENT |  | 9 | 0.30 | 0.82 | 0.706 | 0.937 | 1.000 | 1723 | tags=33%, list=14%, signal=39% |
| 1445 | GOBP\_POSITIVE\_REGULATION\_OF\_CALCINEURIN\_MEDIATED\_SIGNALING |  | 9 | 0.30 | 0.82 | 0.703 | 0.938 | 1.000 | 493 | tags=22%, list=4%, signal=23% |
| 1446 | GOBP\_REGULATION\_OF\_HYDROGEN\_PEROXIDE\_METABOLIC\_PROCESS |  | 10 | 0.29 | 0.82 | 0.710 | 0.938 | 1.000 | 2067 | tags=40%, list=17%, signal=48% |
| 1447 | GOCC\_EARP\_COMPLEX |  | 3 | 0.44 | 0.82 | 0.681 | 0.940 | 1.000 | 6673 | tags=100%, list=56%, signal=225% |
| 1448 | GOBP\_NEGATIVE\_REGULATION\_OF\_INTRINSIC\_APOPTOTIC\_SIGNALING\_PATHWAY\_IN\_RESPONSE\_TO\_DNA\_DAMAGE |  | 19 | 0.24 | 0.81 | 0.755 | 0.939 | 1.000 | 2059 | tags=32%, list=17%, signal=38% |
| 1449 | GOMF\_PALMITOYL\_COA\_HYDROLASE\_ACTIVITY |  | 10 | 0.29 | 0.81 | 0.712 | 0.939 | 1.000 | 2118 | tags=40%, list=18%, signal=49% |
| 1450 | GOBP\_SCF\_COMPLEX\_ASSEMBLY |  | 6 | 0.34 | 0.81 | 0.688 | 0.939 | 1.000 | 7875 | tags=100%, list=66%, signal=290% |
| 1451 | GOBP\_URETER\_DEVELOPMENT |  | 12 | 0.27 | 0.81 | 0.713 | 0.939 | 1.000 | 1723 | tags=33%, list=14%, signal=39% |
| 1452 | GOBP\_PROTEIN\_LOCALIZATION\_TO\_ENDOPLASMIC\_RETICULUM |  | 118 | 0.16 | 0.81 | 0.957 | 0.939 | 1.000 | 3085 | tags=41%, list=26%, signal=54% |
| 1453 | GOBP\_PANCREAS\_DEVELOPMENT |  | 51 | 0.19 | 0.81 | 0.852 | 0.939 | 1.000 | 1084 | tags=16%, list=9%, signal=17% |
| 1454 | GOBP\_CELLULAR\_RESPONSE\_TO\_COLD |  | 7 | 0.33 | 0.81 | 0.712 | 0.938 | 1.000 | 79 | tags=14%, list=1%, signal=14% |
| 1455 | GOBP\_PROTEIN\_CONTAINING\_COMPLEX\_REMODELING |  | 19 | 0.23 | 0.81 | 0.750 | 0.939 | 1.000 | 299 | tags=11%, list=2%, signal=11% |
| 1456 | GOBP\_PROTEIN\_INSERTION\_INTO\_MEMBRANE |  | 49 | 0.18 | 0.81 | 0.847 | 0.939 | 1.000 | 2373 | tags=31%, list=20%, signal=38% |
| 1457 | GOBP\_AMIDE\_BIOSYNTHETIC\_PROCESS |  | 603 | 0.13 | 0.81 | 1.000 | 0.939 | 1.000 | 2369 | tags=26%, list=20%, signal=30% |
| 1458 | GOBP\_REGULATION\_OF\_EPITHELIAL\_TO\_MESENCHYMAL\_TRANSITION\_INVOLVED\_IN\_ENDOCARDIAL\_CUSHION\_FORMATION |  | 5 | 0.38 | 0.81 | 0.683 | 0.938 | 1.000 | 7506 | tags=100%, list=62%, signal=267% |
| 1459 | GOBP\_GALACTOLIPID\_METABOLIC\_PROCESS |  | 5 | 0.37 | 0.81 | 0.702 | 0.938 | 1.000 | 827 | tags=40%, list=7%, signal=43% |
| 1460 | GOBP\_GLUCOSAMINE\_CONTAINING\_COMPOUND\_CATABOLIC\_PROCESS |  | 5 | 0.37 | 0.81 | 0.696 | 0.938 | 1.000 | 130 | tags=20%, list=1%, signal=20% |
| 1461 | GOCC\_AMPA\_GLUTAMATE\_RECEPTOR\_COMPLEX |  | 5 | 0.37 | 0.81 | 0.691 | 0.937 | 1.000 | 2759 | tags=60%, list=23%, signal=78% |
| 1462 | GOCC\_KINOCILIUM |  | 5 | 0.37 | 0.81 | 0.690 | 0.937 | 1.000 | 611 | tags=20%, list=5%, signal=21% |
| 1463 | GOBP\_UMP\_BIOSYNTHETIC\_PROCESS |  | 10 | 0.29 | 0.81 | 0.716 | 0.937 | 1.000 | 8584 | tags=100%, list=71%, signal=350% |
| 1464 | GOBP\_REGULATION\_OF\_NEUROTRANSMITTER\_UPTAKE |  | 11 | 0.28 | 0.81 | 0.717 | 0.937 | 1.000 | 969 | tags=18%, list=8%, signal=20% |
| 1465 | GOBP\_NEGATIVE\_REGULATION\_OF\_RYANODINE\_SENSITIVE\_CALCIUM\_RELEASE\_CHANNEL\_ACTIVITY |  | 7 | 0.33 | 0.81 | 0.700 | 0.937 | 1.000 | 8103 | tags=100%, list=67%, signal=307% |
| 1466 | GOBP\_POSTTRANSLATIONAL\_PROTEIN\_TARGETING\_TO\_MEMBRANE\_TRANSLOCATION |  | 7 | 0.32 | 0.81 | 0.711 | 0.937 | 1.000 | 960 | tags=29%, list=8%, signal=31% |
| 1467 | GOBP\_POSITIVE\_REGULATION\_OF\_HISTONE\_H3\_K9\_ACETYLATION |  | 3 | 0.45 | 0.81 | 0.689 | 0.937 | 1.000 | 525 | tags=33%, list=4%, signal=35% |
| 1468 | GOBP\_REGULATION\_BY\_VIRUS\_OF\_VIRAL\_PROTEIN\_LEVELS\_IN\_HOST\_CELL |  | 6 | 0.35 | 0.81 | 0.694 | 0.939 | 1.000 | 103 | tags=17%, list=1%, signal=17% |
| 1469 | GOCC\_PROTEIN\_PHOSPHATASE\_TYPE\_2A\_COMPLEX |  | 16 | 0.25 | 0.81 | 0.739 | 0.939 | 1.000 | 475 | tags=13%, list=4%, signal=13% |
| 1470 | GOBP\_POSITIVE\_REGULATION\_OF\_INTEGRIN\_MEDIATED\_SIGNALING\_PATHWAY |  | 7 | 0.33 | 0.81 | 0.696 | 0.938 | 1.000 | 8075 | tags=100%, list=67%, signal=305% |
| 1471 | GOBP\_MODULATION\_OF\_AGE\_RELATED\_BEHAVIORAL\_DECLINE |  | 5 | 0.36 | 0.81 | 0.692 | 0.938 | 1.000 | 7643 | tags=100%, list=64%, signal=275% |
| 1472 | GOBP\_MULTI\_ORGANISM\_MEMBRANE\_FUSION |  | 6 | 0.34 | 0.81 | 0.705 | 0.938 | 1.000 | 7900 | tags=100%, list=66%, signal=292% |
| 1473 | GOMF\_TRANSLATION\_REPRESSOR\_ACTIVITY\_MRNA\_REGULATORY\_ELEMENT\_BINDING |  | 10 | 0.29 | 0.81 | 0.732 | 0.940 | 1.000 | 184 | tags=10%, list=2%, signal=10% |
| 1474 | GOBP\_ARGININE\_CATABOLIC\_PROCESS |  | 7 | 0.32 | 0.81 | 0.707 | 0.941 | 1.000 | 1515 | tags=43%, list=13%, signal=49% |
| 1475 | GOBP\_PYRIMIDINE\_RIBONUCLEOTIDE\_METABOLIC\_PROCESS |  | 23 | 0.22 | 0.80 | 0.783 | 0.942 | 1.000 | 1965 | tags=22%, list=16%, signal=26% |
| 1476 | GOBP\_STRIATED\_MUSCLE\_ATROPHY |  | 6 | 0.34 | 0.80 | 0.703 | 0.942 | 1.000 | 1463 | tags=33%, list=12%, signal=38% |
| 1477 | GOBP\_REGULATION\_OF\_NEURONAL\_ACTION\_POTENTIAL |  | 3 | 0.44 | 0.80 | 0.699 | 0.941 | 1.000 | 6705 | tags=100%, list=56%, signal=226% |
| 1478 | GOBP\_SNRNA\_MODIFICATION |  | 6 | 0.34 | 0.80 | 0.694 | 0.941 | 1.000 | 7882 | tags=100%, list=66%, signal=291% |
| 1479 | GOBP\_POINTED\_END\_ACTIN\_FILAMENT\_CAPPING |  | 5 | 0.37 | 0.80 | 0.696 | 0.941 | 1.000 | 7565 | tags=100%, list=63%, signal=270% |
| 1480 | GOBP\_TRNA\_METABOLIC\_PROCESS |  | 125 | 0.16 | 0.80 | 0.951 | 0.941 | 1.000 | 2633 | tags=29%, list=22%, signal=37% |
| 1481 | GOCC\_GARP\_COMPLEX |  | 3 | 0.44 | 0.80 | 0.704 | 0.942 | 1.000 | 6673 | tags=100%, list=56%, signal=225% |
| 1482 | GOBP\_NUCLEAR\_ENVELOPE\_REASSEMBLY |  | 16 | 0.25 | 0.80 | 0.745 | 0.942 | 1.000 | 2811 | tags=50%, list=23%, signal=65% |
| 1483 | GOBP\_T\_CELL\_ACTIVATION\_VIA\_T\_CELL\_RECEPTOR\_CONTACT\_WITH\_ANTIGEN\_BOUND\_TO\_MHC\_MOLECULE\_ON\_ANTIGEN\_PRESENTING\_CELL |  | 4 | 0.40 | 0.80 | 0.695 | 0.942 | 1.000 | 7236 | tags=100%, list=60%, signal=251% |
| 1484 | GOBP\_GLUCOSE\_CATABOLIC\_PROCESS |  | 25 | 0.22 | 0.80 | 0.800 | 0.941 | 1.000 | 1465 | tags=24%, list=12%, signal=27% |
| 1485 | GOBP\_ARGININE\_METABOLIC\_PROCESS |  | 11 | 0.28 | 0.80 | 0.739 | 0.941 | 1.000 | 1515 | tags=36%, list=13%, signal=42% |
| 1486 | GOBP\_NUCLEOBASE\_CONTAINING\_SMALL\_MOLECULE\_INTERCONVERSION |  | 21 | 0.22 | 0.80 | 0.784 | 0.941 | 1.000 | 1965 | tags=29%, list=16%, signal=34% |
| 1487 | GOMF\_UBIQUITIN\_PROTEIN\_TRANSFERASE\_ACTIVATOR\_ACTIVITY |  | 7 | 0.32 | 0.80 | 0.697 | 0.941 | 1.000 | 2187 | tags=43%, list=18%, signal=52% |
| 1488 | GOBP\_NEGATIVE\_REGULATION\_OF\_TRANSCRIPTION\_FROM\_RNA\_POLYMERASE\_II\_PROMOTER\_IN\_RESPONSE\_TO\_STRESS |  | 9 | 0.30 | 0.80 | 0.722 | 0.941 | 1.000 | 8438 | tags=100%, list=70%, signal=336% |
| 1489 | GOBP\_VENTRICULAR\_CARDIAC\_MUSCLE\_CELL\_MEMBRANE\_REPOLARIZATION |  | 16 | 0.24 | 0.80 | 0.763 | 0.942 | 1.000 | 9104 | tags=100%, list=76%, signal=413% |
| 1490 | GOMF\_RNA\_CAP\_BINDING |  | 15 | 0.25 | 0.80 | 0.774 | 0.942 | 1.000 | 113 | tags=7%, list=1%, signal=7% |
| 1491 | GOBP\_REGULATION\_OF\_TOLL\_LIKE\_RECEPTOR\_9\_SIGNALING\_PATHWAY |  | 8 | 0.30 | 0.80 | 0.728 | 0.944 | 1.000 | 1070 | tags=25%, list=9%, signal=27% |
| 1492 | GOBP\_NEGATIVE\_REGULATION\_OF\_MACROPHAGE\_DERIVED\_FOAM\_CELL\_DIFFERENTIATION |  | 11 | 0.27 | 0.80 | 0.730 | 0.943 | 1.000 | 83 | tags=9%, list=1%, signal=9% |
| 1493 | GOBP\_REGULATION\_OF\_HEART\_MORPHOGENESIS |  | 7 | 0.32 | 0.80 | 0.710 | 0.943 | 1.000 | 134 | tags=14%, list=1%, signal=14% |
| 1494 | GOBP\_ESTABLISHMENT\_OF\_PIGMENT\_GRANULE\_LOCALIZATION |  | 19 | 0.23 | 0.80 | 0.787 | 0.945 | 1.000 | 505 | tags=11%, list=4%, signal=11% |
| 1495 | GOCC\_CONTRACTILE\_FIBER |  | 147 | 0.14 | 0.80 | 0.977 | 0.944 | 1.000 | 626 | tags=7%, list=5%, signal=8% |
| 1496 | GOBP\_REGULATION\_OF\_METALLOPEPTIDASE\_ACTIVITY |  | 12 | 0.26 | 0.80 | 0.745 | 0.944 | 1.000 | 1073 | tags=25%, list=9%, signal=27% |
| 1497 | GOCC\_EKC\_KEOPS\_COMPLEX |  | 3 | 0.44 | 0.80 | 0.706 | 0.945 | 1.000 | 1171 | tags=33%, list=10%, signal=37% |
| 1498 | GOBP\_PROTEIN\_LOCALIZATION\_TO\_BASOLATERAL\_PLASMA\_MEMBRANE |  | 5 | 0.36 | 0.80 | 0.720 | 0.944 | 1.000 | 7727 | tags=100%, list=64%, signal=280% |
| 1499 | GOCC\_AUTOLYSOSOME |  | 9 | 0.29 | 0.79 | 0.734 | 0.945 | 1.000 | 594 | tags=22%, list=5%, signal=23% |
| 1500 | GOMF\_OXIDOREDUCTASE\_ACTIVITY\_ACTING\_ON\_PAIRED\_DONORS\_WITH\_INCORPORATION\_OR\_REDUCTION\_OF\_MOLECULAR\_OXYGEN\_REDUCED\_PTERIDINE\_AS\_ONE\_DONOR\_AND\_INCORPORATION\_OF\_ONE\_ATOM\_OF\_OXYGEN |  | 4 | 0.40 | 0.79 | 0.700 | 0.945 | 1.000 | 586 | tags=25%, list=5%, signal=26% |
| 1501 | GOMF\_NUCLEOSIDE\_DIPHOSPHATASE\_ACTIVITY |  | 12 | 0.26 | 0.79 | 0.755 | 0.944 | 1.000 | 2527 | tags=33%, list=21%, signal=42% |
| 1502 | GOBP\_NEGATIVE\_REGULATION\_OF\_TELOMERASE\_ACTIVITY |  | 9 | 0.29 | 0.79 | 0.733 | 0.943 | 1.000 | 997 | tags=22%, list=8%, signal=24% |
| 1503 | GOBP\_GLOMERULAR\_MESANGIAL\_CELL\_DEVELOPMENT |  | 4 | 0.40 | 0.79 | 0.703 | 0.943 | 1.000 | 7263 | tags=100%, list=60%, signal=253% |
| 1504 | GOBP\_REGULATION\_OF\_SODIUM\_DEPENDENT\_PHOSPHATE\_TRANSPORT |  | 3 | 0.45 | 0.79 | 0.721 | 0.943 | 1.000 | 989 | tags=33%, list=8%, signal=36% |
| 1505 | GOCC\_LATE\_ENDOSOME\_MEMBRANE |  | 104 | 0.16 | 0.79 | 0.946 | 0.944 | 1.000 | 1762 | tags=21%, list=15%, signal=25% |
| 1506 | GOCC\_COPI\_COATED\_VESICLE |  | 23 | 0.22 | 0.79 | 0.802 | 0.943 | 1.000 | 1783 | tags=22%, list=15%, signal=25% |
| 1507 | GOCC\_BRCA1\_A\_COMPLEX |  | 6 | 0.34 | 0.79 | 0.718 | 0.943 | 1.000 | 425 | tags=17%, list=4%, signal=17% |
| 1508 | GOBP\_ACTIN\_FILAMENT\_REORGANIZATION |  | 7 | 0.32 | 0.79 | 0.746 | 0.943 | 1.000 | 1481 | tags=43%, list=12%, signal=49% |
| 1509 | GOBP\_POSITIVE\_REGULATION\_OF\_LIGASE\_ACTIVITY |  | 7 | 0.32 | 0.79 | 0.730 | 0.942 | 1.000 | 1388 | tags=43%, list=12%, signal=48% |
| 1510 | GOBP\_PURINE\_DEOXYRIBONUCLEOTIDE\_METABOLIC\_PROCESS |  | 9 | 0.29 | 0.79 | 0.725 | 0.942 | 1.000 | 383 | tags=22%, list=3%, signal=23% |
| 1511 | GOBP\_HEMOGLOBIN\_BIOSYNTHETIC\_PROCESS |  | 8 | 0.30 | 0.79 | 0.725 | 0.942 | 1.000 | 8365 | tags=100%, list=70%, signal=329% |
| 1512 | GOBP\_TRIPEPTIDE\_TRANSMEMBRANE\_TRANSPORT |  | 4 | 0.39 | 0.79 | 0.708 | 0.941 | 1.000 | 1442 | tags=50%, list=12%, signal=57% |
| 1513 | GOBP\_REGULATION\_OF\_RECEPTOR\_RECYCLING |  | 19 | 0.23 | 0.79 | 0.776 | 0.942 | 1.000 | 915 | tags=16%, list=8%, signal=17% |
| 1514 | GOBP\_LIPID\_IMPORT\_INTO\_CELL |  | 24 | 0.21 | 0.79 | 0.803 | 0.941 | 1.000 | 1278 | tags=21%, list=11%, signal=23% |
| 1515 | GOBP\_POLYADENYLATION\_DEPENDENT\_RNA\_CATABOLIC\_PROCESS |  | 7 | 0.32 | 0.79 | 0.727 | 0.941 | 1.000 | 2421 | tags=43%, list=20%, signal=54% |
| 1516 | GOBP\_PEPTIDYL\_LYSINE\_MONOMETHYLATION |  | 8 | 0.30 | 0.79 | 0.736 | 0.941 | 1.000 | 836 | tags=25%, list=7%, signal=27% |
| 1517 | GOBP\_REGULATION\_OF\_MITOCHONDRIAL\_MEMBRANE\_POTENTIAL |  | 51 | 0.18 | 0.79 | 0.880 | 0.941 | 1.000 | 1694 | tags=18%, list=14%, signal=20% |
| 1518 | GOBP\_REGULATION\_OF\_SODIUM\_PROTON\_ANTIPORTER\_ACTIVITY |  | 5 | 0.36 | 0.79 | 0.717 | 0.940 | 1.000 | 7726 | tags=100%, list=64%, signal=280% |
| 1519 | GOMF\_4\_IRON\_4\_SULFUR\_CLUSTER\_BINDING |  | 36 | 0.20 | 0.79 | 0.835 | 0.941 | 1.000 | 1371 | tags=22%, list=11%, signal=25% |
| 1520 | GOBP\_NEGATIVE\_REGULATION\_OF\_VASCULAR\_ENDOTHELIAL\_CELL\_PROLIFERATION |  | 3 | 0.44 | 0.79 | 0.707 | 0.942 | 1.000 | 83 | tags=33%, list=1%, signal=34% |
| 1521 | GOBP\_NON\_CANONICAL\_WNT\_SIGNALING\_PATHWAY\_VIA\_MAPK\_CASCADE |  | 5 | 0.36 | 0.79 | 0.735 | 0.942 | 1.000 | 7741 | tags=100%, list=64%, signal=281% |
| 1522 | GOMF\_LYSOPHOSPHOLIPASE\_ACTIVITY |  | 19 | 0.23 | 0.79 | 0.791 | 0.942 | 1.000 | 703 | tags=16%, list=6%, signal=17% |
| 1523 | GOBP\_PHOSPHATIDYLSERINE\_ACYL\_CHAIN\_REMODELING |  | 12 | 0.26 | 0.79 | 0.758 | 0.941 | 1.000 | 299 | tags=17%, list=2%, signal=17% |
| 1524 | GOMF\_TRNA\_ADENINE\_METHYLTRANSFERASE\_ACTIVITY |  | 4 | 0.39 | 0.79 | 0.728 | 0.941 | 1.000 | 7304 | tags=100%, list=61%, signal=255% |
| 1525 | GOBP\_RRNA\_CATABOLIC\_PROCESS |  | 15 | 0.25 | 0.79 | 0.764 | 0.941 | 1.000 | 1031 | tags=20%, list=9%, signal=22% |
| 1526 | GOBP\_NEGATIVE\_REGULATION\_OF\_RNA\_POLYMERASE\_II\_REGULATORY\_REGION\_SEQUENCE\_SPECIFIC\_DNA\_BINDING |  | 6 | 0.34 | 0.79 | 0.737 | 0.940 | 1.000 | 1724 | tags=50%, list=14%, signal=58% |
| 1527 | GOMF\_ANION\_SODIUM\_SYMPORTER\_ACTIVITY |  | 8 | 0.30 | 0.79 | 0.729 | 0.942 | 1.000 | 1482 | tags=38%, list=12%, signal=43% |
| 1528 | GOBP\_COLLAGEN\_BIOSYNTHETIC\_PROCESS |  | 33 | 0.19 | 0.79 | 0.848 | 0.942 | 1.000 | 1159 | tags=18%, list=10%, signal=20% |
| 1529 | GOBP\_POSITIVE\_REGULATION\_OF\_MITOPHAGY\_IN\_RESPONSE\_TO\_MITOCHONDRIAL\_DEPOLARIZATION |  | 5 | 0.36 | 0.79 | 0.740 | 0.942 | 1.000 | 1534 | tags=40%, list=13%, signal=46% |
| 1530 | GOMF\_CELL\_CELL\_ADHESION\_MEDIATOR\_ACTIVITY |  | 36 | 0.19 | 0.79 | 0.860 | 0.943 | 1.000 | 1725 | tags=22%, list=14%, signal=26% |
| 1531 | GOCC\_TRNA\_METHYLTRANSFERASE\_COMPLEX |  | 4 | 0.39 | 0.78 | 0.723 | 0.948 | 1.000 | 7304 | tags=100%, list=61%, signal=255% |
| 1532 | GOBP\_RNA\_PHOSPHODIESTER\_BOND\_HYDROLYSIS |  | 106 | 0.15 | 0.78 | 0.968 | 0.949 | 1.000 | 1054 | tags=12%, list=9%, signal=13% |
| 1533 | GOMF\_TRIPEPTIDE\_TRANSMEMBRANE\_TRANSPORTER\_ACTIVITY |  | 4 | 0.39 | 0.78 | 0.713 | 0.950 | 1.000 | 1442 | tags=50%, list=12%, signal=57% |
| 1534 | GOBP\_MITOCHONDRIAL\_DEPOLARIZATION |  | 19 | 0.23 | 0.78 | 0.805 | 0.949 | 1.000 | 673 | tags=11%, list=6%, signal=11% |
| 1535 | GOBP\_PIGMENT\_GRANULE\_LOCALIZATION |  | 21 | 0.22 | 0.78 | 0.804 | 0.951 | 1.000 | 505 | tags=10%, list=4%, signal=10% |
| 1536 | GOMF\_PEPTIDASE\_ACTIVATOR\_ACTIVITY\_INVOLVED\_IN\_APOPTOTIC\_PROCESS |  | 13 | 0.25 | 0.78 | 0.781 | 0.952 | 1.000 | 833 | tags=15%, list=7%, signal=17% |
| 1537 | GOBP\_POSITIVE\_REGULATION\_OF\_EXECUTION\_PHASE\_OF\_APOPTOSIS |  | 6 | 0.33 | 0.78 | 0.756 | 0.951 | 1.000 | 8044 | tags=100%, list=67%, signal=303% |
| 1538 | GOMF\_RAGE\_RECEPTOR\_BINDING |  | 4 | 0.39 | 0.78 | 0.724 | 0.951 | 1.000 | 7363 | tags=100%, list=61%, signal=258% |
| 1539 | GOCC\_SAGA\_COMPLEX |  | 11 | 0.27 | 0.78 | 0.767 | 0.952 | 1.000 | 2722 | tags=55%, list=23%, signal=70% |
| 1540 | GOBP\_ESTABLISHMENT\_OF\_VIRAL\_LATENCY |  | 9 | 0.28 | 0.78 | 0.759 | 0.953 | 1.000 | 1942 | tags=44%, list=16%, signal=53% |
| 1541 | GOBP\_PROTEIN\_LOCALIZATION\_TO\_PHOTORECEPTOR\_OUTER\_SEGMENT |  | 3 | 0.43 | 0.78 | 0.750 | 0.953 | 1.000 | 6838 | tags=100%, list=57%, signal=232% |
| 1542 | GOMF\_TROPOMYOSIN\_BINDING |  | 10 | 0.27 | 0.78 | 0.760 | 0.953 | 1.000 | 69 | tags=10%, list=1%, signal=10% |
| 1543 | GOMF\_HEXOSAMINIDASE\_ACTIVITY |  | 9 | 0.29 | 0.78 | 0.763 | 0.952 | 1.000 | 1256 | tags=33%, list=10%, signal=37% |
| 1544 | GOCC\_LOW\_DENSITY\_LIPOPROTEIN\_PARTICLE |  | 6 | 0.33 | 0.78 | 0.741 | 0.952 | 1.000 | 3061 | tags=67%, list=25%, signal=89% |
| 1545 | GOBP\_POTASSIUM\_ION\_IMPORT\_ACROSS\_PLASMA\_MEMBRANE |  | 24 | 0.21 | 0.77 | 0.842 | 0.952 | 1.000 | 2312 | tags=33%, list=19%, signal=41% |
| 1546 | GOBP\_T\_TUBULE\_ORGANIZATION |  | 6 | 0.33 | 0.77 | 0.761 | 0.953 | 1.000 | 183 | tags=17%, list=2%, signal=17% |
| 1547 | GOBP\_PROTEIN\_INSERTION\_INTO\_ER\_MEMBRANE |  | 11 | 0.26 | 0.77 | 0.777 | 0.955 | 1.000 | 8831 | tags=100%, list=74%, signal=377% |
| 1548 | GOBP\_MRNA\_TRANSCRIPTION |  | 11 | 0.27 | 0.77 | 0.771 | 0.956 | 1.000 | 989 | tags=18%, list=8%, signal=20% |
| 1549 | GOBP\_PLASMA\_MEMBRANE\_RAFT\_ORGANIZATION |  | 5 | 0.35 | 0.77 | 0.756 | 0.956 | 1.000 | 2702 | tags=60%, list=22%, signal=77% |
| 1550 | GOBP\_NCRNA\_3\_END\_PROCESSING |  | 31 | 0.20 | 0.77 | 0.860 | 0.956 | 1.000 | 730 | tags=10%, list=6%, signal=10% |
| 1551 | GOBP\_NEGATIVE\_REGULATION\_OF\_HYDROGEN\_PEROXIDE\_MEDIATED\_PROGRAMMED\_CELL\_DEATH |  | 4 | 0.38 | 0.77 | 0.742 | 0.957 | 1.000 | 7414 | tags=100%, list=62%, signal=261% |
| 1552 | GOMF\_NUCLEOSIDE\_KINASE\_ACTIVITY |  | 9 | 0.29 | 0.77 | 0.781 | 0.957 | 1.000 | 8584 | tags=100%, list=71%, signal=350% |
| 1553 | GOMF\_PROTEIN\_TYROSINE\_KINASE\_INHIBITOR\_ACTIVITY |  | 3 | 0.42 | 0.77 | 0.748 | 0.959 | 1.000 | 6977 | tags=100%, list=58%, signal=239% |
| 1554 | GOBP\_HYALURONAN\_METABOLIC\_PROCESS |  | 28 | 0.20 | 0.77 | 0.867 | 0.959 | 1.000 | 1440 | tags=21%, list=12%, signal=24% |
| 1555 | GOBP\_MODULATION\_OF\_MOLECULAR\_FUNCTION\_IN\_OTHER\_ORGANISM |  | 5 | 0.35 | 0.77 | 0.763 | 0.959 | 1.000 | 514 | tags=20%, list=4%, signal=21% |
| 1556 | GOBP\_MAMMARY\_GLAND\_BRANCHING\_INVOLVED\_IN\_PREGNANCY |  | 5 | 0.35 | 0.77 | 0.775 | 0.958 | 1.000 | 449 | tags=20%, list=4%, signal=21% |
| 1557 | GOCC\_PROTEASOME\_REGULATORY\_PARTICLE\_BASE\_SUBCOMPLEX |  | 11 | 0.27 | 0.77 | 0.774 | 0.958 | 1.000 | 2619 | tags=36%, list=22%, signal=46% |
| 1558 | GOMF\_DOPAMINE\_BINDING |  | 3 | 0.42 | 0.77 | 0.742 | 0.958 | 1.000 | 670 | tags=33%, list=6%, signal=35% |
| 1559 | GOCC\_SEPTIN\_COMPLEX |  | 3 | 0.42 | 0.77 | 0.748 | 0.958 | 1.000 | 6917 | tags=100%, list=58%, signal=236% |
| 1560 | GOCC\_ANAPHASE\_PROMOTING\_COMPLEX |  | 18 | 0.22 | 0.77 | 0.815 | 0.958 | 1.000 | 1471 | tags=17%, list=12%, signal=19% |
| 1561 | GOBP\_PROTEIN\_N\_LINKED\_GLYCOSYLATION |  | 61 | 0.17 | 0.77 | 0.940 | 0.958 | 1.000 | 810 | tags=13%, list=7%, signal=14% |
| 1562 | GOBP\_MATURE\_RIBOSOME\_ASSEMBLY |  | 5 | 0.35 | 0.77 | 0.735 | 0.957 | 1.000 | 1306 | tags=40%, list=11%, signal=45% |
| 1563 | GOBP\_EYELID\_DEVELOPMENT\_IN\_CAMERA\_TYPE\_EYE |  | 8 | 0.30 | 0.77 | 0.773 | 0.957 | 1.000 | 908 | tags=25%, list=8%, signal=27% |
| 1564 | GOMF\_ENDORIBONUCLEASE\_ACTIVITY |  | 38 | 0.19 | 0.76 | 0.897 | 0.959 | 1.000 | 1054 | tags=18%, list=9%, signal=20% |
| 1565 | GOMF\_TBP\_CLASS\_PROTEIN\_BINDING |  | 21 | 0.22 | 0.76 | 0.828 | 0.959 | 1.000 | 2986 | tags=43%, list=25%, signal=57% |
| 1566 | GOBP\_GLUTATHIONE\_TRANSMEMBRANE\_TRANSPORT |  | 3 | 0.43 | 0.76 | 0.765 | 0.959 | 1.000 | 1442 | tags=67%, list=12%, signal=76% |
| 1567 | GOBP\_NEGATIVE\_REGULATION\_OF\_THYMOCYTE\_APOPTOTIC\_PROCESS |  | 5 | 0.35 | 0.76 | 0.766 | 0.959 | 1.000 | 246 | tags=20%, list=2%, signal=20% |
| 1568 | GOMF\_GLYCEROL\_TRANSMEMBRANE\_TRANSPORTER\_ACTIVITY |  | 4 | 0.38 | 0.76 | 0.768 | 0.960 | 1.000 | 7455 | tags=100%, list=62%, signal=264% |
| 1569 | GOMF\_DOPAMINE\_NEUROTRANSMITTER\_RECEPTOR\_ACTIVITY |  | 3 | 0.42 | 0.76 | 0.754 | 0.960 | 1.000 | 670 | tags=33%, list=6%, signal=35% |
| 1570 | GOBP\_POSITIVE\_REGULATION\_OF\_ISOTYPE\_SWITCHING |  | 17 | 0.23 | 0.76 | 0.820 | 0.960 | 1.000 | 799 | tags=18%, list=7%, signal=19% |
| 1571 | GOCC\_NURF\_COMPLEX |  | 3 | 0.42 | 0.76 | 0.758 | 0.959 | 1.000 | 6963 | tags=100%, list=58%, signal=238% |
| 1572 | GOBP\_NEUROTRANSMITTER\_LOADING\_INTO\_SYNAPTIC\_VESICLE |  | 3 | 0.42 | 0.76 | 0.766 | 0.959 | 1.000 | 715 | tags=33%, list=6%, signal=35% |
| 1573 | GOMF\_PROTEIN\_DISULFIDE\_ISOMERASE\_ACTIVITY |  | 17 | 0.22 | 0.76 | 0.827 | 0.958 | 1.000 | 576 | tags=12%, list=5%, signal=12% |
| 1574 | GOBP\_REGULATION\_OF\_PLASMA\_MEMBRANE\_ORGANIZATION |  | 12 | 0.25 | 0.76 | 0.804 | 0.959 | 1.000 | 1463 | tags=25%, list=12%, signal=28% |
| 1575 | GOBP\_GLYCEROL\_TRANSPORT |  | 4 | 0.38 | 0.76 | 0.759 | 0.958 | 1.000 | 7455 | tags=100%, list=62%, signal=264% |
| 1576 | GOBP\_PROTEIN\_LOCALIZATION\_TO\_NON\_MOTILE\_CILIUM |  | 6 | 0.32 | 0.76 | 0.776 | 0.958 | 1.000 | 8123 | tags=100%, list=68%, signal=309% |
| 1577 | GOBP\_NEGATIVE\_REGULATION\_OF\_HEART\_RATE |  | 5 | 0.35 | 0.76 | 0.764 | 0.958 | 1.000 | 7848 | tags=100%, list=65%, signal=288% |
| 1578 | GOBP\_MATERNAL\_PROCESS\_INVOLVED\_IN\_PARTURITION |  | 4 | 0.38 | 0.76 | 0.759 | 0.958 | 1.000 | 7480 | tags=100%, list=62%, signal=265% |
| 1579 | GOBP\_REGULATION\_OF\_OXIDATIVE\_STRESS\_INDUCED\_CELL\_DEATH |  | 43 | 0.18 | 0.76 | 0.914 | 0.958 | 1.000 | 2060 | tags=28%, list=17%, signal=34% |
| 1580 | GOBP\_MRNA\_CLEAVAGE |  | 15 | 0.24 | 0.76 | 0.819 | 0.958 | 1.000 | 110 | tags=7%, list=1%, signal=7% |
| 1581 | GOBP\_NEGATIVE\_REGULATION\_OF\_NOREPINEPHRINE\_SECRETION |  | 5 | 0.35 | 0.76 | 0.768 | 0.957 | 1.000 | 7864 | tags=100%, list=65%, signal=290% |
| 1582 | GOMF\_OPIOID\_RECEPTOR\_ACTIVITY |  | 4 | 0.37 | 0.76 | 0.765 | 0.958 | 1.000 | 7517 | tags=100%, list=63%, signal=267% |
| 1583 | GOCC\_ER\_MEMBRANE\_INSERTION\_COMPLEX |  | 3 | 0.42 | 0.76 | 0.768 | 0.958 | 1.000 | 6976 | tags=100%, list=58%, signal=238% |
| 1584 | GOBP\_SNRNA\_METABOLIC\_PROCESS |  | 31 | 0.19 | 0.76 | 0.888 | 0.957 | 1.000 | 9729 | tags=100%, list=81%, signal=525% |
| 1585 | GOBP\_POSITIVE\_REGULATION\_OF\_POTASSIUM\_ION\_TRANSMEMBRANE\_TRANSPORT |  | 21 | 0.21 | 0.76 | 0.852 | 0.957 | 1.000 | 2534 | tags=43%, list=21%, signal=54% |
| 1586 | GOBP\_PYRIMIDINE\_CONTAINING\_COMPOUND\_BIOSYNTHETIC\_PROCESS |  | 31 | 0.19 | 0.76 | 0.883 | 0.957 | 1.000 | 463 | tags=6%, list=4%, signal=7% |
| 1587 | GOBP\_RESPONSE\_TO\_STILBENOID |  | 4 | 0.38 | 0.75 | 0.768 | 0.960 | 1.000 | 339 | tags=25%, list=3%, signal=26% |
| 1588 | GOBP\_RECEPTOR\_MEDIATED\_ENDOCYTOSIS\_INVOLVED\_IN\_CHOLESTEROL\_TRANSPORT |  | 5 | 0.34 | 0.75 | 0.777 | 0.962 | 1.000 | 158 | tags=20%, list=1%, signal=20% |
| 1589 | GOBP\_ADENOHYPOPHYSIS\_DEVELOPMENT |  | 5 | 0.34 | 0.75 | 0.773 | 0.963 | 1.000 | 1084 | tags=40%, list=9%, signal=44% |
| 1590 | GOBP\_REGULATION\_OF\_PHOSPHOLIPID\_BIOSYNTHETIC\_PROCESS |  | 14 | 0.24 | 0.75 | 0.817 | 0.962 | 1.000 | 2169 | tags=29%, list=18%, signal=35% |
| 1591 | GOBP\_NCRNA\_METABOLIC\_PROCESS |  | 353 | 0.13 | 0.75 | 1.000 | 0.962 | 1.000 | 2425 | tags=26%, list=20%, signal=31% |
| 1592 | GOBP\_2\_OXOGLUTARATE\_METABOLIC\_PROCESS |  | 15 | 0.23 | 0.75 | 0.825 | 0.962 | 1.000 | 673 | tags=13%, list=6%, signal=14% |
| 1593 | GOBP\_VIRAL\_GENE\_EXPRESSION |  | 161 | 0.14 | 0.75 | 1.000 | 0.962 | 1.000 | 3085 | tags=39%, list=26%, signal=52% |
| 1594 | GOBP\_AXONEMAL\_DYNEIN\_COMPLEX\_ASSEMBLY |  | 12 | 0.25 | 0.75 | 0.800 | 0.962 | 1.000 | 1660 | tags=25%, list=14%, signal=29% |
| 1595 | GOMF\_CO\_RECEPTOR\_BINDING |  | 6 | 0.32 | 0.75 | 0.777 | 0.963 | 1.000 | 1723 | tags=50%, list=14%, signal=58% |
| 1596 | GOBP\_POSITIVE\_REGULATION\_OF\_PROTEIN\_KINASE\_A\_SIGNALING |  | 6 | 0.32 | 0.75 | 0.786 | 0.965 | 1.000 | 317 | tags=17%, list=3%, signal=17% |
| 1597 | GOBP\_MEMBRANE\_FISSION |  | 11 | 0.25 | 0.75 | 0.815 | 0.966 | 1.000 | 16 | tags=9%, list=0%, signal=9% |
| 1598 | GOBP\_EPITHELIAL\_CELL\_PROLIFERATION\_INVOLVED\_IN\_PROSTATE\_GLAND\_DEVELOPMENT |  | 7 | 0.30 | 0.75 | 0.805 | 0.967 | 1.000 | 8447 | tags=100%, list=70%, signal=337% |
| 1599 | GOBP\_PROGESTERONE\_RECEPTOR\_SIGNALING\_PATHWAY |  | 9 | 0.27 | 0.75 | 0.813 | 0.967 | 1.000 | 766 | tags=22%, list=6%, signal=24% |
| 1600 | GOBP\_POSTTRANSLATIONAL\_PROTEIN\_TARGETING\_TO\_ENDOPLASMIC\_RETICULUM\_MEMBRANE |  | 10 | 0.26 | 0.75 | 0.807 | 0.967 | 1.000 | 1638 | tags=30%, list=14%, signal=35% |
| 1601 | GOBP\_RETINAL\_PIGMENT\_EPITHELIUM\_DEVELOPMENT |  | 4 | 0.36 | 0.75 | 0.797 | 0.966 | 1.000 | 489 | tags=25%, list=4%, signal=26% |
| 1602 | GOCC\_CHROMATIN\_SILENCING\_COMPLEX |  | 7 | 0.30 | 0.74 | 0.788 | 0.967 | 1.000 | 1173 | tags=29%, list=10%, signal=32% |
| 1603 | GOMF\_K48\_LINKED\_POLYUBIQUITIN\_MODIFICATION\_DEPENDENT\_PROTEIN\_BINDING |  | 3 | 0.41 | 0.74 | 0.777 | 0.967 | 1.000 | 672 | tags=33%, list=6%, signal=35% |
| 1604 | GOMF\_CYSTEINE\_TYPE\_EXOPEPTIDASE\_ACTIVITY |  | 3 | 0.41 | 0.74 | 0.791 | 0.967 | 1.000 | 7088 | tags=100%, list=59%, signal=244% |
| 1605 | GOCC\_CUL5\_RING\_UBIQUITIN\_LIGASE\_COMPLEX |  | 4 | 0.37 | 0.74 | 0.788 | 0.967 | 1.000 | 729 | tags=25%, list=6%, signal=27% |
| 1606 | GOBP\_REGULATION\_OF\_CELLULAR\_RESPONSE\_TO\_HYPOXIA |  | 7 | 0.30 | 0.74 | 0.803 | 0.968 | 1.000 | 2221 | tags=43%, list=18%, signal=53% |
| 1607 | GOBP\_PROTEIN\_LIPOYLATION |  | 5 | 0.34 | 0.74 | 0.800 | 0.968 | 1.000 | 356 | tags=20%, list=3%, signal=21% |
| 1608 | GOBP\_RESPONSE\_TO\_SYMBIONT |  | 5 | 0.34 | 0.74 | 0.779 | 0.968 | 1.000 | 190 | tags=20%, list=2%, signal=20% |
| 1609 | GOBP\_PROTEIN\_AUTOPROCESSING |  | 14 | 0.23 | 0.74 | 0.845 | 0.969 | 1.000 | 489 | tags=14%, list=4%, signal=15% |
| 1610 | GOBP\_PROTEIN\_O\_LINKED\_FUCOSYLATION |  | 4 | 0.37 | 0.74 | 0.781 | 0.968 | 1.000 | 507 | tags=25%, list=4%, signal=26% |
| 1611 | GOBP\_NEGATIVE\_REGULATION\_OF\_RECEPTOR\_MEDIATED\_ENDOCYTOSIS |  | 21 | 0.21 | 0.74 | 0.873 | 0.969 | 1.000 | 1587 | tags=19%, list=13%, signal=22% |
| 1612 | GOCC\_SCHMIDT\_LANTERMAN\_INCISURE |  | 8 | 0.28 | 0.74 | 0.814 | 0.968 | 1.000 | 8620 | tags=100%, list=72%, signal=354% |
| 1613 | GOBP\_RESPONSE\_TO\_ACIDIC\_PH |  | 18 | 0.22 | 0.74 | 0.848 | 0.968 | 1.000 | 1292 | tags=22%, list=11%, signal=25% |
| 1614 | GOBP\_TRNA\_3\_END\_PROCESSING |  | 6 | 0.31 | 0.74 | 0.790 | 0.968 | 1.000 | 8271 | tags=100%, list=69%, signal=321% |
| 1615 | GOBP\_POST\_ANAL\_TAIL\_MORPHOGENESIS |  | 10 | 0.26 | 0.74 | 0.827 | 0.968 | 1.000 | 752 | tags=20%, list=6%, signal=21% |
| 1616 | GOBP\_CITRATE\_METABOLIC\_PROCESS |  | 5 | 0.34 | 0.74 | 0.789 | 0.968 | 1.000 | 7973 | tags=100%, list=66%, signal=297% |
| 1617 | GOMF\_AMMONIA\_LYASE\_ACTIVITY |  | 4 | 0.37 | 0.74 | 0.796 | 0.968 | 1.000 | 2320 | tags=75%, list=19%, signal=93% |
| 1618 | GOMF\_NUCLEOBASE\_BINDING |  | 4 | 0.37 | 0.74 | 0.802 | 0.969 | 1.000 | 1755 | tags=50%, list=15%, signal=59% |
| 1619 | GOBP\_REGULATION\_OF\_AUTOPHAGY\_OF\_MITOCHONDRION\_IN\_RESPONSE\_TO\_MITOCHONDRIAL\_DEPOLARIZATION |  | 8 | 0.28 | 0.74 | 0.821 | 0.969 | 1.000 | 2060 | tags=38%, list=17%, signal=45% |
| 1620 | GOBP\_S\_SHAPED\_BODY\_MORPHOGENESIS |  | 4 | 0.36 | 0.74 | 0.787 | 0.968 | 1.000 | 1723 | tags=50%, list=14%, signal=58% |
| 1621 | GOBP\_MAINTENANCE\_OF\_CELL\_POLARITY |  | 14 | 0.23 | 0.74 | 0.835 | 0.968 | 1.000 | 79 | tags=7%, list=1%, signal=7% |
| 1622 | GOBP\_UMP\_CATABOLIC\_PROCESS |  | 4 | 0.37 | 0.74 | 0.796 | 0.969 | 1.000 | 346 | tags=25%, list=3%, signal=26% |
| 1623 | GOBP\_NEGATIVE\_REGULATION\_OF\_BONE\_MINERALIZATION |  | 7 | 0.29 | 0.73 | 0.817 | 0.968 | 1.000 | 8524 | tags=100%, list=71%, signal=344% |
| 1624 | GOBP\_PYRIMIDINE\_NUCLEOSIDE\_MONOPHOSPHATE\_CATABOLIC\_PROCESS |  | 4 | 0.37 | 0.73 | 0.800 | 0.969 | 1.000 | 346 | tags=25%, list=3%, signal=26% |
| 1625 | GOBP\_INSEMINATION |  | 8 | 0.28 | 0.73 | 0.819 | 0.970 | 1.000 | 1090 | tags=25%, list=9%, signal=27% |
| 1626 | GOBP\_SIGNAL\_TRANSDUCTION\_INVOLVED\_IN\_REGULATION\_OF\_GENE\_EXPRESSION |  | 8 | 0.28 | 0.73 | 0.811 | 0.970 | 1.000 | 1104 | tags=25%, list=9%, signal=28% |
| 1627 | GOBP\_CDP\_CHOLINE\_PATHWAY |  | 5 | 0.33 | 0.73 | 0.813 | 0.969 | 1.000 | 7997 | tags=100%, list=67%, signal=299% |
| 1628 | GOCC\_HIGH\_DENSITY\_LIPOPROTEIN\_PARTICLE |  | 10 | 0.26 | 0.73 | 0.827 | 0.970 | 1.000 | 216 | tags=10%, list=2%, signal=10% |
| 1629 | GOMF\_LOW\_DENSITY\_LIPOPROTEIN\_PARTICLE\_BINDING |  | 14 | 0.23 | 0.73 | 0.859 | 0.971 | 1.000 | 1609 | tags=21%, list=13%, signal=25% |
| 1630 | GOMF\_FIBRONECTIN\_BINDING |  | 21 | 0.20 | 0.73 | 0.892 | 0.971 | 1.000 | 9564 | tags=100%, list=80%, signal=490% |
| 1631 | GOBP\_REGULATION\_OF\_INWARD\_RECTIFIER\_POTASSIUM\_CHANNEL\_ACTIVITY |  | 4 | 0.36 | 0.73 | 0.786 | 0.971 | 1.000 | 7695 | tags=100%, list=64%, signal=278% |
| 1632 | GOMF\_FILAMIN\_BINDING |  | 8 | 0.28 | 0.73 | 0.818 | 0.971 | 1.000 | 1156 | tags=25%, list=10%, signal=28% |
| 1633 | GOCC\_COPI\_VESICLE\_COAT |  | 10 | 0.26 | 0.73 | 0.820 | 0.971 | 1.000 | 8897 | tags=100%, list=74%, signal=385% |
| 1634 | GOBP\_REGULATION\_OF\_PROTEIN\_AUTOUBIQUITINATION |  | 4 | 0.36 | 0.73 | 0.796 | 0.973 | 1.000 | 729 | tags=25%, list=6%, signal=27% |
| 1635 | GOBP\_R\_LOOP\_DISASSEMBLY |  | 5 | 0.33 | 0.73 | 0.807 | 0.972 | 1.000 | 8049 | tags=100%, list=67%, signal=303% |
| 1636 | GOMF\_IONOTROPIC\_GLUTAMATE\_RECEPTOR\_BINDING |  | 10 | 0.26 | 0.73 | 0.838 | 0.972 | 1.000 | 969 | tags=20%, list=8%, signal=22% |
| 1637 | GOBP\_TRNA\_WOBBLE\_BASE\_MODIFICATION |  | 15 | 0.23 | 0.72 | 0.859 | 0.975 | 1.000 | 2975 | tags=47%, list=25%, signal=62% |
| 1638 | GOCC\_FEMALE\_PRONUCLEUS |  | 5 | 0.33 | 0.72 | 0.819 | 0.975 | 1.000 | 178 | tags=20%, list=1%, signal=20% |
| 1639 | GOBP\_NEGATIVE\_REGULATION\_OF\_MACROPHAGE\_DIFFERENTIATION |  | 6 | 0.31 | 0.72 | 0.825 | 0.976 | 1.000 | 8320 | tags=100%, list=69%, signal=325% |
| 1640 | GOBP\_TRANSLATIONAL\_INITIATION |  | 157 | 0.13 | 0.72 | 1.000 | 0.978 | 1.000 | 3089 | tags=40%, list=26%, signal=53% |
| 1641 | GOBP\_REGULATION\_OF\_CELL\_FATE\_COMMITMENT |  | 16 | 0.22 | 0.72 | 0.875 | 0.979 | 1.000 | 2145 | tags=38%, list=18%, signal=46% |
| 1642 | GOCC\_COPI\_COATED\_VESICLE\_MEMBRANE |  | 14 | 0.23 | 0.72 | 0.866 | 0.979 | 1.000 | 9274 | tags=100%, list=77%, signal=438% |
| 1643 | GOBP\_REGULATION\_OF\_TRANSMISSION\_OF\_NERVE\_IMPULSE |  | 6 | 0.31 | 0.72 | 0.817 | 0.978 | 1.000 | 179 | tags=17%, list=1%, signal=17% |
| 1644 | GOBP\_VASCULAR\_ASSOCIATED\_SMOOTH\_MUSCLE\_CELL\_DEVELOPMENT |  | 9 | 0.27 | 0.72 | 0.839 | 0.980 | 1.000 | 8816 | tags=100%, list=73%, signal=376% |
| 1645 | GOBP\_NUCLEOTIDE\_TRANSMEMBRANE\_TRANSPORT |  | 12 | 0.24 | 0.72 | 0.848 | 0.981 | 1.000 | 2023 | tags=25%, list=17%, signal=30% |
| 1646 | GOBP\_MINUS\_END\_DIRECTED\_ORGANELLE\_TRANSPORT\_ALONG\_MICROTUBULE |  | 5 | 0.32 | 0.72 | 0.826 | 0.981 | 1.000 | 384 | tags=20%, list=3%, signal=21% |
| 1647 | GOBP\_VITAMIN\_K\_METABOLIC\_PROCESS |  | 3 | 0.39 | 0.72 | 0.837 | 0.980 | 1.000 | 7299 | tags=100%, list=61%, signal=255% |
| 1648 | GOBP\_RESPONSE\_TO\_FOLLICLE\_STIMULATING\_HORMONE |  | 9 | 0.26 | 0.71 | 0.860 | 0.982 | 1.000 | 1388 | tags=22%, list=12%, signal=25% |
| 1649 | GOMF\_PYRUVATE\_DEHYDROGENASE\_ACTIVITY |  | 5 | 0.33 | 0.71 | 0.819 | 0.982 | 1.000 | 8090 | tags=100%, list=67%, signal=306% |
| 1650 | GOBP\_NEGATIVE\_REGULATION\_OF\_ENDOCYTOSIS |  | 33 | 0.18 | 0.71 | 0.908 | 0.982 | 1.000 | 539 | tags=9%, list=4%, signal=9% |
| 1651 | GOBP\_METANEPHRIC\_NEPHRON\_TUBULE\_EPITHELIAL\_CELL\_DIFFERENTIATION |  | 4 | 0.36 | 0.71 | 0.826 | 0.982 | 1.000 | 103 | tags=25%, list=1%, signal=25% |
| 1652 | GOCC\_IKAPPAB\_KINASE\_COMPLEX |  | 5 | 0.33 | 0.71 | 0.824 | 0.982 | 1.000 | 113 | tags=20%, list=1%, signal=20% |
| 1653 | GOBP\_EMBRYONIC\_SKELETAL\_JOINT\_MORPHOGENESIS |  | 8 | 0.27 | 0.71 | 0.847 | 0.982 | 1.000 | 2011 | tags=50%, list=17%, signal=60% |
| 1654 | GOBP\_MESENCHYMAL\_CELL\_APOPTOTIC\_PROCESS |  | 6 | 0.30 | 0.71 | 0.851 | 0.982 | 1.000 | 488 | tags=17%, list=4%, signal=17% |
| 1655 | GOBP\_RIBONUCLEOPROTEIN\_COMPLEX\_BIOGENESIS |  | 342 | 0.12 | 0.71 | 1.000 | 0.983 | 1.000 | 2799 | tags=30%, list=23%, signal=38% |
| 1656 | GOBP\_DENDRITE\_SELF\_AVOIDANCE |  | 12 | 0.24 | 0.71 | 0.868 | 0.983 | 1.000 | 1523 | tags=25%, list=13%, signal=29% |
| 1657 | GOBP\_REGULATION\_OF\_CELLULAR\_CARBOHYDRATE\_CATABOLIC\_PROCESS |  | 8 | 0.27 | 0.71 | 0.831 | 0.983 | 1.000 | 8729 | tags=100%, list=73%, signal=366% |
| 1658 | GOMF\_ICOSANOID\_RECEPTOR\_ACTIVITY |  | 9 | 0.26 | 0.71 | 0.855 | 0.983 | 1.000 | 83 | tags=11%, list=1%, signal=11% |
| 1659 | GOBP\_POSITIVE\_REGULATION\_OF\_CILIUM\_MOVEMENT |  | 4 | 0.35 | 0.71 | 0.818 | 0.982 | 1.000 | 265 | tags=25%, list=2%, signal=26% |
| 1660 | GOMF\_CYSTEINE\_TYPE\_ENDOPEPTIDASE\_ACTIVITY\_INVOLVED\_IN\_APOPTOTIC\_PROCESS |  | 9 | 0.26 | 0.71 | 0.861 | 0.982 | 1.000 | 778 | tags=22%, list=6%, signal=24% |
| 1661 | GOBP\_FIBROBLAST\_ACTIVATION |  | 8 | 0.27 | 0.71 | 0.848 | 0.983 | 1.000 | 1388 | tags=38%, list=12%, signal=42% |
| 1662 | GOBP\_PYRIMIDINE\_RIBONUCLEOTIDE\_CATABOLIC\_PROCESS |  | 6 | 0.30 | 0.71 | 0.856 | 0.985 | 1.000 | 346 | tags=17%, list=3%, signal=17% |
| 1663 | GOCC\_PARANODAL\_JUNCTION |  | 7 | 0.29 | 0.71 | 0.849 | 0.986 | 1.000 | 8580 | tags=100%, list=71%, signal=350% |
| 1664 | GOBP\_MATURATION\_OF\_5\_8S\_RRNA\_FROM\_TRICISTRONIC\_RRNA\_TRANSCRIPT\_SSU\_RRNA\_5\_8S\_RRNA\_LSU\_RRNA |  | 21 | 0.20 | 0.70 | 0.920 | 0.985 | 1.000 | 2421 | tags=33%, list=20%, signal=42% |
| 1665 | GOBP\_BIOTIN\_METABOLIC\_PROCESS |  | 9 | 0.25 | 0.70 | 0.863 | 0.985 | 1.000 | 172 | tags=11%, list=1%, signal=11% |
| 1666 | GOMF\_ATPASE\_REGULATOR\_ACTIVITY |  | 33 | 0.18 | 0.70 | 0.923 | 0.985 | 1.000 | 1712 | tags=21%, list=14%, signal=25% |
| 1667 | GOBP\_ANION\_HOMEOSTASIS |  | 17 | 0.21 | 0.70 | 0.893 | 0.985 | 1.000 | 1482 | tags=24%, list=12%, signal=27% |
| 1668 | GOBP\_CELL\_DIFFERENTIATION\_INVOLVED\_IN\_PHENOTYPIC\_SWITCHING |  | 3 | 0.38 | 0.70 | 0.835 | 0.987 | 1.000 | 7401 | tags=100%, list=62%, signal=260% |
| 1669 | GOBP\_PEPTIDYL\_GLUTAMIC\_ACID\_MODIFICATION |  | 17 | 0.21 | 0.70 | 0.901 | 0.987 | 1.000 | 1311 | tags=18%, list=11%, signal=20% |
| 1670 | GOMF\_DNA\_METHYLTRANSFERASE\_ACTIVITY |  | 6 | 0.30 | 0.70 | 0.844 | 0.987 | 1.000 | 8417 | tags=100%, list=70%, signal=334% |
| 1671 | GOBP\_KERATINOCYTE\_APOPTOTIC\_PROCESS |  | 3 | 0.38 | 0.70 | 0.840 | 0.986 | 1.000 | 7394 | tags=100%, list=62%, signal=260% |
| 1672 | GOBP\_POSITIVE\_REGULATION\_OF\_INOSITOL\_TRISPHOSPHATE\_BIOSYNTHETIC\_PROCESS |  | 3 | 0.39 | 0.70 | 0.842 | 0.986 | 1.000 | 2436 | tags=67%, list=20%, signal=84% |
| 1673 | GOBP\_POSITIVE\_REGULATION\_OF\_HORMONE\_BIOSYNTHETIC\_PROCESS |  | 7 | 0.28 | 0.70 | 0.857 | 0.986 | 1.000 | 1478 | tags=29%, list=12%, signal=33% |
| 1674 | GOMF\_METALLOENDOPEPTIDASE\_INHIBITOR\_ACTIVITY |  | 9 | 0.26 | 0.70 | 0.851 | 0.986 | 1.000 | 8844 | tags=100%, list=74%, signal=379% |
| 1675 | GOBP\_REGULATION\_OF\_DENDRITIC\_CELL\_DIFFERENTIATION |  | 3 | 0.39 | 0.70 | 0.842 | 0.987 | 1.000 | 7363 | tags=100%, list=61%, signal=258% |
| 1676 | GOBP\_EXTRACELLULAR\_REGULATION\_OF\_SIGNAL\_TRANSDUCTION |  | 9 | 0.26 | 0.70 | 0.869 | 0.989 | 1.000 | 8948 | tags=100%, list=74%, signal=392% |
| 1677 | GOMF\_CYSTEINE\_TYPE\_ENDOPEPTIDASE\_ACTIVITY\_INVOLVED\_IN\_EXECUTION\_PHASE\_OF\_APOPTOSIS |  | 8 | 0.27 | 0.69 | 0.864 | 0.991 | 1.000 | 778 | tags=25%, list=6%, signal=27% |
| 1678 | GOBP\_EMBRYONIC\_BRAIN\_DEVELOPMENT |  | 6 | 0.30 | 0.69 | 0.858 | 0.991 | 1.000 | 8452 | tags=100%, list=70%, signal=337% |
| 1679 | GOBP\_TRANSLESION\_SYNTHESIS |  | 32 | 0.18 | 0.69 | 0.934 | 0.990 | 1.000 | 141 | tags=6%, list=1%, signal=6% |
| 1680 | GOMF\_LIGASE\_ACTIVITY\_FORMING\_CARBON\_NITROGEN\_BONDS |  | 38 | 0.16 | 0.69 | 0.953 | 0.990 | 1.000 | 10042 | tags=100%, list=84%, signal=608% |
| 1681 | GOBP\_POSITIVE\_REGULATION\_OF\_CEREBELLAR\_GRANULE\_CELL\_PRECURSOR\_PROLIFERATION |  | 6 | 0.30 | 0.69 | 0.848 | 0.989 | 1.000 | 8447 | tags=100%, list=70%, signal=337% |
| 1682 | GOBP\_REGULATION\_OF\_PROTEIN\_COMPLEX\_STABILITY |  | 9 | 0.25 | 0.69 | 0.879 | 0.989 | 1.000 | 1497 | tags=33%, list=12%, signal=38% |
| 1683 | GOBP\_REGULATION\_OF\_INOSITOL\_TRISPHOSPHATE\_BIOSYNTHETIC\_PROCESS |  | 4 | 0.35 | 0.69 | 0.857 | 0.989 | 1.000 | 7864 | tags=100%, list=65%, signal=290% |
| 1684 | GOBP\_LIPID\_DROPLET\_FORMATION |  | 5 | 0.32 | 0.69 | 0.852 | 0.989 | 1.000 | 581 | tags=20%, list=5%, signal=21% |
| 1685 | GOBP\_PROLINE\_BIOSYNTHETIC\_PROCESS |  | 3 | 0.39 | 0.69 | 0.852 | 0.988 | 1.000 | 7380 | tags=100%, list=61%, signal=259% |
| 1686 | GOBP\_NEGATIVE\_REGULATION\_OF\_PROTEIN\_EXPORT\_FROM\_NUCLEUS |  | 5 | 0.31 | 0.69 | 0.860 | 0.988 | 1.000 | 8246 | tags=100%, list=69%, signal=319% |
| 1687 | GOBP\_REGULATION\_OF\_POTASSIUM\_ION\_EXPORT\_ACROSS\_PLASMA\_MEMBRANE |  | 5 | 0.32 | 0.69 | 0.842 | 0.988 | 1.000 | 2179 | tags=60%, list=18%, signal=73% |
| 1688 | GOCC\_STEREOCILIUM\_TIP |  | 7 | 0.28 | 0.69 | 0.850 | 0.988 | 1.000 | 1241 | tags=29%, list=10%, signal=32% |
| 1689 | GOBP\_REGULATION\_OF\_CHOLESTEROL\_ESTERIFICATION |  | 7 | 0.28 | 0.69 | 0.866 | 0.988 | 1.000 | 2794 | tags=57%, list=23%, signal=74% |
| 1690 | GOBP\_MINERALOCORTICOID\_METABOLIC\_PROCESS |  | 6 | 0.29 | 0.69 | 0.859 | 0.987 | 1.000 | 134 | tags=17%, list=1%, signal=17% |
| 1691 | GOBP\_NAD\_BIOSYNTHESIS\_VIA\_NICOTINAMIDE\_RIBOSIDE\_SALVAGE\_PATHWAY |  | 8 | 0.27 | 0.69 | 0.866 | 0.987 | 1.000 | 644 | tags=13%, list=5%, signal=13% |
| 1692 | GOBP\_UTERINE\_SMOOTH\_MUSCLE\_CONTRACTION |  | 6 | 0.30 | 0.69 | 0.838 | 0.987 | 1.000 | 8468 | tags=100%, list=71%, signal=339% |
| 1693 | GOMF\_TRANSLATION\_REGULATOR\_ACTIVITY |  | 103 | 0.14 | 0.69 | 1.000 | 0.987 | 1.000 | 2782 | tags=28%, list=23%, signal=36% |
| 1694 | GOMF\_LIPASE\_ACTIVATOR\_ACTIVITY |  | 8 | 0.26 | 0.69 | 0.888 | 0.987 | 1.000 | 216 | tags=13%, list=2%, signal=13% |
| 1695 | GOMF\_MOLYBDOPTERIN\_COFACTOR\_BINDING |  | 3 | 0.38 | 0.69 | 0.868 | 0.988 | 1.000 | 282 | tags=33%, list=2%, signal=34% |
| 1696 | GOBP\_MODULATION\_BY\_VIRUS\_OF\_HOST\_PROCESS |  | 24 | 0.19 | 0.69 | 0.931 | 0.987 | 1.000 | 568 | tags=13%, list=5%, signal=13% |
| 1697 | GOMF\_RNA\_METHYLTRANSFERASE\_ACTIVITY |  | 52 | 0.16 | 0.69 | 0.977 | 0.988 | 1.000 | 2371 | tags=33%, list=20%, signal=41% |
| 1698 | GOBP\_NEGATIVE\_REGULATION\_OF\_OXIDATIVE\_STRESS\_INDUCED\_NEURON\_DEATH |  | 9 | 0.25 | 0.69 | 0.873 | 0.988 | 1.000 | 1130 | tags=22%, list=9%, signal=25% |
| 1699 | GOBP\_REGULATION\_OF\_ALDOSTERONE\_METABOLIC\_PROCESS |  | 6 | 0.29 | 0.69 | 0.876 | 0.988 | 1.000 | 134 | tags=17%, list=1%, signal=17% |
| 1700 | GOMF\_CLATHRIN\_LIGHT\_CHAIN\_BINDING |  | 5 | 0.31 | 0.69 | 0.882 | 0.987 | 1.000 | 400 | tags=20%, list=3%, signal=21% |
| 1701 | GOMF\_GLYCEROL\_CHANNEL\_ACTIVITY |  | 3 | 0.38 | 0.69 | 0.852 | 0.987 | 1.000 | 7455 | tags=100%, list=62%, signal=264% |
| 1702 | GOMF\_GUANYLYLTRANSFERASE\_ACTIVITY |  | 4 | 0.34 | 0.68 | 0.861 | 0.988 | 1.000 | 7879 | tags=100%, list=66%, signal=291% |
| 1703 | GOBP\_CELLULAR\_RESPONSE\_TO\_FATTY\_ACID |  | 25 | 0.18 | 0.68 | 0.936 | 0.989 | 1.000 | 1192 | tags=16%, list=10%, signal=18% |
| 1704 | GOMF\_ACETYL\_COA\_C\_ACYLTRANSFERASE\_ACTIVITY |  | 6 | 0.29 | 0.68 | 0.877 | 0.989 | 1.000 | 1648 | tags=33%, list=14%, signal=39% |
| 1705 | GOBP\_NEGATIVE\_REGULATION\_OF\_NITRIC\_OXIDE\_SYNTHASE\_BIOSYNTHETIC\_PROCESS |  | 3 | 0.38 | 0.68 | 0.864 | 0.989 | 1.000 | 7490 | tags=100%, list=62%, signal=266% |
| 1706 | GOBP\_ANTIVIRAL\_INNATE\_IMMUNE\_RESPONSE |  | 3 | 0.38 | 0.68 | 0.856 | 0.989 | 1.000 | 7469 | tags=100%, list=62%, signal=264% |
| 1707 | GOBP\_REGULATION\_OF\_DEFENSE\_RESPONSE\_TO\_BACTERIUM |  | 8 | 0.26 | 0.68 | 0.867 | 0.988 | 1.000 | 1413 | tags=25%, list=12%, signal=28% |
| 1708 | GOBP\_HEART\_FORMATION |  | 20 | 0.19 | 0.68 | 0.923 | 0.989 | 1.000 | 1723 | tags=25%, list=14%, signal=29% |
| 1709 | GOBP\_CARDIAC\_MUSCLE\_TISSUE\_REGENERATION |  | 4 | 0.34 | 0.68 | 0.860 | 0.989 | 1.000 | 843 | tags=25%, list=7%, signal=27% |
| 1710 | GOBP\_NORADRENERGIC\_NEURON\_DIFFERENTIATION |  | 4 | 0.34 | 0.68 | 0.854 | 0.989 | 1.000 | 584 | tags=25%, list=5%, signal=26% |
| 1711 | GOBP\_ALDOSTERONE\_METABOLIC\_PROCESS |  | 6 | 0.29 | 0.68 | 0.863 | 0.988 | 1.000 | 134 | tags=17%, list=1%, signal=17% |
| 1712 | GOCC\_TRIGLYCERIDE\_RICH\_PLASMA\_LIPOPROTEIN\_PARTICLE |  | 9 | 0.25 | 0.68 | 0.881 | 0.988 | 1.000 | 1445 | tags=22%, list=12%, signal=25% |
| 1713 | GOCC\_VCB\_COMPLEX |  | 3 | 0.37 | 0.68 | 0.858 | 0.988 | 1.000 | 729 | tags=33%, list=6%, signal=35% |
| 1714 | GOCC\_INTRINSIC\_COMPONENT\_OF\_THE\_CYTOPLASMIC\_SIDE\_OF\_THE\_PLASMA\_MEMBRANE |  | 4 | 0.34 | 0.68 | 0.866 | 0.988 | 1.000 | 946 | tags=25%, list=8%, signal=27% |
| 1715 | GOBP\_NEGATIVE\_REGULATION\_OF\_OXIDATIVE\_STRESS\_INDUCED\_NEURON\_INTRINSIC\_APOPTOTIC\_SIGNALING\_PATHWAY |  | 3 | 0.38 | 0.68 | 0.863 | 0.987 | 1.000 | 7441 | tags=100%, list=62%, signal=263% |
| 1716 | GOBP\_FATTY\_ACID\_BETA\_OXIDATION\_USING\_ACYL\_COA\_OXIDASE |  | 13 | 0.22 | 0.68 | 0.908 | 0.988 | 1.000 | 9387 | tags=100%, list=78%, signal=457% |
| 1717 | GOCC\_GPI\_ANCHOR\_TRANSAMIDASE\_COMPLEX |  | 4 | 0.34 | 0.68 | 0.880 | 0.989 | 1.000 | 7955 | tags=100%, list=66%, signal=296% |
| 1718 | GOCC\_INTRINSIC\_COMPONENT\_OF\_PEROXISOMAL\_MEMBRANE |  | 13 | 0.22 | 0.68 | 0.896 | 0.988 | 1.000 | 2023 | tags=31%, list=17%, signal=37% |
| 1719 | GOBP\_REGULATION\_OF\_TRANSCRIPTION\_BY\_RNA\_POLYMERASE\_I |  | 24 | 0.19 | 0.68 | 0.939 | 0.988 | 1.000 | 1038 | tags=13%, list=9%, signal=14% |
| 1720 | GOCC\_NUCLEOID |  | 33 | 0.17 | 0.67 | 0.965 | 0.989 | 1.000 | 2339 | tags=27%, list=19%, signal=34% |
| 1721 | GOBP\_DEPHOSPHORYLATION\_OF\_RNA\_POLYMERASE\_II\_C\_TERMINAL\_DOMAIN |  | 5 | 0.31 | 0.67 | 0.872 | 0.988 | 1.000 | 528 | tags=20%, list=4%, signal=21% |
| 1722 | GOCC\_MITOCHONDRIAL\_ALPHA\_KETOGLUTARATE\_DEHYDROGENASE\_COMPLEX |  | 4 | 0.33 | 0.67 | 0.865 | 0.988 | 1.000 | 733 | tags=25%, list=6%, signal=27% |
| 1723 | GOBP\_LATE\_ENDOSOME\_TO\_LYSOSOME\_TRANSPORT |  | 6 | 0.29 | 0.67 | 0.874 | 0.988 | 1.000 | 8529 | tags=100%, list=71%, signal=345% |
| 1724 | GOCC\_TRICARBOXYLIC\_ACID\_CYCLE\_ENZYME\_COMPLEX |  | 13 | 0.22 | 0.67 | 0.903 | 0.988 | 1.000 | 2550 | tags=38%, list=21%, signal=49% |
| 1725 | GOBP\_LEUCINE\_TRANSPORT |  | 3 | 0.37 | 0.67 | 0.871 | 0.987 | 1.000 | 804 | tags=33%, list=7%, signal=36% |
| 1726 | GOBP\_AZOLE\_TRANSMEMBRANE\_TRANSPORT |  | 8 | 0.26 | 0.67 | 0.891 | 0.987 | 1.000 | 2235 | tags=38%, list=19%, signal=46% |
| 1727 | GOBP\_NEGATIVE\_REGULATION\_OF\_PROTEIN\_K63\_LINKED\_UBIQUITINATION |  | 4 | 0.33 | 0.67 | 0.867 | 0.986 | 1.000 | 311 | tags=25%, list=3%, signal=26% |
| 1728 | GOBP\_MITOCHONDRIAL\_RNA\_PROCESSING |  | 15 | 0.21 | 0.67 | 0.917 | 0.986 | 1.000 | 9520 | tags=100%, list=79%, signal=482% |
| 1729 | GOBP\_POSITIVE\_REGULATION\_OF\_PHOSPHOLIPID\_BIOSYNTHETIC\_PROCESS |  | 8 | 0.26 | 0.67 | 0.894 | 0.987 | 1.000 | 1292 | tags=25%, list=11%, signal=28% |
| 1730 | GOBP\_TRNA\_PROCESSING |  | 95 | 0.13 | 0.67 | 1.000 | 0.988 | 1.000 | 2371 | tags=24%, list=20%, signal=30% |
| 1731 | GOBP\_CARBON\_CATABOLITE\_REGULATION\_OF\_TRANSCRIPTION |  | 4 | 0.33 | 0.67 | 0.877 | 0.987 | 1.000 | 8011 | tags=100%, list=67%, signal=300% |
| 1732 | GOMF\_OLIGOPEPTIDE\_TRANSMEMBRANE\_TRANSPORTER\_ACTIVITY |  | 6 | 0.28 | 0.67 | 0.880 | 0.987 | 1.000 | 1442 | tags=33%, list=12%, signal=38% |
| 1733 | GOMF\_U6\_SNRNA\_BINDING |  | 11 | 0.23 | 0.67 | 0.904 | 0.989 | 1.000 | 9293 | tags=100%, list=77%, signal=442% |
| 1734 | GOBP\_CONSTITUTIVE\_SECRETORY\_PATHWAY |  | 7 | 0.27 | 0.67 | 0.887 | 0.989 | 1.000 | 403 | tags=14%, list=3%, signal=15% |
| 1735 | GOCC\_GEMINI\_OF\_COILED\_BODIES |  | 8 | 0.26 | 0.67 | 0.906 | 0.989 | 1.000 | 8924 | tags=100%, list=74%, signal=389% |
| 1736 | GOBP\_NEGATIVE\_REGULATION\_OF\_SINGLE\_STRANDED\_VIRAL\_RNA\_REPLICATION\_VIA\_DOUBLE\_STRANDED\_DNA\_INTERMEDIATE |  | 5 | 0.30 | 0.67 | 0.891 | 0.989 | 1.000 | 8388 | tags=100%, list=70%, signal=331% |
| 1737 | GOCC\_SMN\_COMPLEX |  | 8 | 0.26 | 0.67 | 0.878 | 0.989 | 1.000 | 8924 | tags=100%, list=74%, signal=389% |
| 1738 | GOBP\_DEOXYRIBONUCLEOSIDE\_MONOPHOSPHATE\_BIOSYNTHETIC\_PROCESS |  | 7 | 0.27 | 0.67 | 0.891 | 0.988 | 1.000 | 238 | tags=14%, list=2%, signal=15% |
| 1739 | GOBP\_BLOOD\_COAGULATION\_INTRINSIC\_PATHWAY |  | 9 | 0.24 | 0.67 | 0.905 | 0.988 | 1.000 | 1306 | tags=22%, list=11%, signal=25% |
| 1740 | GOMF\_FATTY\_ACID\_DERIVATIVE\_BINDING |  | 18 | 0.20 | 0.67 | 0.945 | 0.987 | 1.000 | 1895 | tags=33%, list=16%, signal=40% |
| 1741 | GOBP\_TRIPEPTIDE\_TRANSPORT |  | 5 | 0.30 | 0.67 | 0.886 | 0.987 | 1.000 | 1442 | tags=40%, list=12%, signal=45% |
| 1742 | GOBP\_POSITIVE\_REGULATION\_OF\_RETINOIC\_ACID\_RECEPTOR\_SIGNALING\_PATHWAY |  | 4 | 0.33 | 0.67 | 0.881 | 0.986 | 1.000 | 8050 | tags=100%, list=67%, signal=303% |
| 1743 | GOBP\_REGULATION\_OF\_HEMOGLOBIN\_BIOSYNTHETIC\_PROCESS |  | 5 | 0.30 | 0.66 | 0.891 | 0.986 | 1.000 | 8365 | tags=100%, list=70%, signal=329% |
| 1744 | GOBP\_LEUKOCYTE\_TETHERING\_OR\_ROLLING |  | 19 | 0.20 | 0.66 | 0.929 | 0.986 | 1.000 | 1235 | tags=16%, list=10%, signal=18% |
| 1745 | GOMF\_THREONINE\_TYPE\_PEPTIDASE\_ACTIVITY |  | 11 | 0.23 | 0.66 | 0.913 | 0.987 | 1.000 | 3006 | tags=55%, list=25%, signal=73% |
| 1746 | GOMF\_OXIDOREDUCTASE\_ACTIVITY\_ACTING\_ON\_METAL\_IONS\_OXYGEN\_AS\_ACCEPTOR |  | 6 | 0.28 | 0.66 | 0.901 | 0.987 | 1.000 | 222 | tags=17%, list=2%, signal=17% |
| 1747 | GOBP\_PUTRESCINE\_BIOSYNTHETIC\_PROCESS |  | 5 | 0.30 | 0.66 | 0.879 | 0.987 | 1.000 | 8359 | tags=100%, list=70%, signal=329% |
| 1748 | GOBP\_OSSIFICATION\_INVOLVED\_IN\_BONE\_REMODELING |  | 3 | 0.36 | 0.66 | 0.907 | 0.987 | 1.000 | 822 | tags=33%, list=7%, signal=36% |
| 1749 | GOBP\_MYOFIBROBLAST\_DIFFERENTIATION |  | 5 | 0.30 | 0.66 | 0.880 | 0.987 | 1.000 | 839 | tags=20%, list=7%, signal=21% |
| 1750 | GOBP\_RESPONSE\_TO\_GRANULOCYTE\_MACROPHAGE\_COLONY\_STIMULATING\_FACTOR |  | 5 | 0.30 | 0.66 | 0.895 | 0.987 | 1.000 | 8399 | tags=100%, list=70%, signal=332% |
| 1751 | GOBP\_NEGATIVE\_REGULATION\_OF\_PROTEIN\_POLYUBIQUITINATION |  | 6 | 0.28 | 0.66 | 0.904 | 0.987 | 1.000 | 2222 | tags=50%, list=18%, signal=61% |
| 1752 | GOBP\_L\_SERINE\_TRANSPORT |  | 4 | 0.33 | 0.66 | 0.886 | 0.988 | 1.000 | 2597 | tags=50%, list=22%, signal=64% |
| 1753 | GOBP\_RECEPTOR\_LOCALIZATION\_TO\_NON\_MOTILE\_CILIUM |  | 4 | 0.32 | 0.66 | 0.904 | 0.989 | 1.000 | 8123 | tags=100%, list=68%, signal=309% |
| 1754 | GOBP\_PYRIMIDINE\_RIBONUCLEOSIDE\_MONOPHOSPHATE\_METABOLIC\_PROCESS |  | 13 | 0.21 | 0.65 | 0.918 | 0.990 | 1.000 | 346 | tags=8%, list=3%, signal=8% |
| 1755 | GOMF\_C\_PALMITOYLTRANSFERASE\_ACTIVITY |  | 4 | 0.32 | 0.65 | 0.893 | 0.991 | 1.000 | 462 | tags=25%, list=4%, signal=26% |
| 1756 | GOMF\_TRANSLATION\_FACTOR\_ACTIVITY\_RNA\_BINDING |  | 67 | 0.14 | 0.65 | 0.983 | 0.992 | 1.000 | 3089 | tags=33%, list=26%, signal=44% |
| 1757 | GOMF\_L\_SERINE\_TRANSMEMBRANE\_TRANSPORTER\_ACTIVITY |  | 4 | 0.33 | 0.65 | 0.892 | 0.991 | 1.000 | 2597 | tags=50%, list=22%, signal=64% |
| 1758 | GOMF\_PEPTIDE\_DISULFIDE\_OXIDOREDUCTASE\_ACTIVITY |  | 11 | 0.22 | 0.65 | 0.935 | 0.992 | 1.000 | 9354 | tags=100%, list=78%, signal=452% |
| 1759 | GOCC\_PHOTORECEPTOR\_RIBBON\_SYNAPSE |  | 4 | 0.32 | 0.65 | 0.891 | 0.992 | 1.000 | 539 | tags=25%, list=4%, signal=26% |
| 1760 | GOBP\_REGULATION\_OF\_SYSTEMIC\_ARTERIAL\_BLOOD\_PRESSURE\_BY\_ENDOTHELIN |  | 4 | 0.33 | 0.65 | 0.901 | 0.991 | 1.000 | 8091 | tags=100%, list=67%, signal=306% |
| 1761 | GOBP\_FORMATION\_OF\_CYTOPLASMIC\_TRANSLATION\_INITIATION\_COMPLEX |  | 13 | 0.21 | 0.65 | 0.931 | 0.992 | 1.000 | 2782 | tags=38%, list=23%, signal=50% |
| 1762 | GOMF\_DRUG\_BINDING |  | 41 | 0.15 | 0.65 | 0.981 | 0.991 | 1.000 | 1942 | tags=22%, list=16%, signal=26% |
| 1763 | GOBP\_CELL\_DIFFERENTIATION\_INVOLVED\_IN\_METANEPHROS\_DEVELOPMENT |  | 16 | 0.20 | 0.65 | 0.934 | 0.991 | 1.000 | 103 | tags=6%, list=1%, signal=6% |
| 1764 | GOBP\_NEGATIVE\_REGULATION\_OF\_NEURON\_MIGRATION |  | 6 | 0.28 | 0.65 | 0.905 | 0.993 | 1.000 | 431 | tags=17%, list=4%, signal=17% |
| 1765 | GOMF\_PURINE\_RIBONUCLEOTIDE\_TRANSMEMBRANE\_TRANSPORTER\_ACTIVITY |  | 12 | 0.21 | 0.65 | 0.932 | 0.992 | 1.000 | 276 | tags=8%, list=2%, signal=9% |
| 1766 | GOBP\_NOTOCHORD\_MORPHOGENESIS |  | 5 | 0.29 | 0.65 | 0.906 | 0.992 | 1.000 | 752 | tags=20%, list=6%, signal=21% |
| 1767 | GOBP\_REGULATION\_OF\_ADENYLATE\_CYCLASE\_ACTIVATING\_G\_PROTEIN\_COUPLED\_RECEPTOR\_SIGNALING\_PATHWAY |  | 6 | 0.28 | 0.65 | 0.922 | 0.991 | 1.000 | 8704 | tags=100%, list=72%, signal=363% |
| 1768 | GOBP\_ASCENDING\_AORTA\_DEVELOPMENT |  | 5 | 0.30 | 0.65 | 0.913 | 0.991 | 1.000 | 1954 | tags=40%, list=16%, signal=48% |
| 1769 | GOBP\_POSITIVE\_REGULATION\_OF\_UTERINE\_SMOOTH\_MUSCLE\_CONTRACTION |  | 3 | 0.35 | 0.64 | 0.896 | 0.991 | 1.000 | 2698 | tags=67%, list=22%, signal=86% |
| 1770 | GOCC\_SMN\_SM\_PROTEIN\_COMPLEX |  | 14 | 0.20 | 0.64 | 0.940 | 0.990 | 1.000 | 2294 | tags=36%, list=19%, signal=44% |
| 1771 | GOBP\_KETONE\_BODY\_METABOLIC\_PROCESS |  | 7 | 0.26 | 0.64 | 0.906 | 0.991 | 1.000 | 8865 | tags=100%, list=74%, signal=382% |
| 1772 | GOBP\_FARNESYL\_DIPHOSPHATE\_METABOLIC\_PROCESS |  | 4 | 0.32 | 0.64 | 0.908 | 0.991 | 1.000 | 714 | tags=25%, list=6%, signal=27% |
| 1773 | GOBP\_ADENOSINE\_TO\_INOSINE\_EDITING |  | 4 | 0.32 | 0.64 | 0.908 | 0.991 | 1.000 | 1017 | tags=25%, list=8%, signal=27% |
| 1774 | GOBP\_PEPTIDYL\_PROLINE\_MODIFICATION |  | 45 | 0.15 | 0.64 | 0.985 | 0.991 | 1.000 | 1175 | tags=11%, list=10%, signal=12% |
| 1775 | GOBP\_POSITIVE\_REGULATION\_OF\_IMMATURE\_T\_CELL\_PROLIFERATION |  | 3 | 0.35 | 0.64 | 0.900 | 0.990 | 1.000 | 1237 | tags=33%, list=10%, signal=37% |
| 1776 | GOBP\_REGULATION\_OF\_PEPTIDYL\_SERINE\_PHOSPHORYLATION\_OF\_STAT\_PROTEIN |  | 4 | 0.32 | 0.64 | 0.909 | 0.990 | 1.000 | 506 | tags=25%, list=4%, signal=26% |
| 1777 | GOCC\_GLYCOGEN\_GRANULE |  | 6 | 0.27 | 0.64 | 0.911 | 0.992 | 1.000 | 8729 | tags=100%, list=73%, signal=366% |
| 1778 | GOBP\_REGULATION\_OF\_MICROTUBULE\_BINDING |  | 8 | 0.24 | 0.64 | 0.923 | 0.991 | 1.000 | 1213 | tags=25%, list=10%, signal=28% |
| 1779 | GOBP\_DNA\_PROTECTION |  | 4 | 0.32 | 0.64 | 0.924 | 0.991 | 1.000 | 937 | tags=25%, list=8%, signal=27% |
| 1780 | GOBP\_D\_AMINO\_ACID\_TRANSPORT |  | 5 | 0.29 | 0.64 | 0.920 | 0.991 | 1.000 | 432 | tags=20%, list=4%, signal=21% |
| 1781 | GOBP\_NEGATIVE\_REGULATION\_OF\_MEMBRANE\_POTENTIAL |  | 9 | 0.23 | 0.64 | 0.932 | 0.991 | 1.000 | 1146 | tags=22%, list=10%, signal=25% |
| 1782 | GOBP\_NUCLEAR\_RETENTION\_OF\_PRE\_MRNA\_AT\_THE\_SITE\_OF\_TRANSCRIPTION |  | 5 | 0.29 | 0.64 | 0.909 | 0.991 | 1.000 | 8526 | tags=100%, list=71%, signal=345% |
| 1783 | GOBP\_SUBSTRATE\_DEPENDENT\_CELL\_MIGRATION\_CELL\_EXTENSION |  | 7 | 0.26 | 0.64 | 0.924 | 0.991 | 1.000 | 1340 | tags=29%, list=11%, signal=32% |
| 1784 | GOBP\_NEGATIVE\_REGULATION\_OF\_EXTRACELLULAR\_MATRIX\_DISASSEMBLY |  | 4 | 0.31 | 0.63 | 0.918 | 0.991 | 1.000 | 721 | tags=25%, list=6%, signal=27% |
| 1785 | GOMF\_RNA\_POLYMERASE\_ACTIVITY |  | 30 | 0.16 | 0.63 | 0.969 | 0.991 | 1.000 | 2334 | tags=30%, list=19%, signal=37% |
| 1786 | GOBP\_POSITIVE\_REGULATION\_OF\_PATHWAY\_RESTRICTED\_SMAD\_PROTEIN\_PHOSPHORYLATION |  | 32 | 0.16 | 0.63 | 0.970 | 0.990 | 1.000 | 134 | tags=6%, list=1%, signal=6% |
| 1787 | GOBP\_SEQUESTERING\_OF\_EXTRACELLULAR\_LIGAND\_FROM\_RECEPTOR |  | 7 | 0.26 | 0.63 | 0.920 | 0.990 | 1.000 | 8948 | tags=100%, list=74%, signal=392% |
| 1788 | GOMF\_MYRISTOYLTRANSFERASE\_ACTIVITY |  | 4 | 0.32 | 0.63 | 0.912 | 0.990 | 1.000 | 8223 | tags=100%, list=68%, signal=317% |
| 1789 | GOBP\_CELLULAR\_RESPONSE\_TO\_PURINE\_CONTAINING\_COMPOUND |  | 10 | 0.22 | 0.63 | 0.927 | 0.990 | 1.000 | 339 | tags=10%, list=3%, signal=10% |
| 1790 | GOBP\_POSITIVE\_REGULATION\_OF\_PEPTIDYL\_THREONINE\_PHOSPHORYLATION |  | 20 | 0.18 | 0.63 | 0.967 | 0.990 | 1.000 | 171 | tags=5%, list=1%, signal=5% |
| 1791 | GOBP\_ASPARAGINE\_METABOLIC\_PROCESS |  | 5 | 0.29 | 0.63 | 0.910 | 0.990 | 1.000 | 8538 | tags=100%, list=71%, signal=346% |
| 1792 | GOMF\_PEPTIDOGLYCAN\_BINDING |  | 6 | 0.27 | 0.63 | 0.934 | 0.990 | 1.000 | 361 | tags=17%, list=3%, signal=17% |
| 1793 | GOBP\_NEGATIVE\_REGULATION\_OF\_SECONDARY\_METABOLITE\_BIOSYNTHETIC\_PROCESS |  | 4 | 0.31 | 0.63 | 0.918 | 0.989 | 1.000 | 8289 | tags=100%, list=69%, signal=323% |
| 1794 | GOCC\_MITOCHONDRIAL\_TRICARBOXYLIC\_ACID\_CYCLE\_ENZYME\_COMPLEX |  | 5 | 0.28 | 0.63 | 0.927 | 0.989 | 1.000 | 733 | tags=20%, list=6%, signal=21% |
| 1795 | GOBP\_NEGATIVE\_REGULATION\_OF\_SYNAPSE\_ORGANIZATION |  | 6 | 0.27 | 0.63 | 0.933 | 0.989 | 1.000 | 822 | tags=17%, list=7%, signal=18% |
| 1796 | GOMF\_RNA\_POLYMERASE\_II\_CTD\_HEPTAPEPTIDE\_REPEAT\_PHOSPHATASE\_ACTIVITY |  | 6 | 0.27 | 0.63 | 0.921 | 0.989 | 1.000 | 528 | tags=17%, list=4%, signal=17% |
| 1797 | GOBP\_CELLULAR\_RESPONSE\_TO\_BRAIN\_DERIVED\_NEUROTROPHIC\_FACTOR\_STIMULUS |  | 9 | 0.23 | 0.63 | 0.944 | 0.989 | 1.000 | 9233 | tags=100%, list=77%, signal=432% |
| 1798 | GOCC\_RIBBON\_SYNAPSE |  | 6 | 0.27 | 0.63 | 0.931 | 0.989 | 1.000 | 539 | tags=17%, list=4%, signal=17% |
| 1799 | GOBP\_GLYCOSYL\_COMPOUND\_BIOSYNTHETIC\_PROCESS |  | 33 | 0.16 | 0.63 | 0.979 | 0.989 | 1.000 | 463 | tags=6%, list=4%, signal=6% |
| 1800 | GOBP\_SERINE\_PHOSPHORYLATION\_OF\_STAT\_PROTEIN |  | 7 | 0.25 | 0.63 | 0.934 | 0.989 | 1.000 | 2482 | tags=43%, list=21%, signal=54% |
| 1801 | GOBP\_DNA\_INTEGRATION |  | 4 | 0.31 | 0.62 | 0.900 | 0.990 | 1.000 | 8295 | tags=100%, list=69%, signal=323% |
| 1802 | GOCC\_EXON\_EXON\_JUNCTION\_COMPLEX |  | 17 | 0.19 | 0.62 | 0.946 | 0.989 | 1.000 | 7219 | tags=94%, list=60%, signal=236% |
| 1803 | GOBP\_POSITIVE\_REGULATION\_OF\_PHOSPHOLIPID\_METABOLIC\_PROCESS |  | 10 | 0.22 | 0.62 | 0.940 | 0.990 | 1.000 | 1292 | tags=20%, list=11%, signal=22% |
| 1804 | GOBP\_S\_ADENOSYLMETHIONINE\_METABOLIC\_PROCESS |  | 14 | 0.20 | 0.62 | 0.955 | 0.990 | 1.000 | 1092 | tags=14%, list=9%, signal=16% |
| 1805 | GOMF\_PHOSPHOTRANSFERASE\_ACTIVITY\_FOR\_OTHER\_SUBSTITUTED\_PHOSPHATE\_GROUPS |  | 18 | 0.18 | 0.62 | 0.962 | 0.990 | 1.000 | 9823 | tags=100%, list=82%, signal=548% |
| 1806 | GOMF\_ANKYRIN\_REPEAT\_BINDING |  | 4 | 0.31 | 0.62 | 0.928 | 0.990 | 1.000 | 8290 | tags=100%, list=69%, signal=323% |
| 1807 | GOCC\_GOLGI\_TRANS\_CISTERNA |  | 10 | 0.22 | 0.62 | 0.942 | 0.990 | 1.000 | 9391 | tags=100%, list=78%, signal=458% |
| 1808 | GOBP\_PYRIMIDINE\_RIBONUCLEOSIDE\_TRIPHOSPHATE\_METABOLIC\_PROCESS |  | 14 | 0.20 | 0.62 | 0.958 | 0.990 | 1.000 | 1836 | tags=21%, list=15%, signal=25% |
| 1809 | GOBP\_MITOCHONDRIAL\_TRNA\_PROCESSING |  | 12 | 0.21 | 0.62 | 0.943 | 0.989 | 1.000 | 9520 | tags=100%, list=79%, signal=482% |
| 1810 | GOBP\_CDC42\_PROTEIN\_SIGNAL\_TRANSDUCTION |  | 9 | 0.23 | 0.62 | 0.949 | 0.990 | 1.000 | 1028 | tags=22%, list=9%, signal=24% |
| 1811 | GOMF\_NERVE\_GROWTH\_FACTOR\_BINDING |  | 4 | 0.31 | 0.62 | 0.930 | 0.989 | 1.000 | 1188 | tags=25%, list=10%, signal=28% |
| 1812 | GOBP\_MITOCHONDRIAL\_RNA\_METABOLIC\_PROCESS |  | 37 | 0.15 | 0.62 | 0.990 | 0.989 | 1.000 | 2723 | tags=30%, list=23%, signal=38% |
| 1813 | GOBP\_VALINE\_METABOLIC\_PROCESS |  | 6 | 0.26 | 0.62 | 0.931 | 0.989 | 1.000 | 572 | tags=17%, list=5%, signal=17% |
| 1814 | GOBP\_ADENYLATE\_CYCLASE\_ACTIVATING\_ADRENERGIC\_RECEPTOR\_SIGNALING\_PATHWAY |  | 16 | 0.19 | 0.62 | 0.961 | 0.988 | 1.000 | 2740 | tags=44%, list=23%, signal=57% |
| 1815 | GOMF\_COA\_HYDROLASE\_ACTIVITY |  | 16 | 0.19 | 0.62 | 0.959 | 0.989 | 1.000 | 9774 | tags=100%, list=81%, signal=536% |
| 1816 | GOBP\_PHOSPHATIDYLCHOLINE\_CATABOLIC\_PROCESS |  | 10 | 0.22 | 0.62 | 0.940 | 0.988 | 1.000 | 2572 | tags=40%, list=21%, signal=51% |
| 1817 | GOBP\_KETONE\_BODY\_BIOSYNTHETIC\_PROCESS |  | 6 | 0.26 | 0.61 | 0.927 | 0.990 | 1.000 | 8865 | tags=100%, list=74%, signal=382% |
| 1818 | GOBP\_GLYCOSIDE\_METABOLIC\_PROCESS |  | 12 | 0.20 | 0.61 | 0.957 | 0.989 | 1.000 | 1464 | tags=25%, list=12%, signal=28% |
| 1819 | GOBP\_POST\_EMBRYONIC\_ANIMAL\_ORGAN\_MORPHOGENESIS |  | 5 | 0.28 | 0.61 | 0.937 | 0.989 | 1.000 | 8676 | tags=100%, list=72%, signal=360% |
| 1820 | GOBP\_POSITIVE\_REGULATION\_OF\_ESTABLISHMENT\_OF\_PROTEIN\_LOCALIZATION\_TO\_MITOCHONDRION |  | 44 | 0.14 | 0.61 | 0.989 | 0.989 | 1.000 | 2260 | tags=23%, list=19%, signal=28% |
| 1821 | GOMF\_POLY\_PYRIMIDINE\_TRACT\_BINDING |  | 22 | 0.17 | 0.61 | 0.972 | 0.989 | 1.000 | 113 | tags=5%, list=1%, signal=5% |
| 1822 | GOCC\_AP\_4\_ADAPTOR\_COMPLEX |  | 3 | 0.34 | 0.61 | 0.935 | 0.989 | 1.000 | 809 | tags=33%, list=7%, signal=36% |
| 1823 | GOBP\_INTRINSIC\_APOPTOTIC\_SIGNALING\_PATHWAY\_IN\_RESPONSE\_TO\_HYPOXIA |  | 4 | 0.30 | 0.61 | 0.942 | 0.989 | 1.000 | 577 | tags=25%, list=5%, signal=26% |
| 1824 | GOBP\_RETROGRADE\_TRANSPORT\_ENDOSOME\_TO\_PLASMA\_MEMBRANE |  | 20 | 0.17 | 0.61 | 0.970 | 0.989 | 1.000 | 1328 | tags=15%, list=11%, signal=17% |
| 1825 | GOBP\_REGULATION\_OF\_PROTEIN\_TARGETING\_TO\_MITOCHONDRION |  | 33 | 0.15 | 0.61 | 0.988 | 0.988 | 1.000 | 1082 | tags=12%, list=9%, signal=13% |
| 1826 | GOBP\_REGULATION\_OF\_ENDOPLASMIC\_RETICULUM\_TUBULAR\_NETWORK\_ORGANIZATION |  | 5 | 0.28 | 0.61 | 0.943 | 0.988 | 1.000 | 8663 | tags=100%, list=72%, signal=359% |
| 1827 | GOMF\_UBIQUITIN\_PROTEIN\_TRANSFERASE\_INHIBITOR\_ACTIVITY |  | 6 | 0.26 | 0.61 | 0.944 | 0.988 | 1.000 | 8910 | tags=100%, list=74%, signal=387% |
| 1828 | GOBP\_DEVELOPMENT\_OF\_SECONDARY\_SEXUAL\_CHARACTERISTICS |  | 4 | 0.30 | 0.60 | 0.940 | 0.989 | 1.000 | 8397 | tags=100%, list=70%, signal=332% |
| 1829 | GOBP\_REVERSE\_CHOLESTEROL\_TRANSPORT |  | 8 | 0.23 | 0.60 | 0.951 | 0.989 | 1.000 | 9260 | tags=100%, list=77%, signal=436% |
| 1830 | GOBP\_PYRIMIDINE\_NUCLEOSIDE\_CATABOLIC\_PROCESS |  | 13 | 0.20 | 0.60 | 0.957 | 0.989 | 1.000 | 346 | tags=8%, list=3%, signal=8% |
| 1831 | GOCC\_PHOTORECEPTOR\_DISC\_MEMBRANE |  | 5 | 0.28 | 0.60 | 0.946 | 0.989 | 1.000 | 8691 | tags=100%, list=72%, signal=362% |
| 1832 | GOBP\_CELLULAR\_RESPONSE\_TO\_SODIUM\_ARSENITE |  | 3 | 0.33 | 0.60 | 0.943 | 0.988 | 1.000 | 8044 | tags=100%, list=67%, signal=303% |
| 1833 | GOBP\_CELLULAR\_ANION\_HOMEOSTASIS |  | 8 | 0.23 | 0.60 | 0.944 | 0.989 | 1.000 | 1482 | tags=25%, list=12%, signal=28% |
| 1834 | GOBP\_RESPONSE\_TO\_SODIUM\_ARSENITE |  | 3 | 0.33 | 0.60 | 0.955 | 0.990 | 1.000 | 8044 | tags=100%, list=67%, signal=303% |
| 1835 | GOMF\_ENONE\_REDUCTASE\_ACTIVITY |  | 4 | 0.30 | 0.60 | 0.945 | 0.990 | 1.000 | 637 | tags=25%, list=5%, signal=26% |
| 1836 | GOBP\_MINERALOCORTICOID\_SECRETION |  | 5 | 0.27 | 0.60 | 0.942 | 0.990 | 1.000 | 8743 | tags=100%, list=73%, signal=367% |
| 1837 | GOBP\_REGULATION\_OF\_SOMATIC\_STEM\_CELL\_POPULATION\_MAINTENANCE |  | 4 | 0.29 | 0.59 | 0.951 | 0.993 | 1.000 | 8489 | tags=100%, list=71%, signal=341% |
| 1838 | GOMF\_TRNA\_BINDING |  | 49 | 0.14 | 0.59 | 0.995 | 0.993 | 1.000 | 9672 | tags=98%, list=81%, signal=501% |
| 1839 | GOBP\_NEGATIVE\_REGULATION\_OF\_INSULIN\_SECRETION\_INVOLVED\_IN\_CELLULAR\_RESPONSE\_TO\_GLUCOSE\_STIMULUS |  | 5 | 0.27 | 0.59 | 0.944 | 0.992 | 1.000 | 8773 | tags=100%, list=73%, signal=371% |
| 1840 | GOBP\_CELLULAR\_RESPONSE\_TO\_SALT |  | 3 | 0.33 | 0.59 | 0.947 | 0.993 | 1.000 | 8044 | tags=100%, list=67%, signal=303% |
| 1841 | GOCC\_EUKARYOTIC\_48S\_PREINITIATION\_COMPLEX |  | 12 | 0.20 | 0.59 | 0.962 | 0.992 | 1.000 | 9642 | tags=100%, list=80%, signal=507% |
| 1842 | GOBP\_PRESYNAPTIC\_MODULATION\_OF\_CHEMICAL\_SYNAPTIC\_TRANSMISSION |  | 8 | 0.22 | 0.59 | 0.962 | 0.993 | 1.000 | 2201 | tags=38%, list=18%, signal=46% |
| 1843 | GOBP\_REGULATION\_OF\_THE\_FORCE\_OF\_HEART\_CONTRACTION\_BY\_CHEMICAL\_SIGNAL |  | 3 | 0.33 | 0.59 | 0.953 | 0.993 | 1.000 | 8091 | tags=100%, list=67%, signal=306% |
| 1844 | GOBP\_CARDIAC\_MUSCLE\_FIBER\_DEVELOPMENT |  | 5 | 0.27 | 0.58 | 0.958 | 0.994 | 1.000 | 8816 | tags=100%, list=73%, signal=376% |
| 1845 | GOBP\_DNA\_DAMAGE\_RESPONSE\_DETECTION\_OF\_DNA\_DAMAGE |  | 34 | 0.14 | 0.58 | 0.997 | 0.994 | 1.000 | 1692 | tags=21%, list=14%, signal=24% |
| 1846 | GOBP\_CELLULAR\_RESPONSE\_TO\_UV\_C |  | 3 | 0.32 | 0.58 | 0.958 | 0.994 | 1.000 | 1279 | tags=33%, list=11%, signal=37% |
| 1847 | GOBP\_PHOSPHATIDYLGLYCEROL\_BIOSYNTHETIC\_PROCESS |  | 9 | 0.22 | 0.58 | 0.965 | 0.994 | 1.000 | 2411 | tags=44%, list=20%, signal=56% |
| 1848 | GOBP\_NEGATIVE\_REGULATION\_OF\_ACTIN\_FILAMENT\_POLYMERIZATION |  | 44 | 0.14 | 0.57 | 0.992 | 0.996 | 1.000 | 2621 | tags=30%, list=22%, signal=38% |
| 1849 | GOMF\_CYSTEINE\_TYPE\_ENDOPEPTIDASE\_ACTIVITY\_INVOLVED\_IN\_APOPTOTIC\_SIGNALING\_PATHWAY |  | 6 | 0.25 | 0.57 | 0.973 | 0.996 | 1.000 | 514 | tags=17%, list=4%, signal=17% |
| 1850 | GOMF\_TRANSLATION\_REGULATOR\_ACTIVITY\_NUCLEIC\_ACID\_BINDING |  | 80 | 0.12 | 0.57 | 1.000 | 0.996 | 1.000 | 2782 | tags=26%, list=23%, signal=34% |
| 1851 | GOBP\_MODULATION\_BY\_VIRUS\_OF\_HOST\_CELLULAR\_PROCESS |  | 15 | 0.18 | 0.57 | 0.983 | 0.997 | 1.000 | 568 | tags=13%, list=5%, signal=14% |
| 1852 | GOBP\_GLIAL\_CELL\_FATE\_COMMITMENT |  | 5 | 0.26 | 0.57 | 0.969 | 0.997 | 1.000 | 2085 | tags=40%, list=17%, signal=48% |
| 1853 | GOBP\_CHAPERONE\_MEDIATED\_PROTEIN\_COMPLEX\_ASSEMBLY |  | 16 | 0.17 | 0.56 | 0.981 | 0.998 | 1.000 | 10002 | tags=100%, list=83%, signal=597% |
| 1854 | GOBP\_MICROGLIAL\_CELL\_PROLIFERATION |  | 5 | 0.26 | 0.56 | 0.968 | 0.998 | 1.000 | 8919 | tags=100%, list=74%, signal=388% |
| 1855 | GOCC\_NUCLEAR\_PORE\_CENTRAL\_TRANSPORT\_CHANNEL |  | 4 | 0.28 | 0.56 | 0.962 | 0.997 | 1.000 | 8639 | tags=100%, list=72%, signal=356% |
| 1856 | GOBP\_CHIASMA\_ASSEMBLY |  | 3 | 0.31 | 0.56 | 0.974 | 0.997 | 1.000 | 8311 | tags=100%, list=69%, signal=325% |
| 1857 | GOMF\_CYCLOSPORIN\_A\_BINDING |  | 13 | 0.18 | 0.56 | 0.979 | 0.997 | 1.000 | 2540 | tags=31%, list=21%, signal=39% |
| 1858 | GOBP\_REGULATION\_OF\_CELL\_PROLIFERATION\_INVOLVED\_IN\_KIDNEY\_DEVELOPMENT |  | 10 | 0.20 | 0.56 | 0.978 | 0.998 | 1.000 | 9638 | tags=100%, list=80%, signal=506% |
| 1859 | GOBP\_REGULATION\_OF\_COLLAGEN\_FIBRIL\_ORGANIZATION |  | 4 | 0.28 | 0.56 | 0.974 | 0.997 | 1.000 | 8678 | tags=100%, list=72%, signal=360% |
| 1860 | GOCC\_PRERIBOSOME\_SMALL\_SUBUNIT\_PRECURSOR |  | 13 | 0.18 | 0.55 | 0.986 | 0.998 | 1.000 | 1472 | tags=23%, list=12%, signal=26% |
| 1861 | GOMF\_IMMUNOGLOBULIN\_BINDING |  | 6 | 0.24 | 0.55 | 0.981 | 0.998 | 1.000 | 9176 | tags=100%, list=76%, signal=423% |
| 1862 | GOMF\_GDP\_BINDING |  | 60 | 0.12 | 0.55 | 1.000 | 0.997 | 1.000 | 736 | tags=7%, list=6%, signal=7% |
| 1863 | GOMF\_TYPE\_II\_TRANSFORMING\_GROWTH\_FACTOR\_BETA\_RECEPTOR\_BINDING |  | 7 | 0.22 | 0.55 | 0.974 | 0.998 | 1.000 | 1954 | tags=29%, list=16%, signal=34% |
| 1864 | GOBP\_NEGATIVE\_REGULATION\_OF\_CALCIUM\_ION\_TRANSPORT\_INTO\_CYTOSOL |  | 11 | 0.19 | 0.55 | 0.987 | 0.998 | 1.000 | 2446 | tags=36%, list=20%, signal=46% |
| 1865 | GOBP\_PHOSPHOLIPASE\_C\_ACTIVATING\_DOPAMINE\_RECEPTOR\_SIGNALING\_PATHWAY |  | 4 | 0.27 | 0.55 | 0.971 | 0.998 | 1.000 | 670 | tags=25%, list=6%, signal=26% |
| 1866 | GOBP\_B\_1\_B\_CELL\_DIFFERENTIATION |  | 3 | 0.30 | 0.54 | 0.981 | 1.000 | 1.000 | 982 | tags=33%, list=8%, signal=36% |
| 1867 | GOBP\_CELL\_PROLIFERATION\_IN\_MIDBRAIN |  | 5 | 0.25 | 0.54 | 0.980 | 0.999 | 1.000 | 1130 | tags=20%, list=9%, signal=22% |
| 1868 | GOBP\_ENDOSOME\_TO\_LYSOSOME\_TRANSPORT\_VIA\_MULTIVESICULAR\_BODY\_SORTING\_PATHWAY |  | 6 | 0.23 | 0.54 | 0.983 | 0.999 | 1.000 | 9238 | tags=100%, list=77%, signal=433% |
| 1869 | GOMF\_CERAMIDE\_1\_PHOSPHATE\_BINDING |  | 5 | 0.24 | 0.54 | 0.984 | 0.999 | 1.000 | 9086 | tags=100%, list=76%, signal=410% |
| 1870 | GOMF\_OXIDOREDUCTASE\_ACTIVITY\_ACTING\_ON\_PAIRED\_DONORS\_WITH\_OXIDATION\_OF\_A\_PAIR\_OF\_DONORS\_RESULTING\_IN\_THE\_REDUCTION\_OF\_MOLECULAR\_OXYGEN\_TO\_TWO\_MOLECULES\_OF\_WATER |  | 5 | 0.24 | 0.53 | 0.984 | 1.000 | 1.000 | 1757 | tags=40%, list=15%, signal=47% |
| 1871 | GOBP\_NEGATIVE\_REGULATION\_OF\_METALLOENDOPEPTIDASE\_ACTIVITY |  | 4 | 0.26 | 0.53 | 0.982 | 0.999 | 1.000 | 8844 | tags=100%, list=74%, signal=379% |
| 1872 | GOBP\_POSITIVE\_REGULATION\_OF\_ADIPOSE\_TISSUE\_DEVELOPMENT |  | 3 | 0.29 | 0.53 | 0.985 | 0.999 | 1.000 | 1073 | tags=33%, list=9%, signal=37% |
| 1873 | GOBP\_NEGATIVE\_REGULATION\_OF\_TRIGLYCERIDE\_CATABOLIC\_PROCESS |  | 3 | 0.29 | 0.53 | 0.981 | 0.999 | 1.000 | 8478 | tags=100%, list=71%, signal=340% |
| 1874 | GOMF\_ALPHA\_2A\_ADRENERGIC\_RECEPTOR\_BINDING |  | 3 | 0.29 | 0.53 | 0.986 | 0.998 | 1.000 | 8498 | tags=100%, list=71%, signal=342% |
| 1875 | GOCC\_AMINOACYL\_TRNA\_SYNTHETASE\_MULTIENZYME\_COMPLEX |  | 3 | 0.30 | 0.53 | 0.983 | 0.998 | 1.000 | 8444 | tags=100%, list=70%, signal=337% |
| 1876 | GOBP\_POSITIVE\_REGULATION\_OF\_DOPAMINE\_RECEPTOR\_SIGNALING\_PATHWAY |  | 3 | 0.29 | 0.53 | 0.983 | 0.998 | 1.000 | 8529 | tags=100%, list=71%, signal=345% |
| 1877 | GOMF\_ALCOHOL\_DEHYDROGENASE\_ACTIVITY\_ZINC\_DEPENDENT |  | 3 | 0.29 | 0.52 | 0.982 | 0.998 | 1.000 | 914 | tags=33%, list=8%, signal=36% |
| 1878 | GOBP\_REGULATION\_OF\_INTEGRIN\_MEDIATED\_SIGNALING\_PATHWAY |  | 12 | 0.17 | 0.52 | 0.990 | 0.999 | 1.000 | 1480 | tags=17%, list=12%, signal=19% |
| 1879 | GOCC\_JUNCTIONAL\_SARCOPLASMIC\_RETICULUM\_MEMBRANE |  | 5 | 0.24 | 0.52 | 0.988 | 0.999 | 1.000 | 9146 | tags=100%, list=76%, signal=419% |
| 1880 | GOBP\_REGULATION\_OF\_ESTABLISHMENT\_OF\_PROTEIN\_LOCALIZATION\_TO\_MITOCHONDRION |  | 53 | 0.11 | 0.51 | 0.998 | 1.000 | 1.000 | 2060 | tags=19%, list=17%, signal=23% |
| 1881 | GOBP\_PYRIMIDINE\_NUCLEOTIDE\_CATABOLIC\_PROCESS |  | 14 | 0.16 | 0.51 | 0.993 | 1.000 | 1.000 | 346 | tags=7%, list=3%, signal=7% |
| 1882 | GOBP\_POSITIVE\_REGULATION\_OF\_VASCULAR\_ENDOTHELIAL\_CELL\_PROLIFERATION |  | 12 | 0.17 | 0.51 | 0.994 | 0.999 | 1.000 | 558 | tags=8%, list=5%, signal=9% |
| 1883 | GOBP\_POSITIVE\_REGULATION\_OF\_PROTEIN\_TARGETING\_TO\_MITOCHONDRION |  | 26 | 0.14 | 0.51 | 1.000 | 0.999 | 1.000 | 1082 | tags=12%, list=9%, signal=13% |
| 1884 | GOBP\_REGULATION\_OF\_MITOCHONDRIAL\_MRNA\_STABILITY |  | 6 | 0.21 | 0.50 | 0.994 | 0.999 | 1.000 | 9468 | tags=100%, list=79%, signal=472% |
| 1885 | GOBP\_APOPTOTIC\_CHROMOSOME\_CONDENSATION |  | 3 | 0.27 | 0.50 | 0.998 | 0.999 | 1.000 | 1031 | tags=33%, list=9%, signal=36% |
| 1886 | GOBP\_PHOSPHOLIPID\_EFFLUX |  | 5 | 0.23 | 0.50 | 0.995 | 0.999 | 1.000 | 9260 | tags=100%, list=77%, signal=436% |
| 1887 | GOBP\_NEGATIVE\_REGULATION\_OF\_CELLULAR\_EXTRAVASATION |  | 4 | 0.24 | 0.50 | 0.993 | 0.999 | 1.000 | 9100 | tags=100%, list=76%, signal=412% |
| 1888 | GOBP\_METANEPHRIC\_S\_SHAPED\_BODY\_MORPHOGENESIS |  | 3 | 0.27 | 0.49 | 0.997 | 0.999 | 1.000 | 8726 | tags=100%, list=73%, signal=366% |
| 1889 | GOBP\_ETHANOL\_METABOLIC\_PROCESS |  | 8 | 0.18 | 0.48 | 0.996 | 1.000 | 1.000 | 9849 | tags=100%, list=82%, signal=555% |
| 1890 | GOBP\_REGULATION\_OF\_PROTEIN\_ADP\_RIBOSYLATION |  | 4 | 0.23 | 0.48 | 0.998 | 1.000 | 1.000 | 929 | tags=25%, list=8%, signal=27% |
| 1891 | GOMF\_LRR\_DOMAIN\_BINDING |  | 13 | 0.15 | 0.46 | 0.996 | 1.000 | 1.000 | 2102 | tags=31%, list=18%, signal=37% |
| 1892 | GOBP\_NEGATIVE\_REGULATION\_OF\_CELL\_VOLUME |  | 5 | 0.21 | 0.45 | 0.999 | 1.000 | 1.000 | 9546 | tags=100%, list=79%, signal=487% |
| 1893 | GOCC\_PRP19\_COMPLEX |  | 11 | 0.15 | 0.45 | 0.998 | 1.000 | 1.000 | 6866 | tags=91%, list=57%, signal=212% |
| 1894 | GOCC\_PRECATALYTIC\_SPLICEOSOME |  | 42 | 0.10 | 0.44 | 1.000 | 0.999 | 1.000 | 2335 | tags=26%, list=19%, signal=32% |
| 1895 | GOBP\_ORGANIC\_ACID\_METABOLIC\_PROCESS |  | 715 | 0.17 | --- | --- | 1.0 | 0.0 | 1530 | tags=18%, list=13%, signal=20% |
| 1896 | GOBP\_PEPTIDE\_METABOLIC\_PROCESS |  | 614 | 0.13468248 | --- | --- | 1.0 | 0.0 | 2783 | tags=31%, list=23%, signal=39% |
| 1897 | GOBP\_CELLULAR\_AMIDE\_METABOLIC\_PROCESS |  | 816 | 0.1311043 | --- | --- | 1.0 | 0.0 | 1929 | tags=19%, list=16%, signal=22% |
| 1898 | GOCC\_MITOCHONDRIAL\_ENVELOPE |  | 559 | 0.3347572 | --- | --- | 1.0 | 0.0 | 1867 | tags=34%, list=16%, signal=38% |
| 1899 | GOCC\_ENVELOPE |  | 885 | 0.20307937 | --- | --- | 1.0 | 0.0 | 1867 | tags=25%, list=16%, signal=28% |
| 1900 | GOCC\_RIBONUCLEOPROTEIN\_COMPLEX |  | 536 | 0.14988111 | --- | --- | 1.0 | 0.0 | 2783 | tags=32%, list=23%, signal=40% |
Table: Gene sets enriched in phenotype **1 (5 samples) **[plain text format]****
